# Comparative connectomics of two distantly related nematode species reveals patterns of nervous system evolution

**DOI:** 10.1126/science.adx2143

**Published:** 2025-07-31

**Authors:** Steven J. Cook, Cristine A. Kalinski, Curtis M. Loer, Nadin Memar, Maryam Majeed, Sarah Rebecca Stephen, Daniel J. Bumbarger, Metta Riebesell, Barbara Conradt, Ralf Schnabel, Ralf J. Sommer, Oliver Hobert

**Affiliations:** 1 Department of Biological Sciences, Columbia University; Howard Hughes Medical Institute, New York, NY 10025, USA; 2 Department of Biology, University of San Diego, San Diego, CA 92110, USA; 3 Institut für Genetik, Technische Universität Braunschweig, 38106 Braunschweig, Germany; 4 Faculty of Biology, Ludwig-Maximilians-University Munich, 82152 Planegg-Martinsried Germany; 5 Max-Planck Institute for Biology Tübingen, Department for Evolutionary Biology, 72076 Tübingen, Germany

## Abstract

Understanding the evolution of the bilaterian brain requires a detailed exploration of the precise nature of cellular and subcellular differences between related species. We undertook an electron micrographic reconstruction of the brain of the predatory nematode *Pristionchus pacificus* and compared the results with the brain of *Caenorhabditis elegans*, which diverged at least 100 million years ago. We revealed changes in neuronal cell death, neuronal cell position, axo-dendritic projection patterns and synaptic connectivity of homologous neurons that display no obvious changes in overall neurite morphology and projection patterns. This comparison of distantly related nematode species provides valuable insights for understanding evolutionary changes in the brain at multiple organizational levels.

Brains have undergone dramatic changes across animal evolution, evidenced by elaborations and increases in size, anatomical complexity and functional capacities. Due to anatomical complexity and difficulties associated with identifying homologous neurons, the precise types of evolutionary changes are often hard to pinpoint with cellular or synaptic resolution in vertebrate brains. However, in organisms with numerically more constrained and anatomically well-described brains, identifying homologous neurons is more feasible, which permits the delineation of the precise substrates of anatomical change in the brain.

The nematodes *Pristionchus pacificus* and *Caenorhabditis elegans* are thought to have diverged more than 100 million years ago (1). Although this substantial divergence is not immediately obvious based on the overall size or morphology of these species, it shows on many other levels. On a molecular level, the *P. pacificus* genome is 70% larger than the *C. elegans* genome, contains 30% more genes and only roughly 30% of its genes have 1:1 orthologs in *C. elegans* (2). The divergence of these two nematode species is also apparent in their adaptation to distinct ecological niches and in unique adaptations in sensory, locomotory and feeding behavior (3–5). The most striking adaptation is the predatory feeding behavior that *P. pacificus* displays toward other nematodes and an associated self-recognition system that prevents cannibalism against kin (6, 7).

The many cataloged differences between *P. pacificus* and *C. elegans,* particularly at behavioral level, prompt the question to what extent the nervous system of these two species has diverged. Previous studies using serial section electron microscopy described differences in the sensory periphery of these worms and the small and isolated nervous system of the foregut (“pharynx”) (8, 9). We analyzed to what extent the entire centralized brain - the cluster of head ganglia that controls many complex nematode behaviors - has diverged among these two distant nematode species. We expected that such analysis might identify and provide a panoramic view of the substrates of evolutionary change, thereby addressing several questions that only a broad, brain-wide comparative connectomic analysis can answer.

## Nanoscale reconstruction of the *P. pacificus* nervous system

**Fig. 1:**
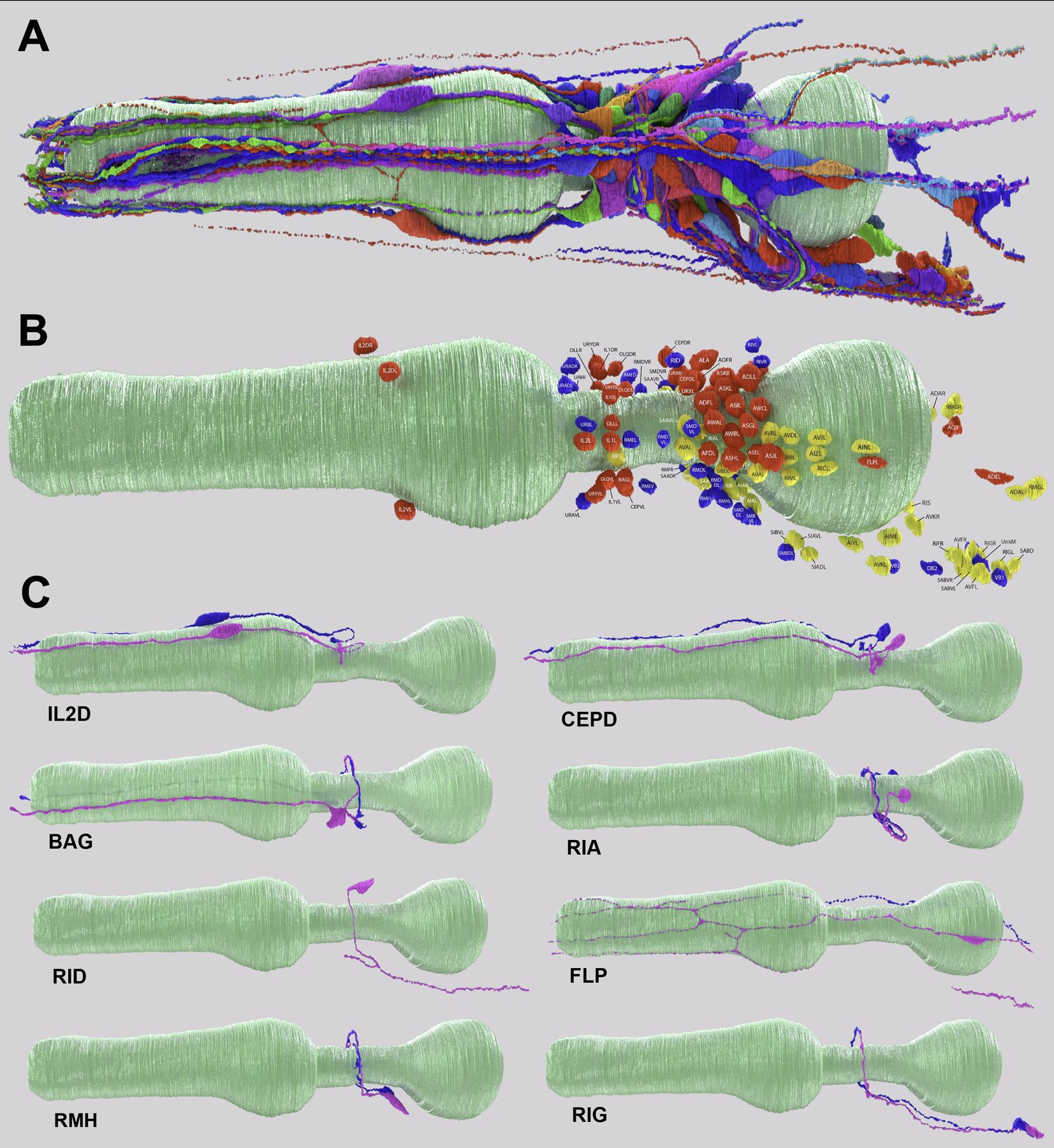
The reconstructed *Pristionchus pacificus* brain. **A:** A left lateral view 3D rendering of all *P. pacificus* neurons reconstructed from serial section electron micrographs of the head. Neurons are shown in different colors for contrast; the pharynx is shown in teal. **B:** A left lateral 3D rendering of all neuronal nuclei in the *P. pacificus* head. Nuclei are colored by neuronal function experimentally determined in nematodes: Sensory – red, interneuron – yellow, motor – blue; the pharynx is shown in teal. **C:** Left lateral views of easily-homologized *P. pacificus* neurons, including: IL2D, CEPD, BAG, RIA, RID, FLP, RMH, and RIG. Left and right homologous neurons are shown in magenta and blue, respectively, and the pharynx is shown in teal.

We analyzed serial section electron microscopy of two adult hermaphrodite *P. pacificus* heads from the anterior nose tip to the retrovesicular ganglion, including the nerve ring, the major neuropil of nematodes (Figure 1A). We reconstructed the ultrastructure of all neurons in these two animals, analyzed the extent of the membrane contacts between their neurites (the “contactome”) (Supplementary Data S1, S2) and their patterns of chemical synaptic connectivity (“connectome”) (Supplementary Data S3, S4). Based on remarkably well-conserved cell body position as well as characteristic features of neurite trajectories and placement within fascicles, we were able to assign homology to all neurons found in the *C. elegans* head ganglia (Figure 1B, 1C; renderings of all neurons are shown in Supplementary Data S5). Such clear one-to-one homology assignments are difficult to make upon comparison of more complex nervous systems and present a unique advantage of the nematode phylum with its limited cellular complexity and apparent stereotypy of cellular composition and lineage. These neuronal homology assignments allowed us to ask if - and to what extent - homologous neurons have evolved distinct features.

## Differences in neuronal composition and location

**Fig. 2:**
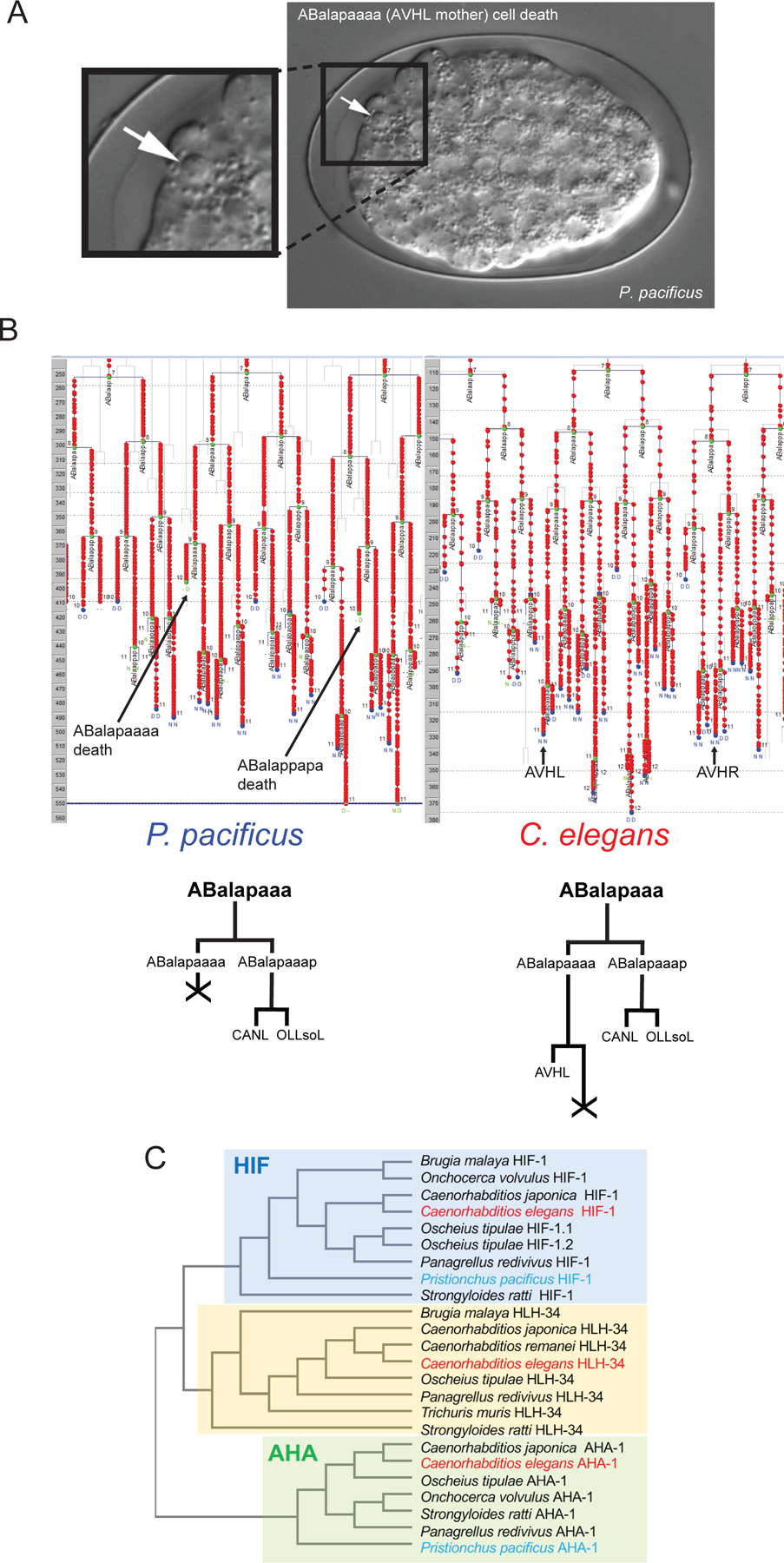
Heterochronic cell death in *P. pacificus.* **A:** Differential interference contrast image of the *P. pacificus* embryo, with an arrow pointing to the dying progenitor cell.. **B:** Lineaging diagram (generated by Simi BioCell) illustrating the death of AVH’s sister cell in *C. elegans* (left) and AVH’s progenitor cell death in *P. pacificus* (right) during embryogenesis. Cell fates are defined by nuclear morphology (N= neuron-like nuclear morphology; D = cell death; U = muscle; M = mitosis; P = pharynx; H = hypodermal; duplicated letter means: first letter = predicted fate; second letter = observed fate). **C:** Phylogenetic tree of the PAS domain of bHLH-PAS proteins from various nematode species.

We found one *C. elegans* interneuron class, the bilaterally symmetric AVH neuron pair, to be missing from the *P. pacificus* head region. This neuron pair is stereotypically located in the lateral ganglion in *C. elegans,* from where it extends a neurite through the nerve ring into and along the ventral nerve cord (10). The sister cells of both left and right AVH neurons undergo programmed cell death in *C. elegans* (11). We traced embryonic cell division patterns of *P. pacificus* using 4D video microscopy (12) and found that the AVH sister cell death in *C. elegans* occurs one cell division earlier in *P. pacificus*, hence explaining the absence of AVHL/R (Figure 2A, 2B, Table S1). Our embryonic lineaging analysis revealed that precocious cell death also explains the previously reported absence of the pharyngeal gland cell g2 in *P. pacificus* (13). Mirroring the AVH case, the sister of g2 dies in *C. elegans* (Figure S1B, S1D), whereas in *P. pacificus,* its progenitor dies (Figure S1A, S1C). Hence, together with additional cell deaths of VC motor neurons in *P. pacificus* (14), alterations in the spatial and temporal specificity of programmed cell death appear to constitute a major mechanism for shaping species-specific nervous system composition. We note that all other 13 embryonic cell deaths in the AB lineage, from which many neurons are born, and which normally occur after the 9^th^ round of cell division in *C. elegans,* are preserved in *P. pacificus* (Figure S2, Table S1). The cell cleavage patterns within a sampling of additional 33 neuroblast and neuronal lineages that cover multiple ganglia were also preserved, arguing for a broad conservation of embryonic patterning programs (Figure S2, Table S1).

We traced AVH’s lineage, as well as the g2 gland lineage, in two more distantly related nematodes that can serve as outgroups to *P. pacificus* and *C. elegans*: *Acrobeloides nanus* and *Plectus sambesii.* In both species, the cleavage patterns of the AVH- and g2 gland-generating lineage resemble the *C. elegans* pattern. Moreover, we found that the sister, rather than the progenitor of the presumptive AVH neuron and g2 gland cell, undergoes programmed cell death (Figure S3A, S3B, S3C). These observations indicate that the loss of *P. pacificus* AVH and g2, due to the heterochronic death of the progenitor cell, is a derived rather than ancestral feature of embryogenesis in *P. pacificus*.

We observed a molecular correlation to the loss of AVH neurons in *P. pacificus.* In *C. elegans*, the AVH neurons require a divergent member of the bHLH-PAS family of transcription factors, *hlh-34*, for their proper differentiation (15, 16). Sequence surveys of multiple highly divergent nematode species with complete and well-annotated genomes show that the *hlh-34* locus is lost in the *Pristionchus* genus (Figure 2C). Hence, the loss of a neuron due to heterochronic cell death is correlated with the genomic loss of its identity regulator.

We asked whether the loss of AVH in *P. pacificus* is accompanied by synaptic wiring changes in a neighboring neuron, AVJ, that shares many anatomical similarities to AVH in *C. elegans,* with both neurons displaying a similar neurite extension pattern through the nerve ring and along the ventral cord (10). The two neurons are electrically coupled and share some synaptic partners (10, 17, 18). However, we do not observe that the synaptic contacts that normally specifically involve AVH are absorbed by *P. pacificus* AVJ. Instead, AVJ generates additional *P. pacificus-*specific synapses (Supplementary Data S6).

## Differences in neuronal location

Another type of evolutionary change concerns cell soma position. Generally, we found that the relative positions of homologous neuronal somas are highly conserved between *P. pacificus* and *C. elegans*. Exceptions are the inner labial, dorsal and ventral IL2 (IL2D and IL2V) sensory neuron pairs. In *C. elegans,* the IL2D and IL2V neuronal soma are located at a similar position along the longitudinal axis in the anterior ganglion as the lateral IL2 neuron pair (10). In *P. pacificus,* however, the IL2D and IL2V soma pairs are positioned much more anteriorly (Figure 1B). Their axons therefore need to take a much longer path to reach the nerve ring, a feat that they may achieve by their close fasciculation with inner labial IL1 neurons, whose position is unchanged compared to *C. elegans* (Figure S4). The more anterior localization of the IL2D/V soma results in substantially shorter dendritic length. Since the *C. elegans* IL2D/V dendritic generate branches during the dauer stage to respond to mechanosensory touch involved in nictation behavior (19, 20), such shortening may result in a reduced sensory perceptive field of the branched IL2D/V dendrites in nictating *P. pacificus* dauer stage animals.

## Differences in neurite projection patterns alter network architecture

**Fig. 3:**
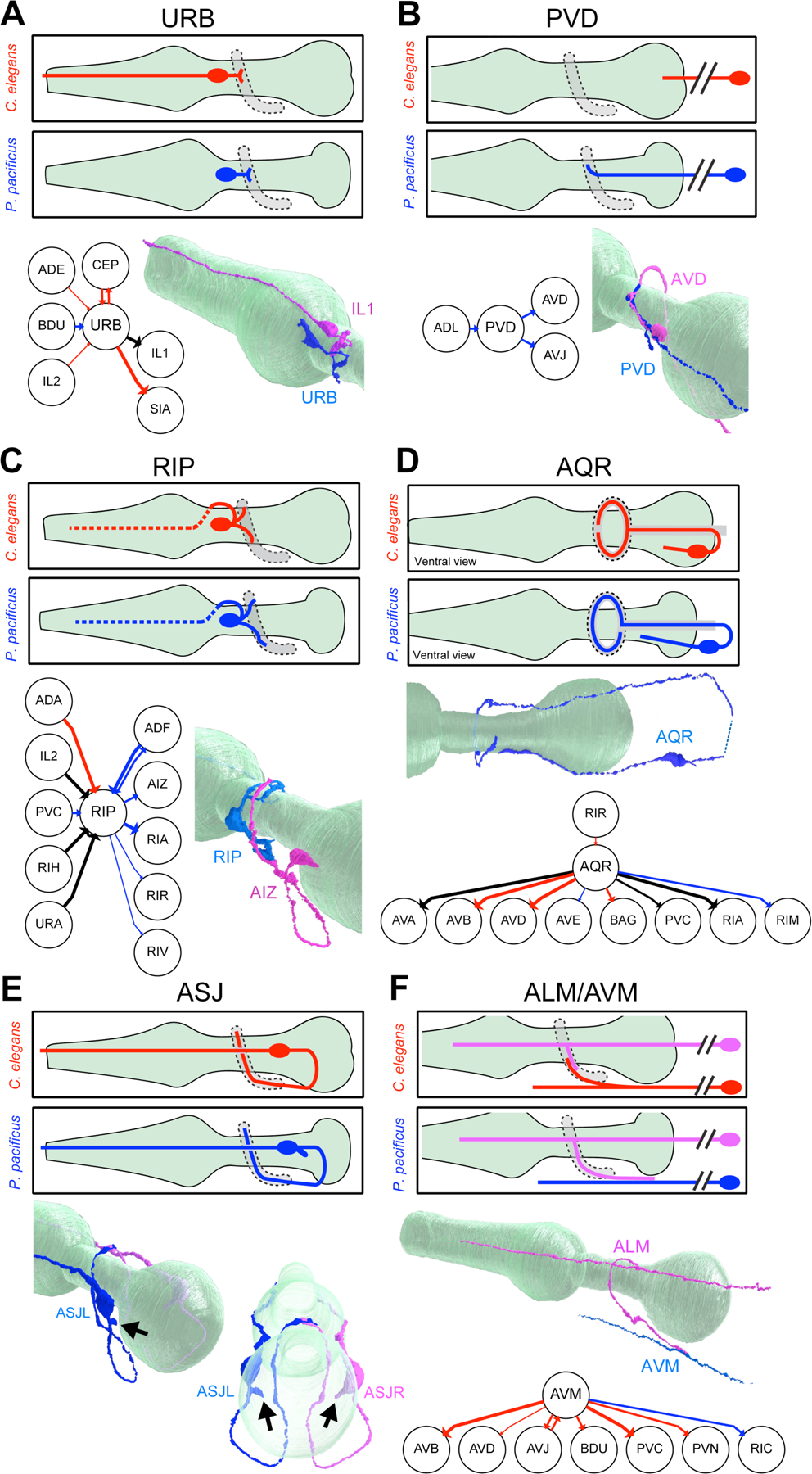
Morphological evolution of neurite trajectories. In each panel (**A-E**), the top section shows a schematic comparison of the neuron’s morphology in *C. elegans* (red) and *P. pacificus* (blue). Schematics are left lateral views (anterior to the left and dorsal up) for all panels except **C** (AQR), which is a ventral view. The pharynx is shown in light green and the nerve ring (neuropil region) in grey, outlined by a dashed line. Below the schematics are a connectivity diagram and 3D rendering of the *P. pacificus* neuron (blue), in some cases with a partner neuron (magenta). Connectivity diagrams show chemical synapses that are shared in all adult nematode samples (black), are *C. elegans*-specific (red), or *P. pacificus*-specific (blue). Line width is proportional to synaptic weight and arrowheads show directionality. A connectivity diagram is not relevant for panel E and is not included. **A:** URB morphology. The 3D rendering of *P. pacificus* URB (blue) is shown with a conserved synaptic partner of URB, the inner labial sensory neuron IL1 (magenta), which retains its dendrite in *P. pacificus*, extending from the nerve ring region to beyond the anterior tip of the pharynx. **B:** PVD morphology. The double slash indicates the PVD soma is posterior to the reconstruction. The 3D rendering shows PVD (blue) with *P. pacificus*-specific synaptic partner AVD (magenta), a closeup of the nerve ring region. **C:** RIP morphology. The 3D rendering shows RIP (blue) r with *P. pacificus*-specific synaptic partner AIZ (magenta), a closeup of the nerve ring region. **D:** AQR morphology *(ventral view)*. The 3D rendering shows AQR (blue) entering the nerve ring on the left (top), extending over dorsally to the right side (bottom). The dashed line indicates that the posterior-most AQR connecting neurite is not included in the reconstruction. **E.** ASJ morphology. 3D renderings of both ASJL (blue) and ASJR (magenta) are shown in the lateral posterior view (left) and dorsal posterior view (right, with nearly transparent pharynx), showing that these branches, indicated by arrows, project near the pseudocoelom. **F.** ALM and AVM morphologies. The double slash indicates the AVM and ALM somas are posterior to the reconstruction. The 3D rendering shows the simpler *P. pacificus* AVM neurite (blue) contacted by an extended ALM neurite (magenta).

Most individual neurons display highly characteristic neurite projection or branching patterns in *C. elegans,* which we leveraged to describe similarities and differences across species. For example, the *C. elegans* AIB neurites display an abrupt neighborhood shift in the nerve ring (10) which is conserved in *P. pacificus* (Supplementary Data S5)(9). Similarly, the unusual, truncated dendrite of the amphid-associated *C. elegans* AUA neuron class (10) is also conserved in *P. pacificus* (Supplementary Data S5)(9). The ring interneurons RIM, RIH, RIS and RIR display characteristic branching patterns in the *C. elegans* nerve ring and these patterns are also similar in both species (Supplementary Data S5). Paralleling these patterns of conservation, we discovered a rich set of evolutionary diversifications of projection patterns; several of them resulting in striking differences in presumptive information flow in the two nervous systems. The most dramatic differences is a complete repurposing of adult URB neuron function. In *C. elegans*, all available EM constructions and reporter genes show that URB extends a long dendrite toward the tip of the nose along the lateral amphid sensory dendrite bundle and extends an axon into the nerve ring to synapse with distinct sensory, inter- and motor neuron classes (10, 17, 18). In contrast, no dendritic extension of URB neurons is observed in either of the two *P. pacificus* samples (Figure 3A). Moreover, unlike in *C. elegans,* the *P. pacificus* URB axon innervates the head muscles in adults (Figure S5A). The concordant loss of dendrite and rewiring of axonal output suggests a functional switch from sensory to motor neuron between these two species.

Altered neurite extension patterns within the lateral nerve also indicate a divergence of routes of synaptic communication in the two nematode species. In *C. elegans,* the PVD sensory neuron extends a primary dendrite that typically terminates before reaching the nerve ring (10, 17). In contrast, in both *P. pacificus* samples, this dendrite projects more anteriorly into the nerve ring where it synapses onto AVD and AVJ and receives inputs from the nociceptive ADL neuron (Figure 3B, Figure S5B). Hence, the PVD neurite, normally purely dendritic in *C. elegans,* is repurposed to become axodendritic in *P. pacificus*, thus allowing the lateral nerve to provide ascending input to the brain.

Conversely, the harsh touch mechanoreceptive neuron, FLP, has a shorter axon in *P. pacificus*. In *C. elegans,* the two FLP neurons extend their axons through the deirid commissure, then project into the nerve ring where they generate synaptic outputs to the pre-motor command interneuron AVA (10, 17, 18). In *P. pacificus,* the FLP axons terminate before the nerve ring resulting in a loss of connections to the backward movement-inducing AVA. Meanwhile, new connections to the forward movement-inducing PVC command interneuron are seen (Supplementary Data S6). Although the precise function of FLP in *P. pacificus* is unknown, exchanging its synaptic output from AVA to PVC suggests a repurposing from avoidance to attractive responses to head touch. This change may relate to the predatory behavior of *P. pacificus*, in which contact with prey may not elicit an escape response.

A substantial alteration in information flow is apparent in the RIP neuron pair, the only neuron that synaptically connects the somatic and pharyngeal nervous systems (8, 10, 17, 18). At all developmental stages, the posteriorly directed neurite of RIP is strictly dendritic in the nerve ring of *C. elegans*, receiving synaptic input from a variety of central neuron classes (18). In *P. pacificus,* the posteriorly directed RIP neurite extends more deeply into the nerve ring and generates synaptic outputs onto different neuron types (Figure 3C). Hence, synaptic information flow through RIP appears unidirectional in *C. elegans* (from the nerve ring to the enteric nervous system), whereas in *P. pacificus*, RIP undergoes a partial polarity change to directly synapse onto both the somatic and enteric nervous systems. It remains to be investigated whether this rewiring relates to the predatory feeding behavior displayed by *P. pacificus.*

The unpaired AQR neuron, an oxygen-sensing neuron in *C. elegans*, represents another example of projection changes in *P. pacificus*. In *C. elegans,* the AQR neurite bifurcates upon entry into the nerve ring to extend symmetric neurites along either side of the nerve ring which then terminate shortly before reaching the dorsal midline (Figure 3D)(10, 18). In *P. pacificus*, the neurite of AQR does not bifurcate; instead, a single neurite reaches across the dorsal midline to the other side of the nerve ring (Figure 3D). The *P. pacificus* AQR neuron makes three small branches not observed in *C. elegans* as well as a large swelling at the dorsal midline, resulting in a preservation of distinctive gap junctions to the PVP neuron, as observed in *C. elegans* (10)(Supplementary Data S5). However, the *P. pacificus* AQR neuron does not innervate pre-motor command interneurons AVB and AVD, suggesting that AQR activation may trigger distinct behavioral read-outs. Indeed, oxygen-induced behavioral responses are divergent between *P. pacificus* and *C. elegans* (21).

The ASJ amphid sensory neurons display another type of projection change. ASJ neurons in both *C. elegans* and *P. pacificus* display a bipolar morphology with a sensory dendrite to the tip of the nose and an axon into the nerve ring. In *P. pacificus*, both ASJ neurons extend an additional short, thick neurite at or near the soma, not directed toward a specific fascicle but extending along amphid cell bodies (Figure 3E). In both samples and on both sides of the animal, this short neurite is filled with dense core vesicles (Figure S5C), indicating that in *P. pacificus* this neuron has intensified its capacity to broadcast neuropeptidergic signals across the nervous system.

Differences in projection patterns do not necessarily alter the information flow. The two anterior light touch receptor neuron classes ALM and AVM are electrically coupled, and each innervates command interneurons to signal reversal behavior in *C. elegans* (10, 22). The ventrally located AVM projects a bifurcating axon into the *C. elegans* nerve ring, where synapses are made to command interneurons, and terminates after meeting branches that are sent into the nerve ring by lateral ALM touch sensory neurons (Figure 3F)(10, 18). In contrast, the AVM neuron does not send a bifurcating branch into the *P. pacificus* nerve ring (Figure 3F), resulting in the loss of many AVM synapses observed in the adult *C. elegans*. The *P. pacificus* ALM neurites instead project further ventrally in *P. pacificus* to reach the unbranched AVM neurite on the ventral side (Figure 3F), each generating a large gap junction connection to AVM as they do in *C. elegans* (Figure S5D). In aggregate, synaptic outputs of the ALM and AVM neuron classes to command interneurons are therefore preserved in both nematodes, which is manifested by similar gentle touch response behaviors in both species (23).

## Synaptic connectivity repatterning without obvious changes in neurite projection.

**Fig. 4:**
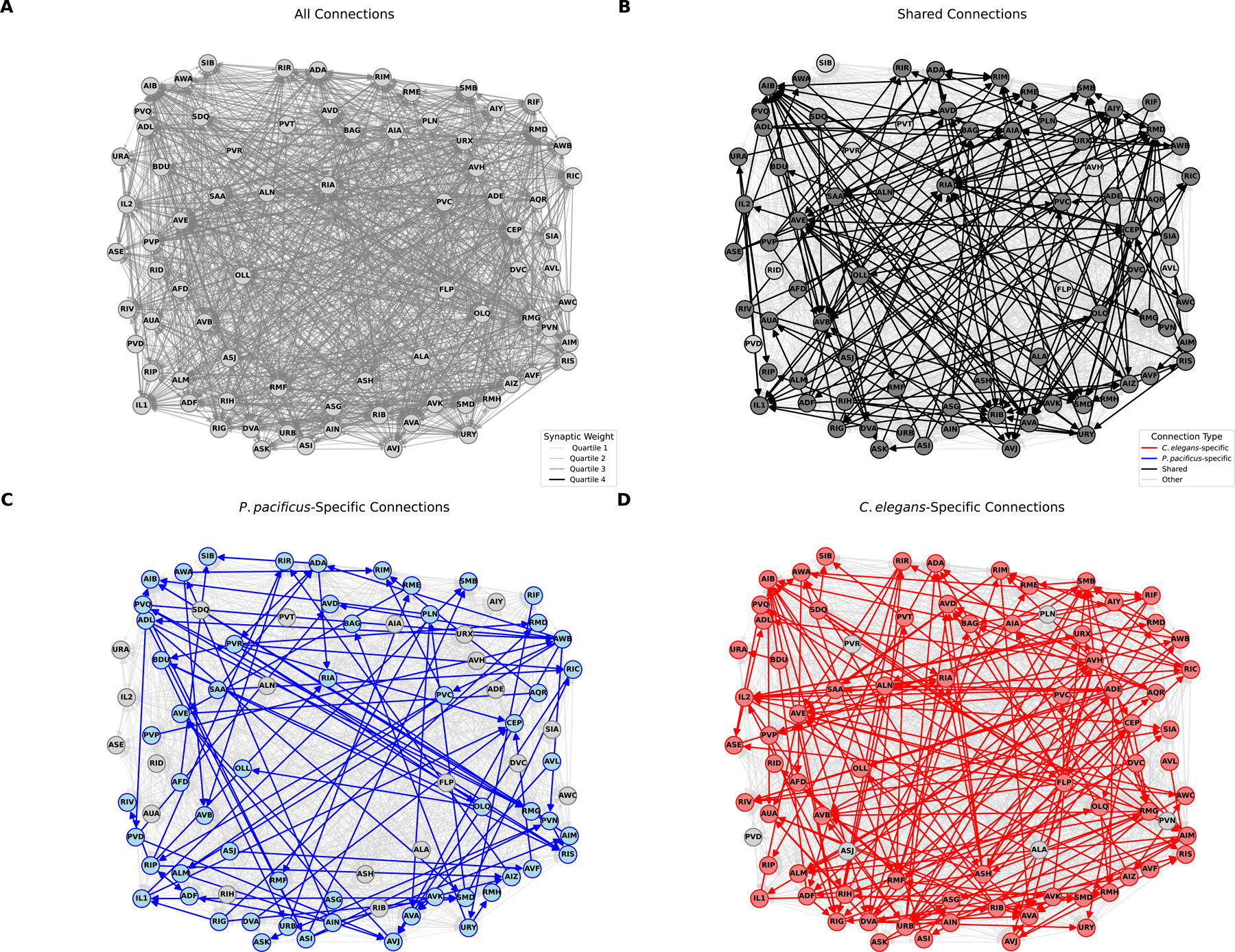
Brain-wide comparison reveals widespread connectivity changes. Panels A-D: Network (wiring) diagrams showing all neurons in the nerve ring and their synaptic connections: shared (black), variable (gray), *P. pacificus*-specific (blue), *C. elegans*-specific (red). Arrow widths are proportional to the average number of synapses across datasets; arrowheads represent directionality. **A.** Complete wiring diagram showing all *P. pacificus* and *C. elegans* connections. Legend for color code is provided. **B:** Shared wiring diagram highlighting shared, non-variable connections in black. Legend for synaptic edge weights is provided. **C.** Diagram highlighting *P. pacificus*-specific connections in blue. **D**. Diagram highlighting *C. elegans*-specific connections in red.

We discovered extensive evolutionary differences in synaptic connectivity patterns in the nerve ring independent of any obvious changes in overall neuron morphology. Analysis of multiple *C. elegans* connectomes across development has indicated a large degree of variability in synaptic connectivity patterns (18). To account for such variability in the context of interspecies comparisons, we first extracted all synaptic connections that are found in all 10 available *C. elegans* samples covering multiple developmental stages (L1, L2, L3, L4, Adult) and our two adult *P. pacificus* samples. These connections define a “shared connectome” that is conserved across development and evolution (see Discussion). In addition, we identified connections that are conserved in both *P. pacificus* samples but absent from all 10 *C. elegans* samples (*P. pacificus*-specific connectome) and connections that are absent in both *P. pacificus* samples but present in all 10 *C. elegans* samples (*C. elegans-*specific connectome). These sets of synaptic classifications are illustrated in Figure 4A–D. Schematic connectivity diagrams of each neuron class are assembled in Supplementary Data S6.

**Fig. 5:**
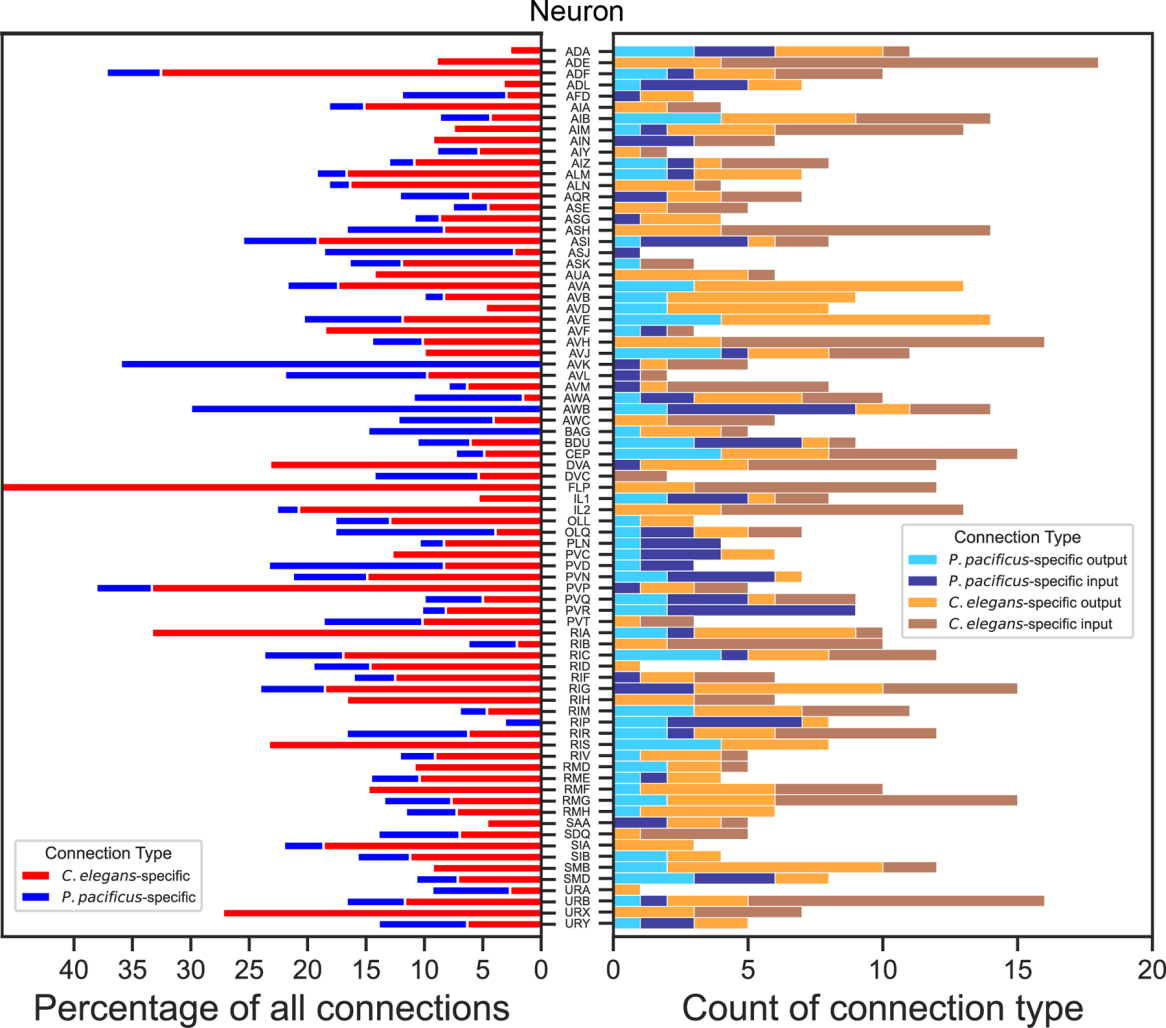
Analysis of species-specific adult chemical connectivity All embryonically born sex-shared nerve ring neurons that make a species-specific synaptic connection are shown. Stacked bar graphs of the percentage of species-specific undirected synaptic connections relative to all other synapses made by the respective neuron class (left). Stacked bar graphs of the number of sex-specific directed inputs and outputs by neuron class (right).

We found that both shared and species-specific connections are widely distributed throughout the nervous system, with no bias toward any neuron type. 88% of all neuron classes contribute to the shared connectome, observed in every available EM sample from both species, independent of developmental stage (Figure 4). Neurons that do not form any “shared nematode connections” (AVH, AVL, CAN, FLP, PVR, PVT, RID, and SIB) fall into multiple functional categories and are also not biased to a specific part of the nervous system. Species-specific synaptic connections are also pervasively distributed throughout the entire connectome (Figure 4). Around 96% of the analyzed neurons of the adult *P. pacificus* and *C. elegans* brain make species-specific connections, 72% of neurons generate *P. pacificus*-specific connections, whereas 87% of neurons generate synaptic partnerships only in *C. elegans* (Figure 4; Figure 5). Differences in the percentage of species-specific synapses are likely a reflection of a smaller sample size in *P. pacificus* compared to *C. elegans*. Species-specific synapses account, on average, for only 11% and 5% of each neuron’s synaptic connections in *C. elegans* and *P. pacificus,* respectively (Figure 5). Only three neuron classes, ALA, CAN and SAB do not form species-specific synapses (Figure 4C, D, Supplementary Data S6).

## Synaptic wiring changes as a function of neighborhood changes

**Fig. 6:**
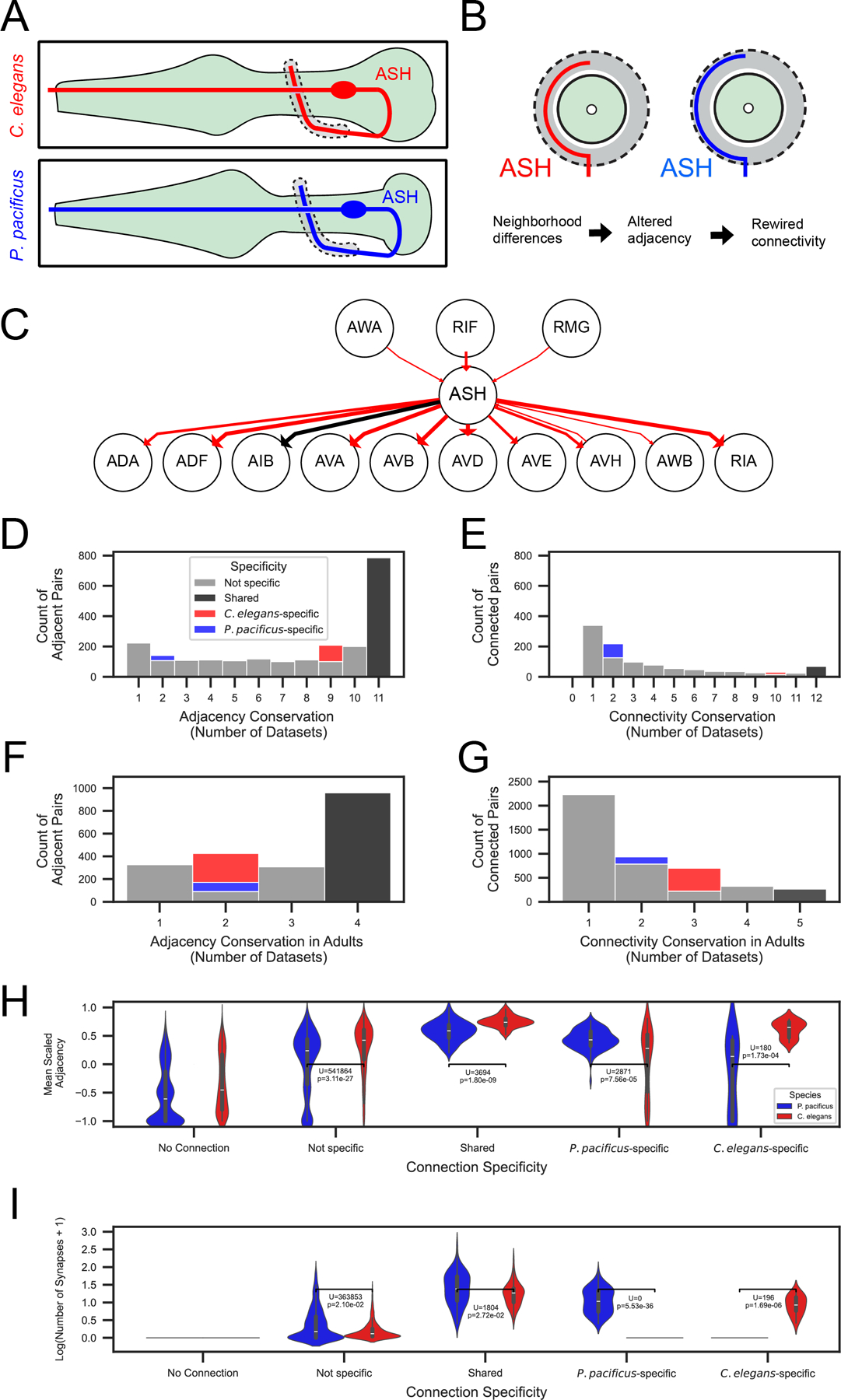
Neurite neighborhood shifts and quantitative analysis of synaptic connectivity differences. **A:** Schematic comparison of ASH neuron morphology in *C. elegans (red)* and *P. pacificus (blue)*. Left lateral view (see legend for Figure 3). **B:** Schematic of nerve ring cross section illustrating lateral displacement of ASH neurite in *P. pacificus* (blue) compared to *C. elegans* (red). **C:** Adult wiring diagram of ASH. *C. elegans*-specific connections are shown in red. **D:** Histogram of adjacency conservation of neuron pairs (Number of EM series with edge present) among all nematode nerve ring series (*C. elegans-*specific edges are red, *P. pacificus*-specific edges are blue, shared edges are black, and variable edges are gray). Legend applies to panels D-G. **E:** Histogram of directed connectivity conservation of neuron pairs (Number of EM series with edge present) among all nematode nerve ring series. **F:** Histogram of adjacency conservation of neuron pairs among adult nerve ring series. **G:** Histogram of directed connectivity conservation of neuron pairs among adult nerve ring series. **H.** Violin plot comparing mean scaled adjacency and connectivity specificity classification Condition: No Connection (no statistical test performed), Not specific, U statistic: 541864, Corrected p-value: 3.11e-27, Condition: Shared, U statistic: 3694, Corrected p-value: 1.80e-9 Condition: *P.* pacificus-specific, U statistic: 2871, Corrected p-value: 7.56e-5 Condition: *C. elegans*-specific, U statistic: 180.0, Corrected p-value: 1.73e-4. Legend applies to panels H and I. **I.** Violin plot comparing log+1 adjusted number of synapses and connectivity specificity classification. Condition: not specific, U statistic: 1842058, Corrected p-value: 9.87e-67 Condition: shared, U statistic: 1804.0, Corrected p-value: 2.72e-2 Condition: *P. pacificus*-specific, U statistic: 0.0, Corrected p-value: 2.33e-36 Condition: *C. elegans*-specific, U statistic: 12100.0, Corrected p-value: 1.02e-42. For underlying data, see Supplementary Data 7 and 8.

Species-specific synapses of morphologically similar neurons can be generated by dramatic shifts in the neuronal neighborhood. The best example is the polymodal, nociceptive ASH neuron. In *C. elegans,* the axon of ASH extends medially through the nerve ring, making ample *en passant* contacts with several interneurons, including command interneurons, until it meets its contralateral homolog at the dorsal midline (Figure 6A, Supplementary Data S5). The synaptic output of ASH transforms ASH-sensed nociceptive stimuli into a reversal response via pre-motor command interneurons (24). Similar to what we observed in *C. elegans,* each ASH axon in *P. pacificus* also extends through the nerve ring to meet its contralateral homolog at the midline, but the path taken through the nerve ring is substantially different, as it mostly travels along the outer side of the nerve ring (Figure 6B). This difference in the neuronal neighborhood has substantial consequences on the *en passant* synaptic connectivity: ASH loses its direct output to the homologs of the backward locomotion-inducing command interneurons in *P. pacificus* (Figure 6C). These differences in synaptic connectivity might be linked to the experimental finding that in *P. pacificus,* the ASH neurons also mediate reversal to nociceptive stimuli as in *C. elegans* (3). Hence, in these two nematodes, nociceptive sensory information is perceived by the same neuron but might be relayed through different synaptic pathways to produce similar motor outputs.

How widespread are small species-specific neighborhood rearrangements and do they instruct species-specific connections? Several above-mentioned extreme examples of changes in morphology (including ASH, PVD, and RIP) produce species-specific adjacencies associated with species-specific connectivity. In total, there are 28 instances of species-specific synaptic connections due to adjacencies that are exclusive to one specific species (Figure S6A, S6B). We found that more frequently, species-specific connections found in the adult occur in the context of shared adjacencies across all adults (Figure S6A, S6B).

To explore this matter further, we undertook a more quantitative, whole-brain analysis of the relationship between neurite adjacency and synaptic connectivity. We assigned a “conservation score” to each neuron pair based on how consistently they form adjacency and synaptic connections across our dataset. This score counts the number of samples where an edge exists between the neurons. For example, a connection present in 5 different EM series would receive a conservation score of 5. We found that a greater proportion of adjacency edges are found in all *C. elegans* and *P. pacificus* samples than synaptic connectivity edges (Figure 6D, 6E). We next limited our analysis to only adults, again finding that neuron pairs are frequently adjacent in all samples (Figure 6F) but rarely are connected in all samples (Figure 6G).

Is the presence of a shared set of adjacencies and connections merely due to chance? To address this possibility, we compared the distribution of adjacency and connectivity conservations across datasets to randomized null distributions. We found that our experimentally observed distributions of shared connections across datasets were more frequent than chance (Figure S7A), and that the count of species-specific connections was statistically different from chance (Figure S7C). This differences also holds for adjacencies, where we observed a greater number of shared adjacencies when compared to chance (Figure S7B), with a statistically greater number of species-specific adjacencies (Figure S7D). Together, these results show that the patterns of shared and species-specific adjacency and connectivity are much greater than chance, amplifying the importance of comparative analyses.

A previous analysis of neurite adjacency and synaptic connectivity revealed that the relative amount of neurite membrane adjacency predicts synaptic connections between neuron pairs in *C. elegans* (25). We scaled and averaged the adjacency data across EM samples and found that neuron pairs with species-specific connections are on average more strongly adjacent in that species than in the other species (Figure 6H). For connections shared across species, a smaller inter-species difference in mean scaled adjacency is observed (Figure 6H). This trend extends to synaptic connectivity, where we observed that the largest connections, on average, are shared between the two nematodes. Variable connections are on average smaller than those found in one species (Figure 6I). Taken together, our analysis indicates that the shared and species-specific connections are strongly related to the relative strength of neuronal adjacency.

Synaptic connectivity can be accurately modeled using relative adjacency in *C. elegans* (25). To explore whether the relationships between connectivity and adjacency are conserved, we extended this modeling approach to *P. pacificus.* We adjusted the training (*C. elegans* contact and connectomes) and test data (*P. pacificus* contact and connectomes), using two predictors (mean scaled adjacency and brain strata)(26) to classify synaptic connectivity (present in at least one sample). We found a moderately stronger relationship between mean scaled adjacency and mean connection weight among neuron pairs within vs. across different brain strata (Figure S8A, S8B) (25). Our modeling approach yielded superior results across classification algorithms (Figure S8C) compared to our previous results modeling connectivity in *C. elegans* alone. For comparative purposes, we plotted the Received Operator and Precision Recall curves for the Logistic Regression classifier, revealing an accurate connectivity prediction from simple neuronal adjacency characteristics (Figure S8D, S8E). The similarity of our modeling results across species supports our previous proposal that relative adjacency is a guiding principle of synaptic organization in nematodes (25).

## Network configurations of the *C. elegans* and *P. pacificus* connectomes

Next, we undertook a comparative analysis of network topology. To assess network consistency, we compared the degree (number of edges attached to a node) distributions of the *C. elegans* and *P. pacificus* chemical synaptic and adjacency networks. The degree distributions of the adjacency networks are strongly similar across datasets (Figure S9A) with no statistically significant pairwise differences present (Figure S9C). Conversely, the synaptic degree distributions displayed widespread differences across development and species (Figure S9B, S9C, S9D). These findings support the notion that whereas the overall contactome structure remains stable, the connectome exhibits greater variability, being more influenced by the nuanced quantities of neuronal adjacency rather than by a binary presence-or-absence effect.

To better describe differences in network architecture across species we calculated several common graph theoretical metrics to make comparisons across all datasets (Figure S10A). There are many key organizational principles conserved across all datasets, including small-worldness, high clustering coefficients, and relatively short processing path lengths. We performed hierarchical clustering using these graph metrics, revealing that networks cluster primarily by developmental stage (Figure S10B). Whereas transitivity and average clustering coefficients remained relatively stable across datasets, indicating the conservation of local processing modules, reciprocity values showed more variation, particularly in *P. pacificus,* suggesting species-specific differences in bilateral connectivity patterns. The degree assortativity coefficients were consistently negative across all networks, indicating that both species maintain a hierarchical hub-based organization where high-degree nodes tend to connect with low-degree nodes. Together, these results demonstrate that despite substantial evolutionary distance and different ecological niches, *C. elegans* and *P. pacificus* maintain similar fundamental organizational principles in their nervous systems.

To examine individual functional units of the nervous system across species, we compared the motifs by which neurons connect to each other across datasets. We found strong differences between species where the motifs overrepresented in *C. elegans* were not overrepresented in *P. pacificus* (Figure S11). Single-edge, and transitive triangle motifs were enriched in *P. pacificus* but not in *C. elegans.* The enrichment of transitive triangles (A→B→C, A→C) particularly indicates a prevalence of feed-forward processing with consistent hierarchical relationships between neurons. We speculate this architectural pattern might reflect *P. pacificus’s* more specialized behavioral repertoire, where direct, reliable signal propagation is prioritized. These findings suggest that whereas basic organizational principles are conserved between species, the relative emphasis on different circuit motifs has diverged through evolution to support species-specific behavioral adaptations.

If specific types of circuit building blocks are different across species, could individual neurons be differentially important across species? To identify highly connected neurons that form the core of each connectome, we performed a conservative rich club analysis (Figure S12). Rich clubs were identified using stringent criteria requiring both strong statistical enrichment (≥3 standard deviations above random networks) and robust topological features (≥3 consecutive degree thresholds). Several neurons with well-defined functional roles were identified as rich club members in both species: AIB, AVE, CEP, RIA, and RMD. The neurons ASI, BDU, and RIP are rich club members in *P. pacificus* but not *C. elegans* whereas other have lost their rich club status in *P. pacificus* compared to *C. elegans* (Figure S12). Taken together, these findings provide evidence for both conserved and species-specific aspects of shared circuit organization in nematode nervous systems. Fundamental information processing differences have been maintained through evolution while allowing for species-specific adaptations in circuit architecture, just as we observed in neuronal morphology.

## Glia connectivity as a substrate of evolutionary change.

**Fig. 7:**
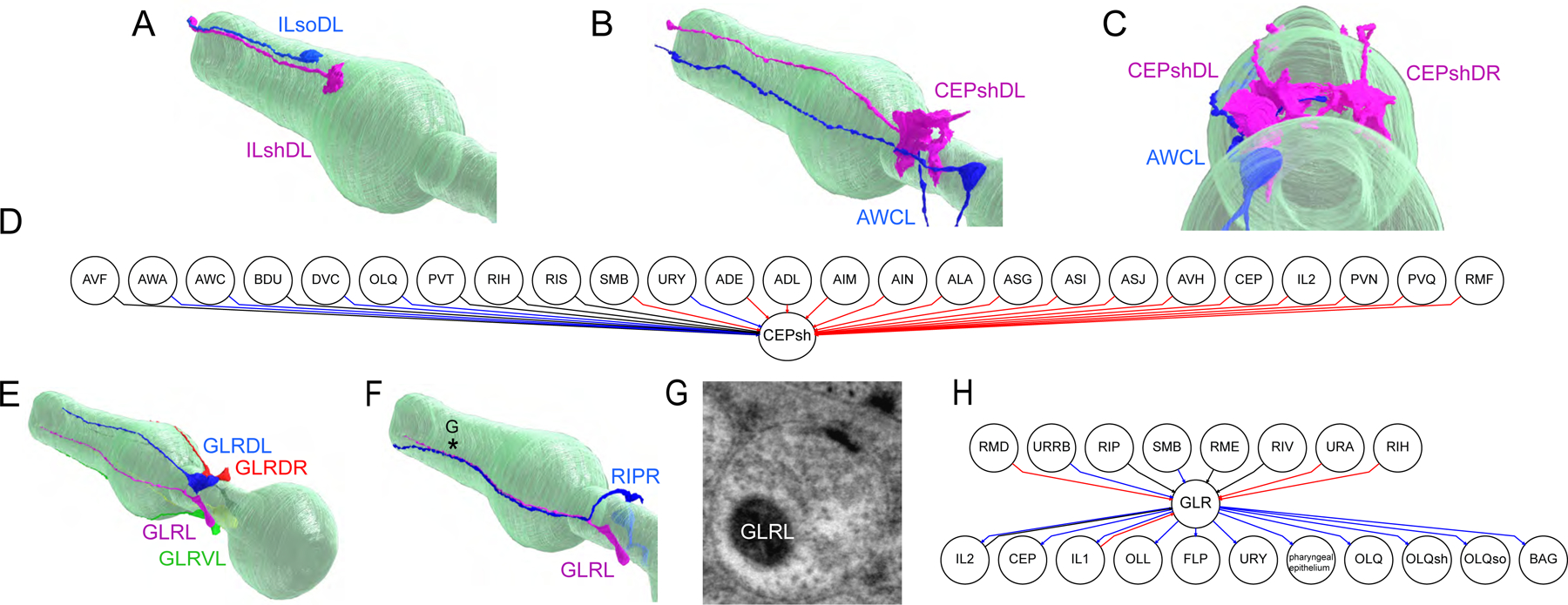
Glial rewiring across species. **A:** Lateral posterior view of 3D rendering of IL sheath (ILshDL, magenta) and socket (ILsoDL, blue) cells with the pharynx shown in green. **B:** Lateral posterior view of 3D rendering of the CEPshDL (magenta) and AWCL (blue) cells with the pharynx shown in green. **C:** Posterior view rendering of CEPshDL/R (magenta) and AWCL cells (blue) with the pharynx shown in green. **D:** Chemical synaptic connectivity of CEPsh in adults. Blue lines are connections found only in *P. pacificus*, red lines are connections found only in *C. elegans*, and black lines are connections shared in both species. **E:** Lateral posterior view rendering of 3D reconstruction of the GLR cells with the pharynx shown in green. **F:** Posterior lateral view of the GLRL (magenta) and RIPL (blue) cells, showing close fasciculation of processes along the pharynx (green). The asterisk denotes the location of the synapse shown in G. **G:** Example GLRL synapse onto the pharyngeal epithelium. The micrograph shows presynaptic specializations including a presynaptic density and clear vesicles. **H:** Chemical synaptic connectivity of GLR in adults. Blue lines are connections found only in *P. pacificus*, red lines are connections found only in *C. elegans*, and black lines are connections found in both species.

We also analyzed the other main component of the nervous system, glial cells. The ultrastructure of *P. pacificus* head glia supports a one-to-one correspondence of the three cardinal glia cell types (sheath, socket, and GLR) (Figure 7A, 7C, 7E). Despite the anatomical similarity, we discovered one distinctive feature of *P. pacificus* GLR glia. These microglia-like cells extend two types of processes in both species: thin, leaf-like posterior processes that wrap the interior of the muscle-neuron plates of the nerve ring as well as anterior processes that fasciculate with inner labial dendritic sensory fascicles. (Figure 7C, 7F)(9). In *P. pacificus,* these anterior extensions contain bona fide presynaptic features, including, swellings, active zones and synaptic vesicles (Figure 7G). The GLR cells receive synaptic input from RIP in both species but make *P. pacificus*-specific output onto inner- and outer labial cells, other glia and the pharyngeal epithelium (Figure 7H). So-called “gliotransmission” has been reported in vertebrate astrocytes (27) but, to our knowledge, it has not yet been revealed as a substrate of evolutionary change in a nervous system. As we already speculated in the context of the wiring differences of the RIP neurons, the ability of *P. pacificus* GLR glia to communicate with pharyngeal tissue may relate to predatory feeding behavior.

We discovered that the astrocyte-like CEPsh glia also displays differences in synaptic connectivity. The extensions of the CEPsh glia that line the nerve ring are innervated by the neurites of several distinct neuron types in the *C. elegans* nerve ring (18). Similar to the GLR glia, the *P. pacificus* CEPsh glia are localized to nearly identical anatomical locations with no gross shift in neighborhood placement. In *P. pacificus*, several of these inputs are lost and others are gained. For example, there is now strong innervation of the CEPsh glia by the AWC olfactory neurons in the nerve ring (Figure 7B–D). Vertebrate astrocytes also receive synaptic inputs that are translated into gliotransmitter release (27). Although we observe no obvious synaptic release sites in the CEPsh glia, we note that at least in *C. elegans*, the CEPsh glia release neuropeptides to control stress resistance and animal lifespan (28). If this CEPsh function is preserved in *P. pacificus*, a rewiring of CEPsh glia indicates that the neuronal control of such important physiological parameters may be subject to evolutionary change.

## DISCUSSION

The well-described neuronal anatomy of *C. elegans* combined with our analysis of multiple *P. pacificus* datasets yields a comprehensive map of how brains change over millions of years of evolution. Our analysis revealed that most neurons appear highly conserved in their overall position and morphology; however, we discovered multi-tiered substrates of evolutionary change, ranging from alteration in the number of individual constituent neurons (exemplified by species-specific neuronal cell death), cell body position, projection patterns of dendrites and/or axons, to changes in synaptic connectivity which result in distinct network structures.

Brain development in vertebrate and invertebrate animals, including nematodes, involves a substantial amount of cell death (29, 30). The discovery of alterations in the cellular and temporal specificity of neuronal cell death programs in *C. elegans* and *P. pacificus* indicates that programmed cell death may constitute an important driving force in shaping brain architecture and, hence, circuit architecture over evolutionary timescales. Such a mechanism may explain the observation that in several nematode species more or fewer inner or outer labial sensory neurons, have been observed (31–33); in *C. elegans* sisters of these labial sensory neurons undergo cell death (11) and, hence, a precocious or a lack of execution of the cell death program in these lineages compared to *C. elegans* could explain these differences in cellular composition of the labial sensory apparatus. Changes in neuronal composition through altered cell death patterns have been observed in related *Drosophila* species (34, 35), indicating that this mechanism of brain evolution is conserved across phylogeny.

We found that most neurons generate species-specific synaptic connectivity, arguing for widespread changes in information flow and processing. Changes in synaptic connectivity are largely, but not exclusively, the result of changes in relative process adjacency, which supports the applicability of Peters’ Rule for the generation of synaptic specificity in nematodes (25). Changes in network structure do not necessarily result in behavioral changes, as illustrated by the ASH sensory neurons which, based on their synaptic connectivity, appear to transmit sensory information to a distinct set of downstream interneurons. Yet, in both species, ASH triggers nociceptive responses to aversive cues (3, 24). Akin to the concept of developmental systems drift (36), the ASH circuit may constitute an example of “circuit drift”, in which the pathways of information processing may have drifted, while keeping behavioral output constant (37–39). We cannot be certain that the behavioral output is indeed identical (for example, there could be substantially distinct response dynamics to ASH-mediated nociceptive inputs), but we speculate that the routing of ASH sensory information through distinct sets of interneurons might allow nociceptive response to be subject to synaptic modulation by other components of the nervous system.

Differences in synaptic wiring indicate functional changes. For example, one neuron class, called URB in *C. elegans*, displays a distinct set of neurite projection patterns and synaptic connectivity patterns, entailing the loss of a putative sensory dendrite and the gain of neuromuscular connections, indicative of a potential functional change of this neuron class. Another example is the bilateral RIP interneuron pair, the sole connector of the somatic and pharyngeal nervous system of both nematode brains. In *P. pacificus,* the RIP neurons enable reverse signal flow from the pharyngeal nervous system to the central nervous system through its generation of synaptic outputs in the nerve ring. Moreover, the new wiring patterns transform RIP into becoming one of the few rich club neurons in the *P. pacificus*. The *P. pacificus*-specific synapses between RIP and glia also speak to the expanded signaling range of RIP. We speculate that RIP’s wiring changes relate to the predatory behavior of *P. pacificus* that involves biting, killing and prey ingestion, in which the pharyngeal nervous system, possibly in coordination with the somatic nervous system, likely plays an important role (8).

By analyzing whole brains rather than isolated components, we were able to address a fundamental question: Are evolutionary changes distributed uniformly across the brain or concentrated in specific hotspots? Our analysis reveals that both evolutionary conservation of synaptic connectivity and evolutionary innovation occur throughout the entire brain.

## LIMITATIONS

Our comparative connectomic analysis of only two nematode species does not presently allow us to infer which anatomical features are ancestral and which are derived. Our lineage analysis of two additional, more basal nematode species (*A. nanus* and *P. sambesii)*, suggest that the AVH neuron loss, as well as the g2 gland cell loss may be specific to diplogastrids like *P. pacificus,* a notion bolstered by the diplogastrid-specific genomic loss of AVH’s terminal selector gene. Reconstructing the nervous system of additional nematode species has the fascinating potential to reveal truly pan-nematode features – from cell types to synaptic connectivity – conserved over more than 400 million years of evolutionary time when nematodes and its most related phylum, the nematomorpha, shared their last common ancestor (1). Any deviations from these pan-nematode features likely represent behavioral adaptations that enabled nematodes to colonize the extremely diverse ecological niches they occupy today.

## MATERIAL AND METHODS

### EM analysis methodology

Two serial section EM series of eurystomatous (‘wide mouth,’ predatory) hermaphrodite animals (8) from the PS312 strain were further reconstructed for ultrastructural morphology and connectivity using TrakEM2 (40). In brief, each neuron profile present in an EM section was manually traced to create volumetric neuron reconstructions. From these series, ROIs were created which correspond to the nerve ring and surrounding neuropil. We limited our comparative analysis of neuronal and adjacency data to this ROI, but report the connectivity data in Supplementary Data S5, S6. Neural adjacency data for *P. pacificus* were computationally extracted as previously described (26), with slight modification to extract ‘areatree’ annotations using multiprocessing. We manually identified chemical synapses and gap junctions between neurons and tissues using criteria established previously (10, 17), but excluded gap junction connectivity from our comparative analysis due to ultrastructural ambiguity, a persistent issue in samples processed for connectomics. Connectivity data were extracted from TrakEM2 using a combination of built-in tools and custom python parsing scripts. These extracted connectivity data include the number of synapses and weight metric (number of consecutive serial sections where synaptic specializations are observed) for each EM series. Our analysis is limited to the number of connections between two neurons unless noted otherwise.

### Randomization and graph theoretic analysis

Synaptic connectivity and adjacency data were compiled across all datasets such that each row represents a neuron pair and its presence or properties are stored in columns. To generate randomized null distributions, we randomly swapped edges between neuron pairs preserving the overall degree distribution for 5x the number of edges in each network, repeating for 10,000 iterations. At each iteration, we calculated the distribution of edge counts and species-specific edges found. We then compared these distributions to the observed data, calculating a Z-score and two-tailed p-value for each.

### Assignment of homologous neurons

We limited our cross-species comparison of ultrastructure to normal *C. elegans* development and only the hermaphrodite sex. Given its rich description of inter-individual variability, we used anatomical landmarks of *C. elegans* as a reference. To identify neuronal identities, we classified cells based on the following features (in order of importance): relative soma location, symmetry (two-fold, four-fold, six-fold), soma size, number and direction of processes, hallmark structural features like AIY’s ‘humped’ morphology in the ventral ganglion (10, 17). Further ambiguities were resolved by evaluating the specific location of a process within the neuropil and distinct swellings in axons or dendrites. We avoided the use of synaptic connectivity to identify cells. In extreme cases, such as the AVH neuron, embryonic lineaging resolved any ambiguity, while in the case of URB, the *P. pacificus* URB neurons (which displays very distinct projection patterns) were identified based on soma position in the anterior ganglion, and a process of “exclusion”, in which all other anterior ganglion neurons were clearly identifiable as homologous, leaving only the URB neuron.

All neuronal cells with connected somas were identified within our region of interest (ROI). However, because the EM series did not cover the entire animal, we were unable to positively identify all processes within the ROI and, therefore, excluded those from our analysis. These cells include ‘unknown’ RVG neurons that project into the ventral ganglion, which do not have homologous counterparts in *C. elegan*s. Many RVG neurons are generated from postembryonically dividing P cell neuroblasts. In the ventral nerve cord, these P cell neuroblasts give rise to neurons, as well as neurons fated to die. Since those ventral neuron death patterns are known to change in *P. pacificus* (14), we suspect that the extra RVG neurons in *P. pacificus* are also the result in changes in cell death patterns.

Additionally, some axons project from the posterior of the animal into the nerve ring but lack distinguishing characteristics in *P. pacificus* which are seen in *C. elegans*, such as prominent neighborhood positions or distinct branching patterns. For example, there is a bilateral pair of *P. pacificus* axons that fasciculate in the nerve ring similar to HSN in *C. elegans*, but unlike *C. elegans* these *P. pacificus* neurons are unbranched and both travel in the right fascicle of the ventral nerve cord. Serotonin staining patterns also suggest that there is no obvious HSN-like neuron in the adult *P. pacificus* (41).

The pharyngeal and amphidial circuits were previously homologized with a 1:1 correspondence between each species in their neuronal identity (8, 9). Due to details of their axonal projection patterns, the analysis of reporter data and discussions with R. Hong, we exchange the identities of *P. pacificus* ASE and AWC in this paper relative to a previous analysis of the amphid circuitry of *P. pacificus* (9). To make accurate morphological comparisons, we evaluated the morphology of *P. pacificus* neurons in comparison to all available 3D reconstructions in *C. elegans* (10, 18, 26).

### Machine learning assumptions, selection, performance

Our methodology for assumptions, selection, and performance of classification algorithms was used as previously described (25). In brief, we classify each edge (pairs of neurons known to be adjacent) as a binary value for synaptic weight (>0 average connection size = 1, no connection observed across samples = 0). We then trained our model using *C. elegans* adjacency and connectivity data using the mean scaled and normalized adjacency of pairs and a categorical variable describing whether two neurons are in the same or different strata (26). Our models (MLPClassifier, DecisionTreeClassifier, RandomForestClassifier, and LogisticRegression from the scikit-learn library (42) were then evaluated on the test dataset of *P. pacificus* adjacency and connectivity data. For comparative purposes, we again chose the parameters previously used in our logistic regression model for evaluating *C. elegans* adjacency and connectivity data.

### Visualizing morphology and circuitry

Renderings of individual neurons or groups of neurons from Specimen 104 were performed using Adobe Dimension or Blender. Some post-rendering modifications of images were done with GraphicConverter. All code required to automate the processing of Blender data are available at https://github.com/stevenjcook/cook_et_al_2024_mipristionchus. Individual neuron renderings, Blender and Adobe Dimension files used for rendering will be made available on Zenodo upon publication.

Circuit diagrams for individual neuron classes were generated using GraphViz (https://graphviz.org/) using the ‘circo’ layout. Display layouts of complete networks were made using Cytoscape (43). The layout structure is an implementation of an affinity view popularized by (44).

### Quantification and statistical analysis

The statistical analysis performed in this paper is a combination of python software packages which include Scikit-learn and SciPy. All information for individual statistical tests including test and p-values can be found in the figure legends, while summary statistics are reported in the main test. All graph theoretic analysis code is available on Github with reproducible figures.

Mann Whitney U tests were used in Figure 6. We used this nonparametric test to avoid assumptions regarding underlying data distributions. To assess the significance of observed connectivity patterns in Supplemental Figure 7, we performed randomization tests by generating null distributions through degree-preserving edge swapping, where each network was randomized 1,000 times using parallel processing while maintaining the original in-degree and out-degree distributions of all neurons. For each connectivity pattern, we calculated z-scores by comparing observed values to the mean and standard deviation of the randomized distributions and computed two-tailed p-values using the standard normal distribution. To assess statistical differences between degree distributions across datasets in Supplemental Figure 9, we performed Dunn’s post-hoc test, a non-parametric multiple comparison procedure suitable for comparing distributions that may not meet normality assumptions. P-values from all pairwise comparisons were adjusted using the Bonferroni correction to control for multiple testing. Rich club coefficients In Supplemental Figure 9 were calculated for each network by measuring the density of connections among high-degree nodes (degree ≥ k) and normalized against 1,000 degree-preserving random networks generated using the directed configuration model to control for degree sequence effects. Statistical significance of rich club organization was determined using conservative criteria requiring normalized coefficients to exceed 3 standard deviations above random expectation for at least 3 consecutive k-values, with additional requirements for minimum rich club size (≥5 neurons) to avoid spurious results.

### 4D-microscopy and lineage analysis

The method for 4D-microscopy was described previously (12). Modifications of this system are described in (45). All recordings were acquired at 25 °C and analyzed using the Software Database SIMI©BioCell (SIMI Reality Motion Systems, Unterschleissheim, Germany; http://www.simi.com/)(12, 45). Cells are followed by the observer and the coordinates are recorded approximately every 2 min. The cell cleavages are assessed by marking the mother cell before the cleavage furrow ingresses and subsequently the centers of the daughter cells three frames later. By marking every cell during the complete embryonic development, the complete cell lineage of an embryo is generated.

### Resource availability

This study did not generate new or unique reagents. Additional requests for information related to data analysis should be directed to SJC and OH.

### Data Sources

*C. elegans* nerve ring connectivity and adjacency data were previously published (17, 18, 26). The *P. pacificus* electron micrographs used in this study were previously published (8) and previously analyzed for amphidial circuitry (9).

## Supplementary Material

Supplementary Material

Data S1

Data S2

Data S3

Data S4

Data S5

Data S6

Data S7

Data S8

## Data Availability

All neuronal adjacency and connectivity data necessary for the analyses presented in this paper are included in supplementary materials. All code necessary to analyze neuronal adjacency and connectivity data in this paper are provided are available at https://github.com/stevenjcook/cook_et_al_2025_pristionchus. Raw EM imagery is available at https://bossdb.org/project/bumbarger2013.
