# Supplementary material for: Comparative connectomics of two distantly related nematode species reveals patterns of nervous system evolution
Source: Science. Author manuscript; Available in PMC 2025 Aug 7. (PMC12330220; doi:10.1126/science.adx2143)

ADAL, ADAR

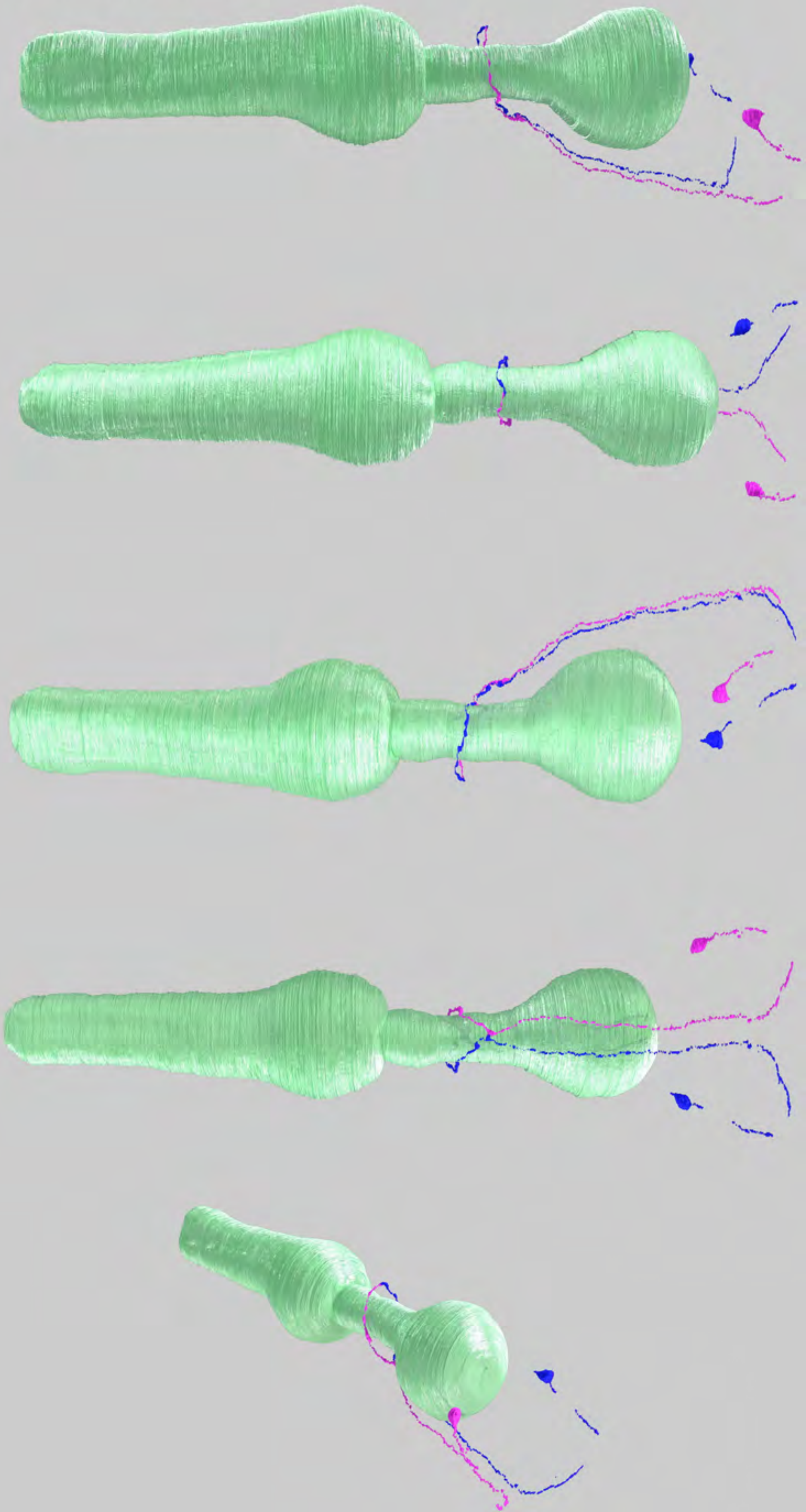

ADEL, ADER

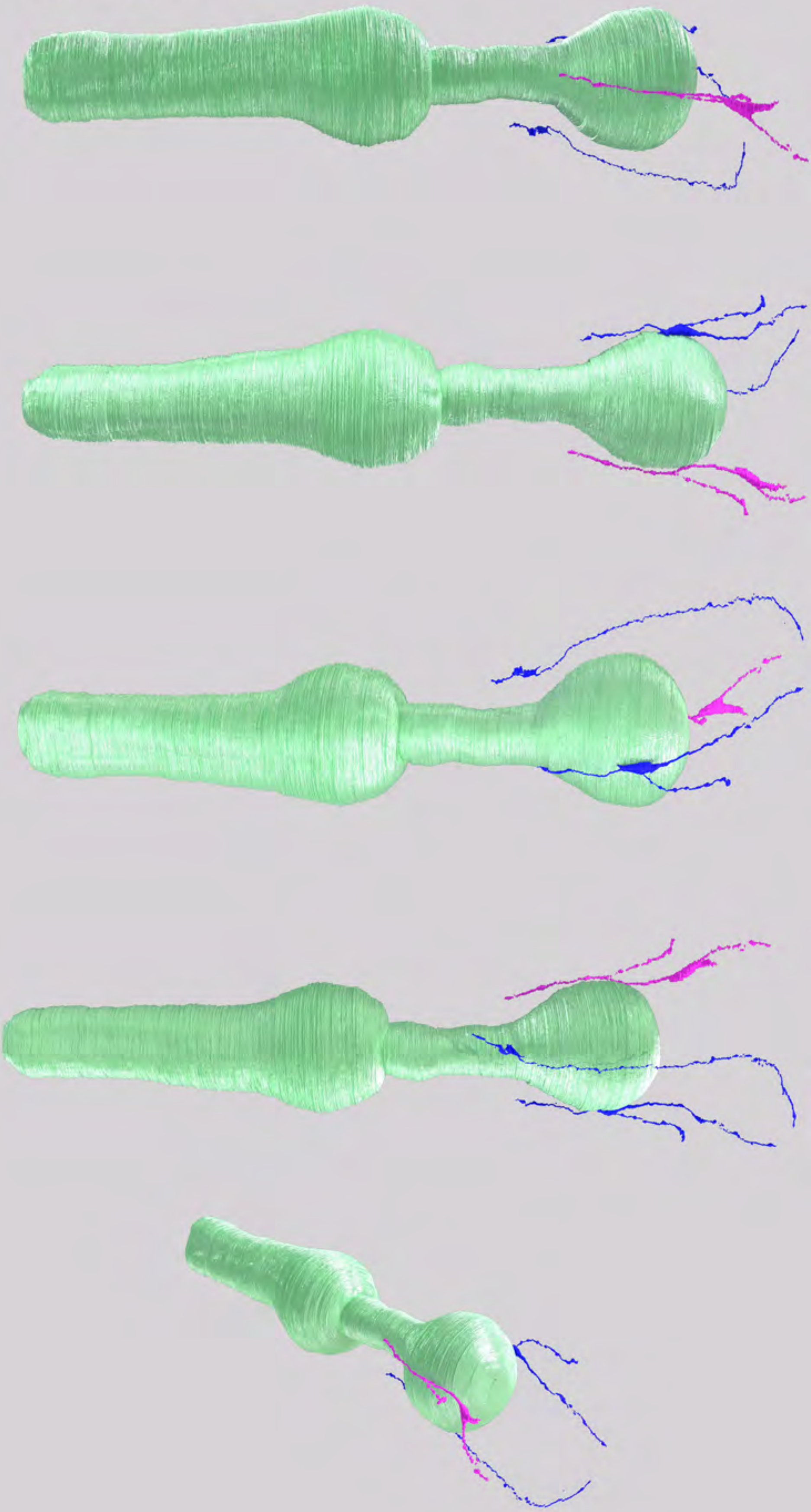

ADFL, ADFR

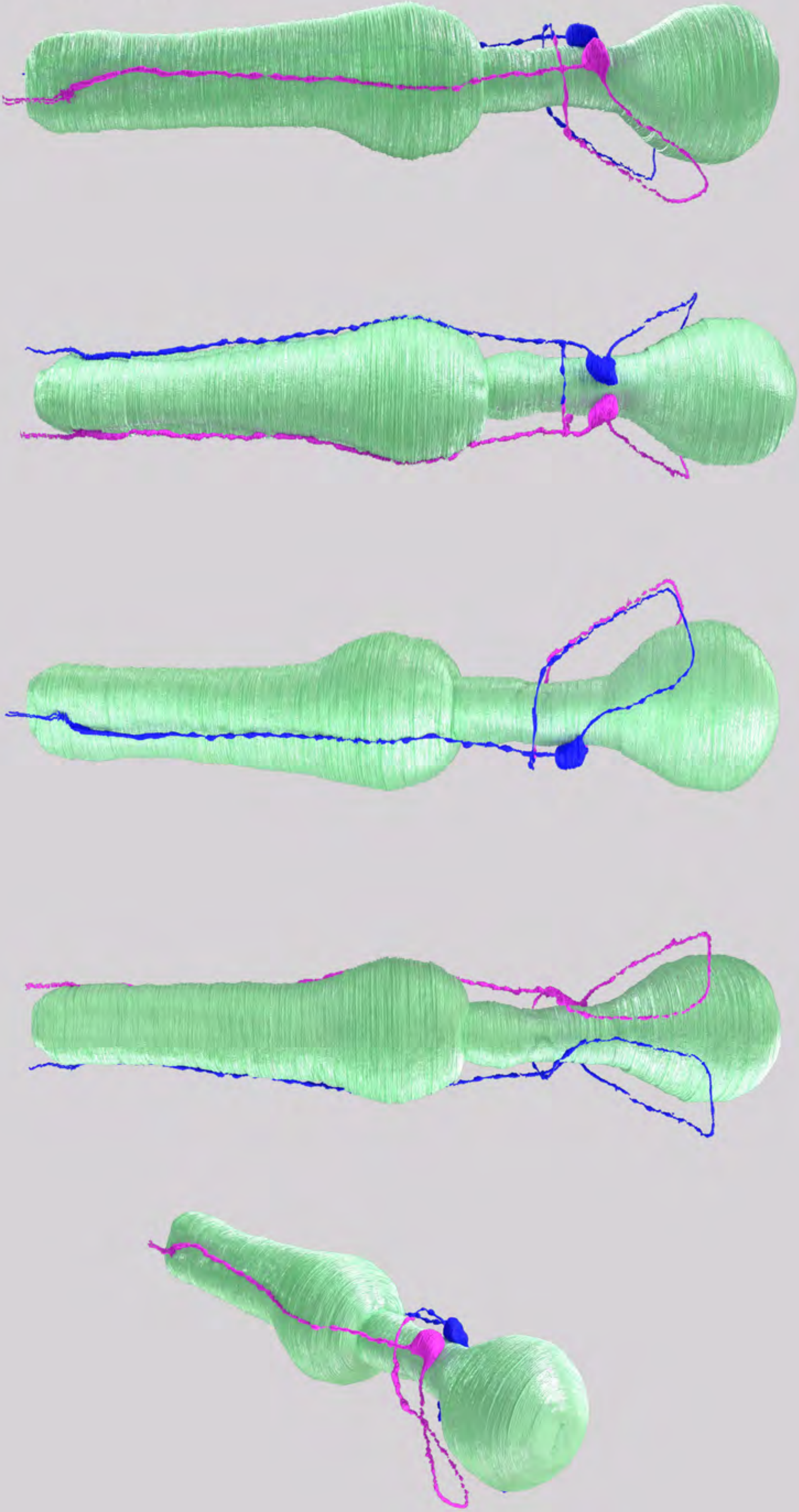

ADLL, ADLR

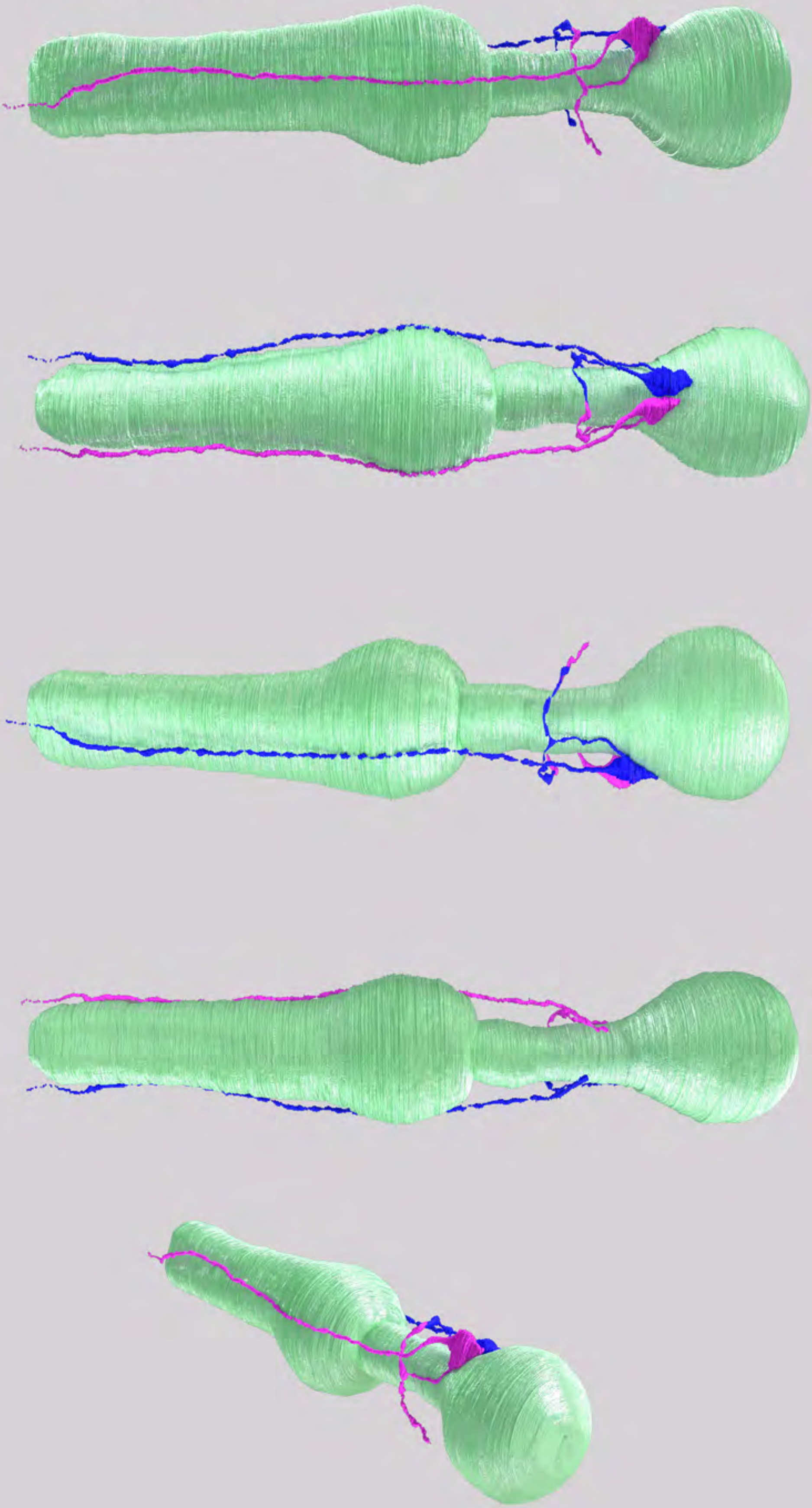

AFDL, AFDR

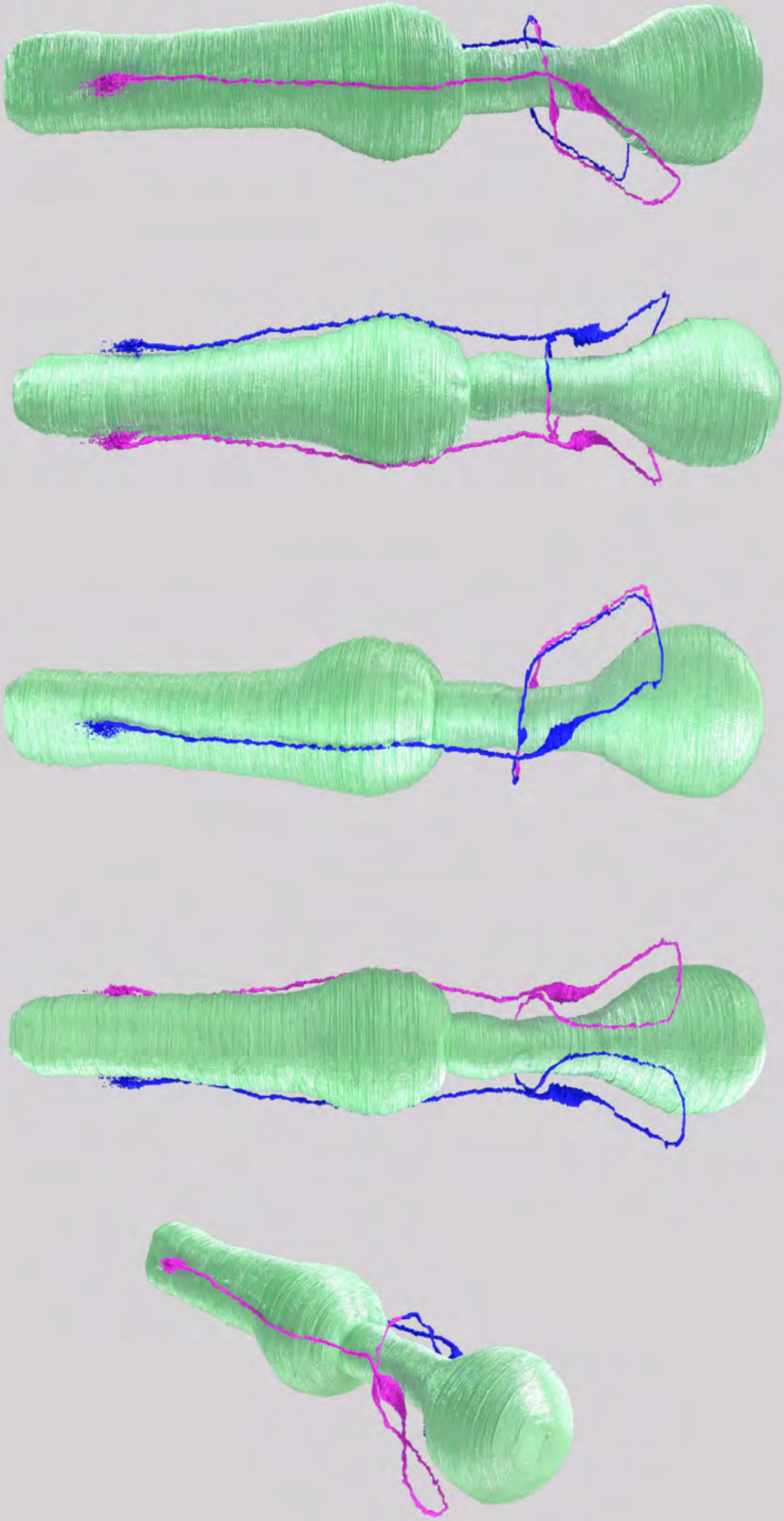

AIAL, AIAR

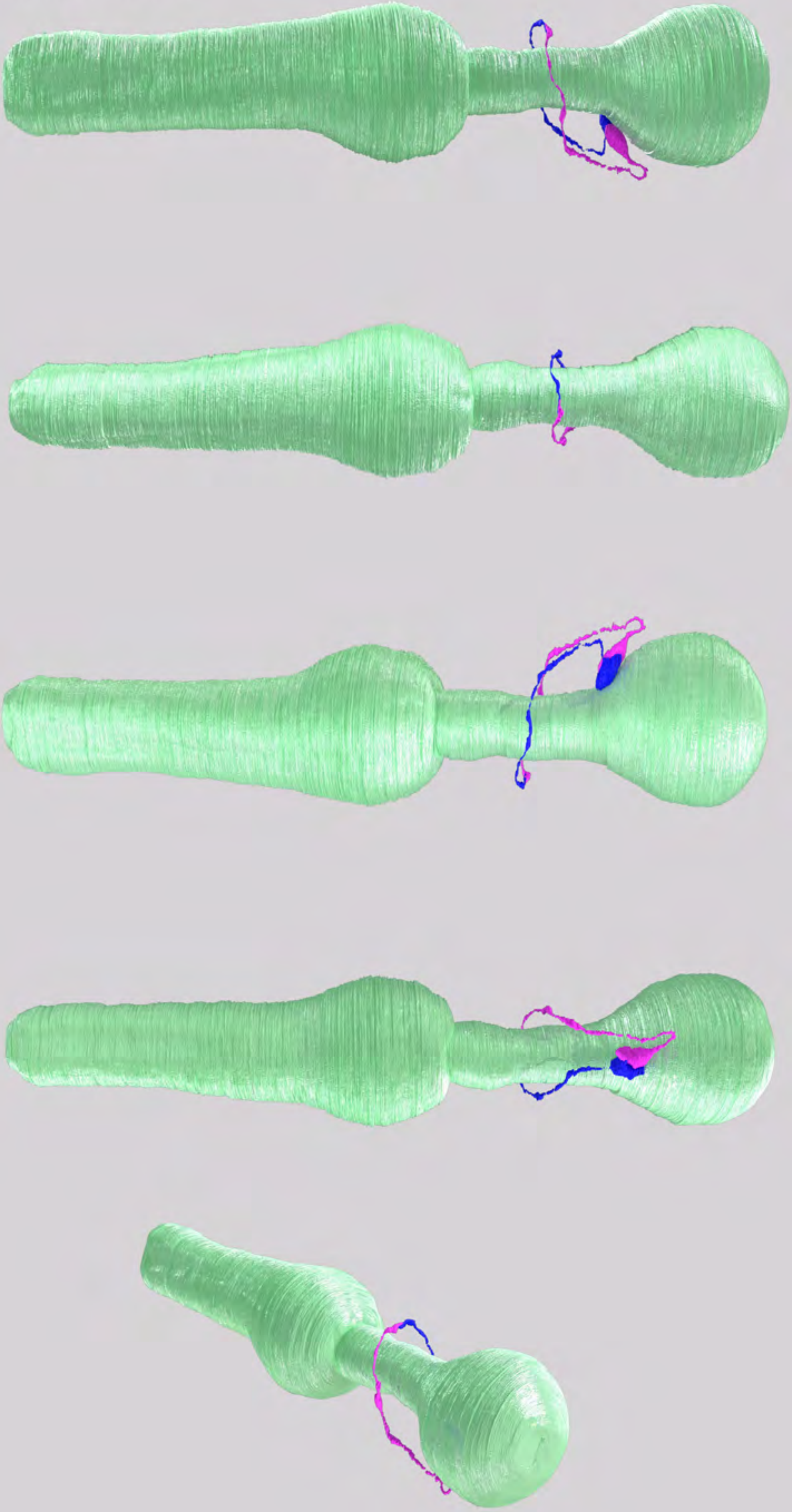

AIBL, AIBR

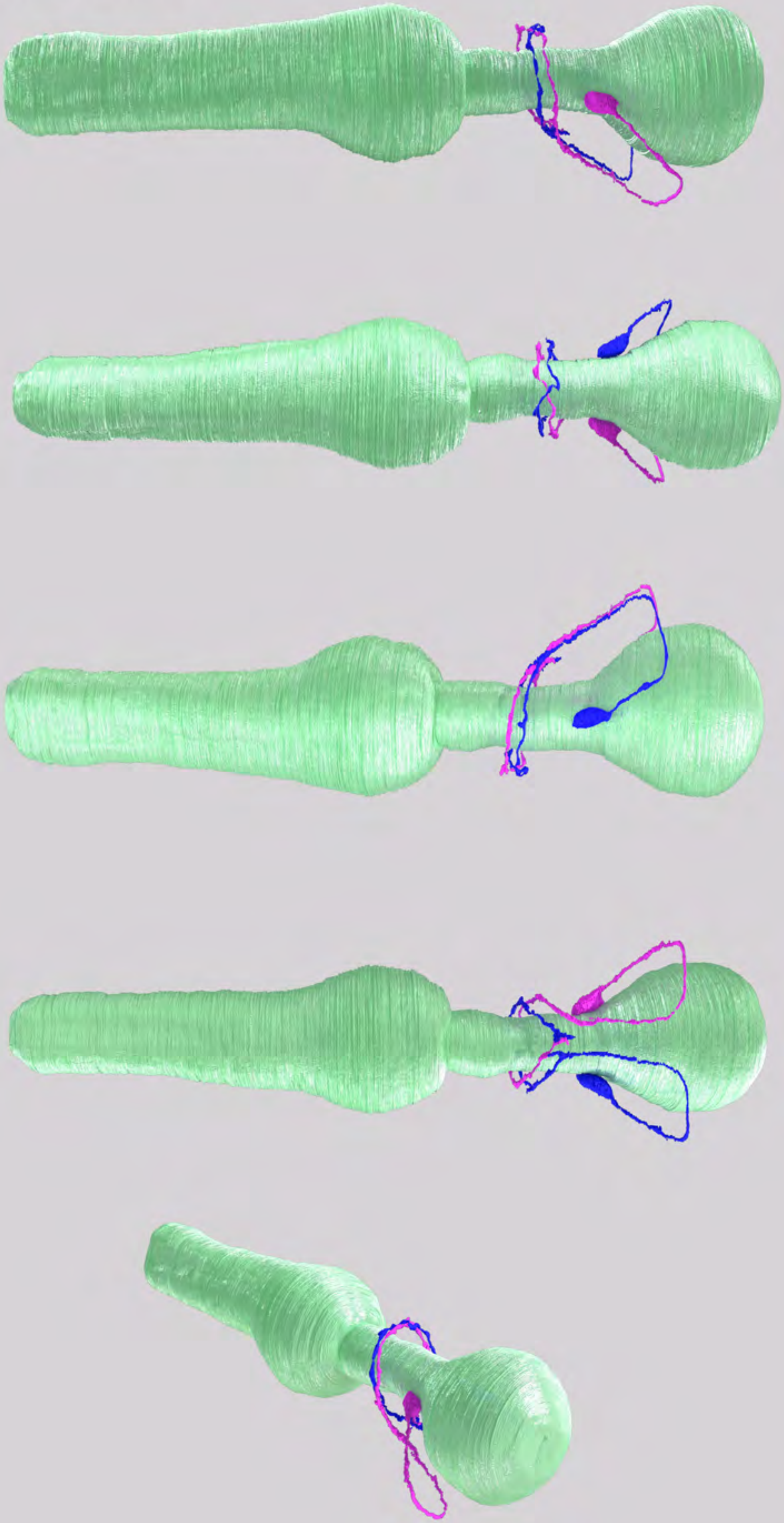

AIML, AIMR

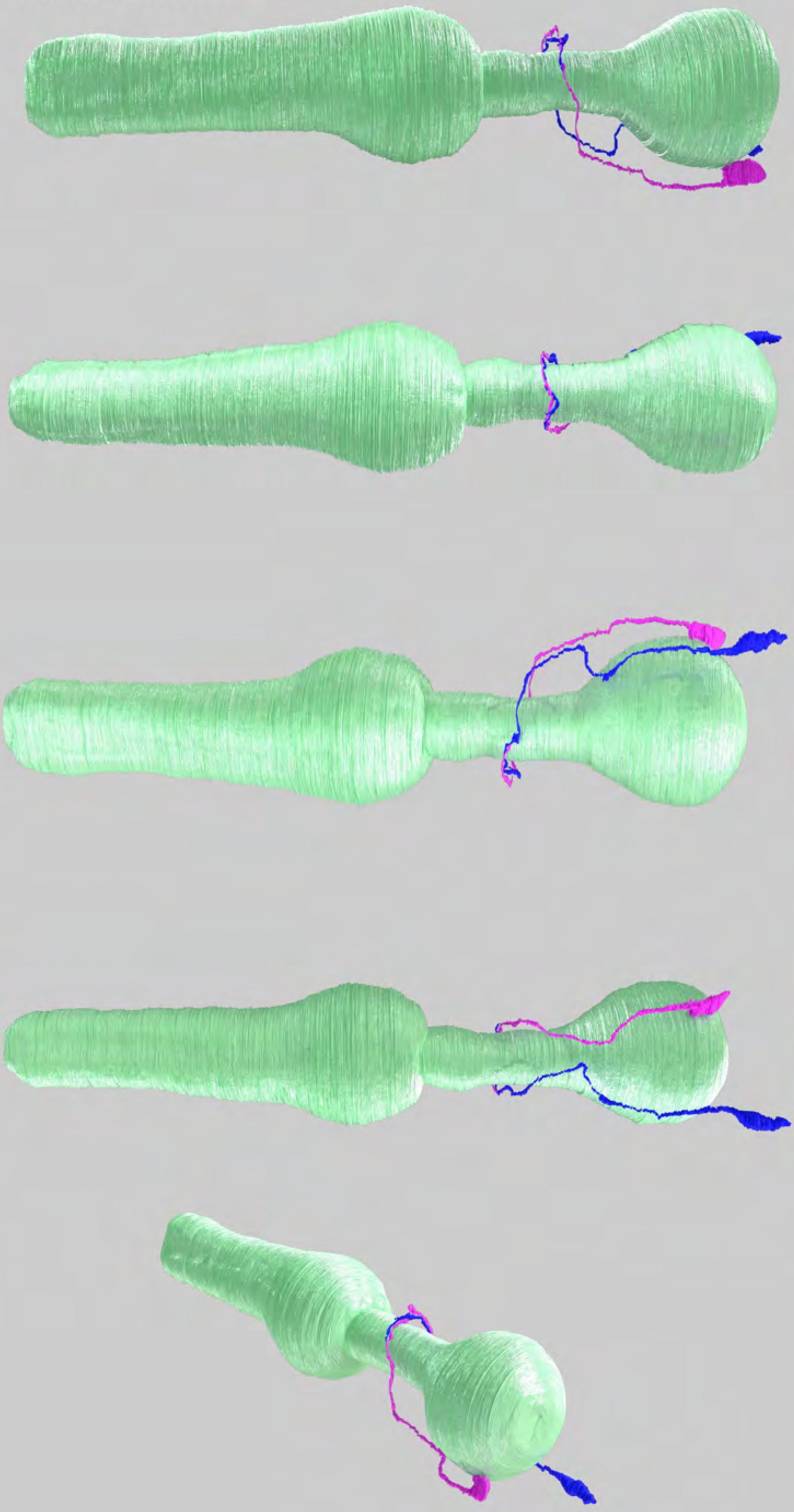

AINL, AINR

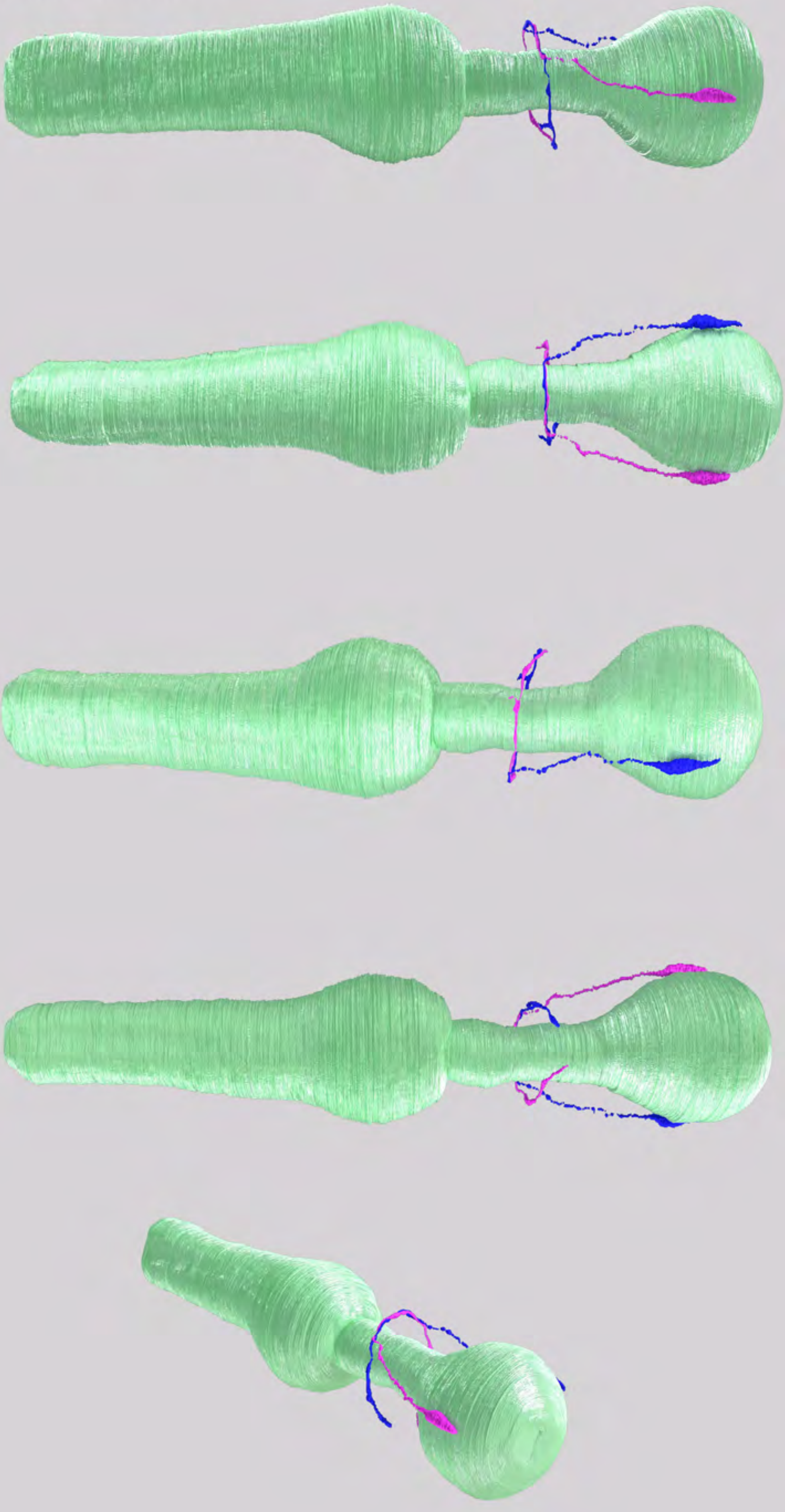

AIYL, AIYR

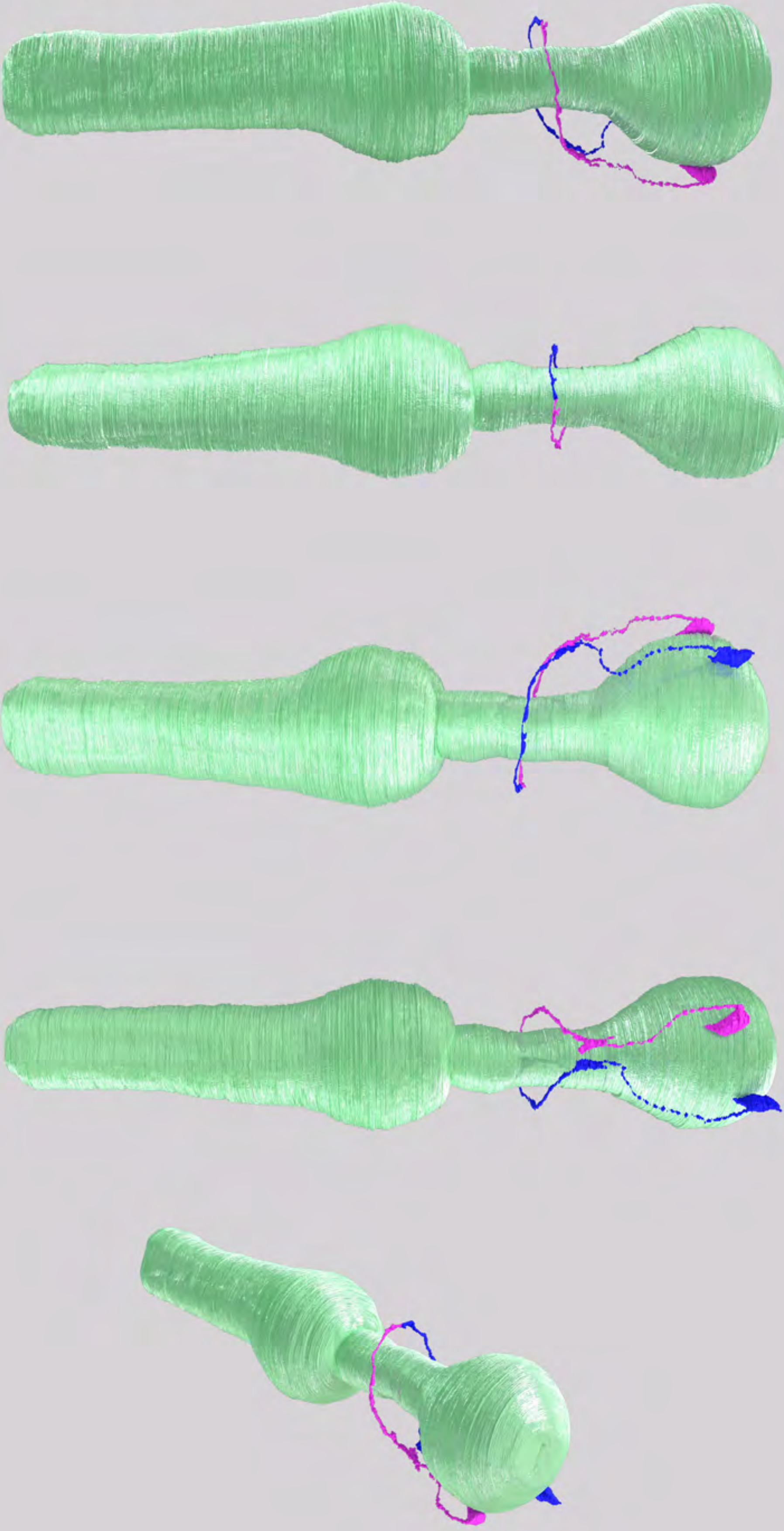

AIZL, AIZR

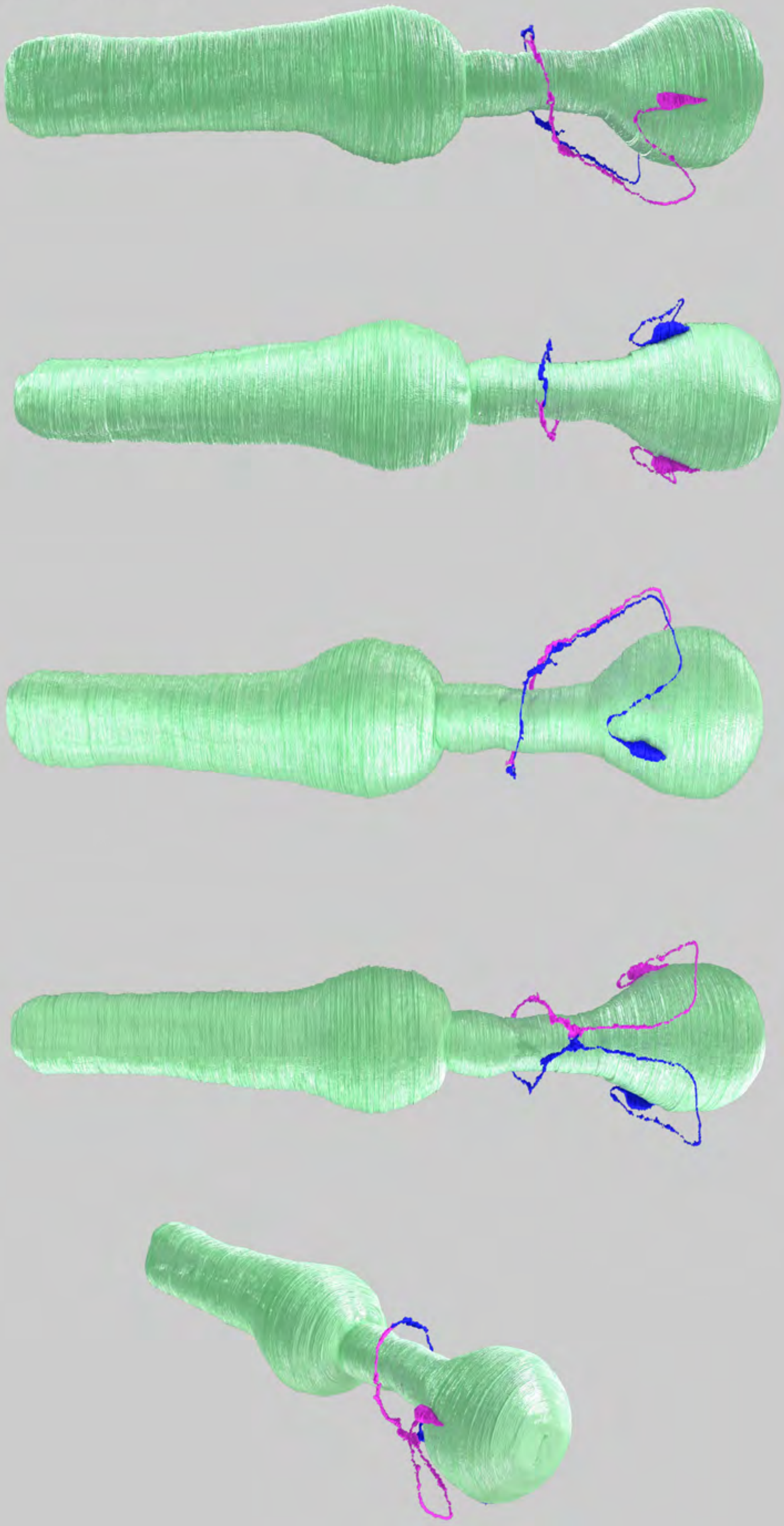

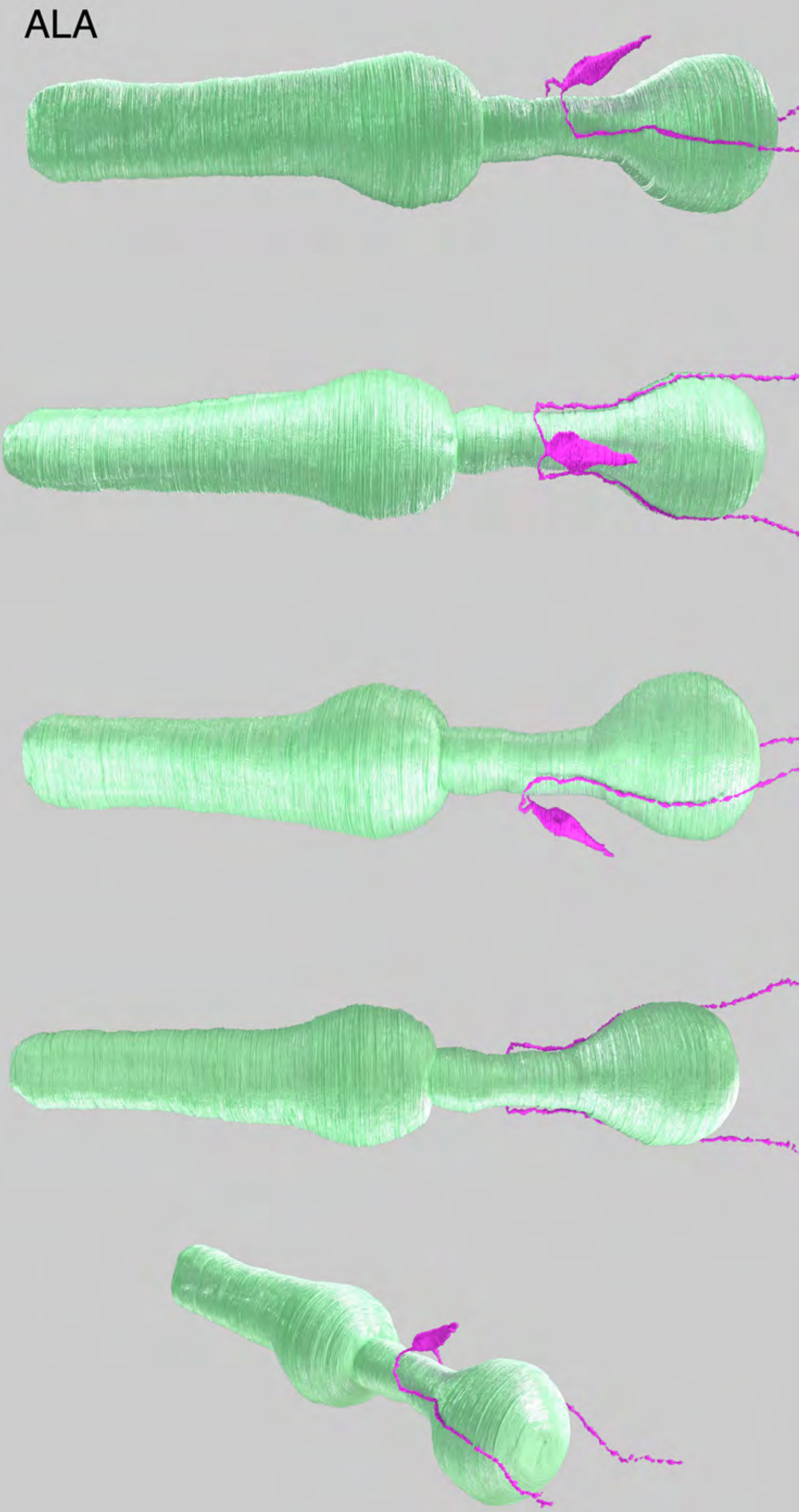
ALA

ALML, ALMR

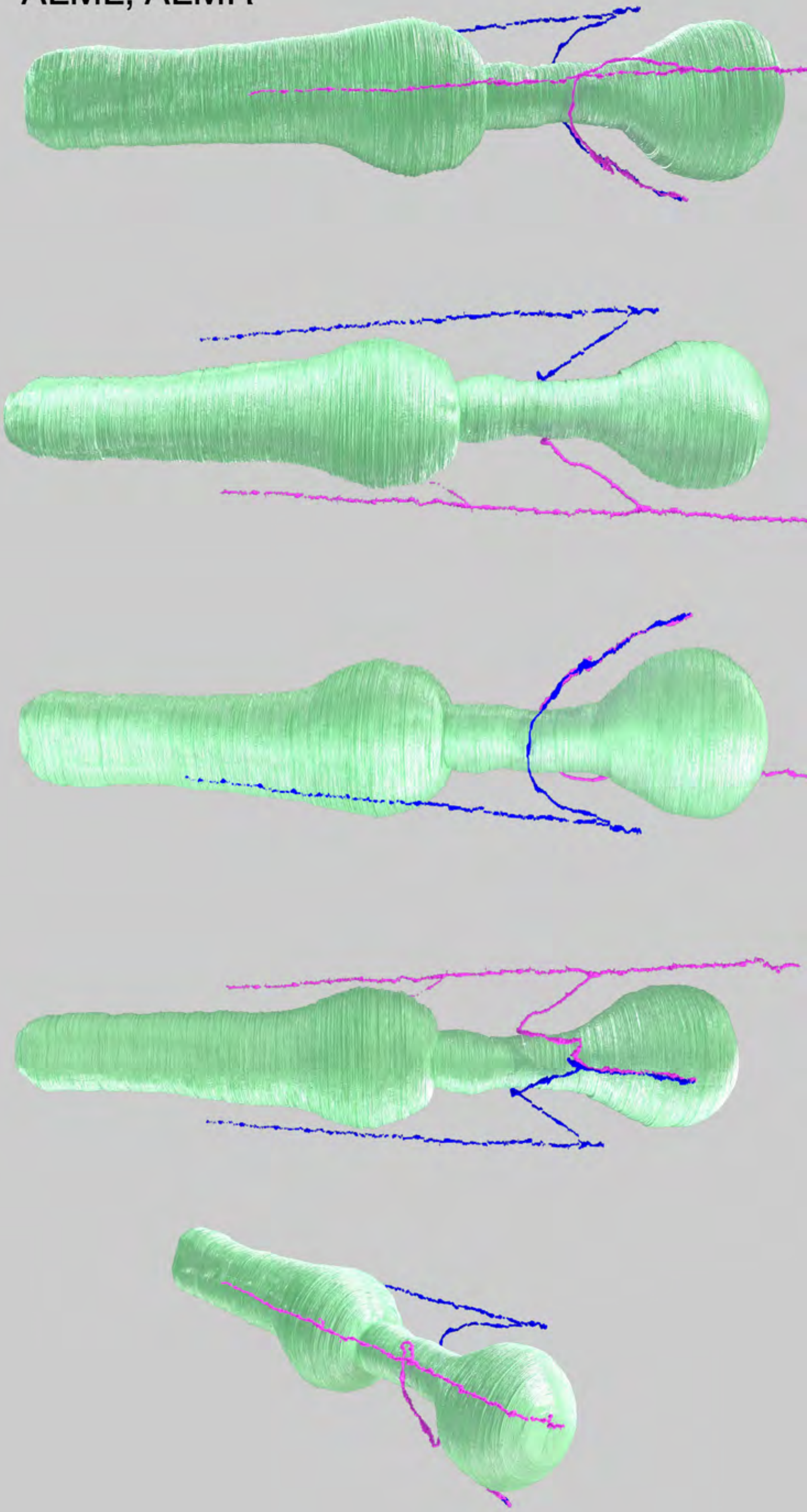

ALNL, ALNR

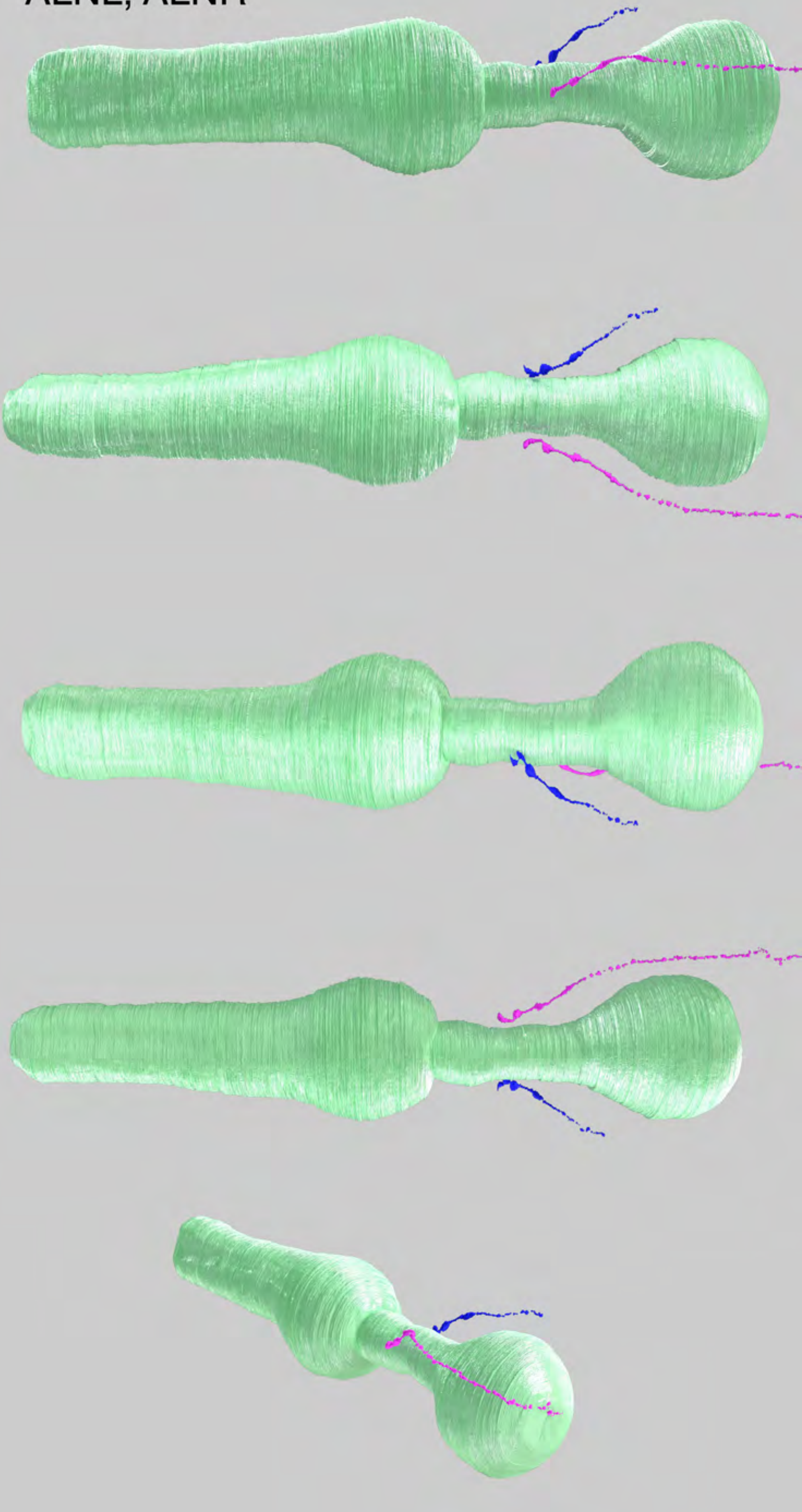

AQR

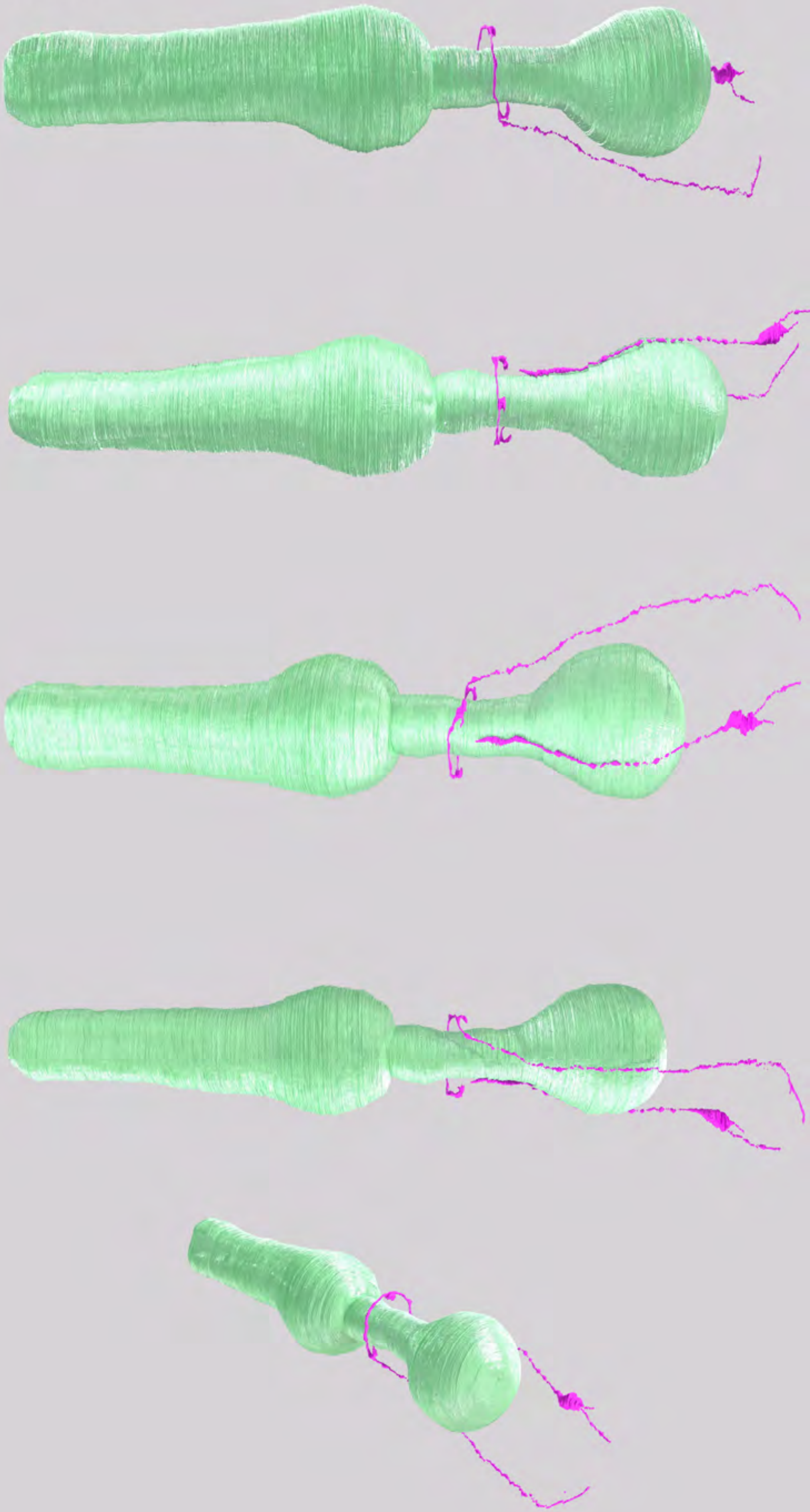

ASEL, ASER

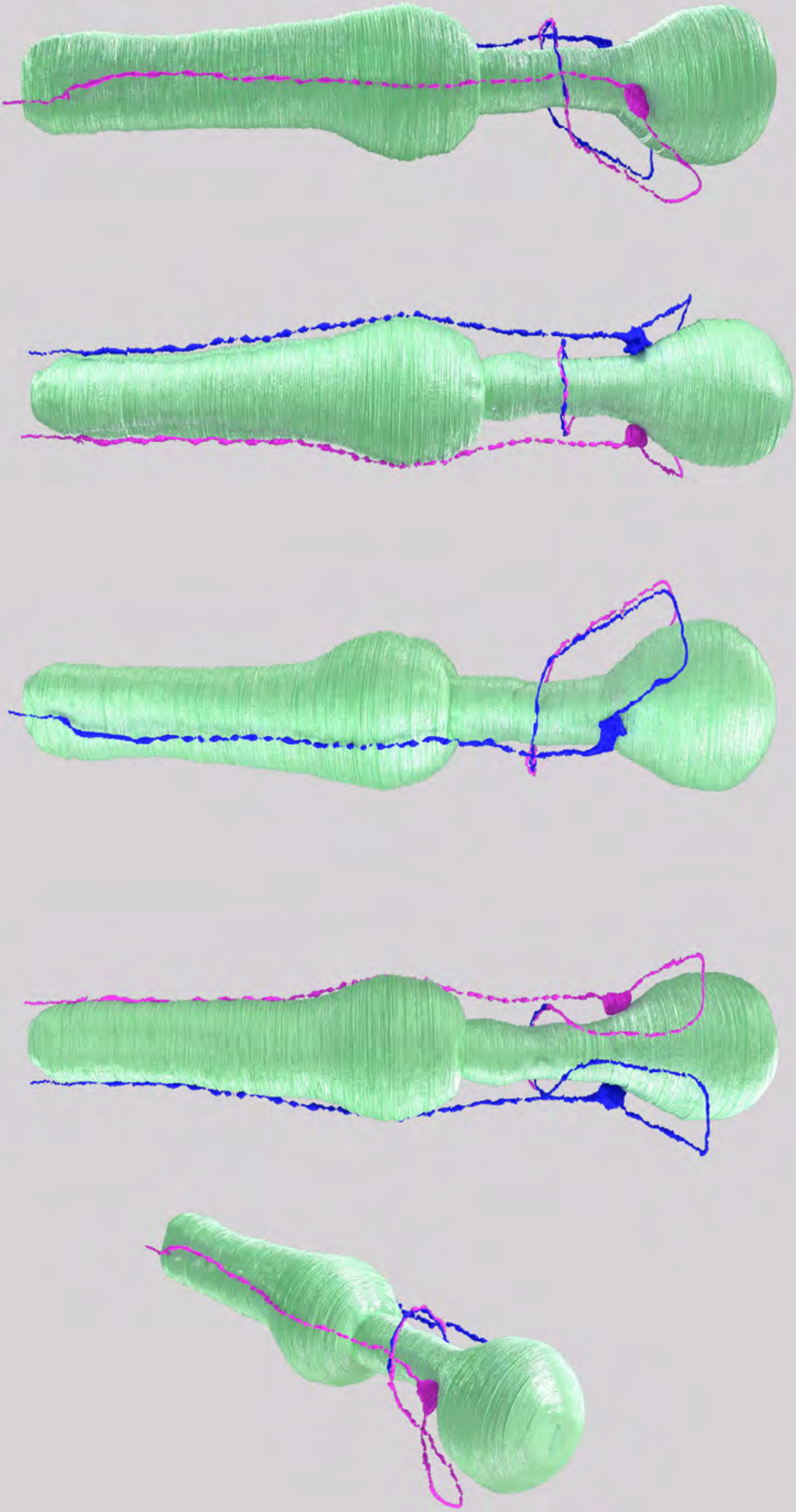

ASGL, ASGR

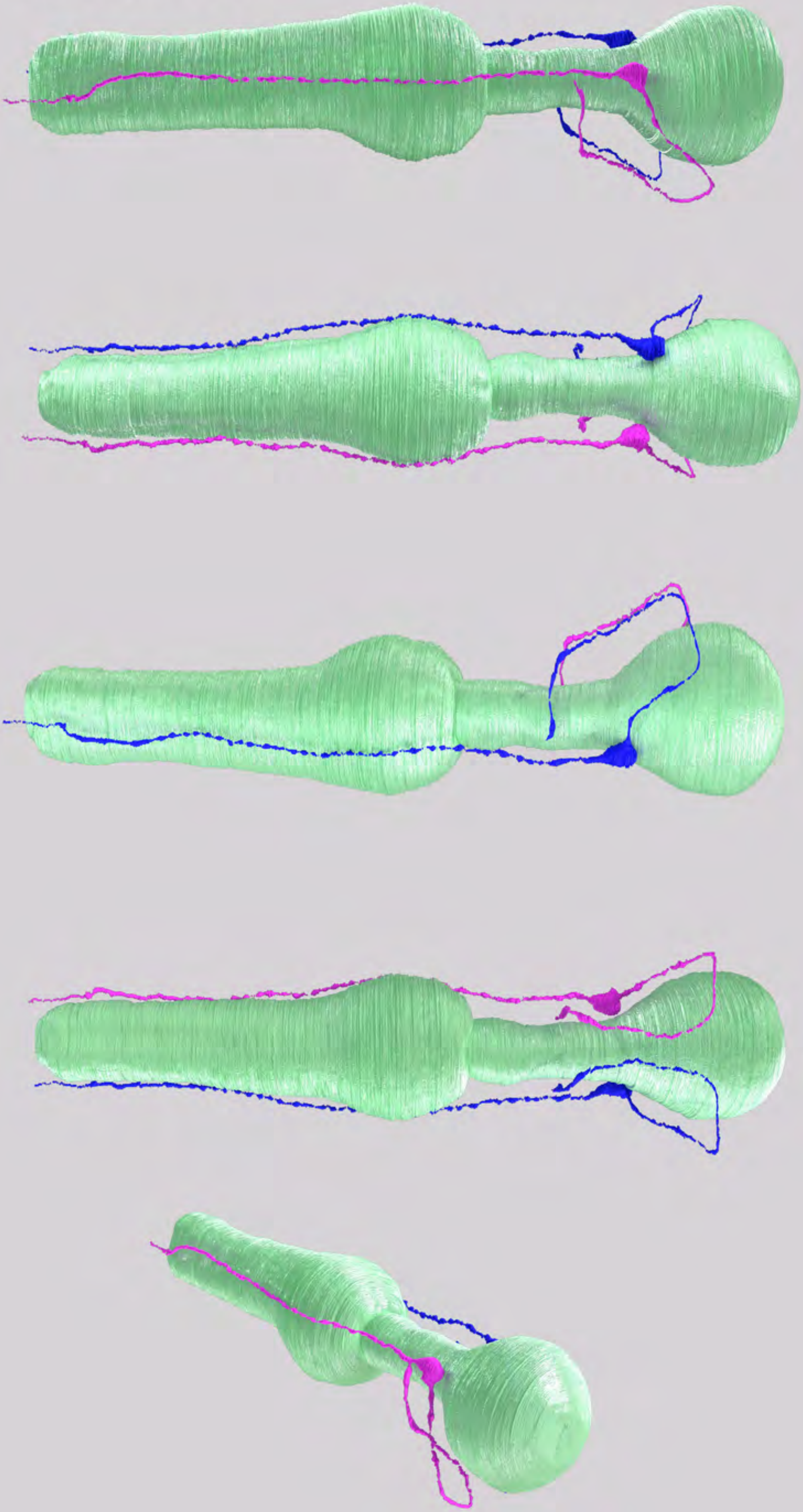

ASHL, ASHR

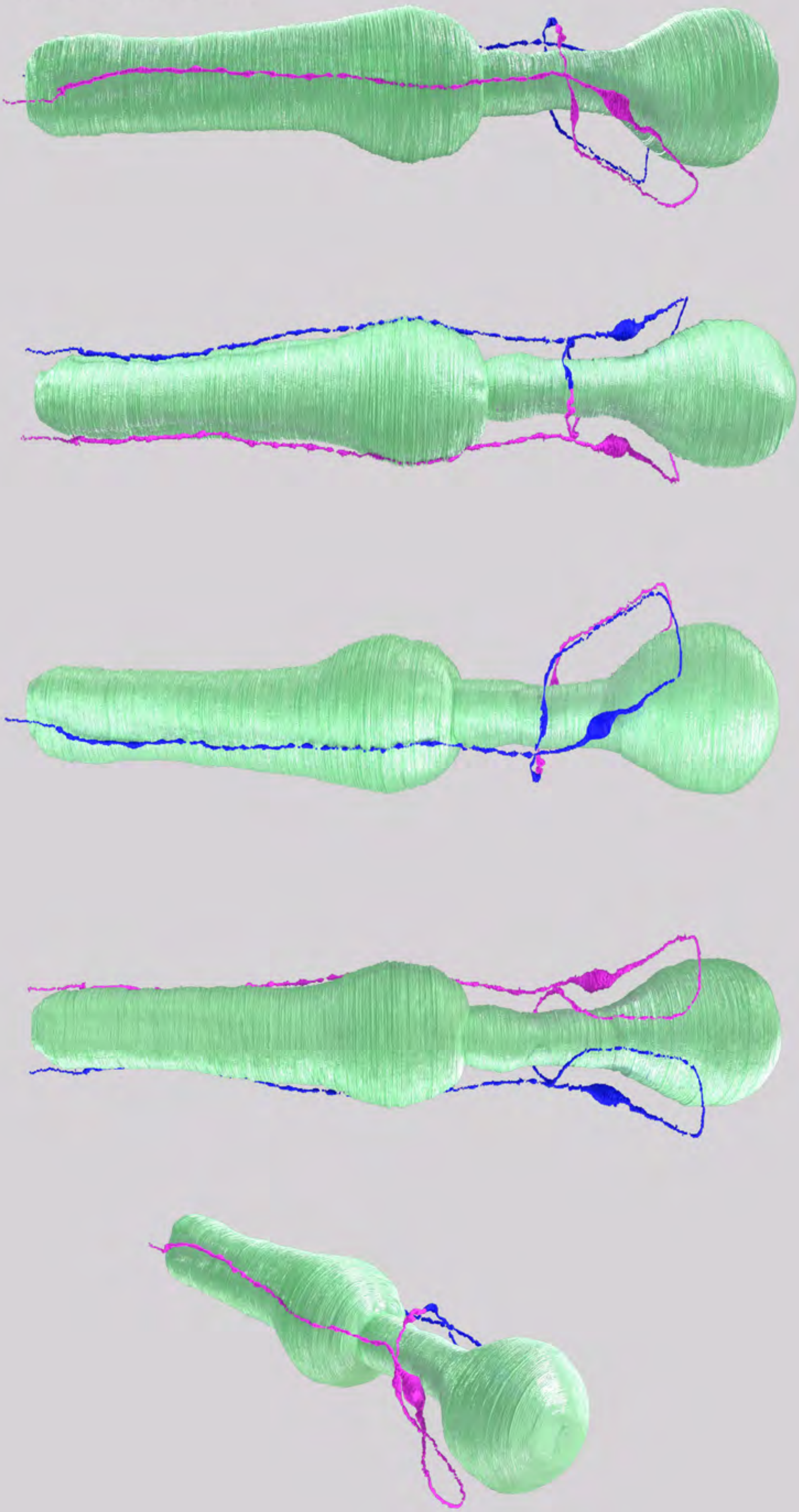

ASIL, ASIR

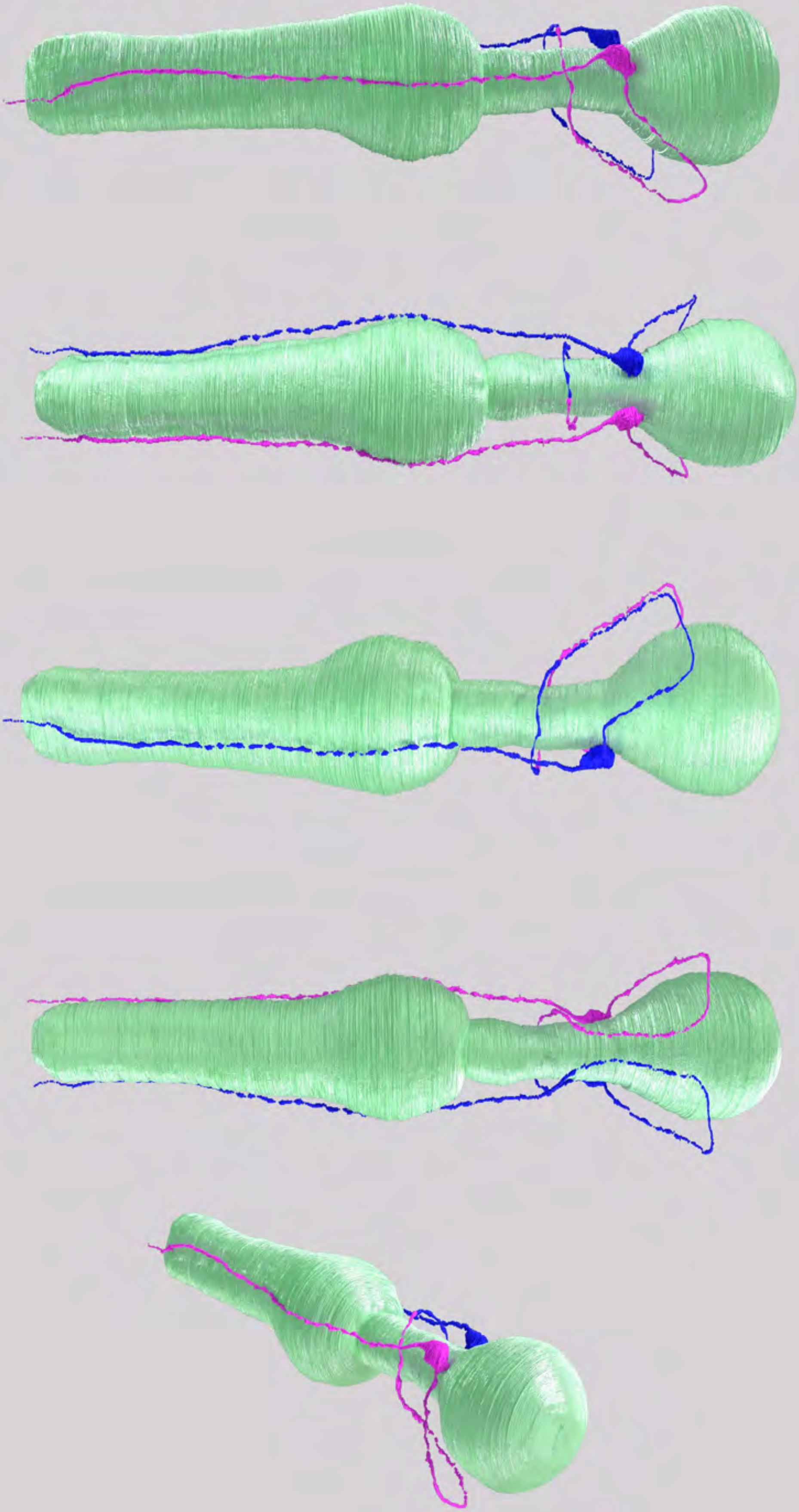

ASJL, ASJR

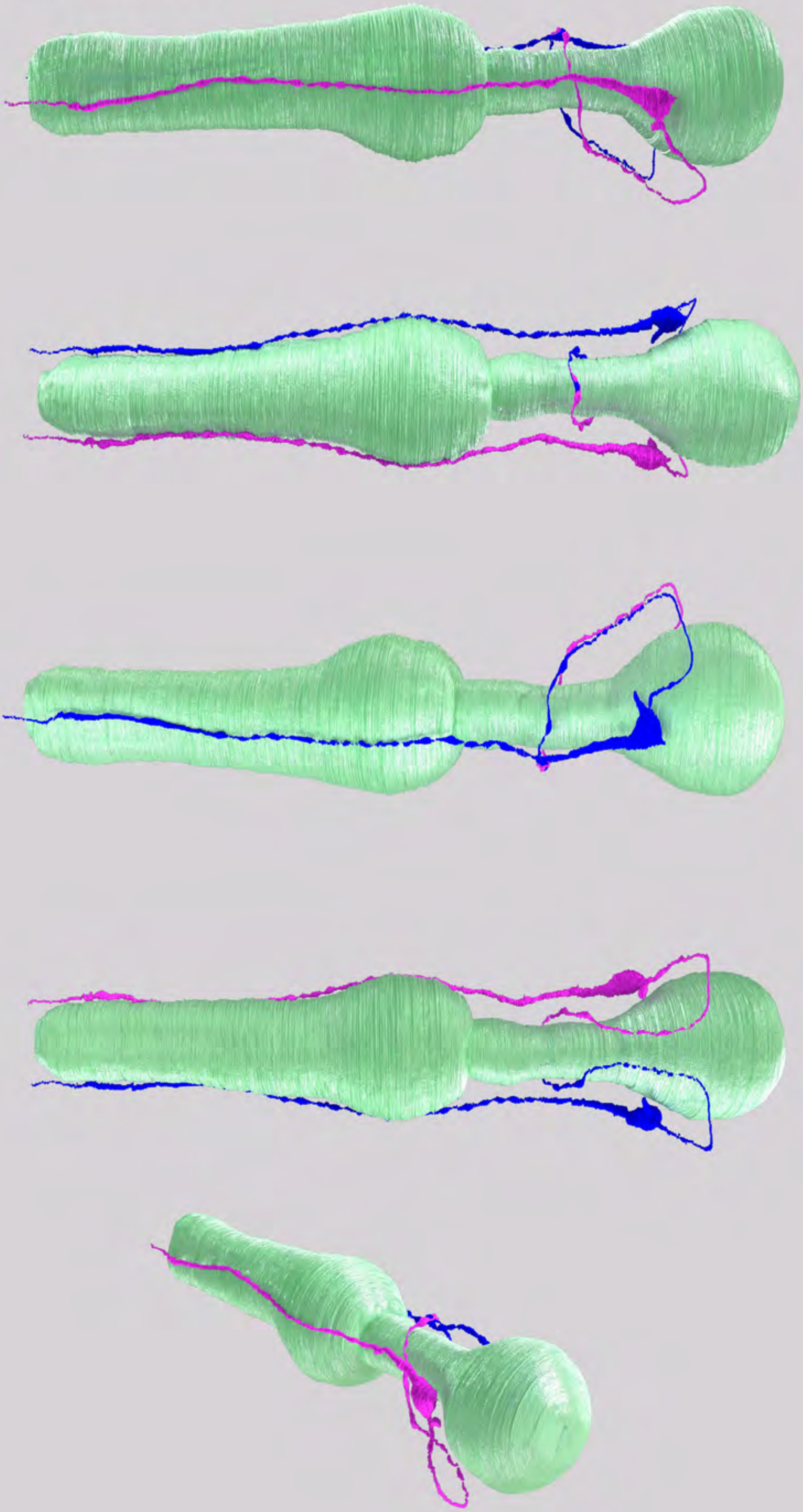

ASKL, ASKR

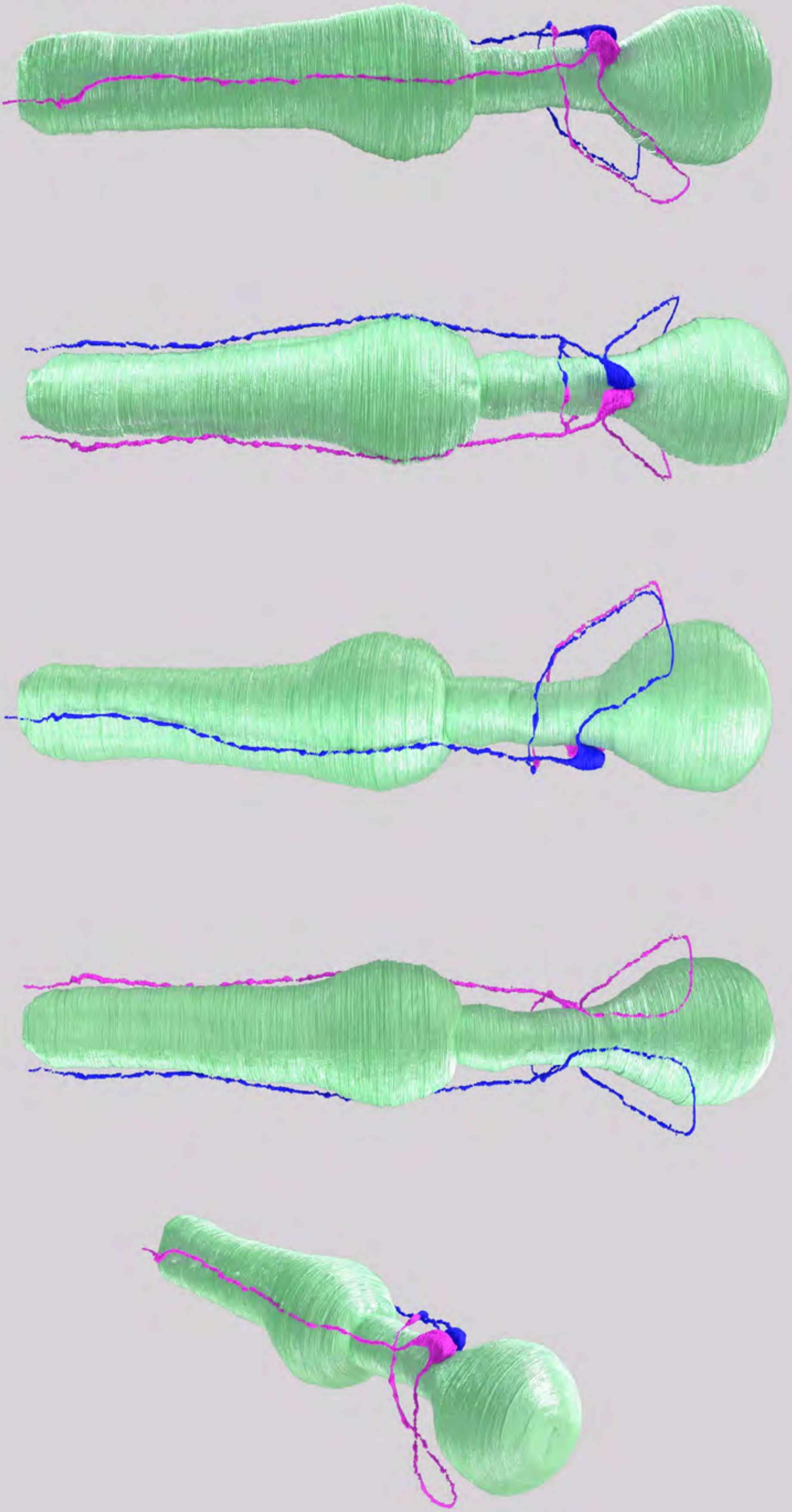

AUAL, AUAR

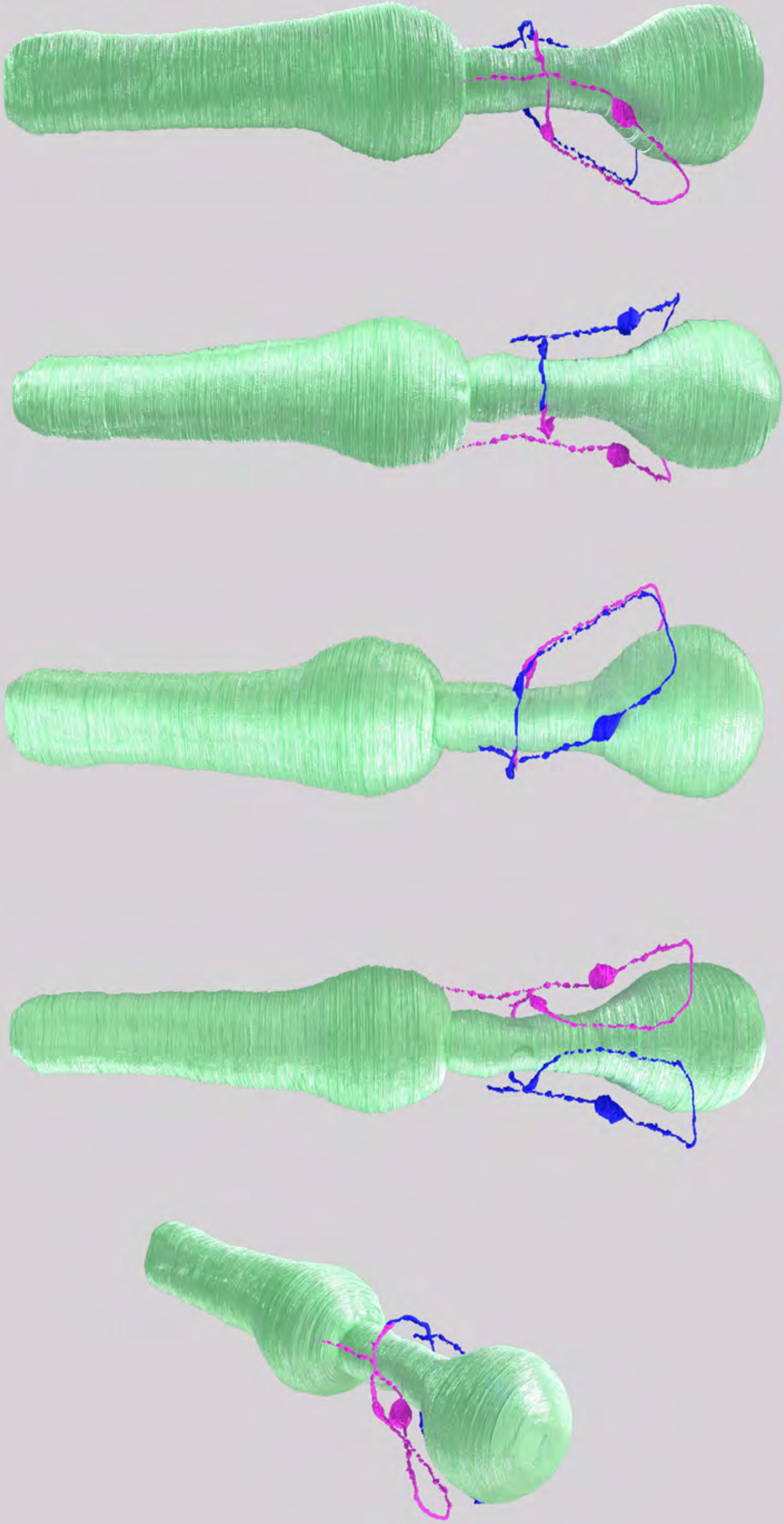

AVAL, AVAR

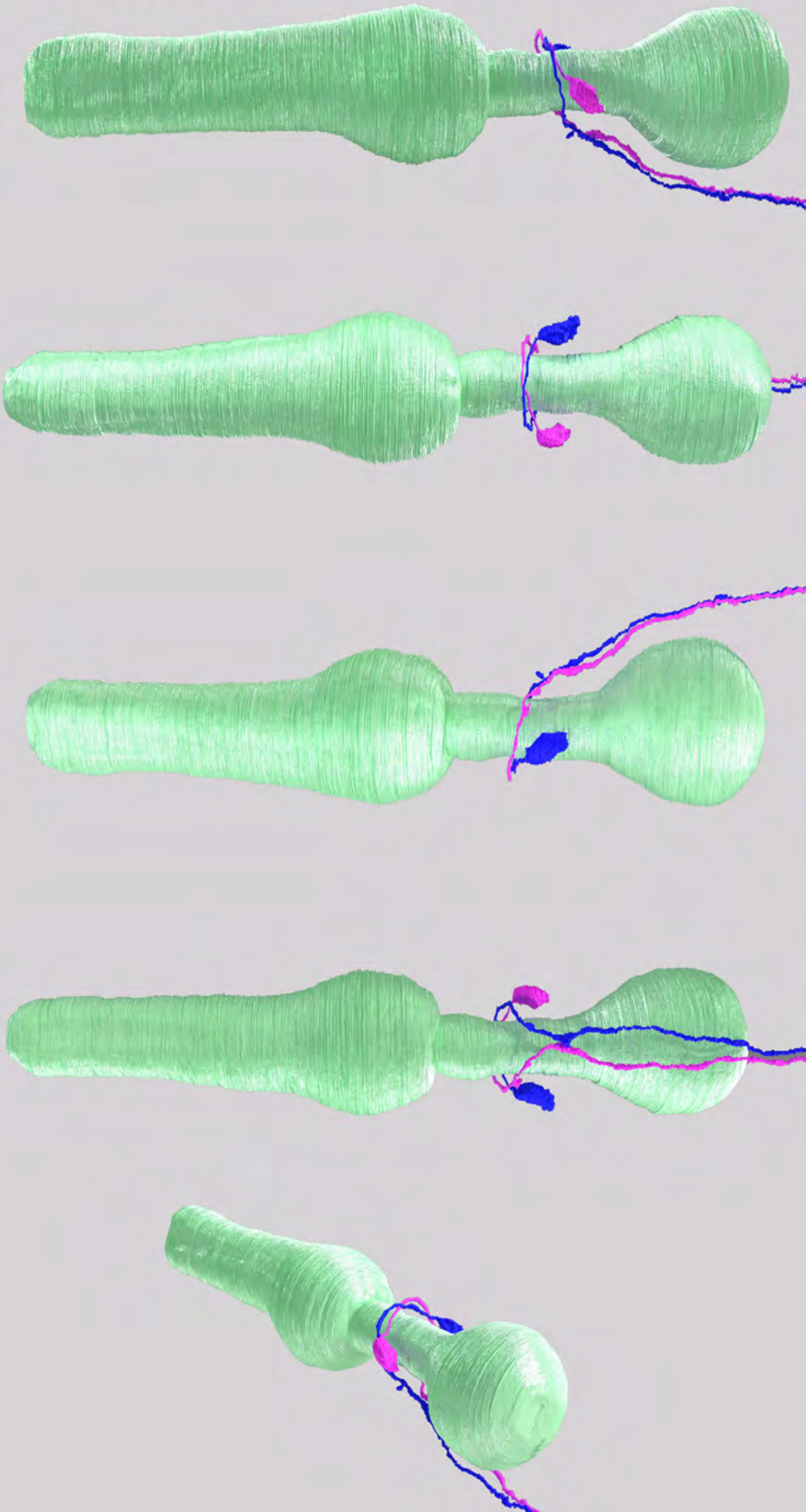

AVBL, AVBR

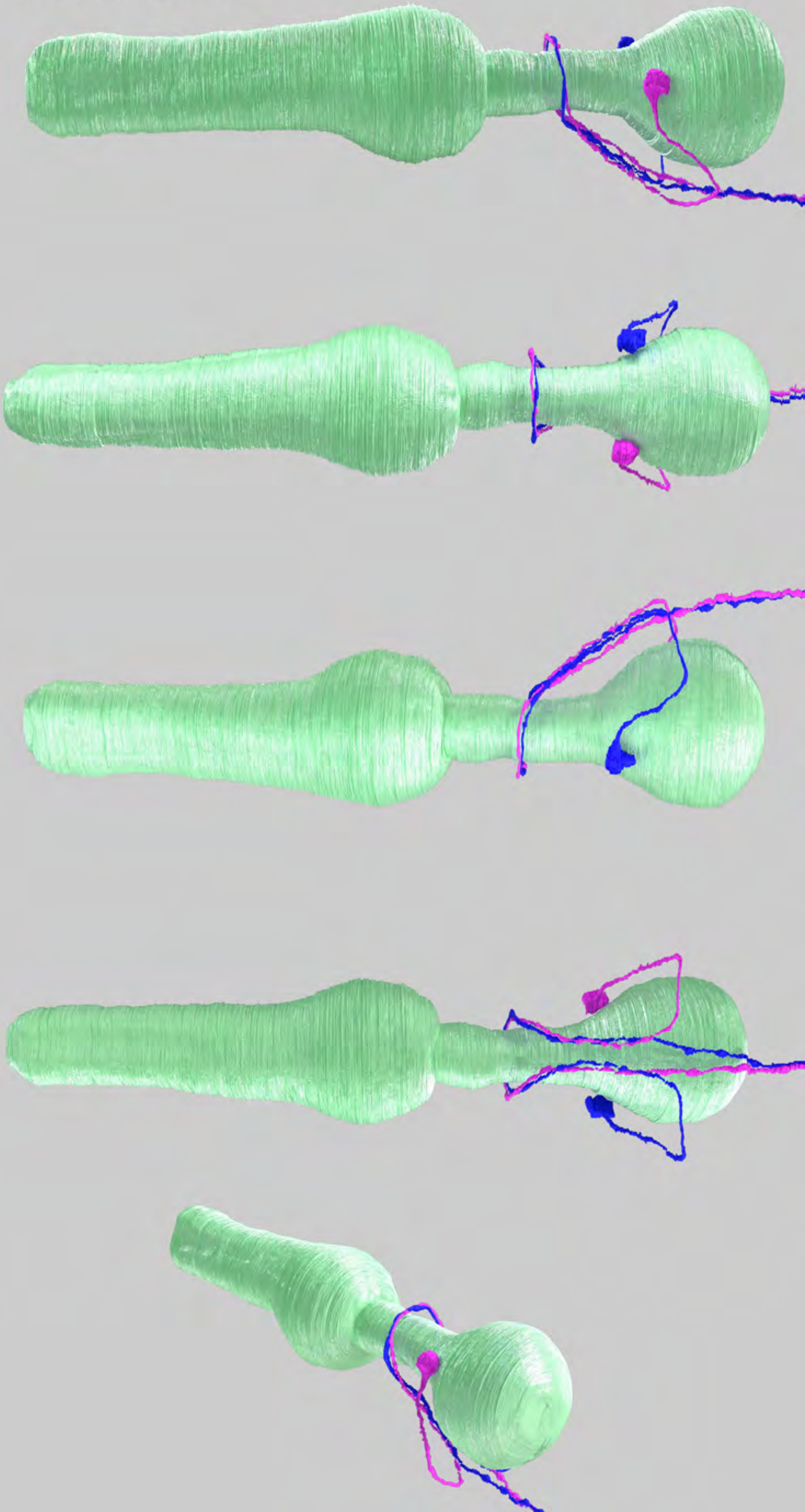

AVDL, AVDR

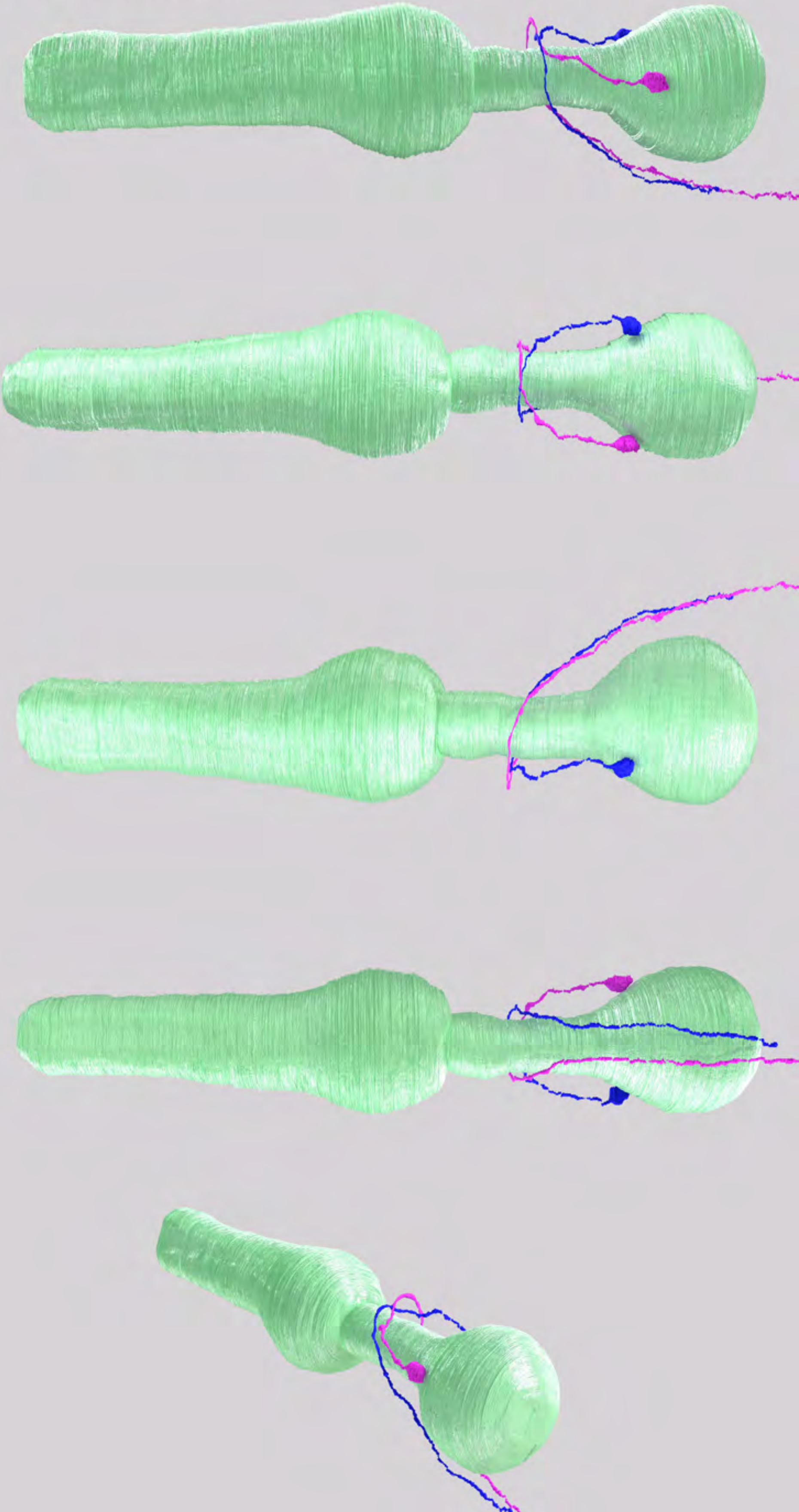

AVEL, AVER

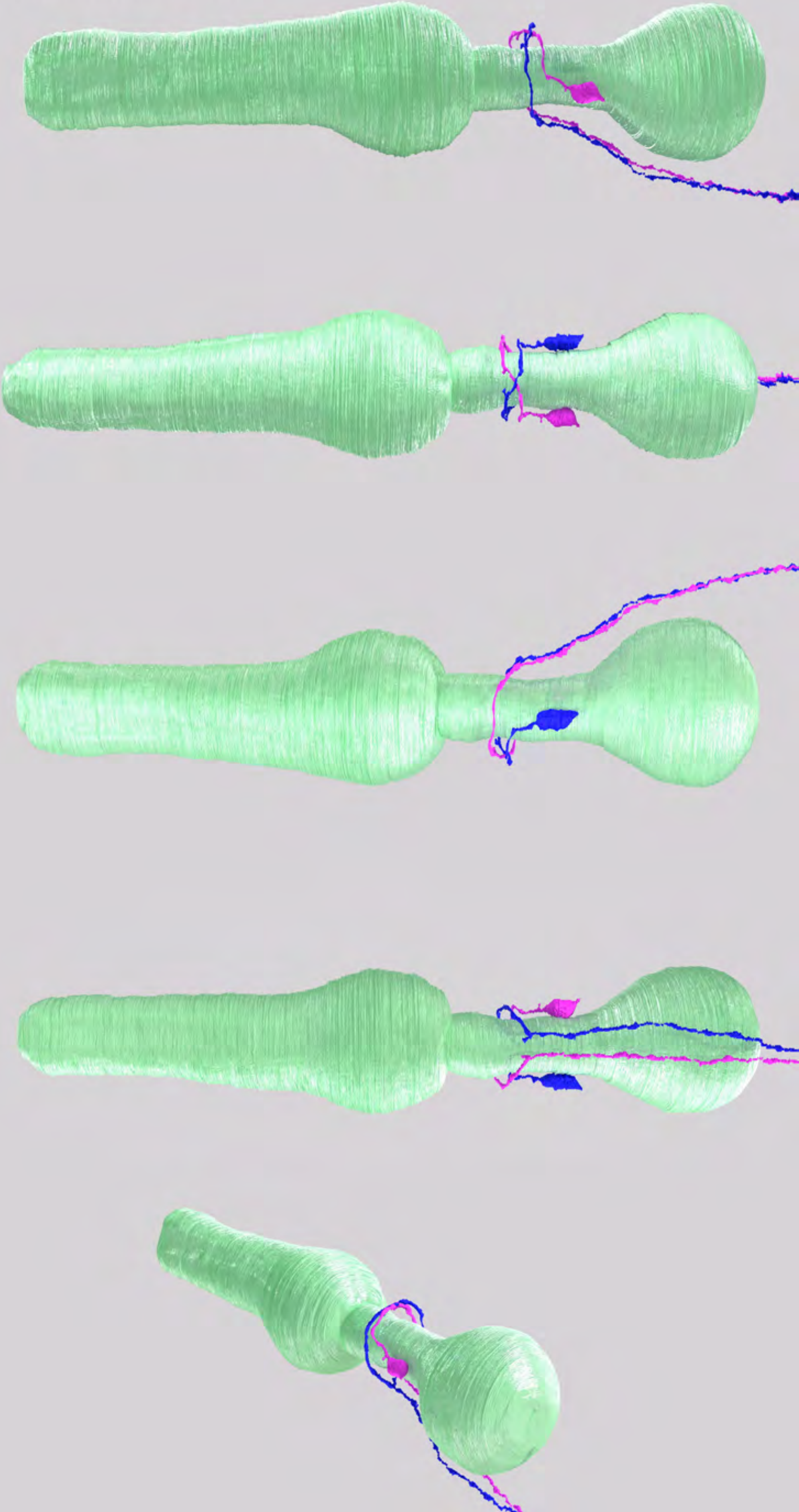

AVFL, AVFR

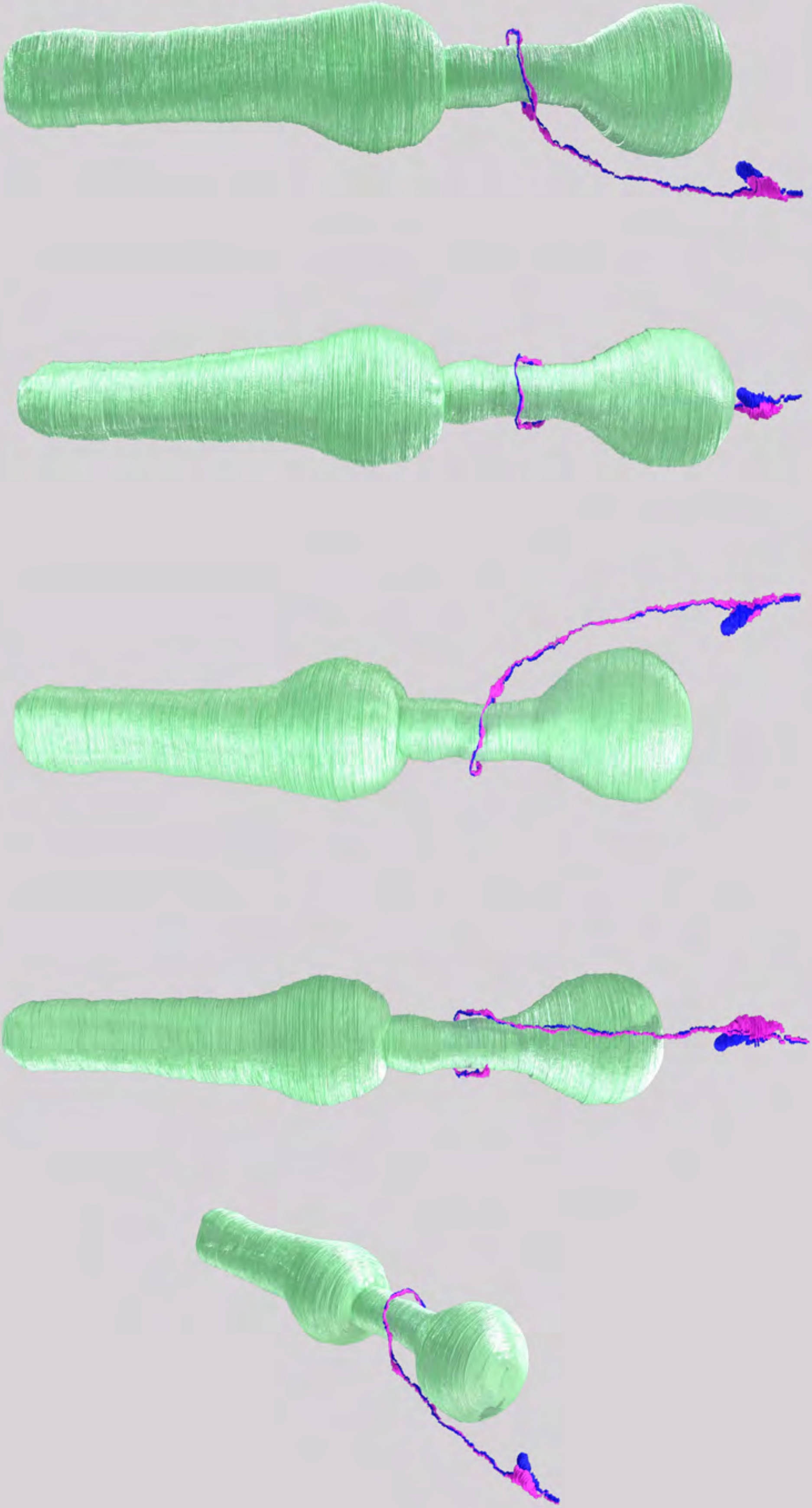

AVJL, AVJR

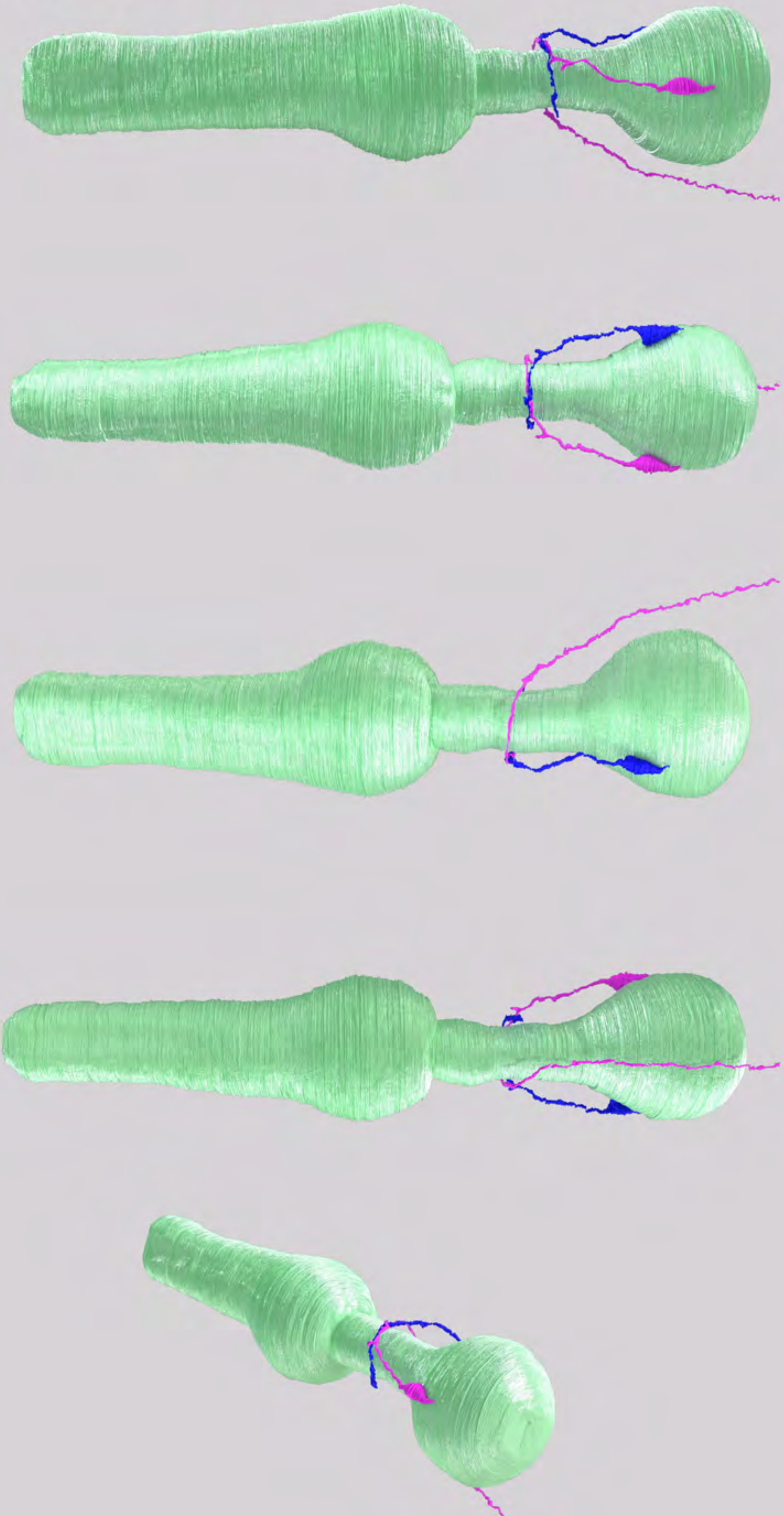

AVKL, AVKR

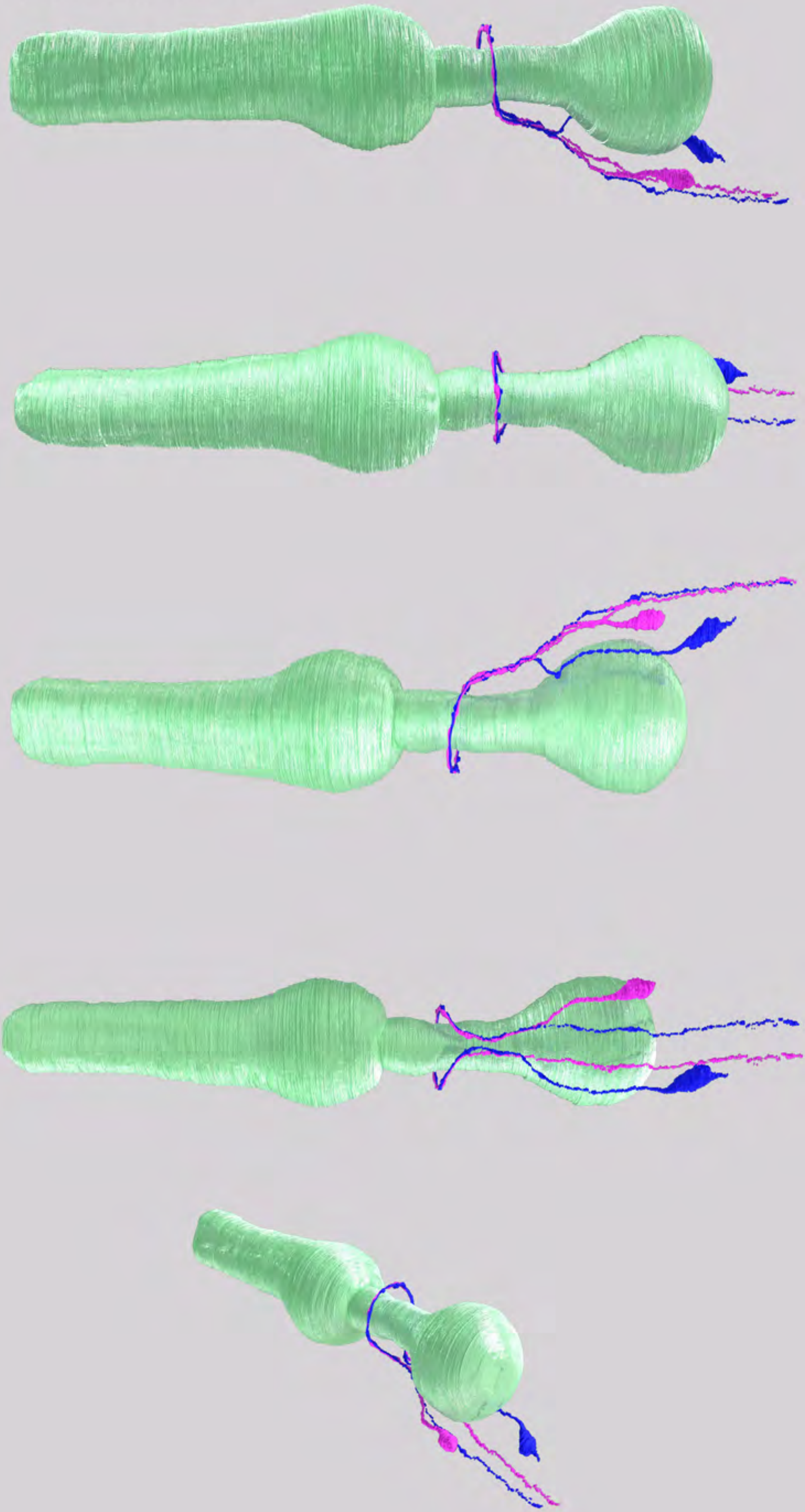

AVL

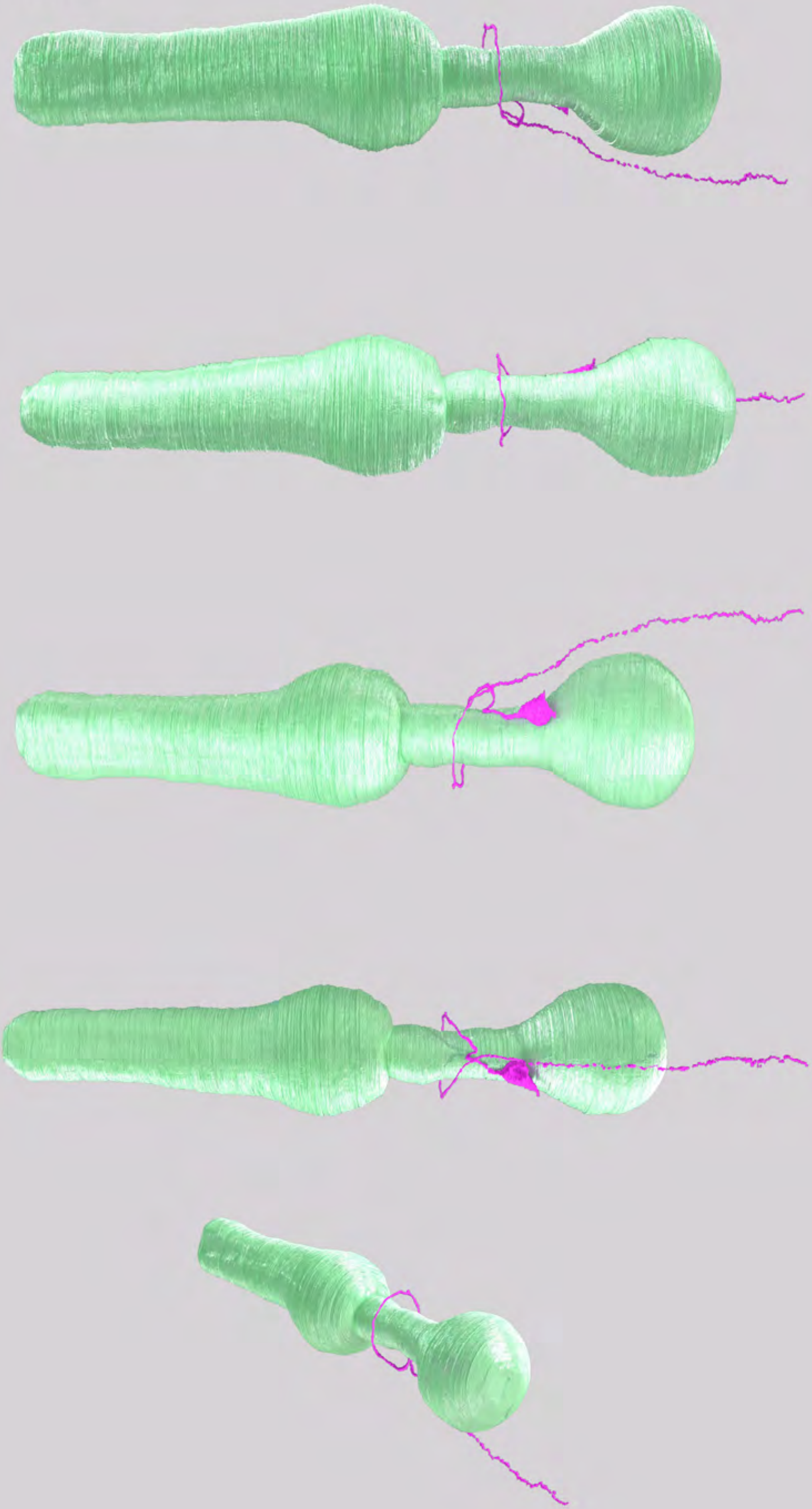

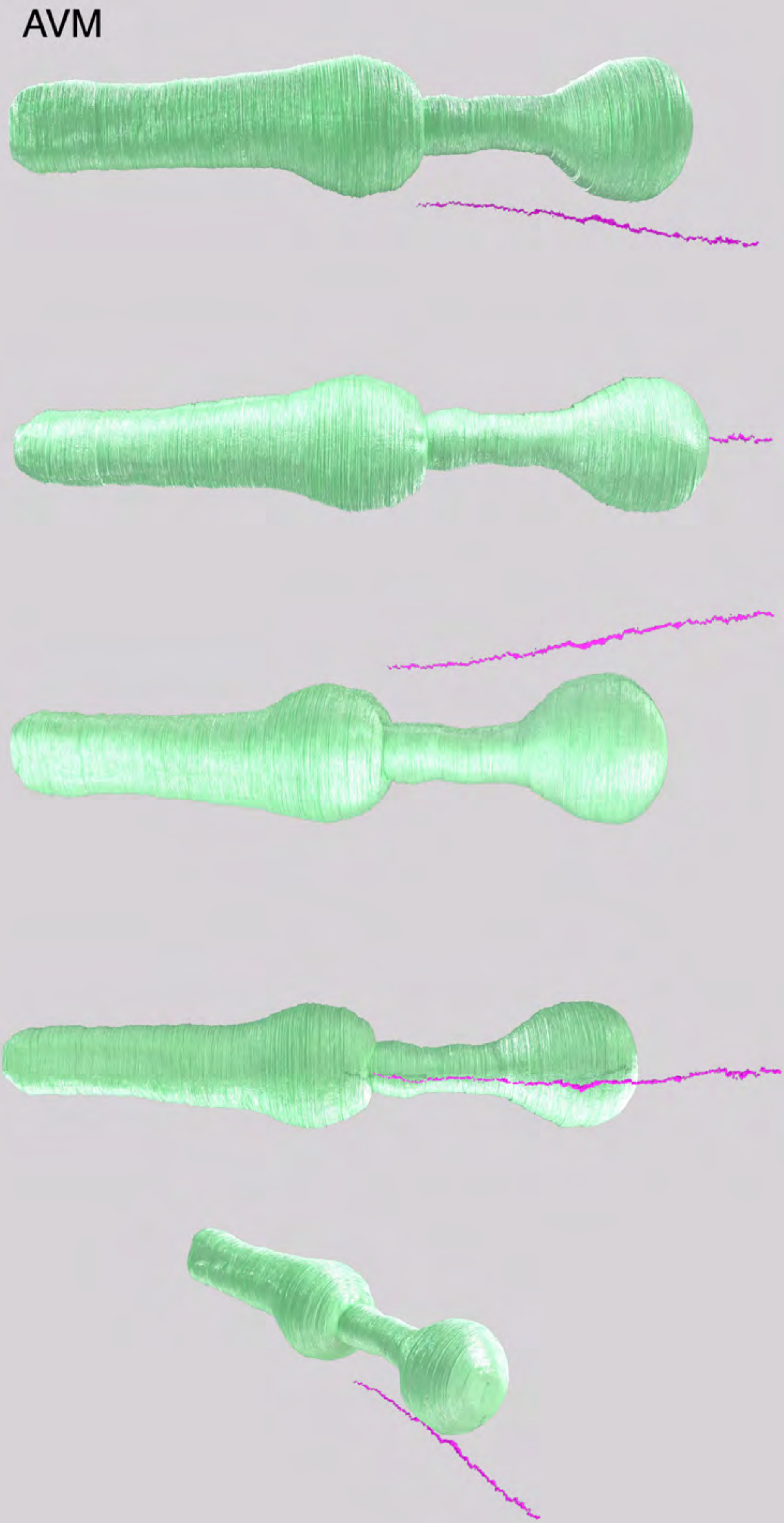
AVM

AWAL, AWAR

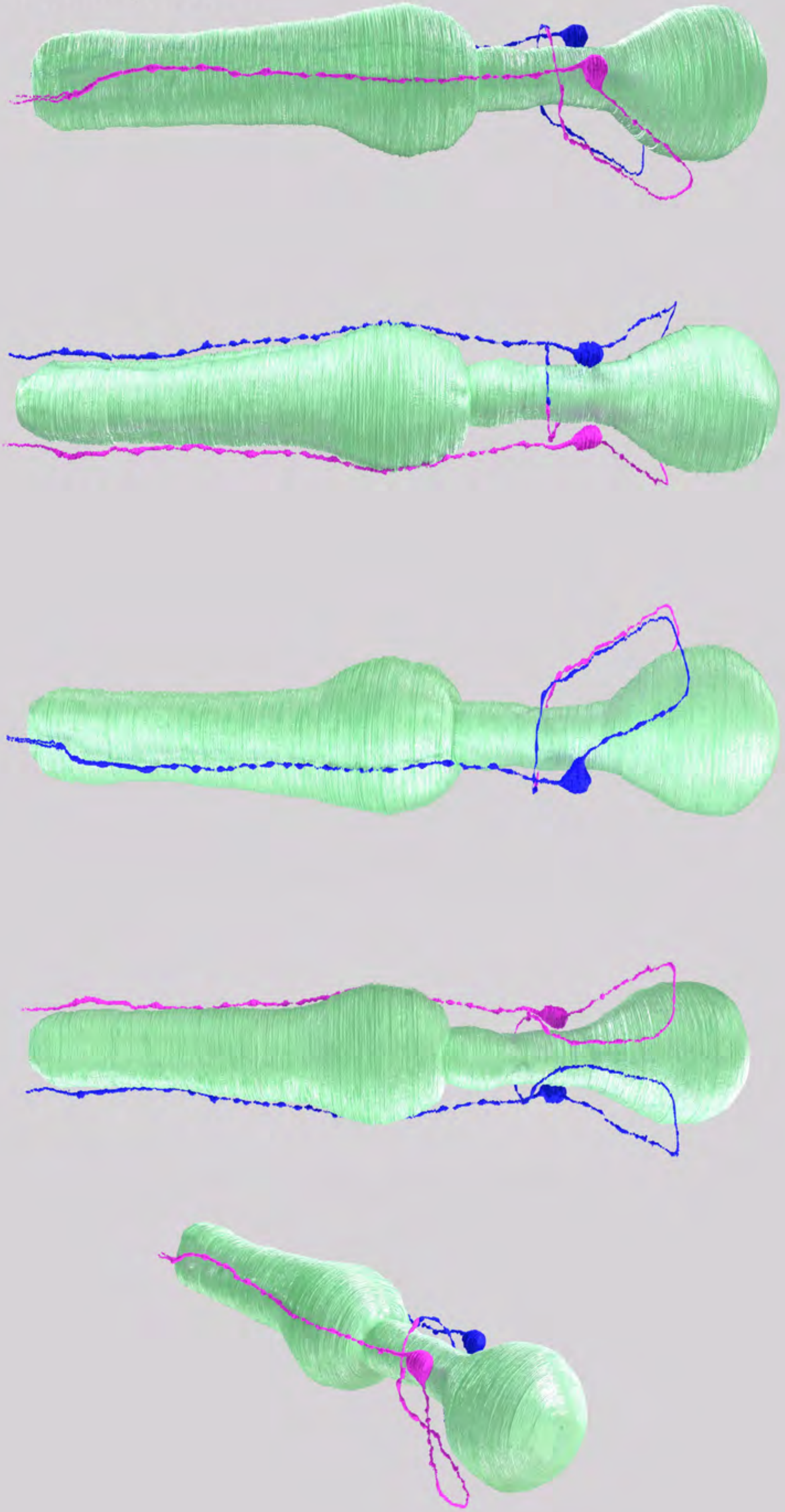

AWBL, AWBR

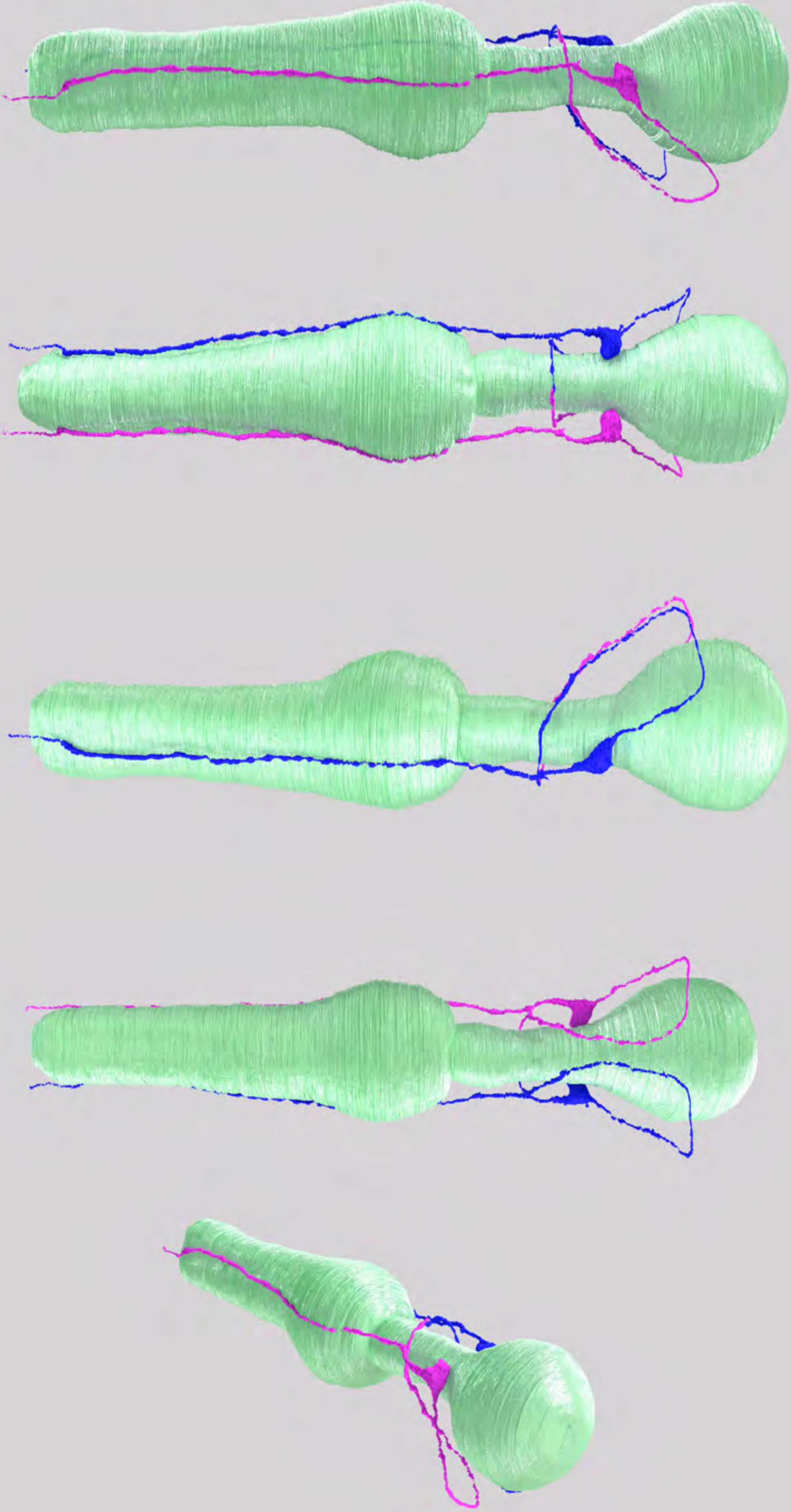

AWCL, AWCR

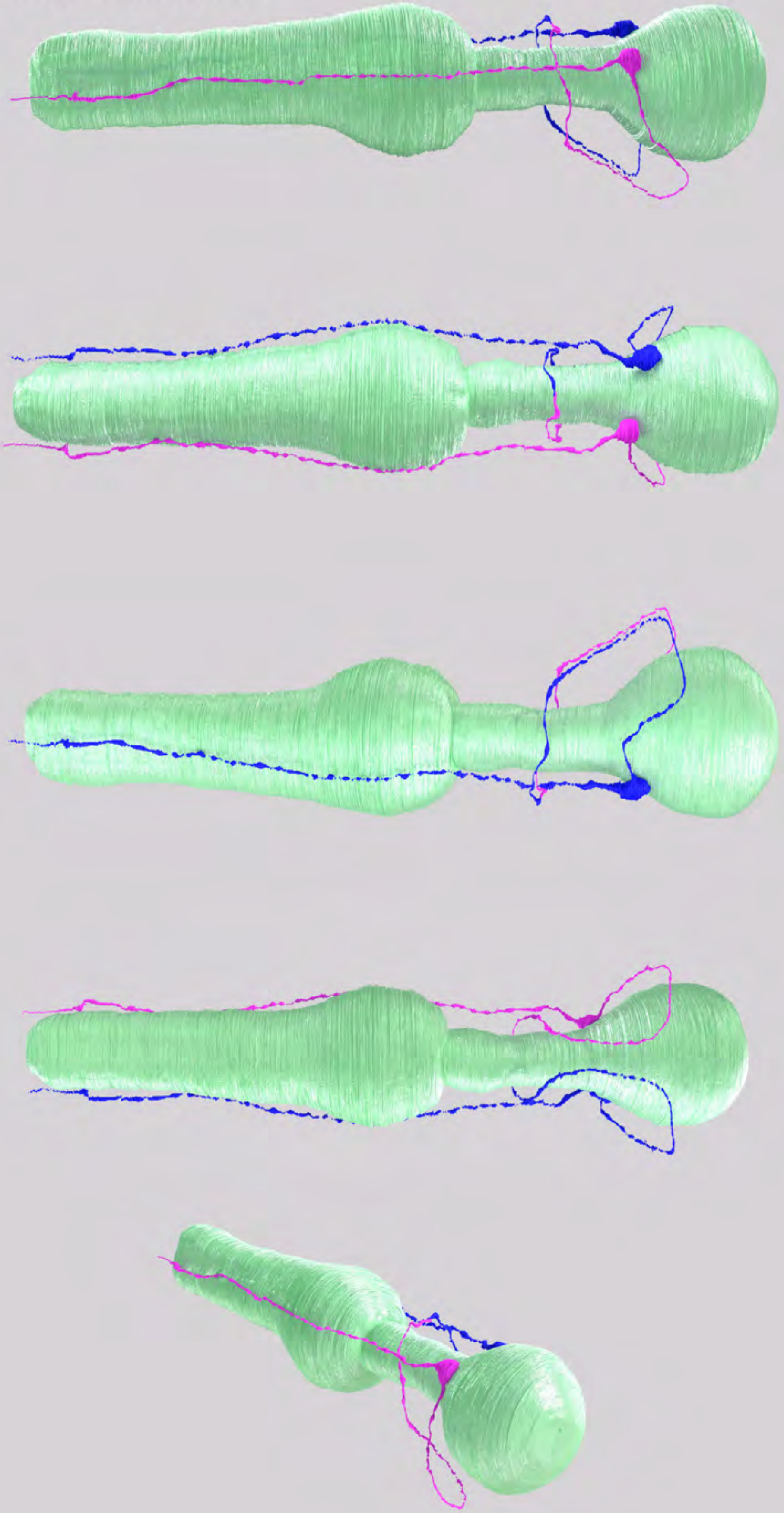

BAGL, BAGR

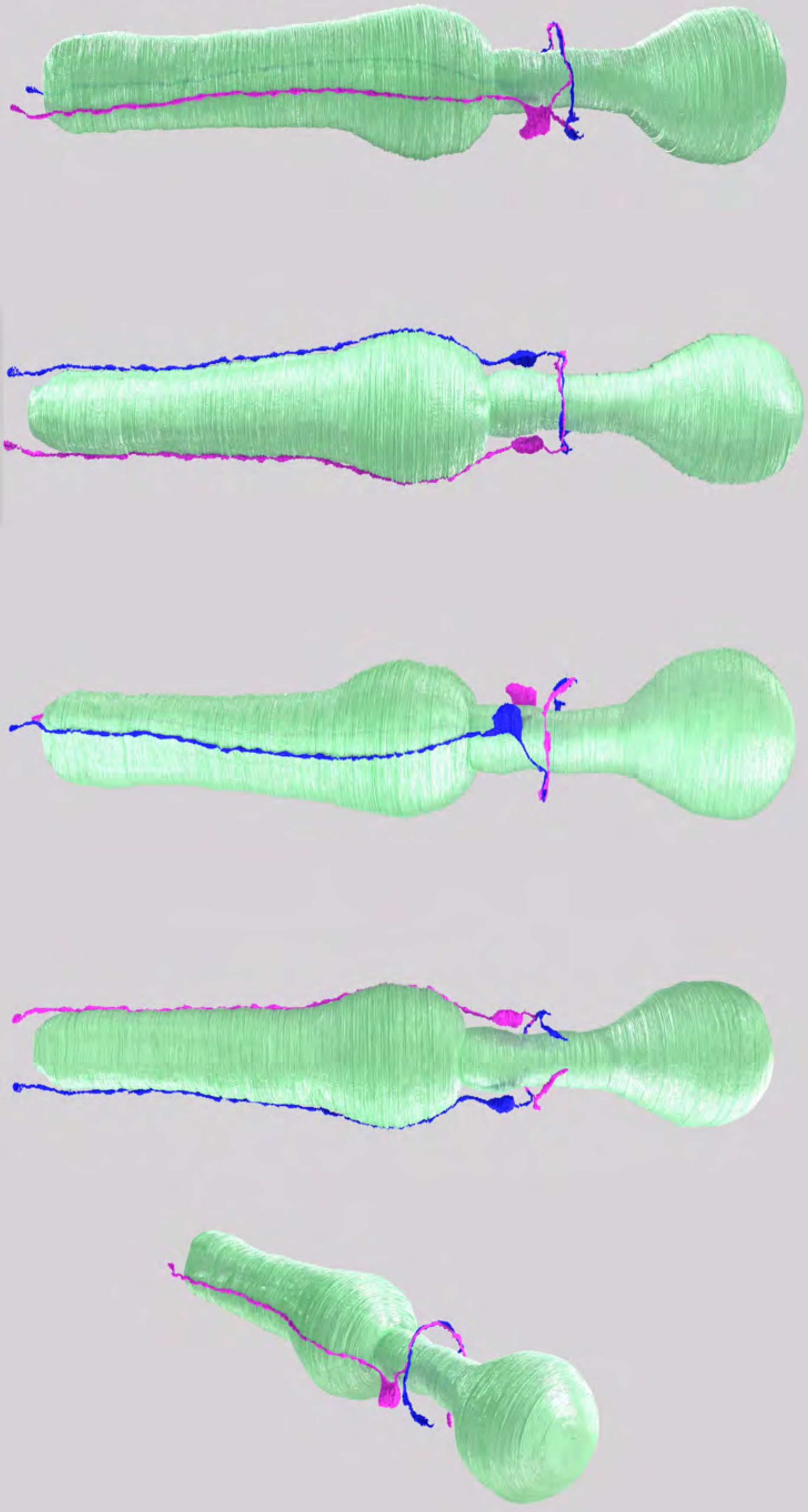

BDUL, BDUR

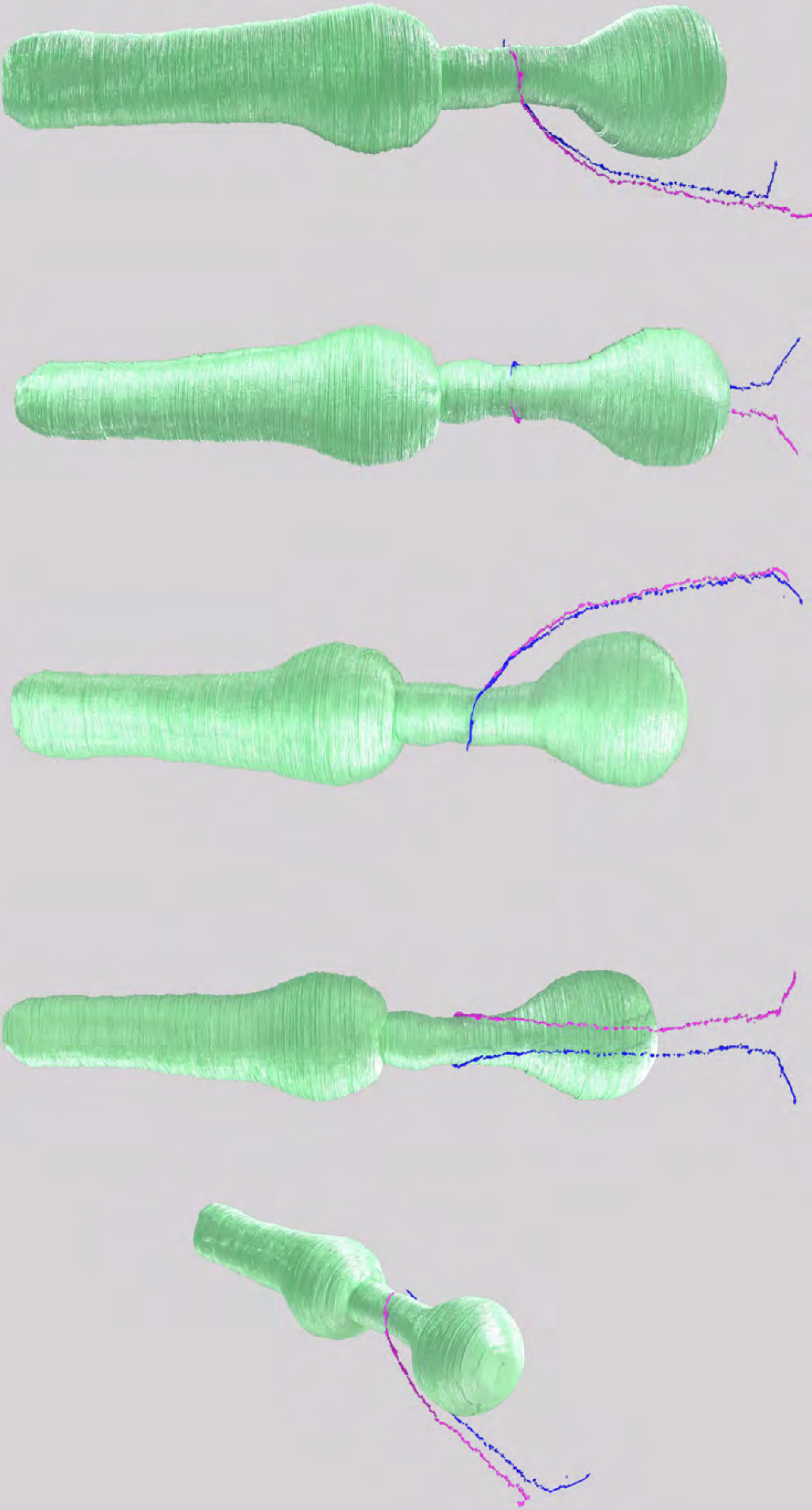

CANL, CANR

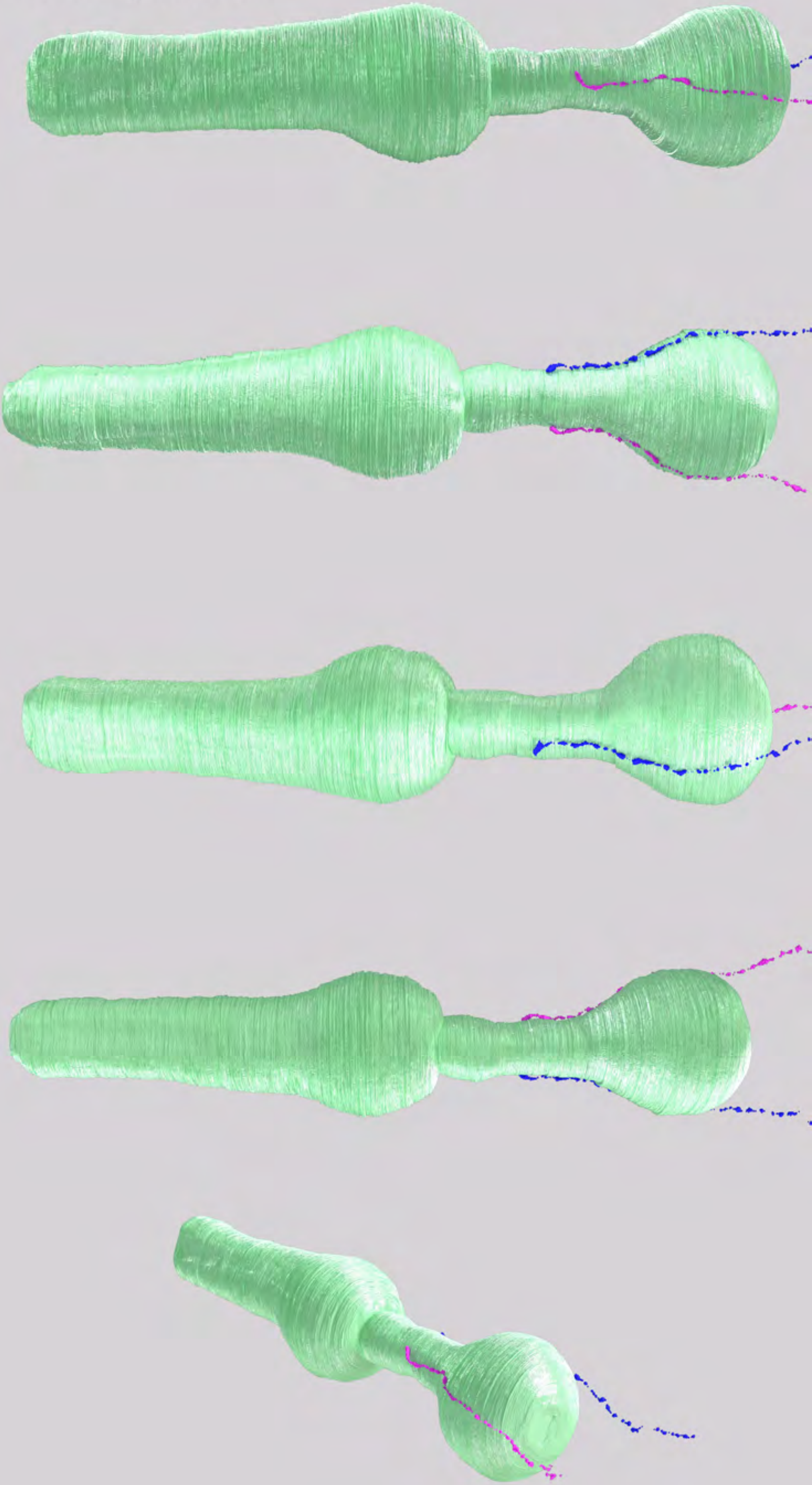

CEPDL, CEPDR

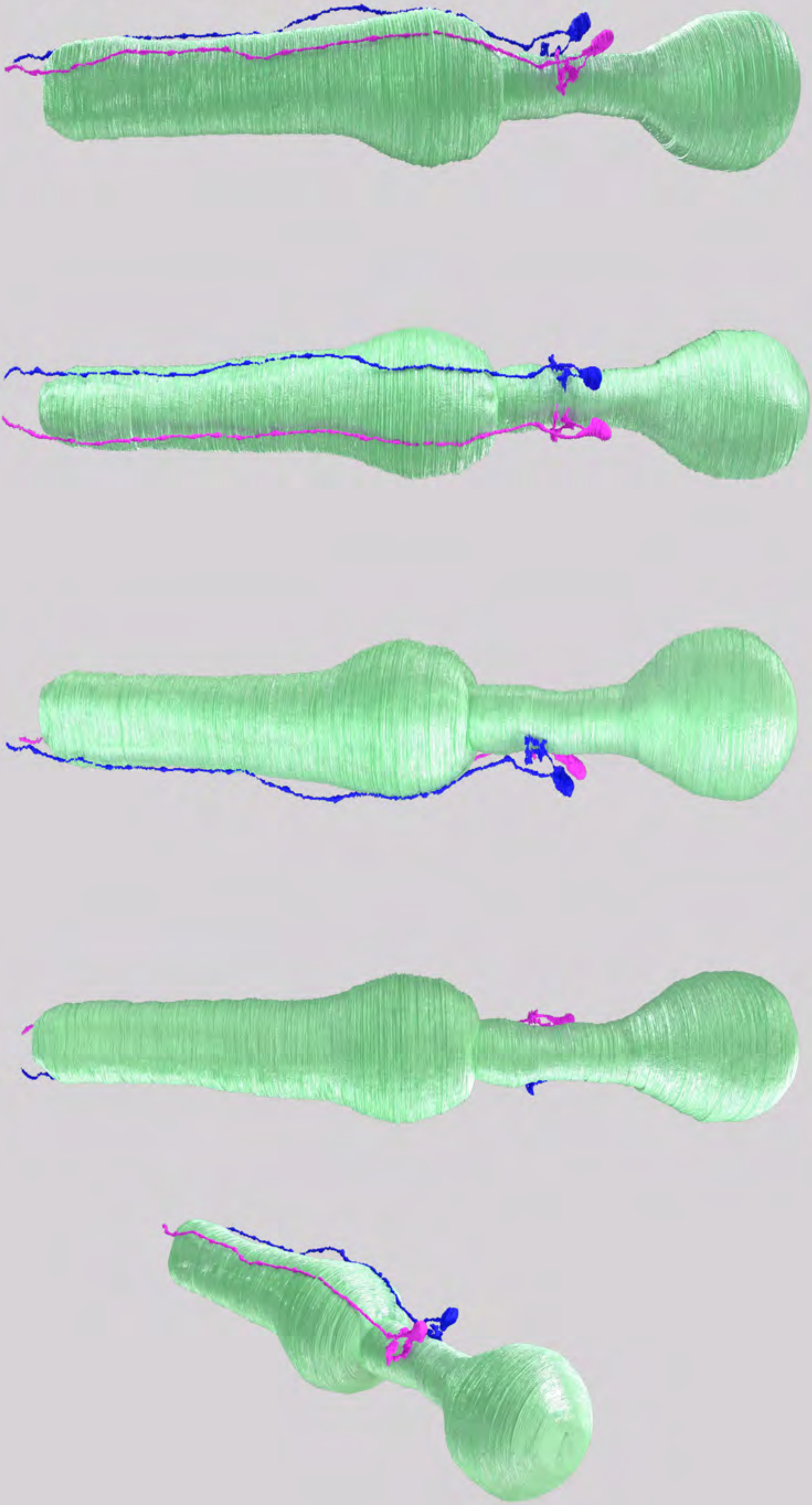

CEPVL, CEPVR

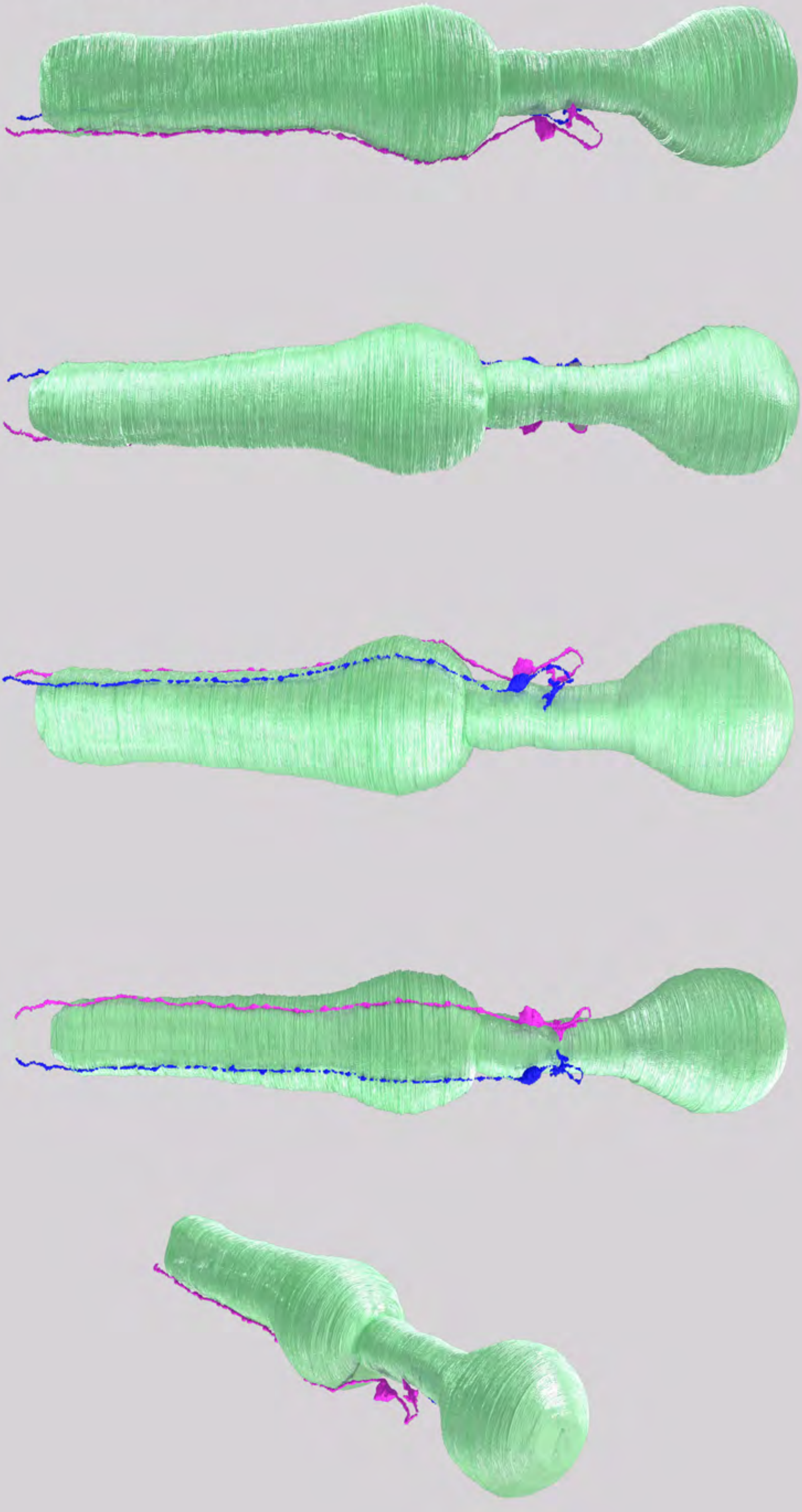

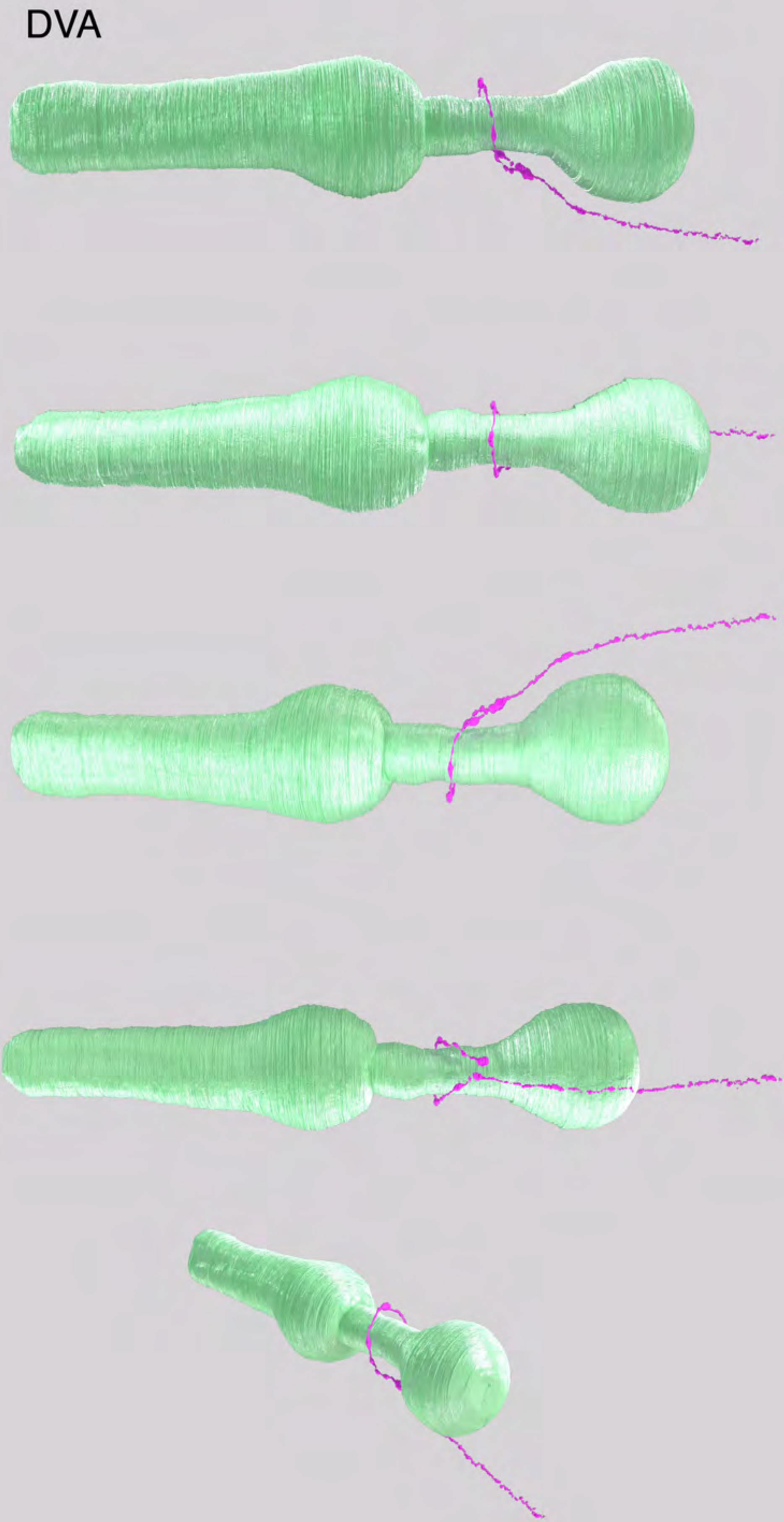
DVA

DVB

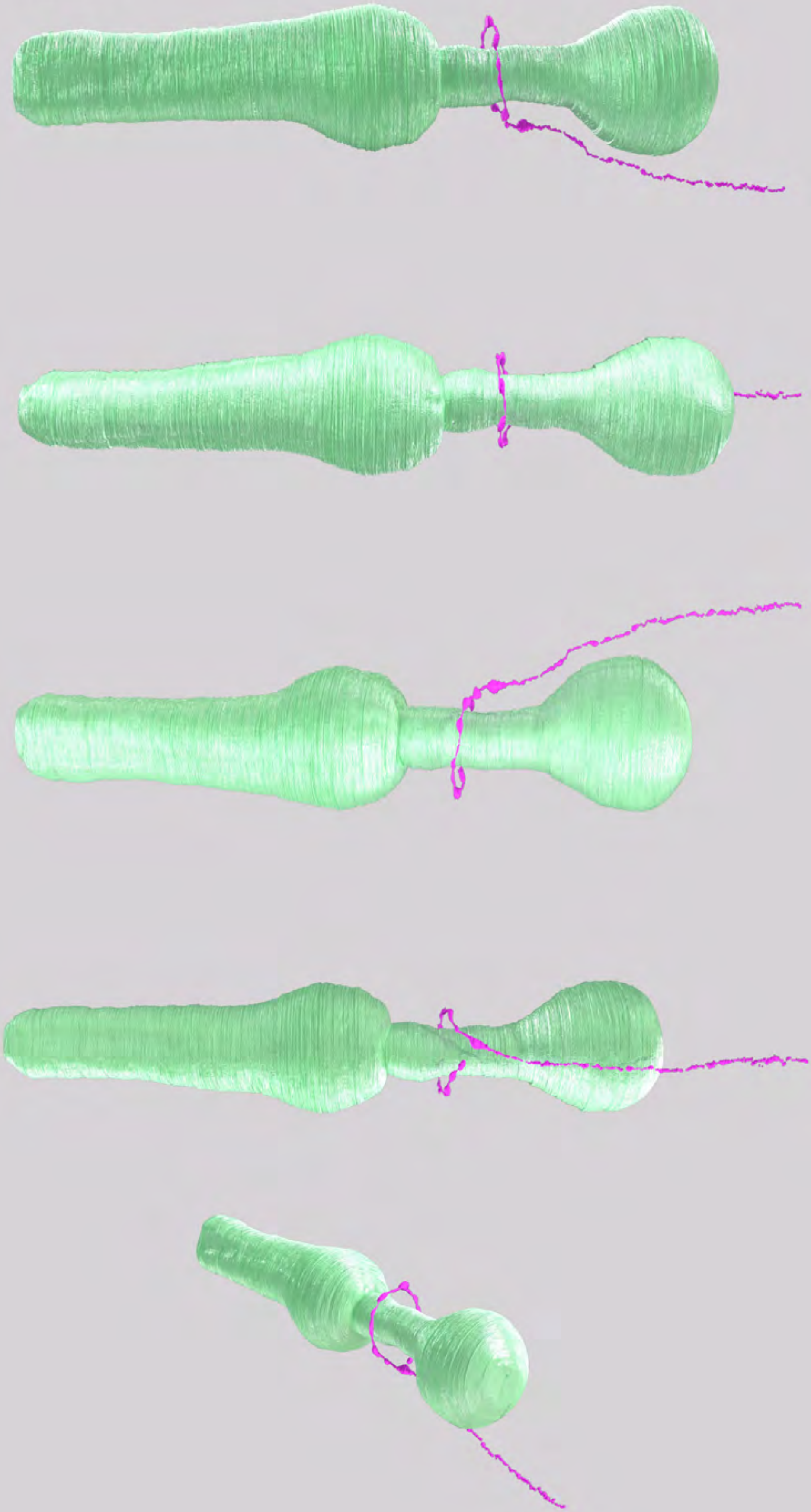

DVC

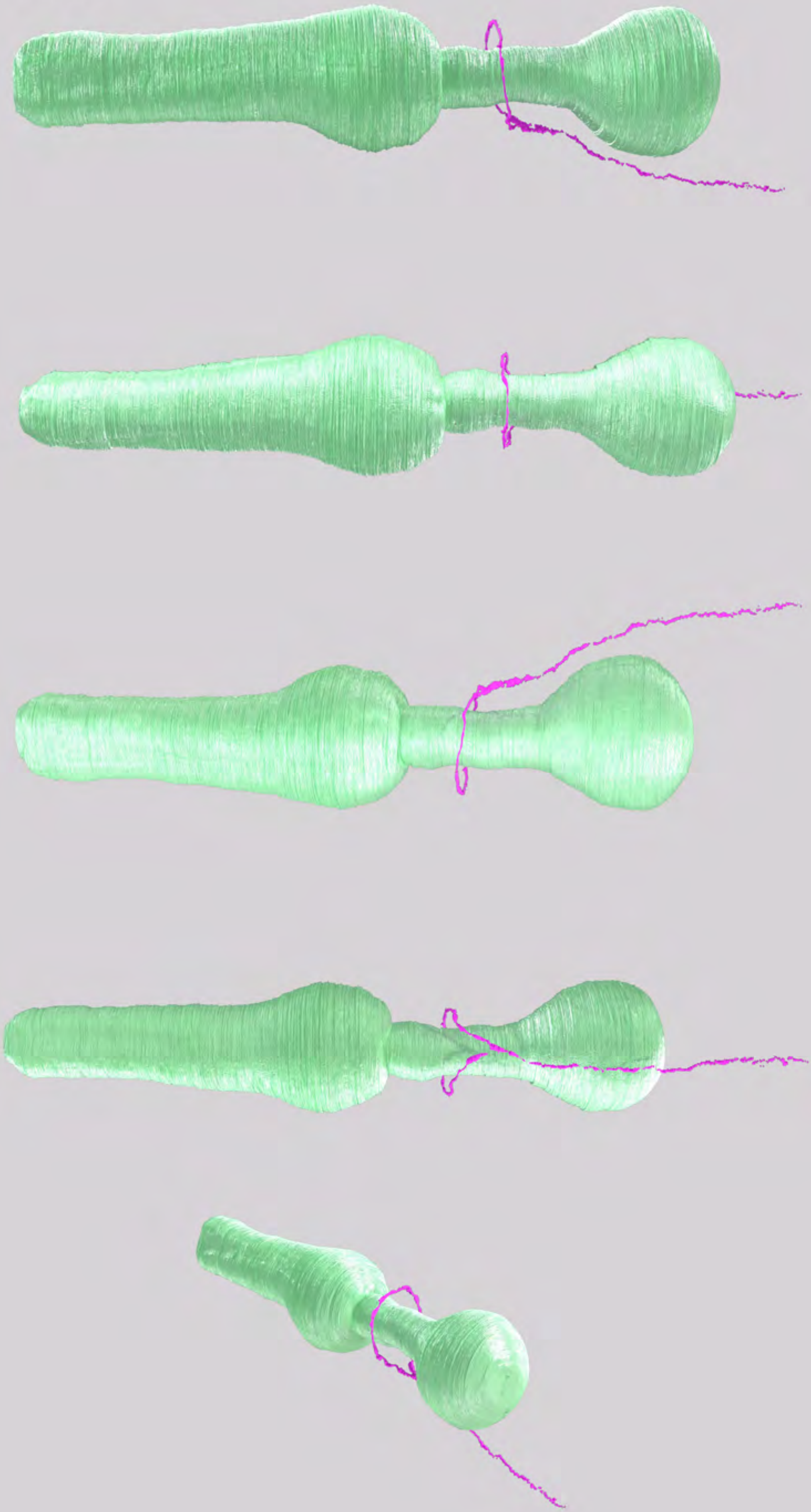

FLPL, FLPR

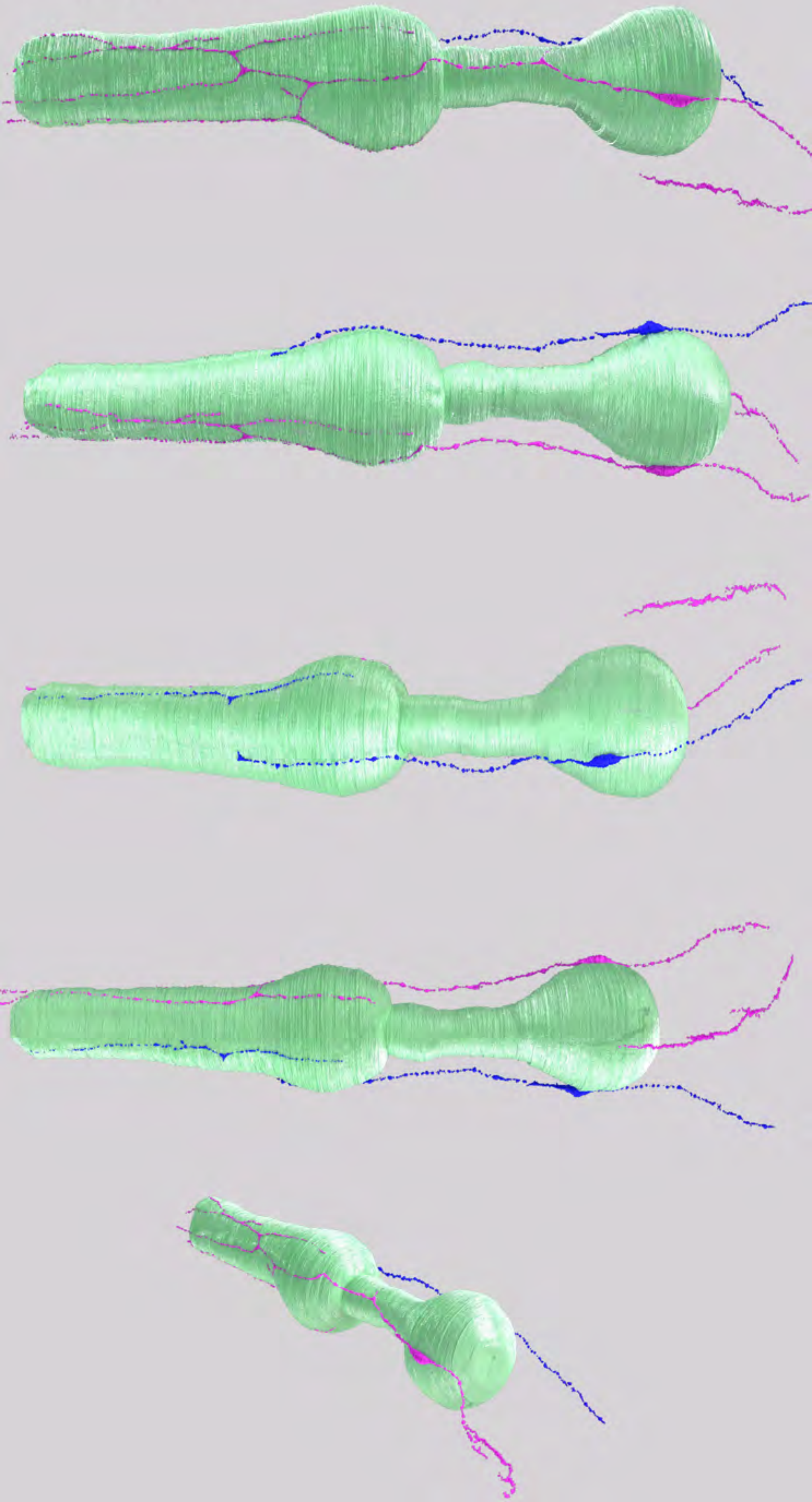

IL1DL, IL1DR

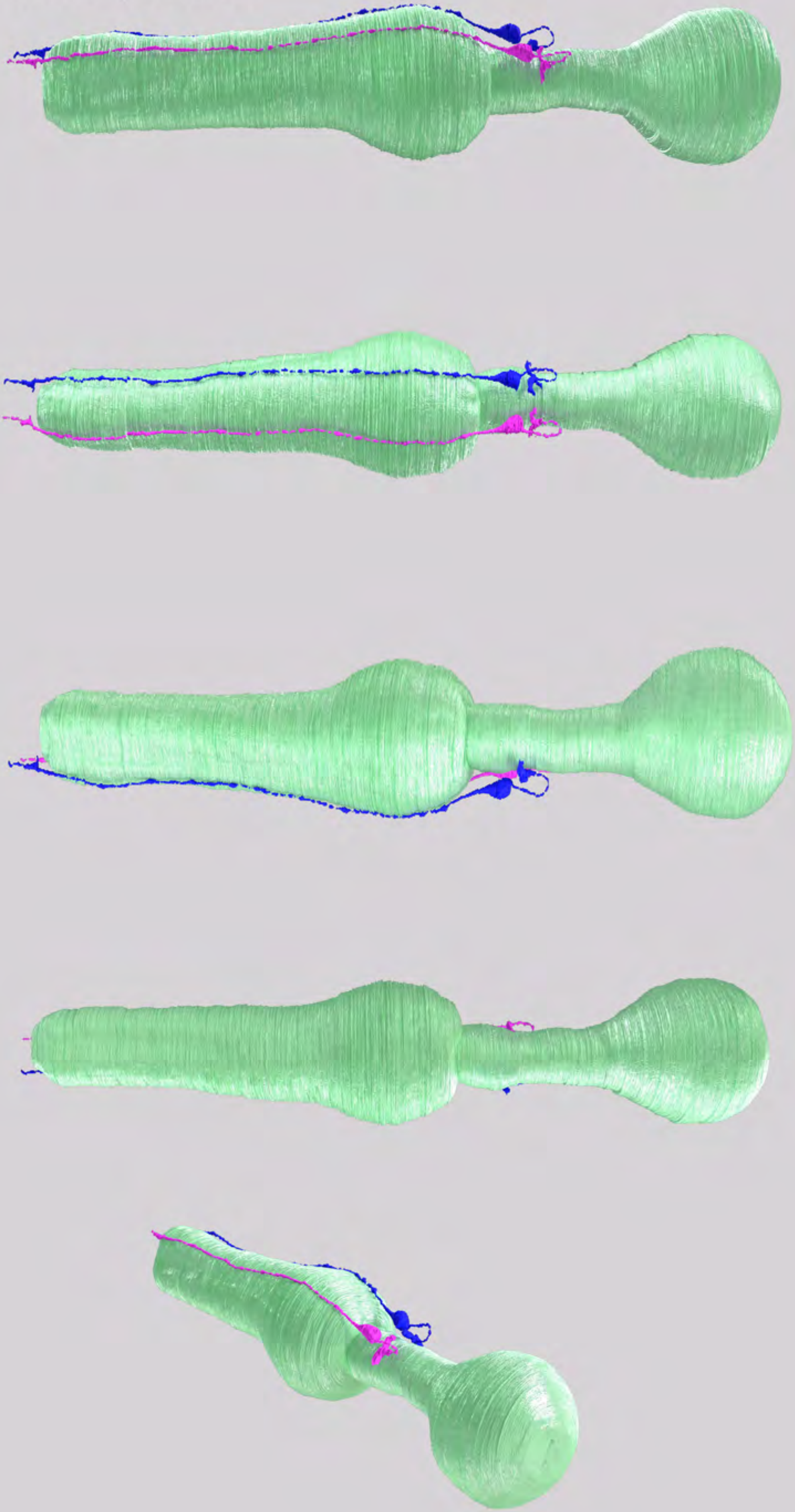

IL1L, IL1R

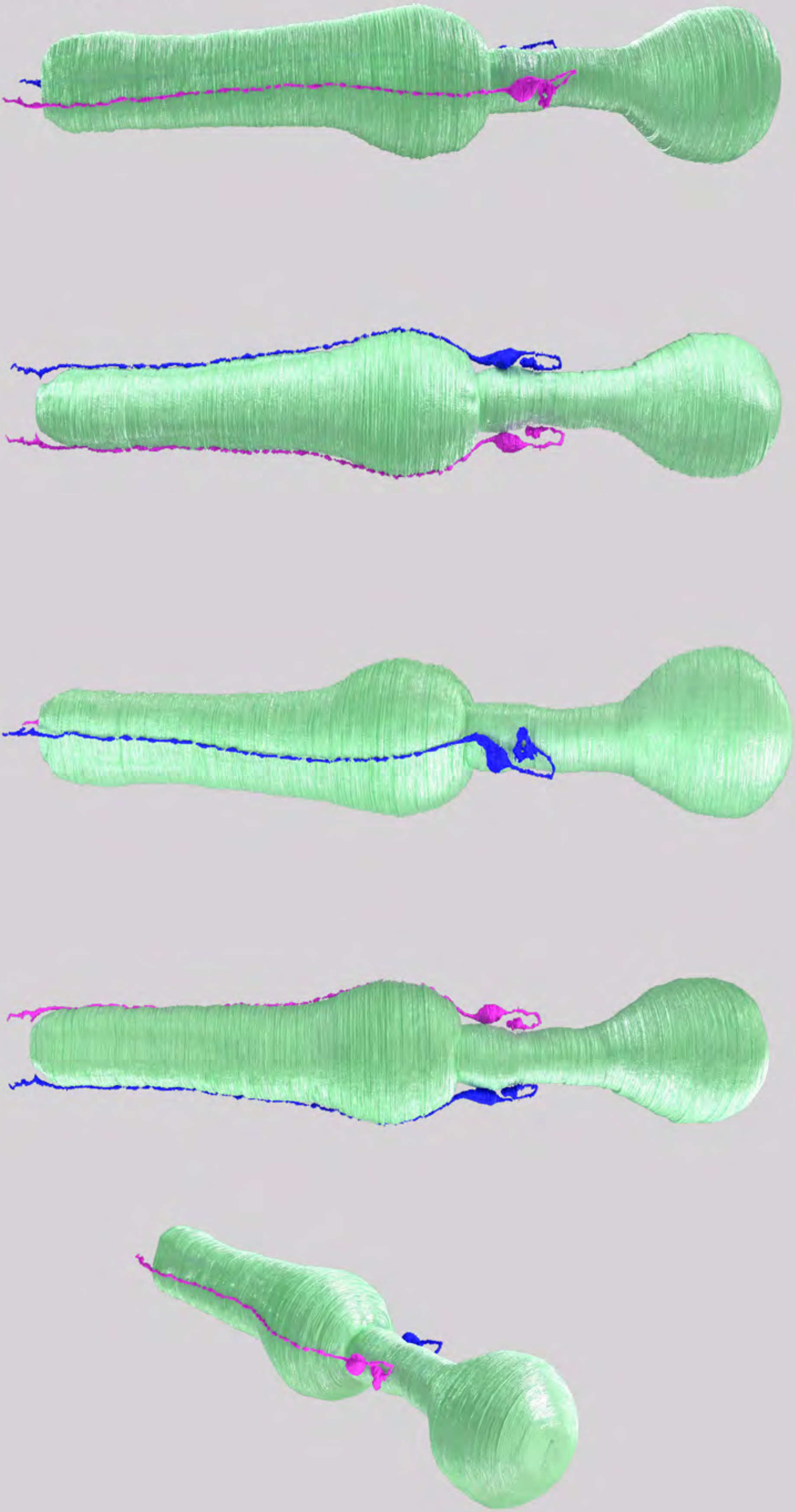

IL1VL, IL1VR

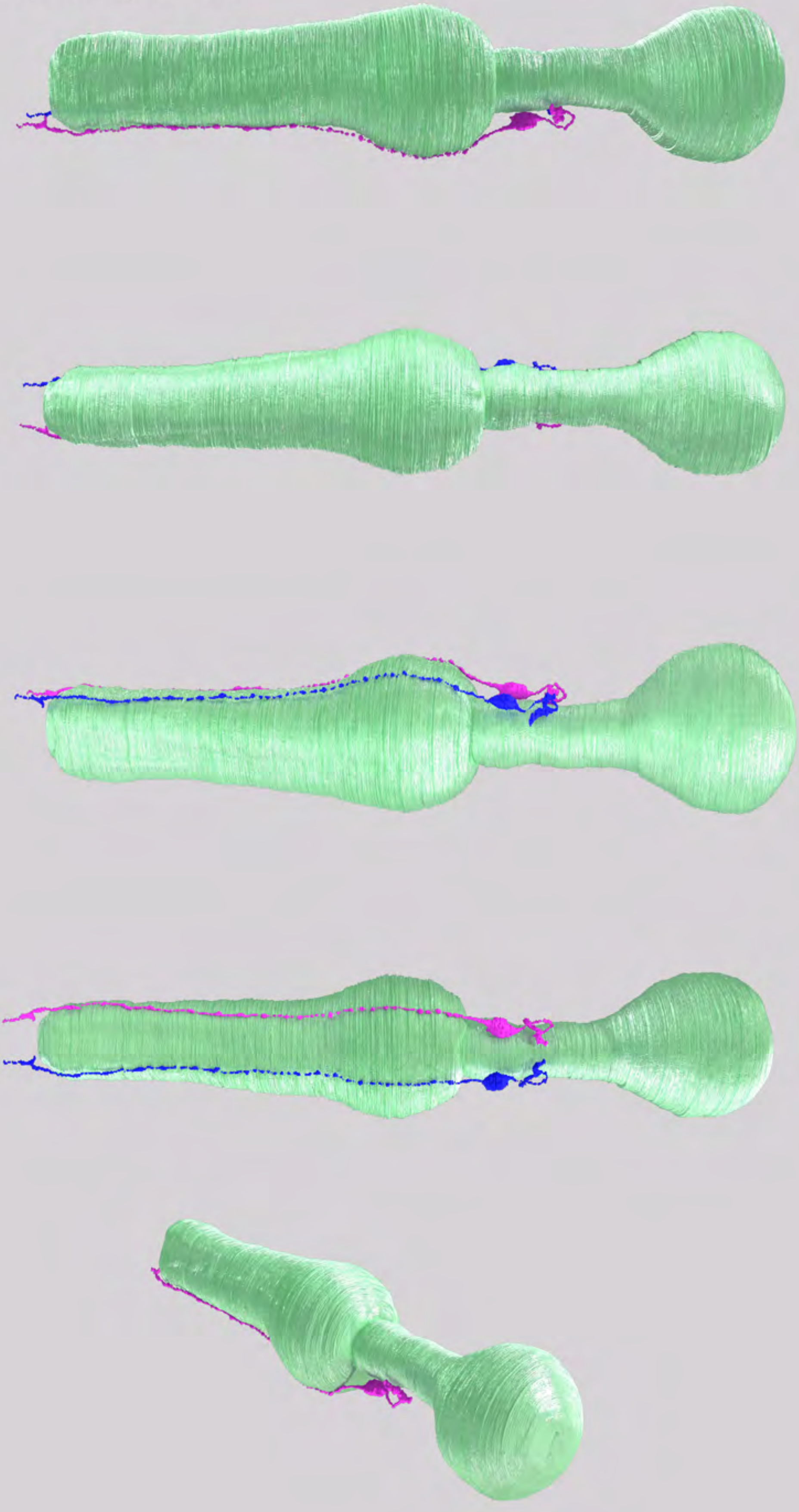

IL2DL, IL2DR

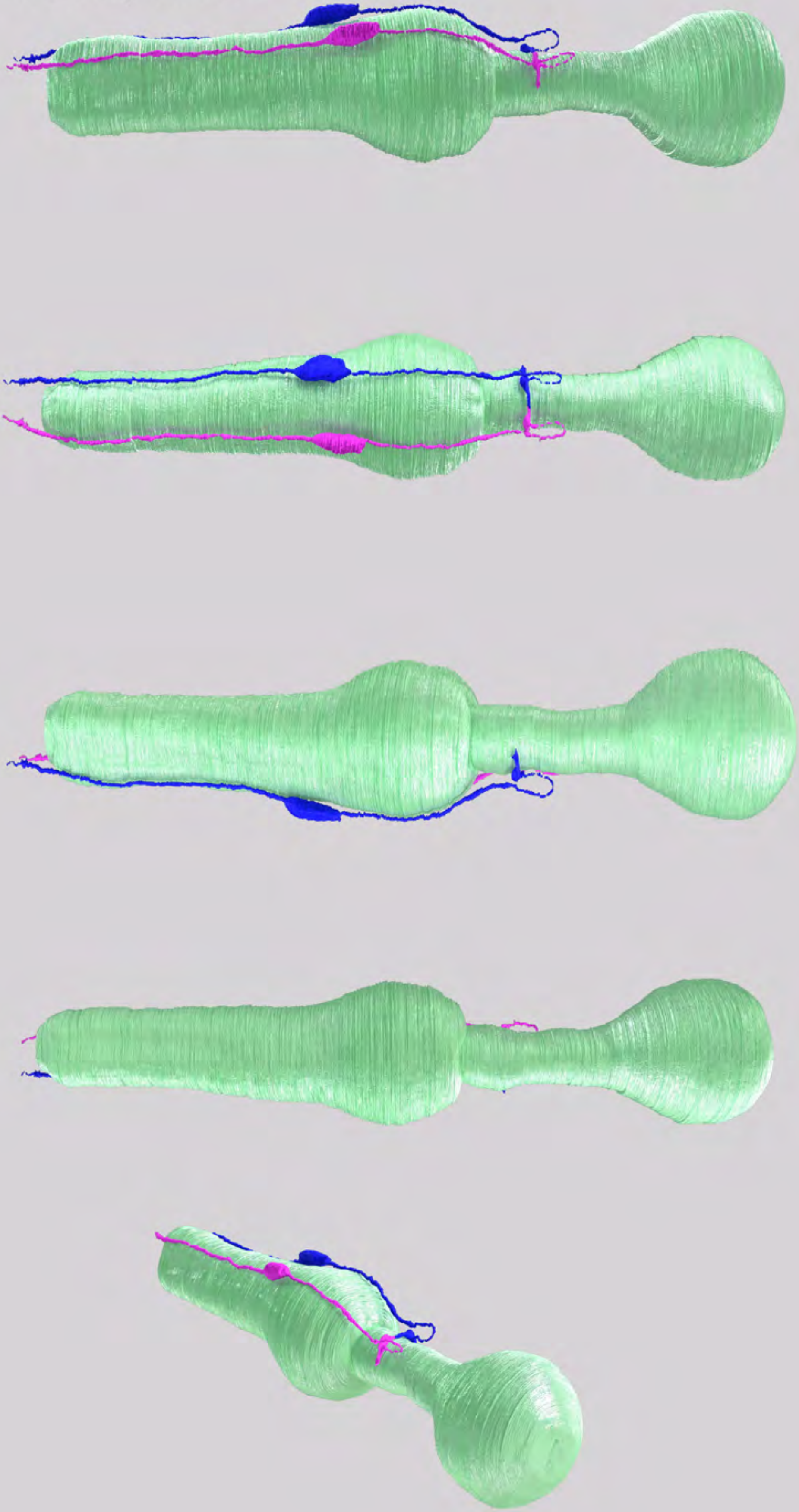

IL2L, IL2R

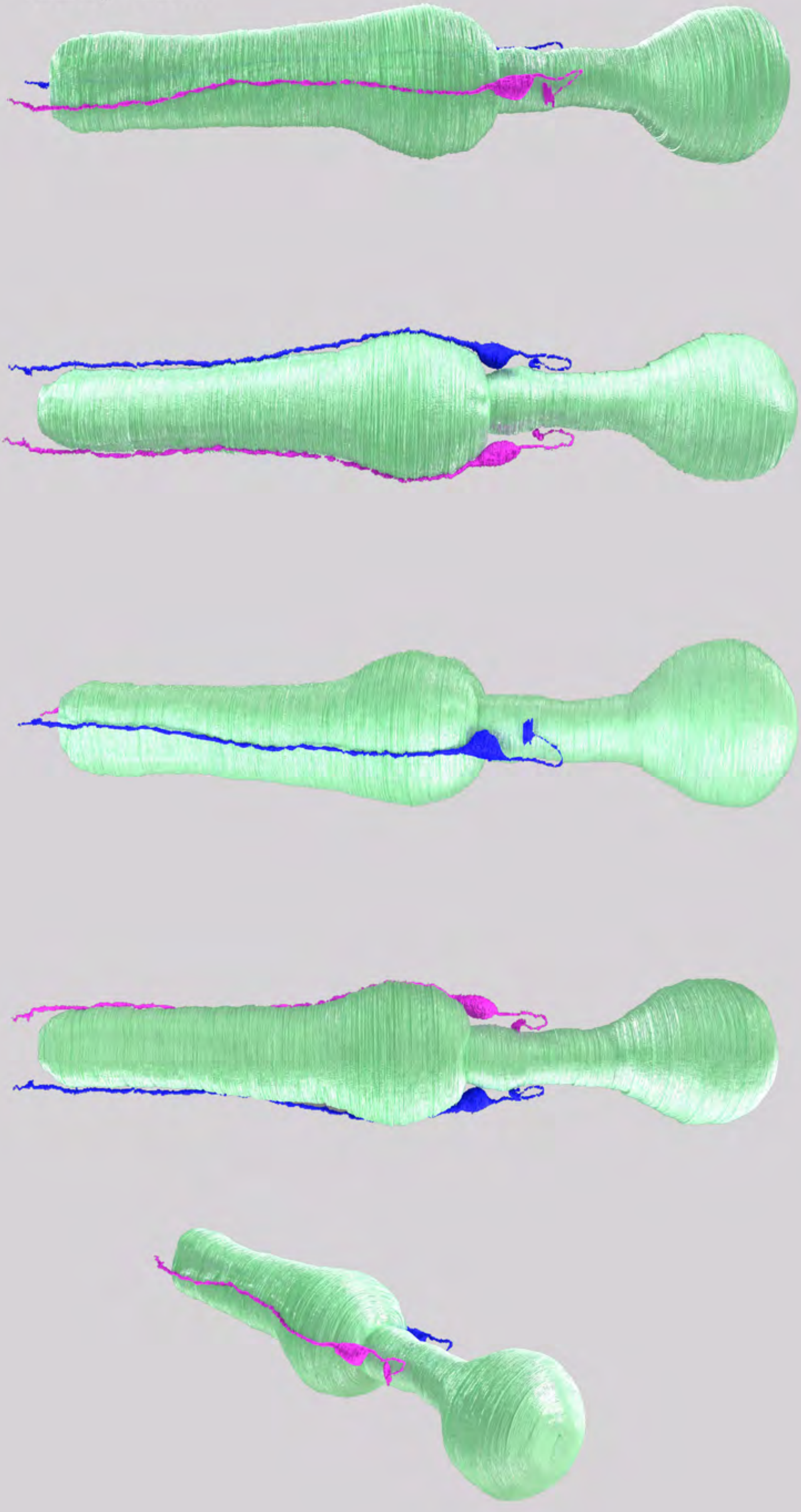

IL2VL, IL2VR

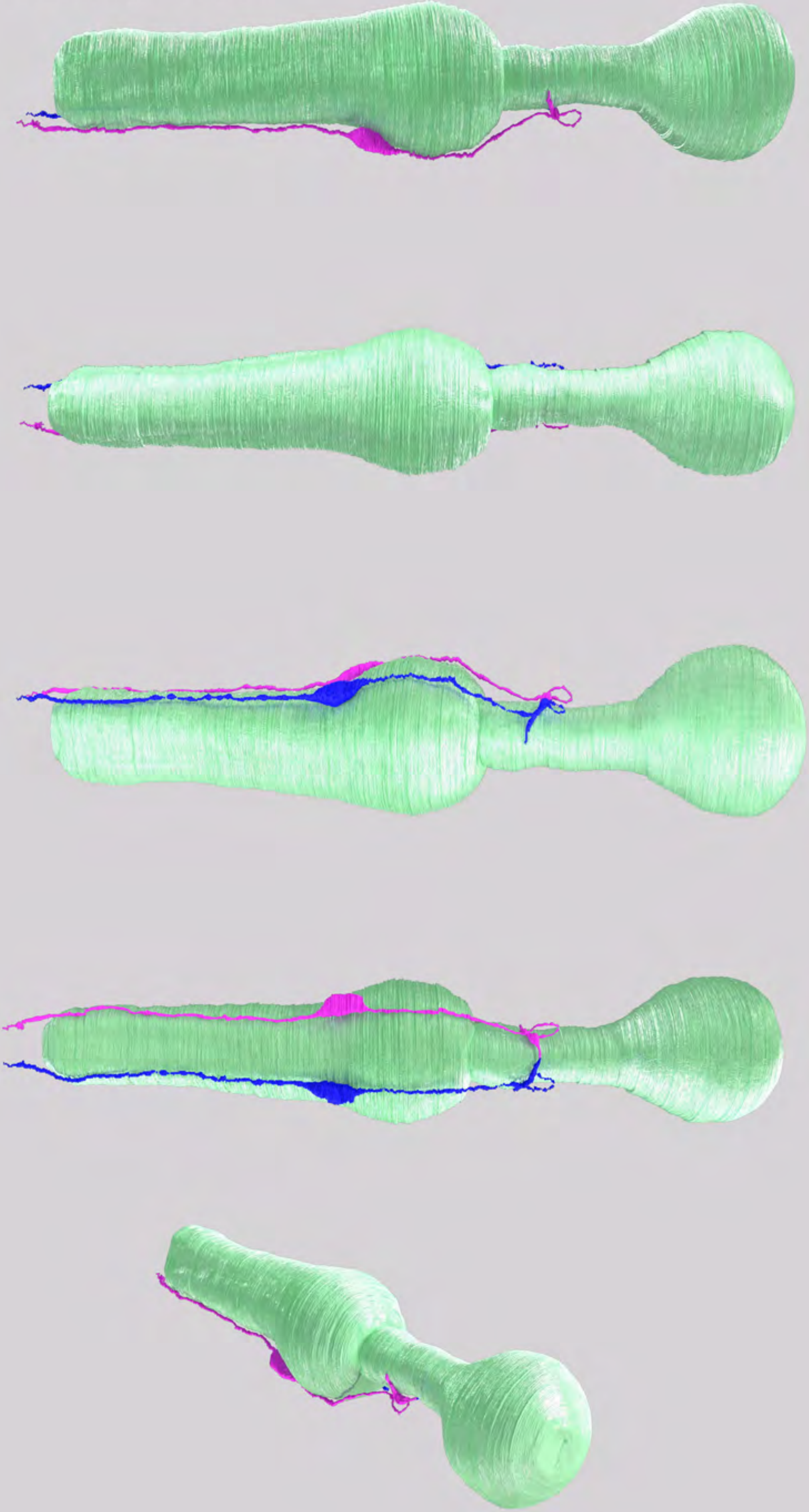

OLLL, OLLR

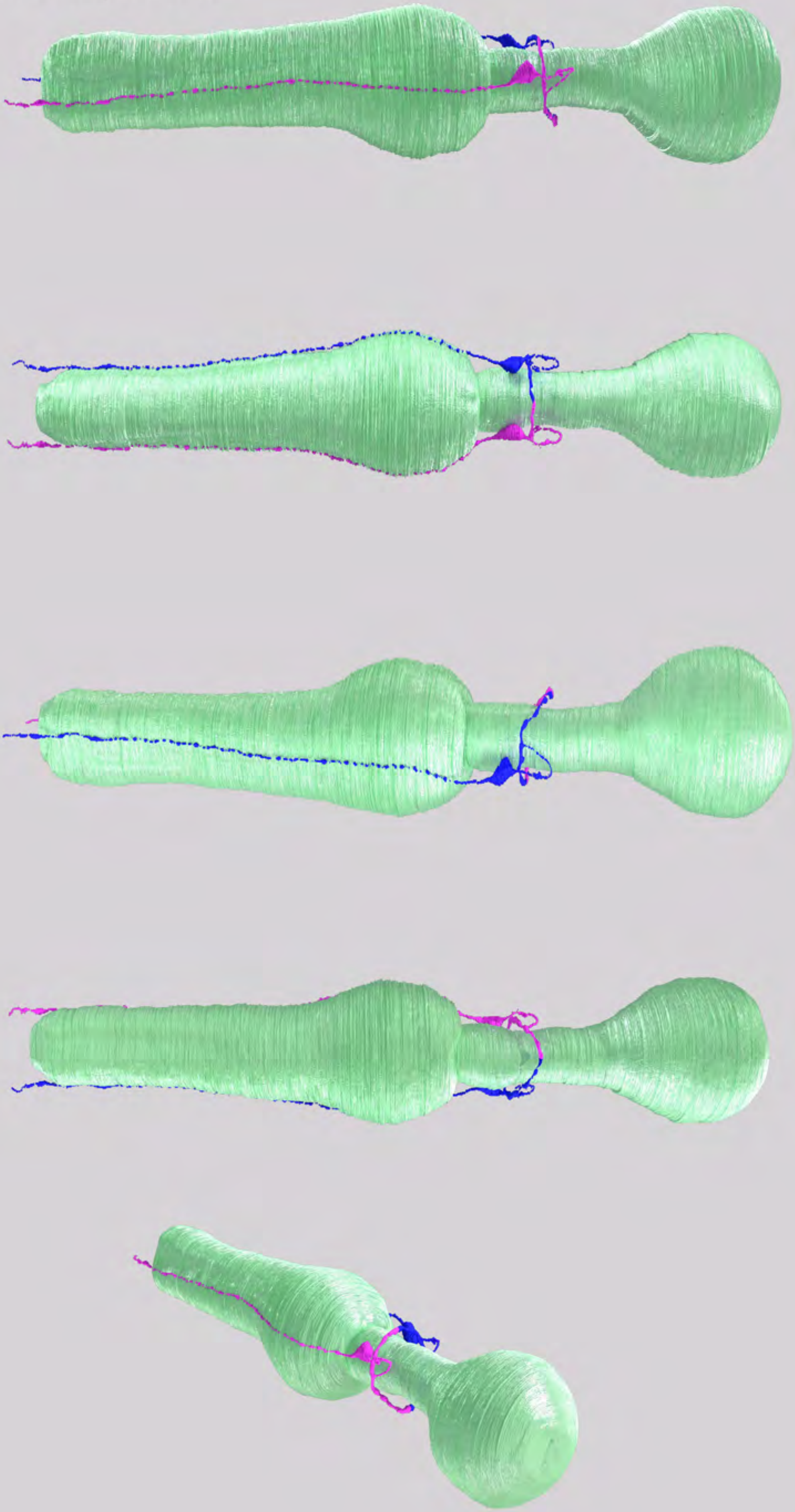

OLQDL, OLQDR

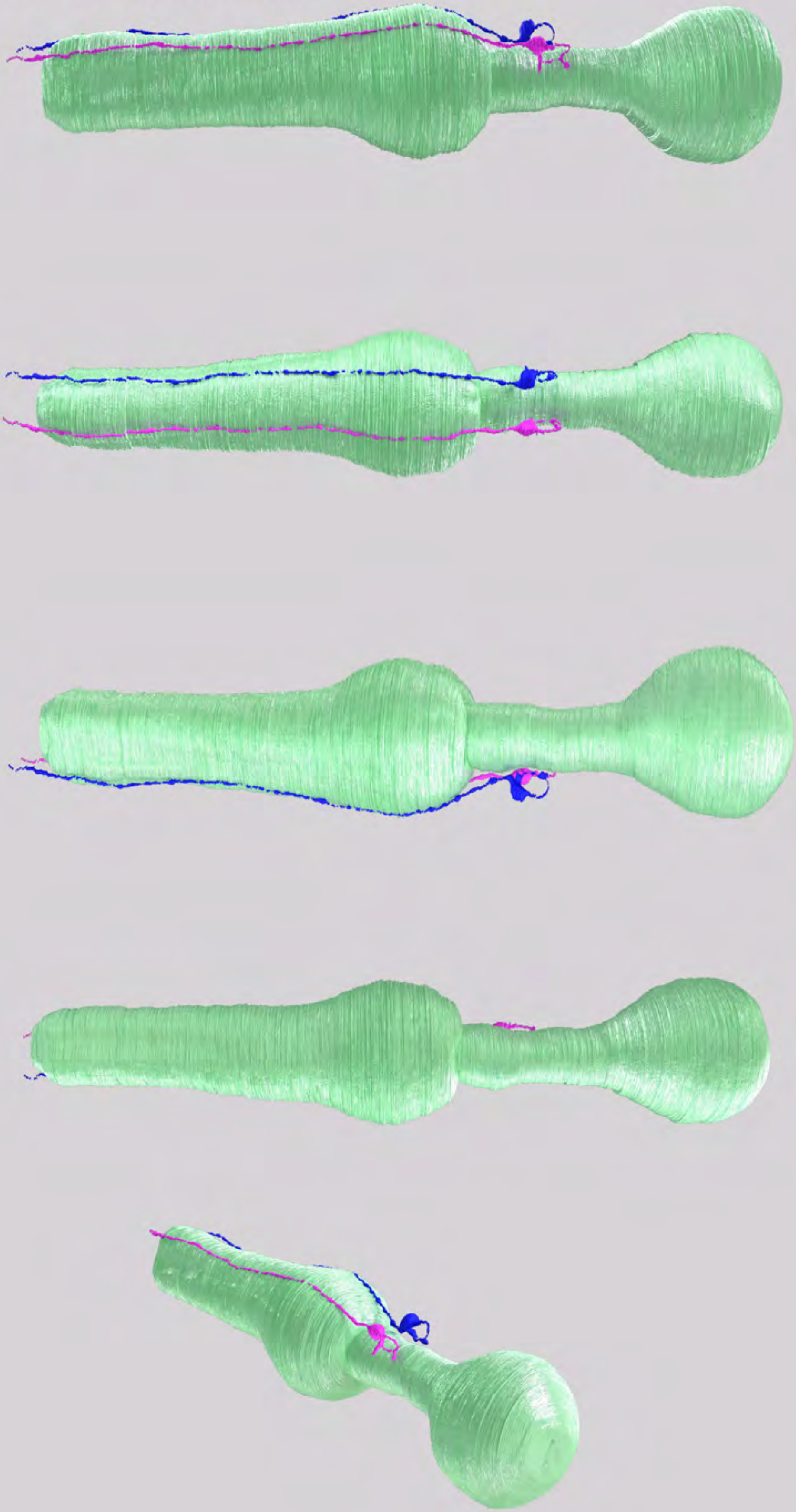

OLQVL, OLQVR

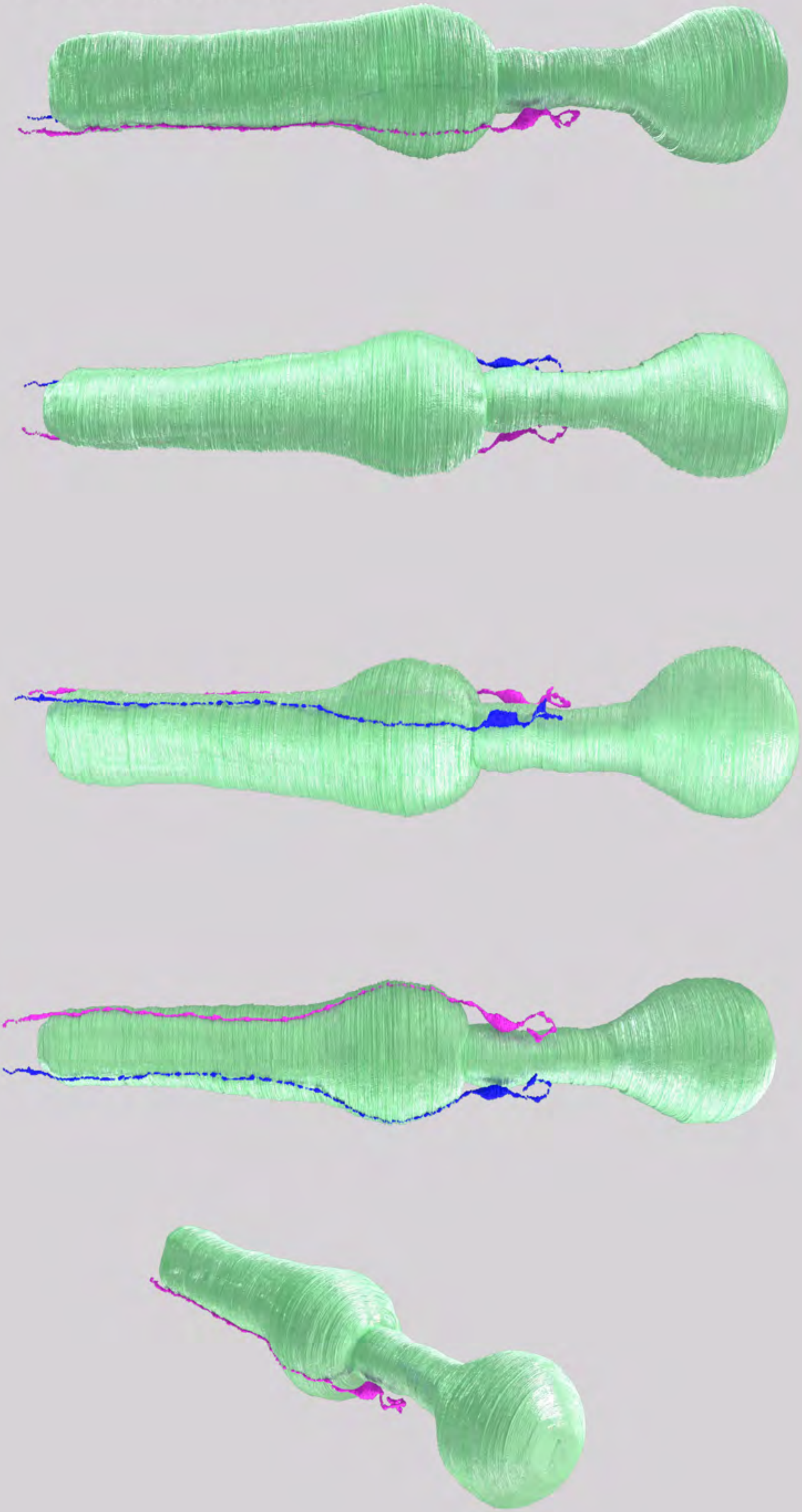

PLNL, PLNR

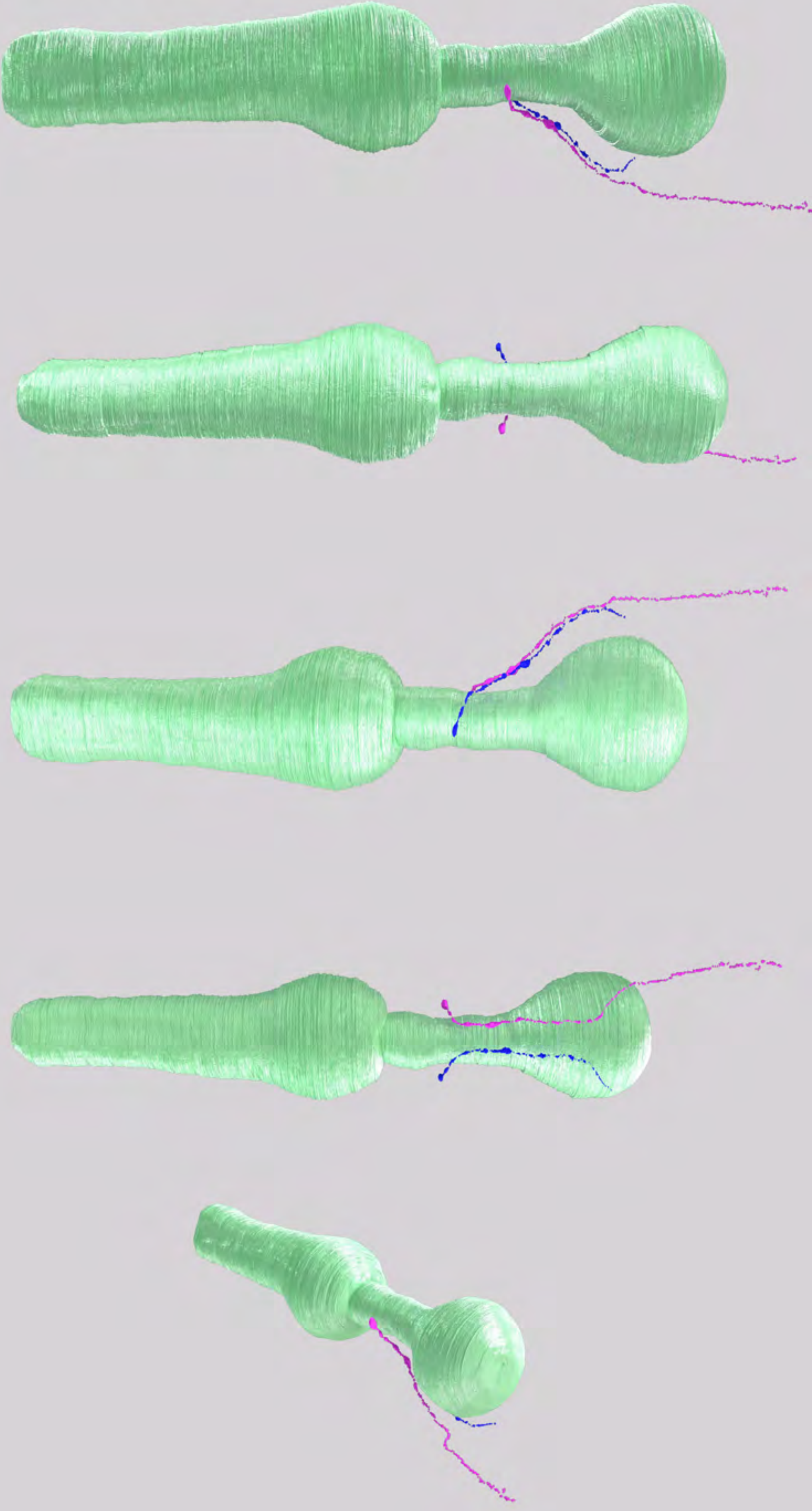

PVCL, PVCR

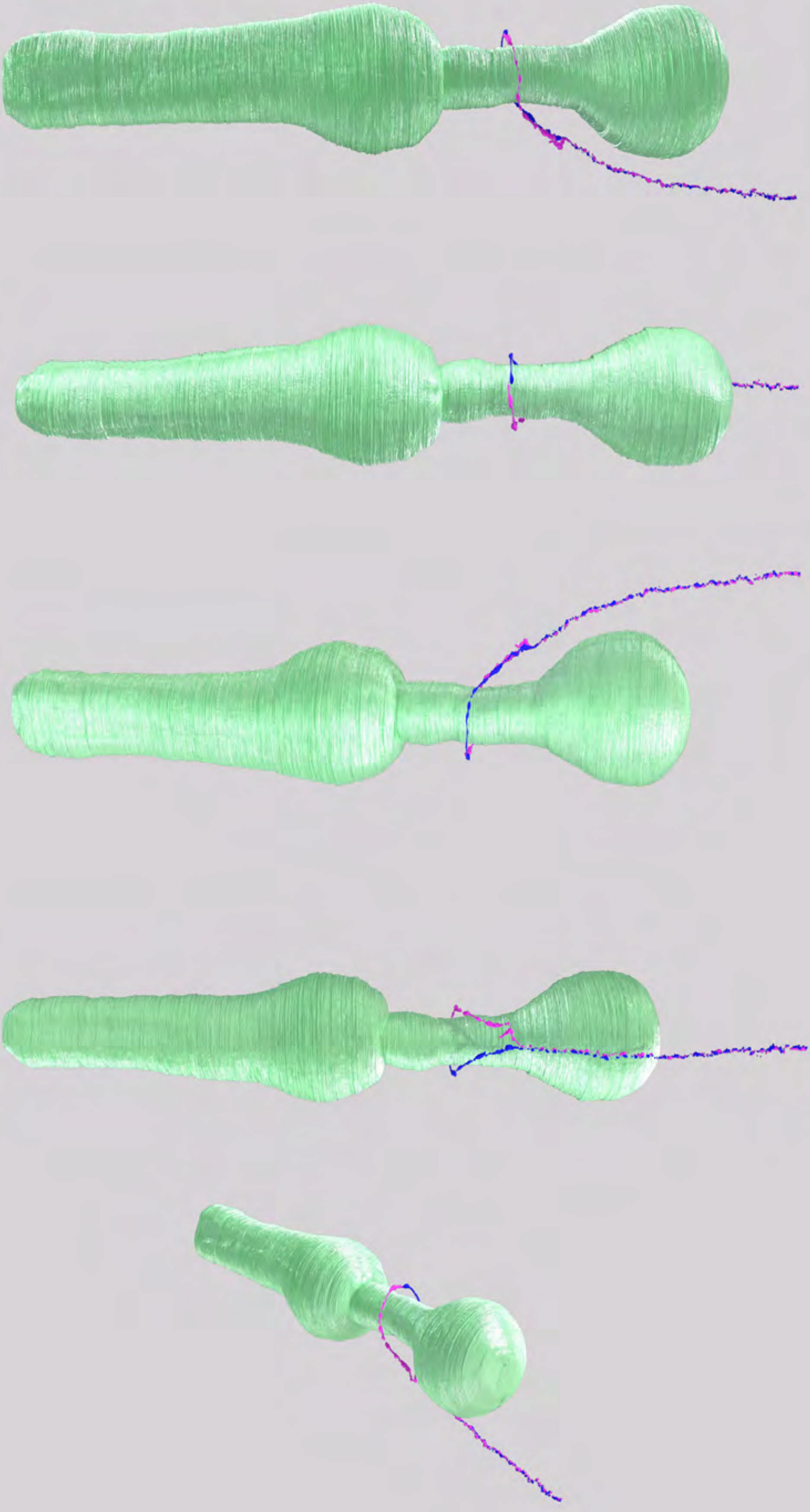

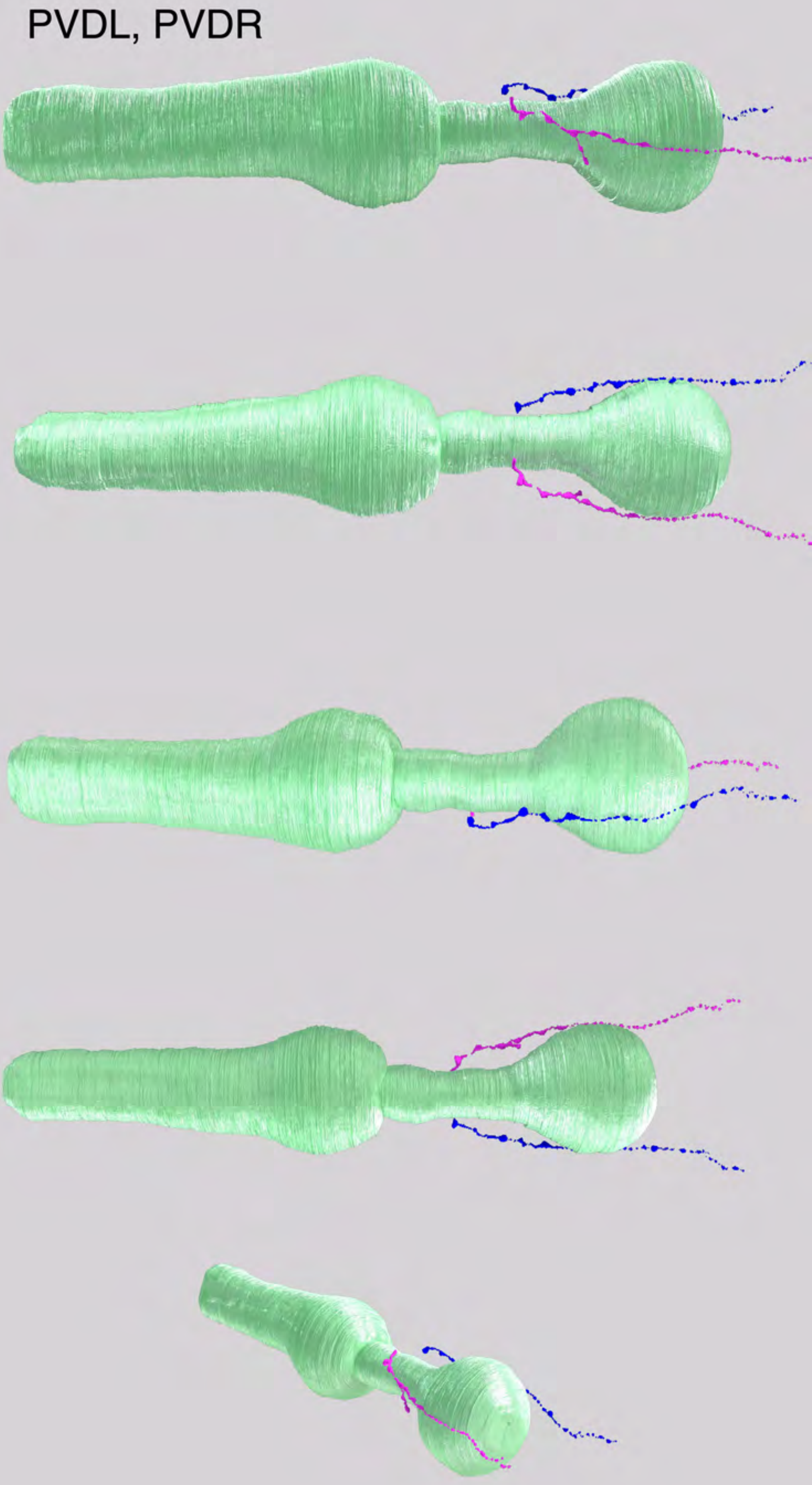
PVDL, PVDR

PVNL, PVNR

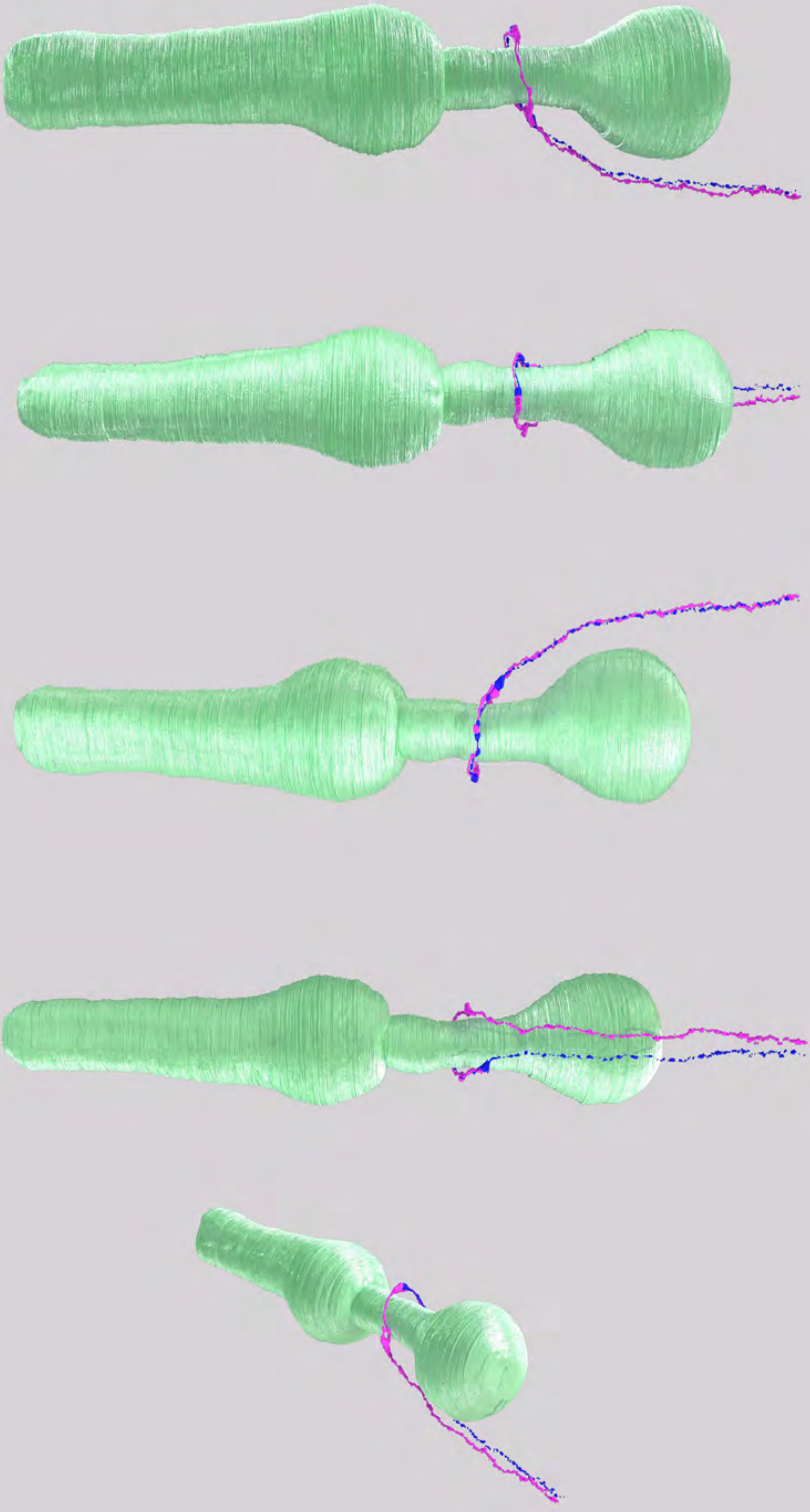

PVPL, PVPR

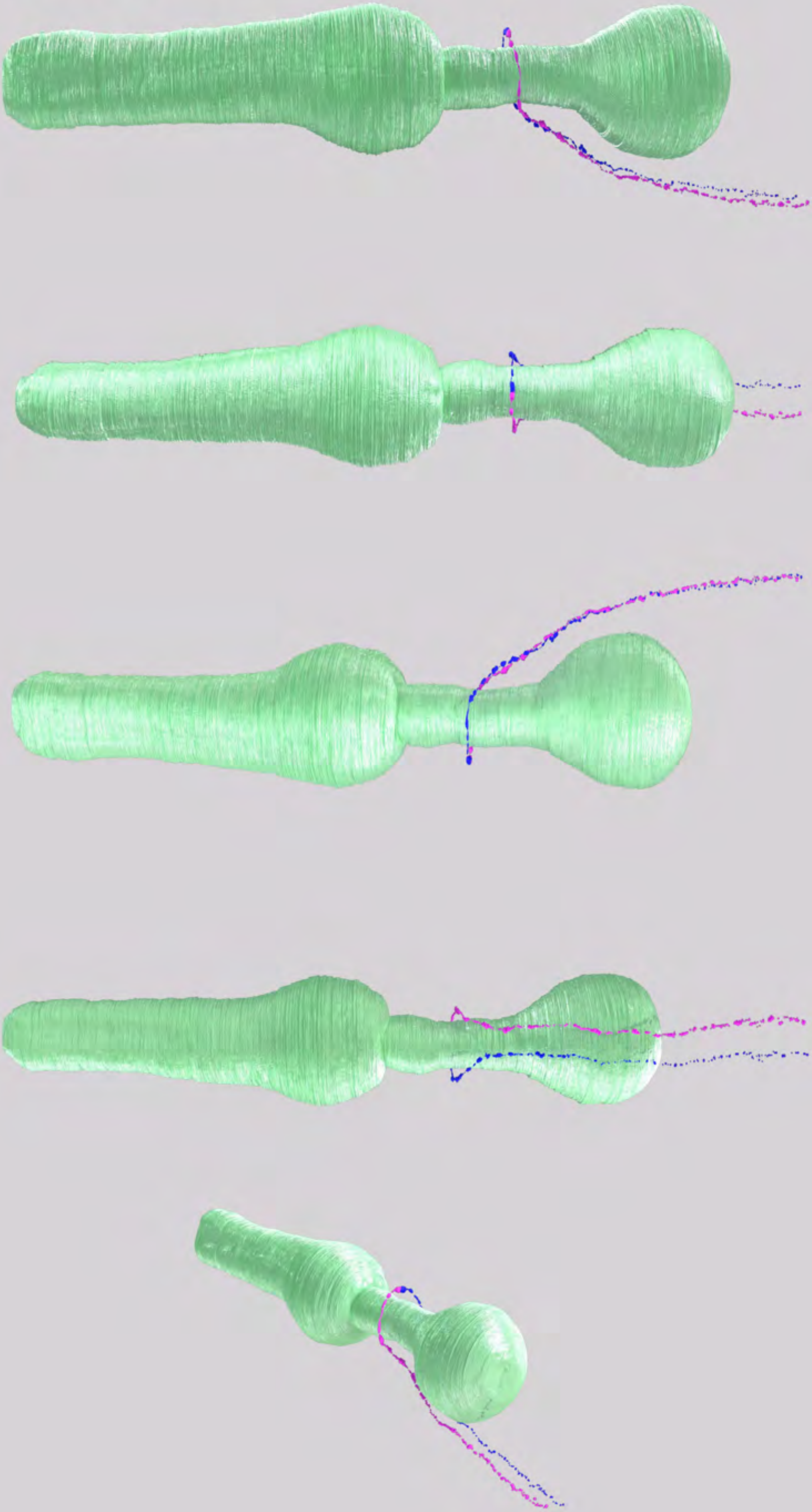

PVQ

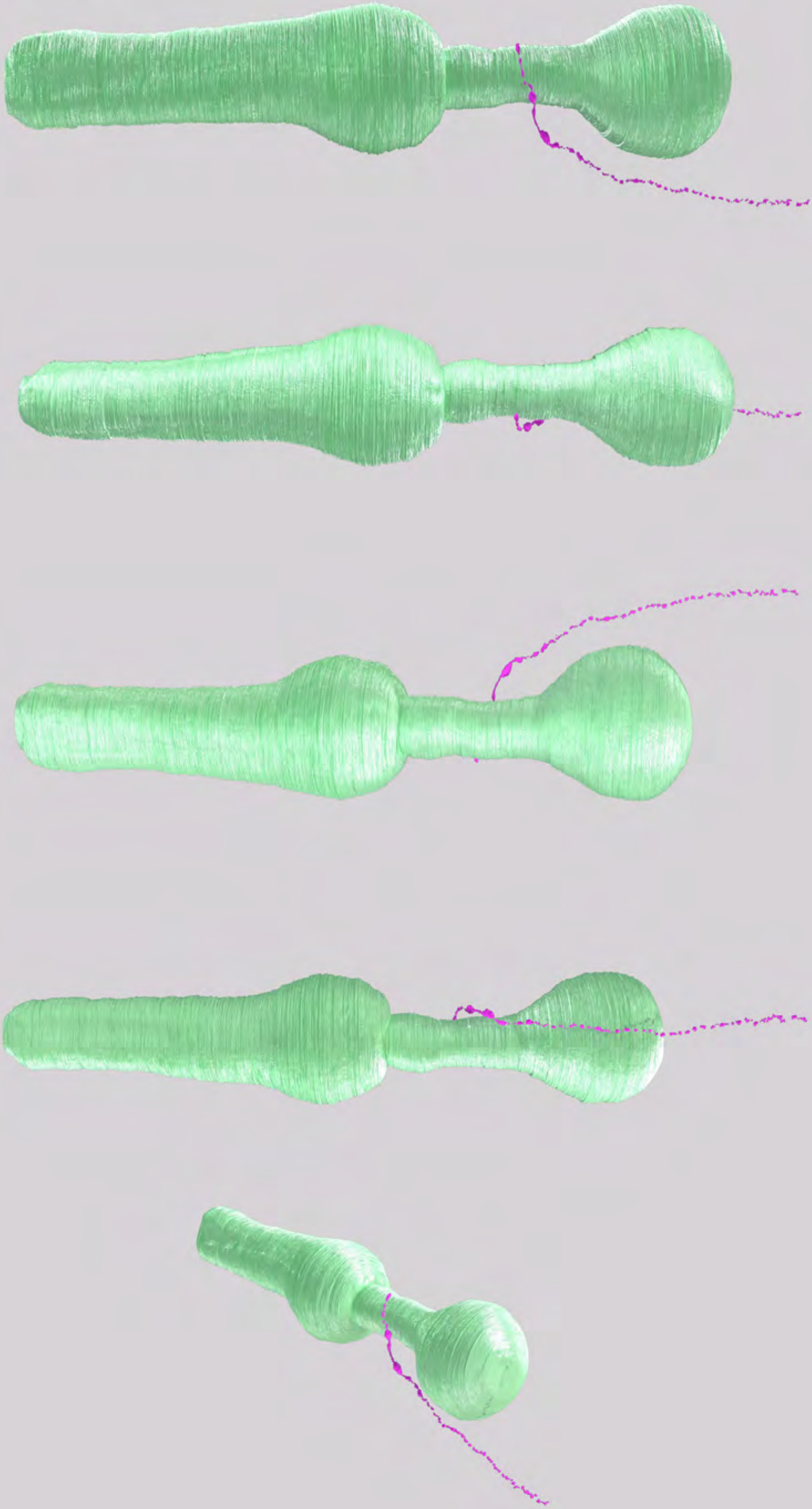

PVR

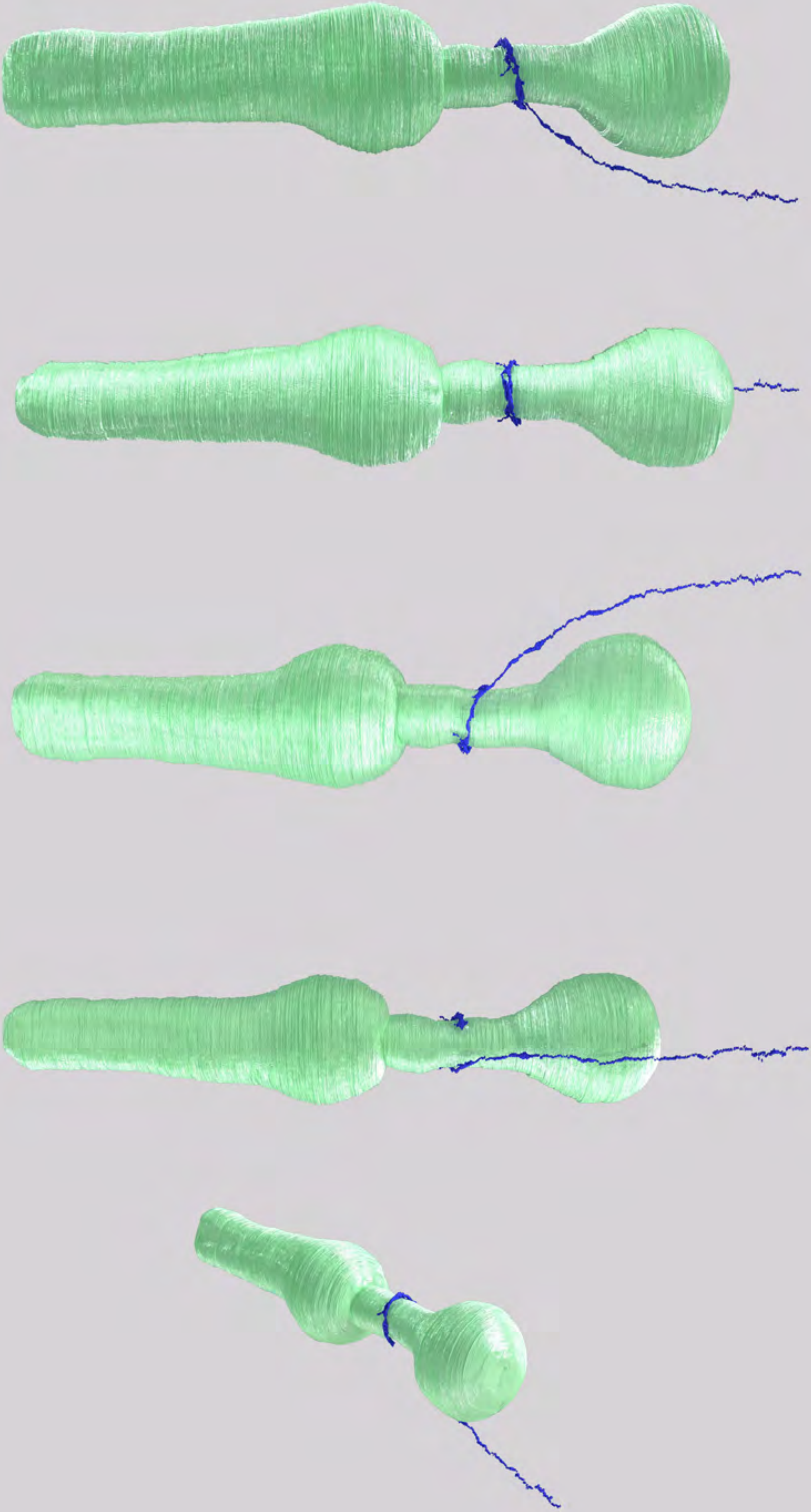

RIAL, RIAR

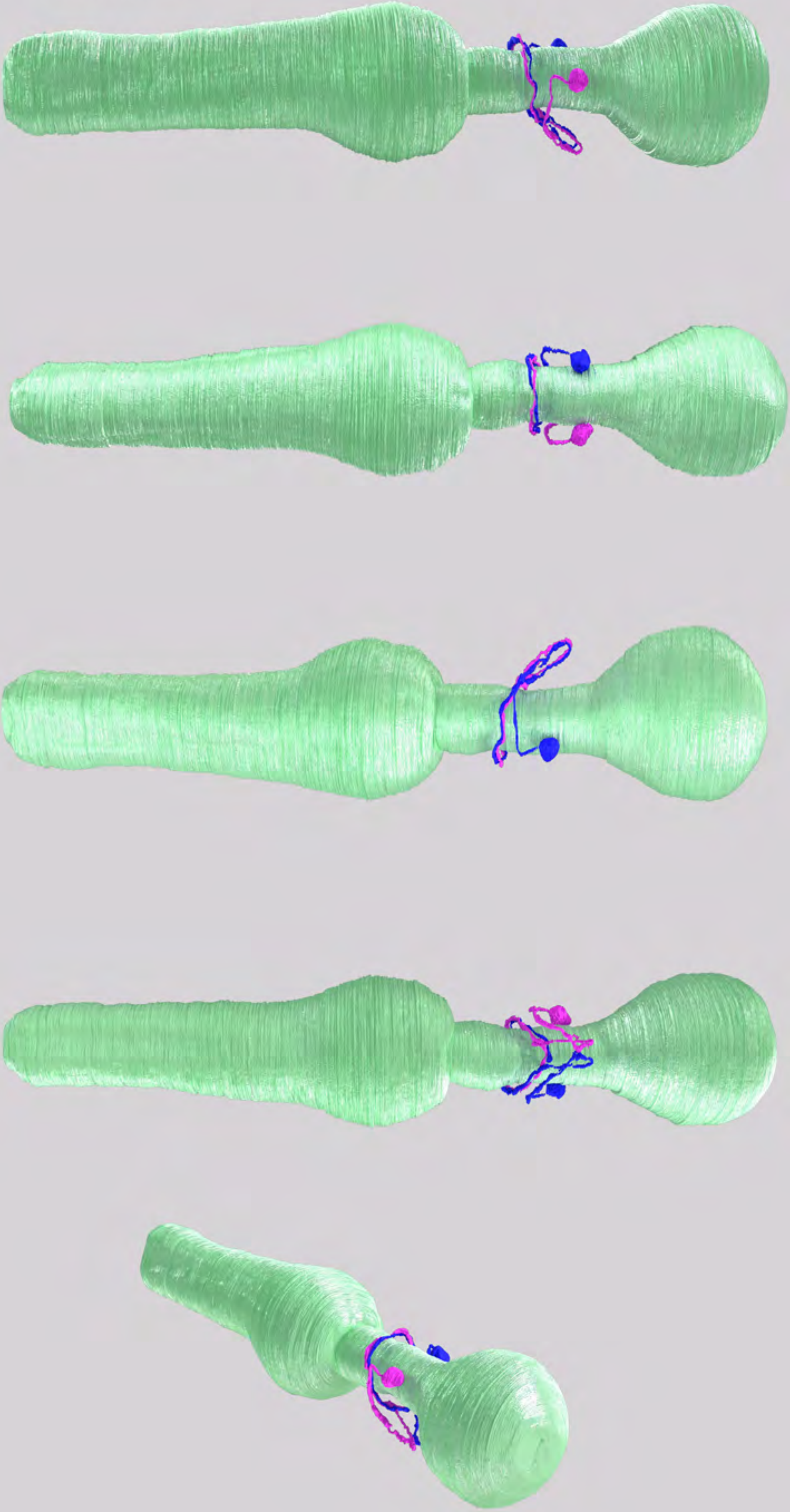

RIBL, RIBR

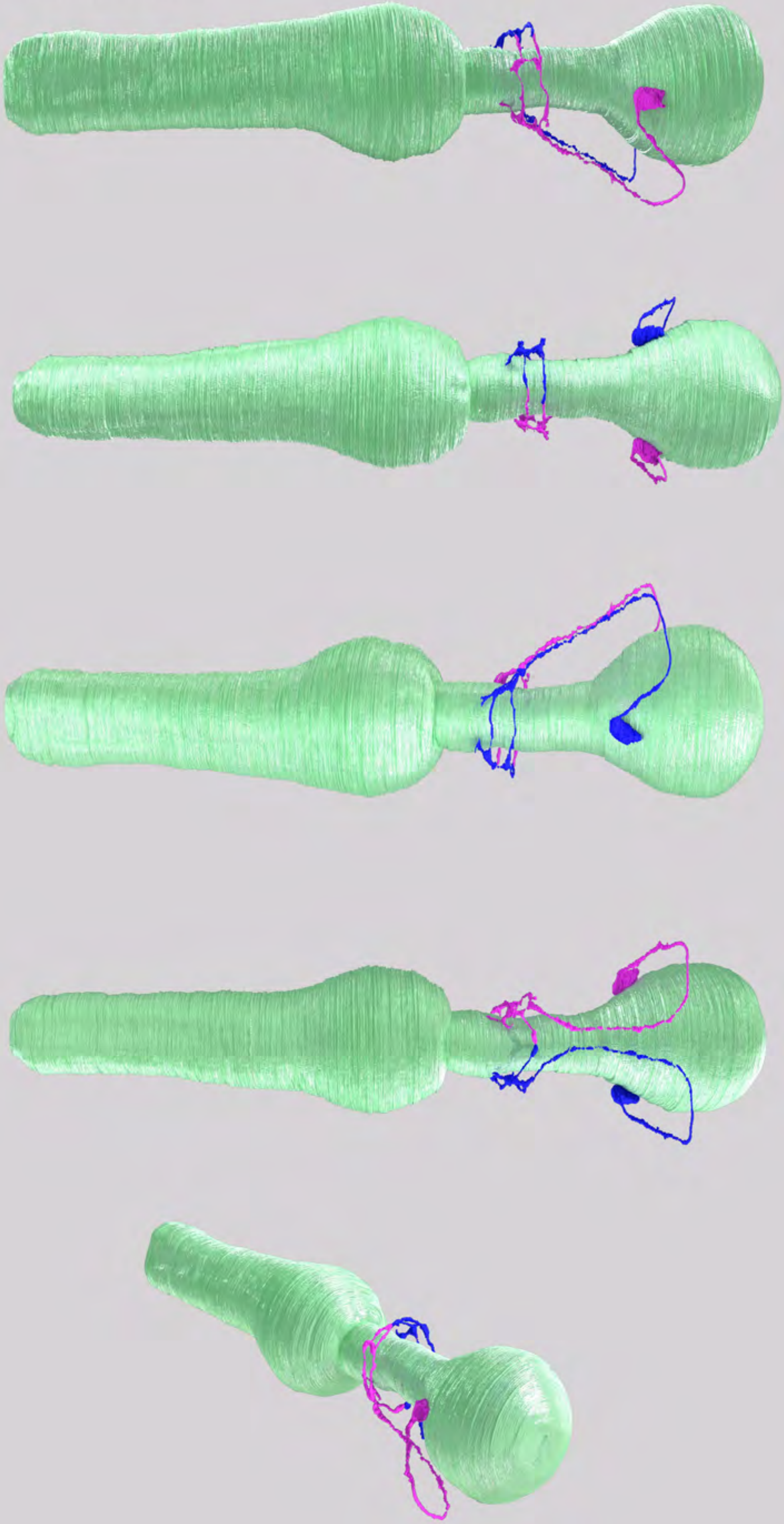

RICL, RICR

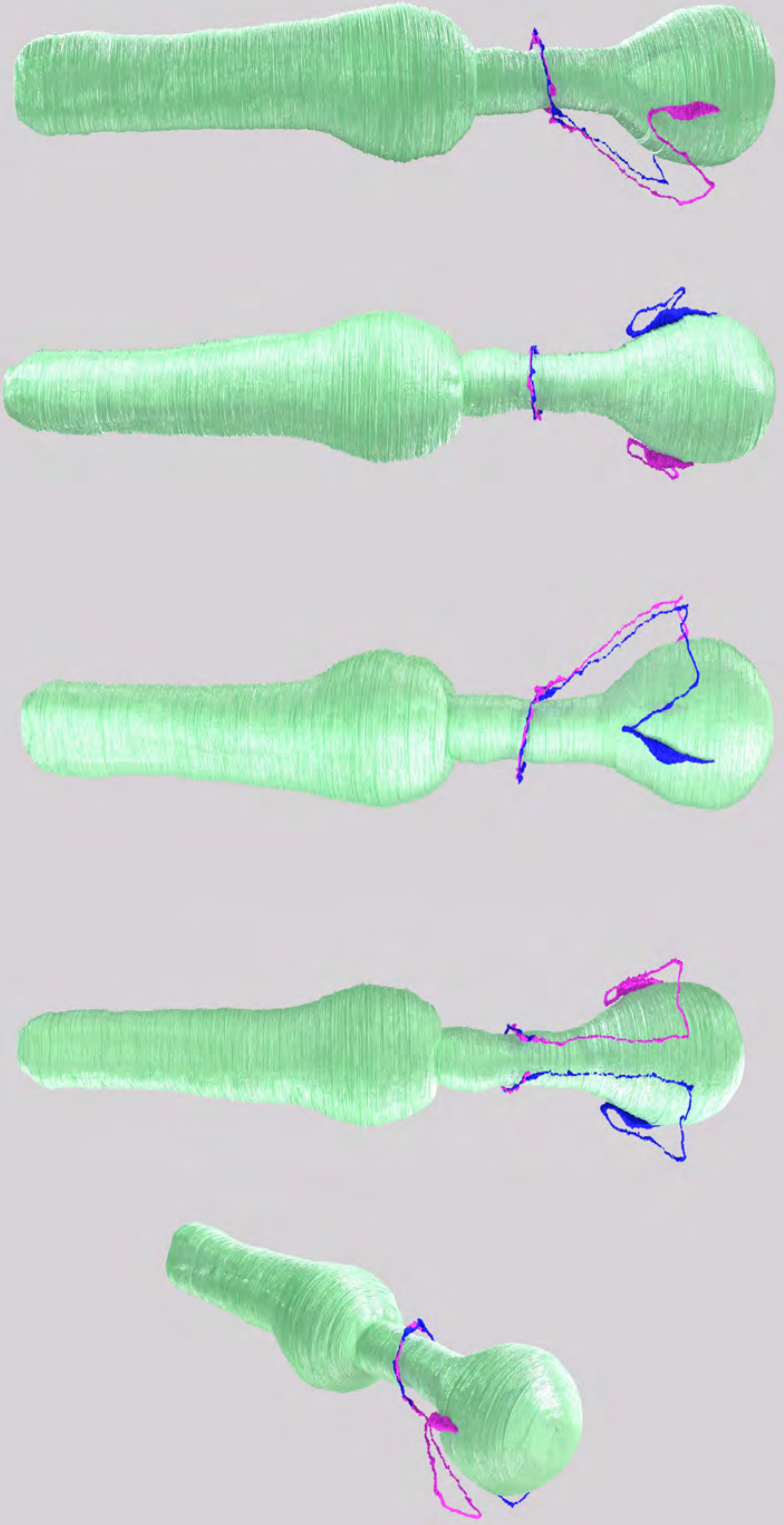

RID

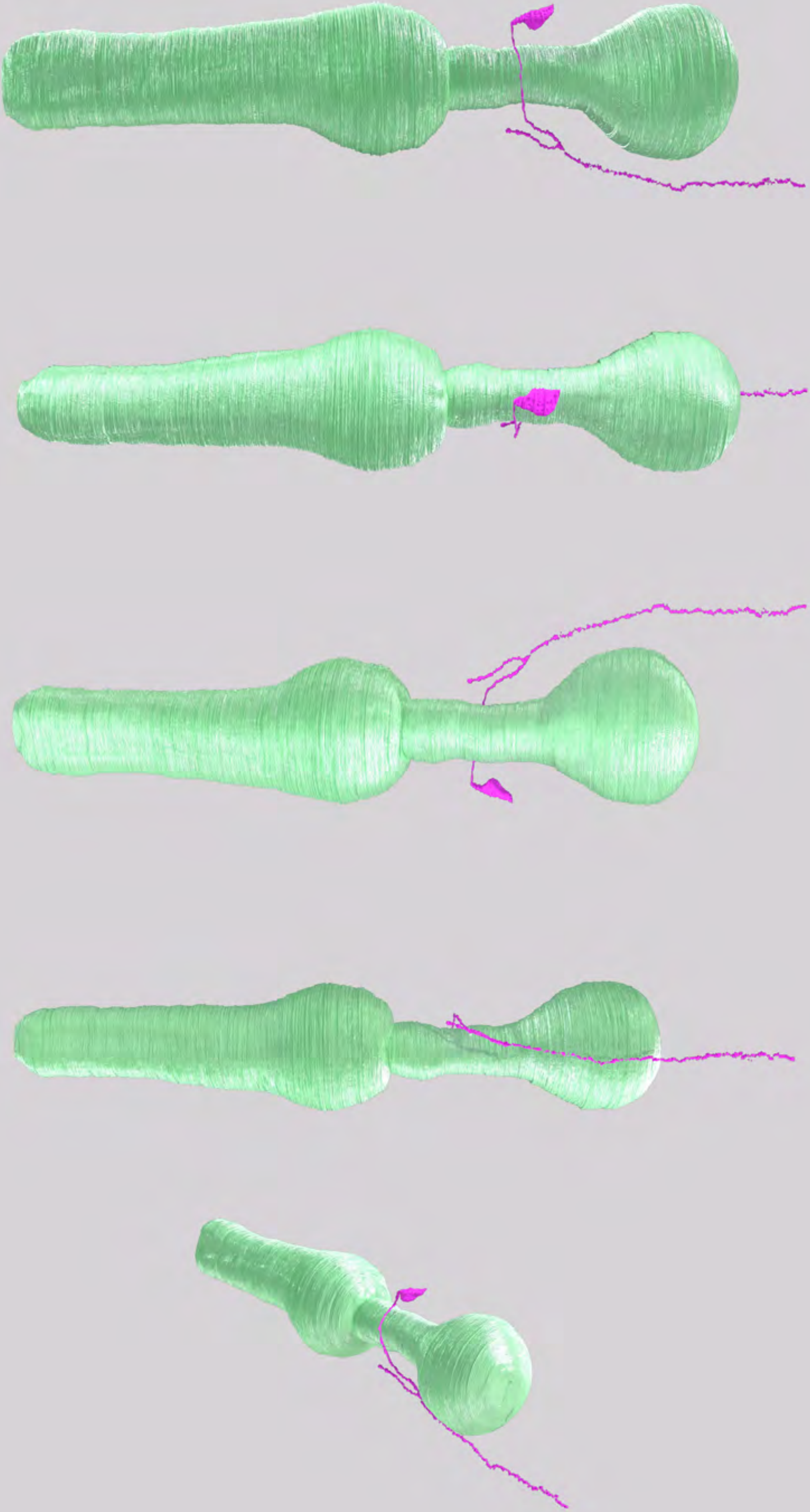

RIFL, RIFR

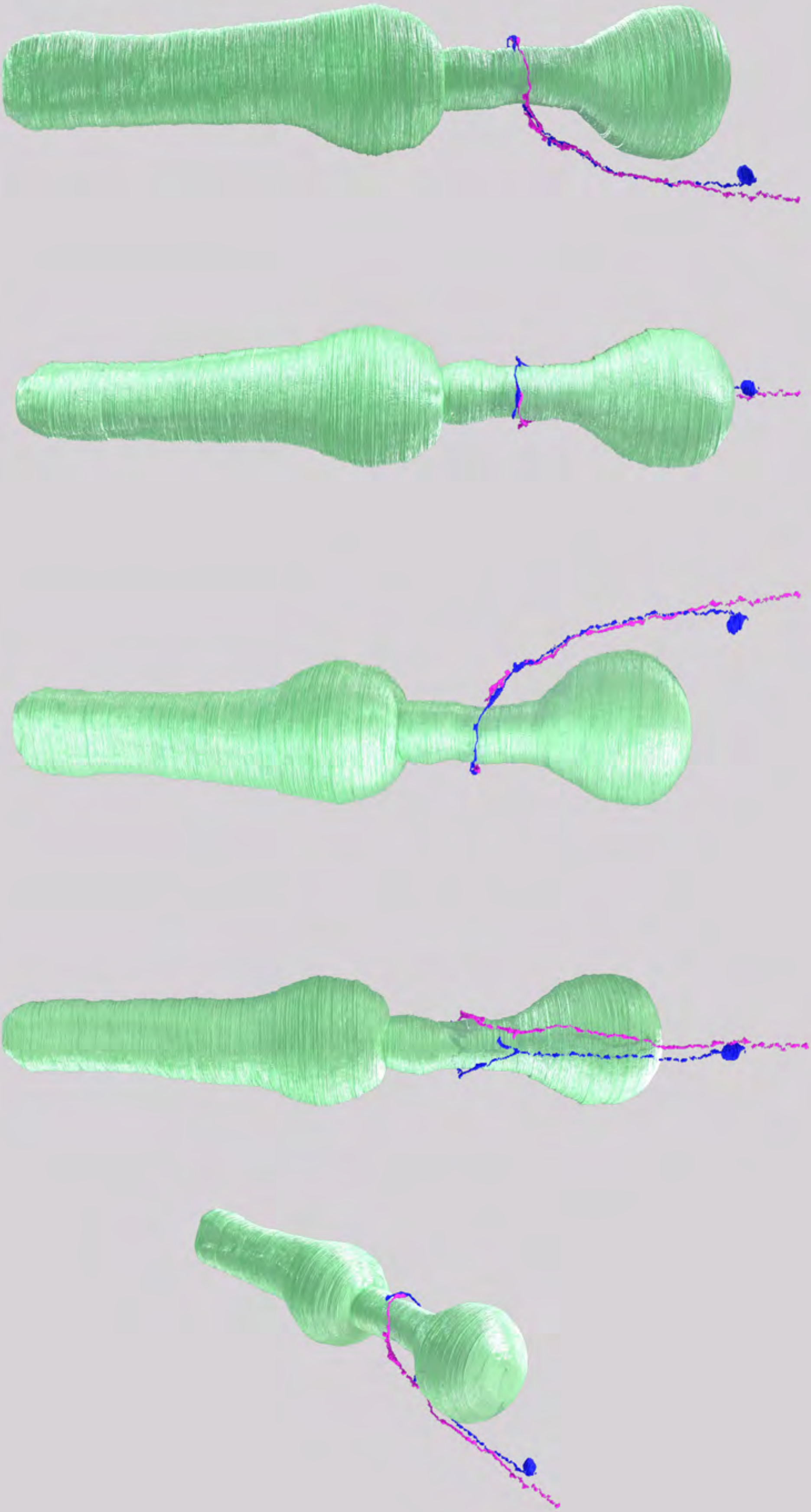

RIGL, RIGR

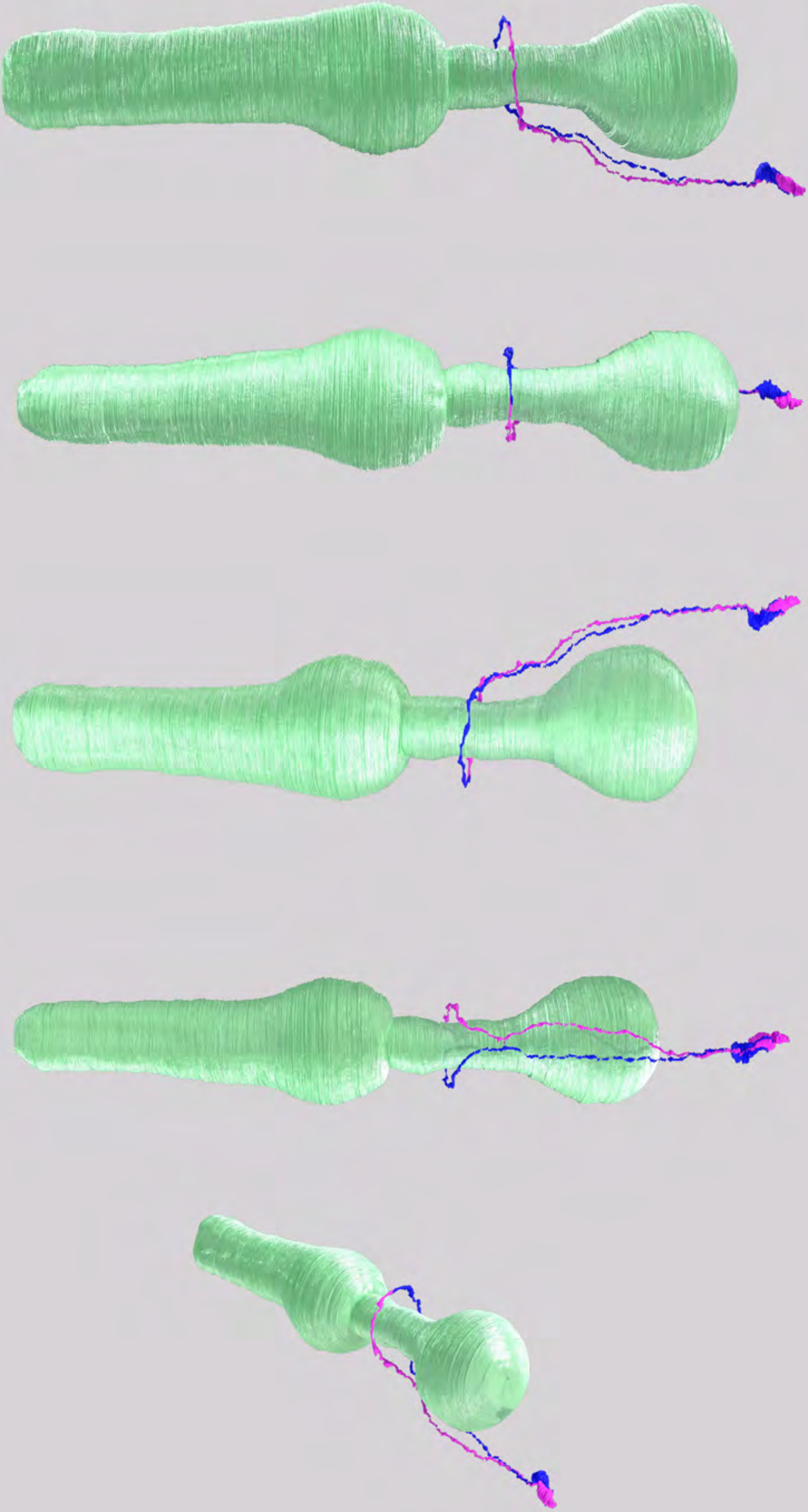

RIH

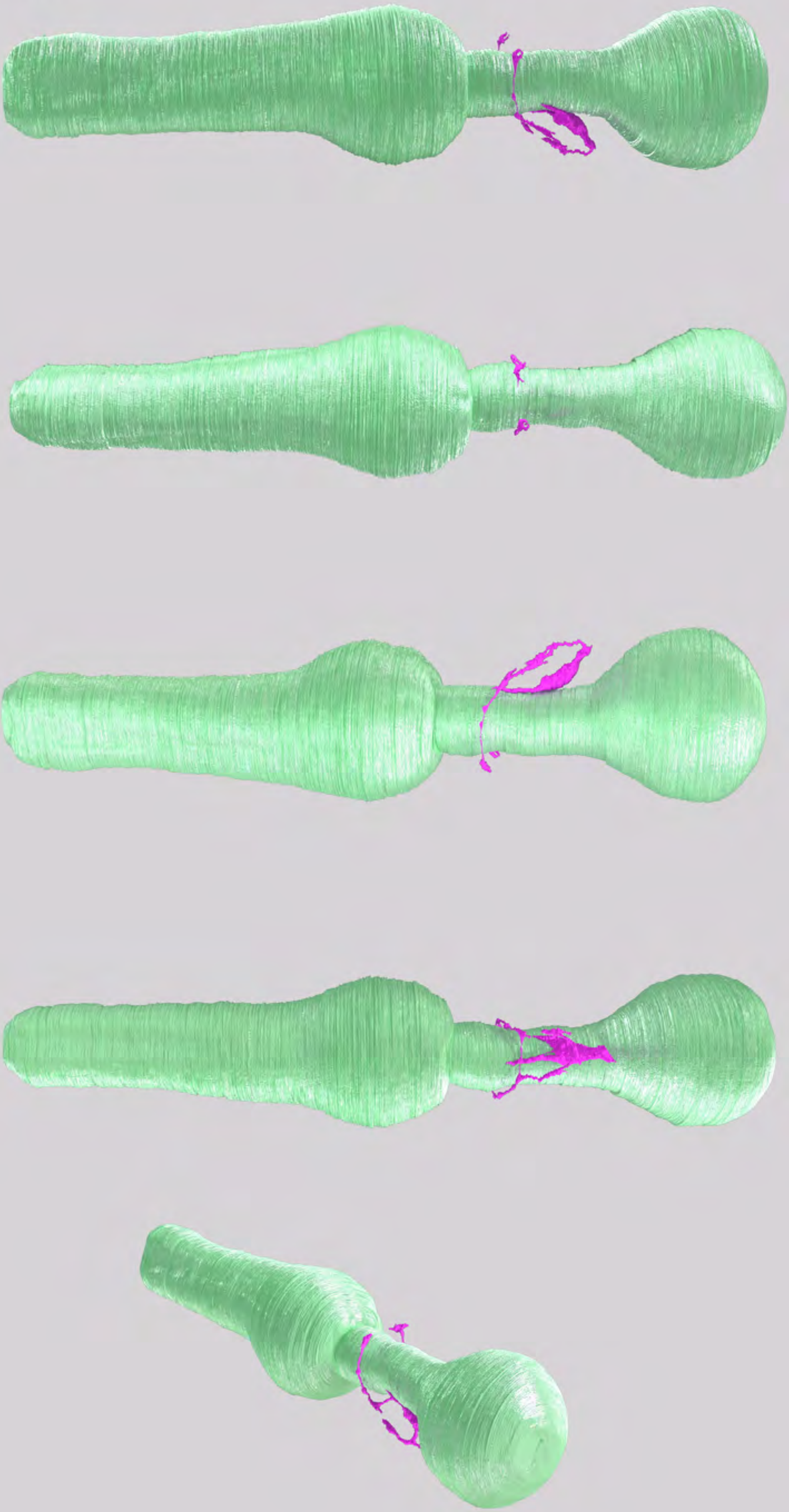

RIML, RIMR

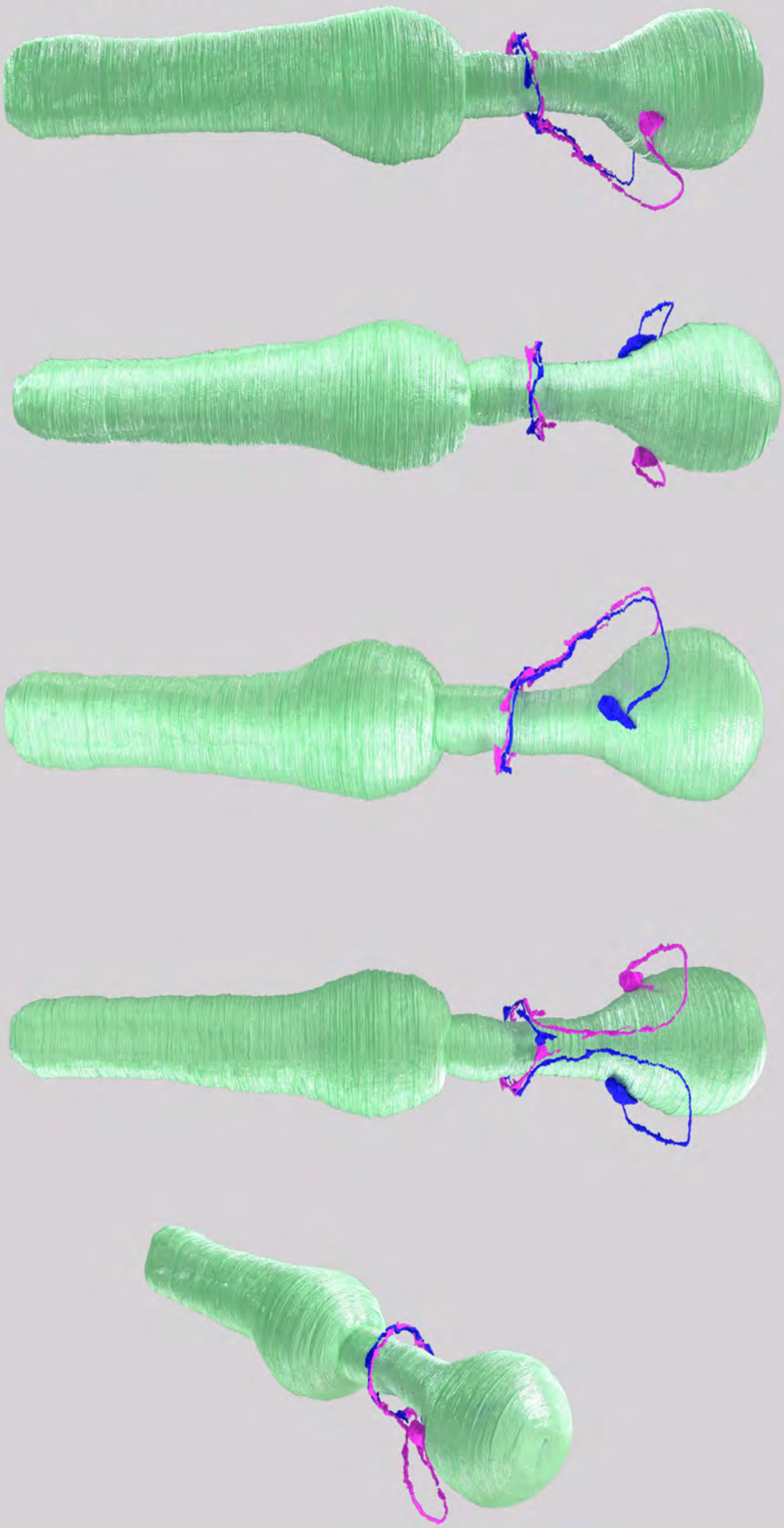

RIPL, RIPR

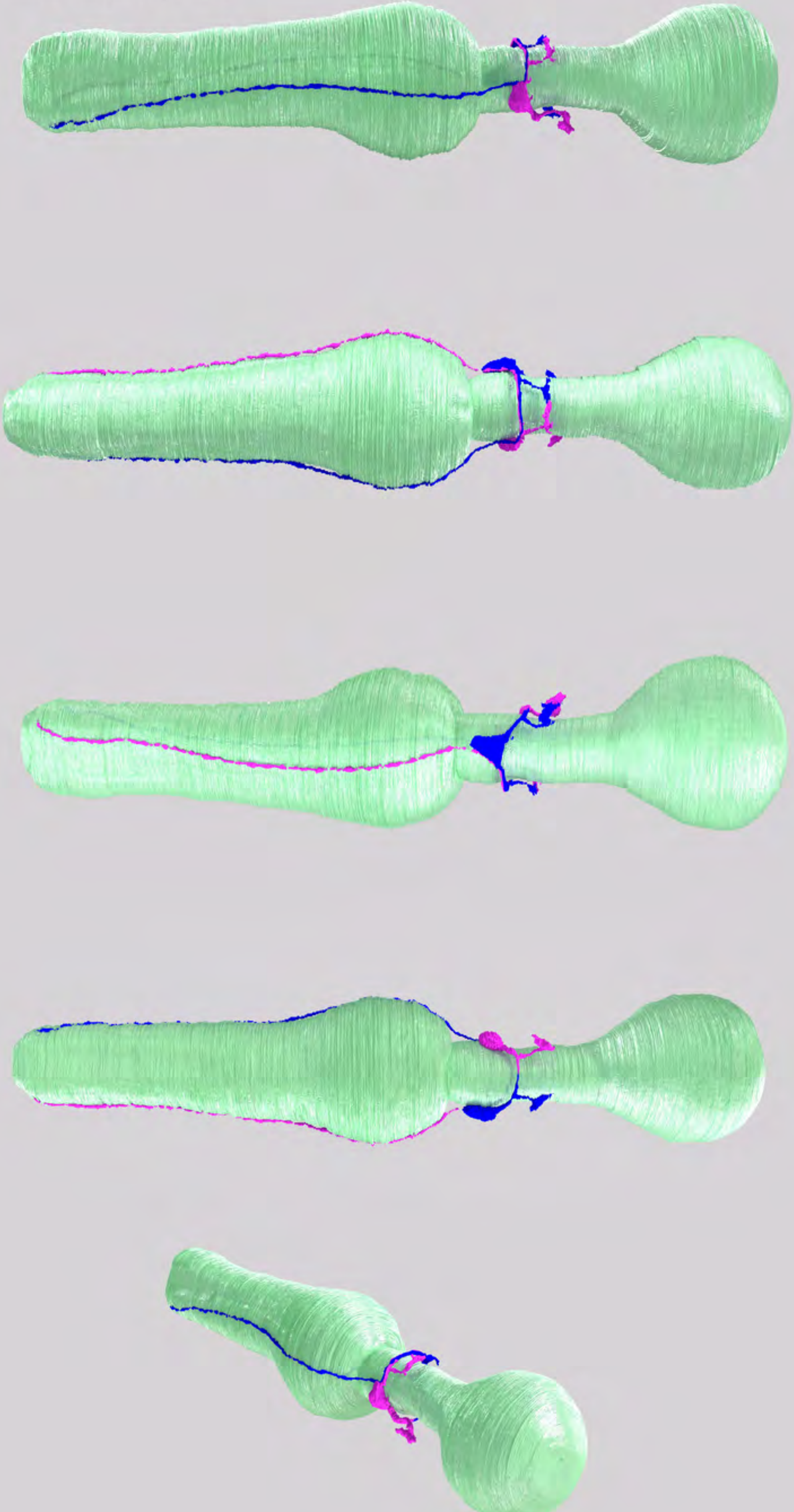

RIR

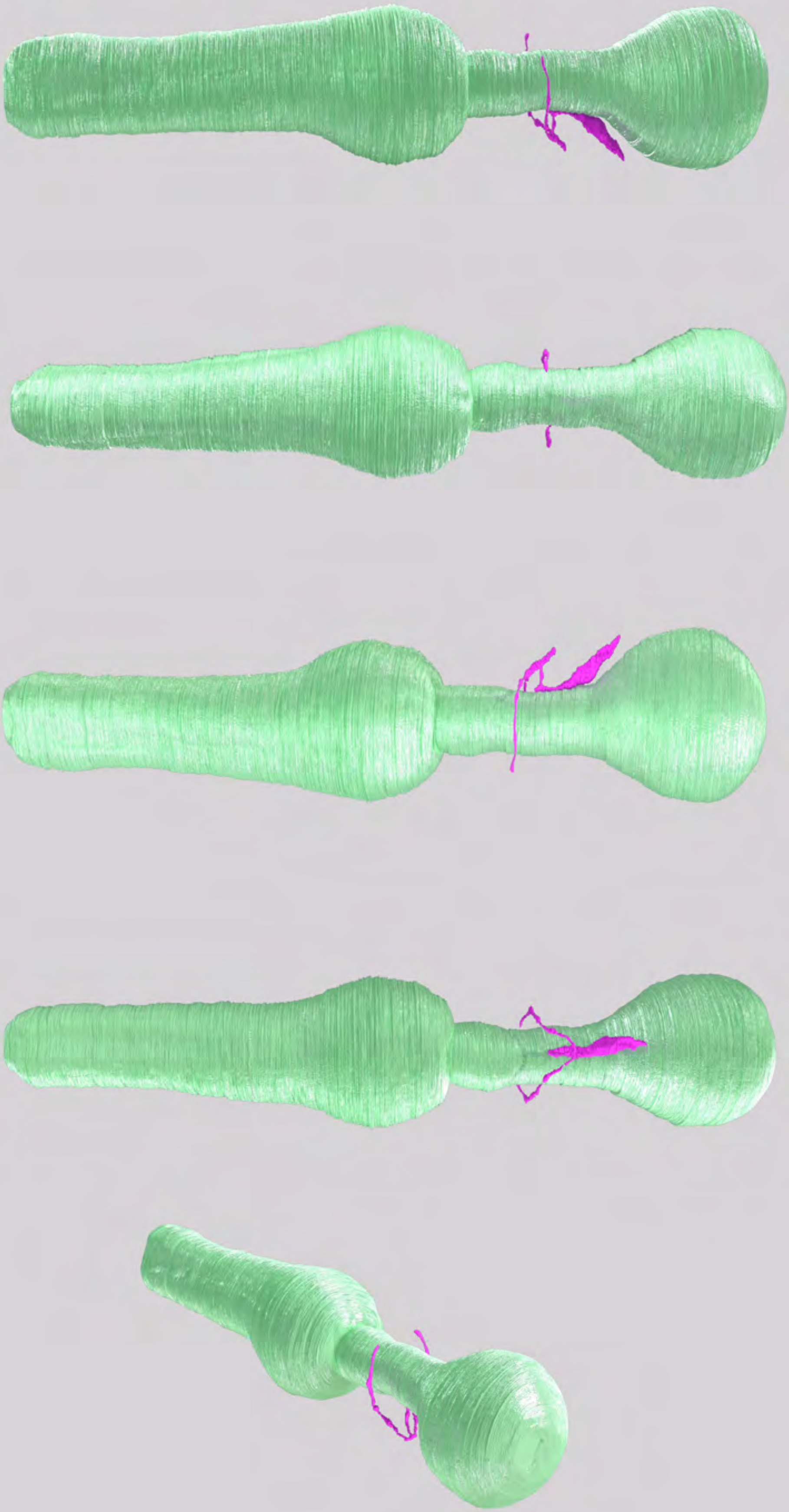

RIS

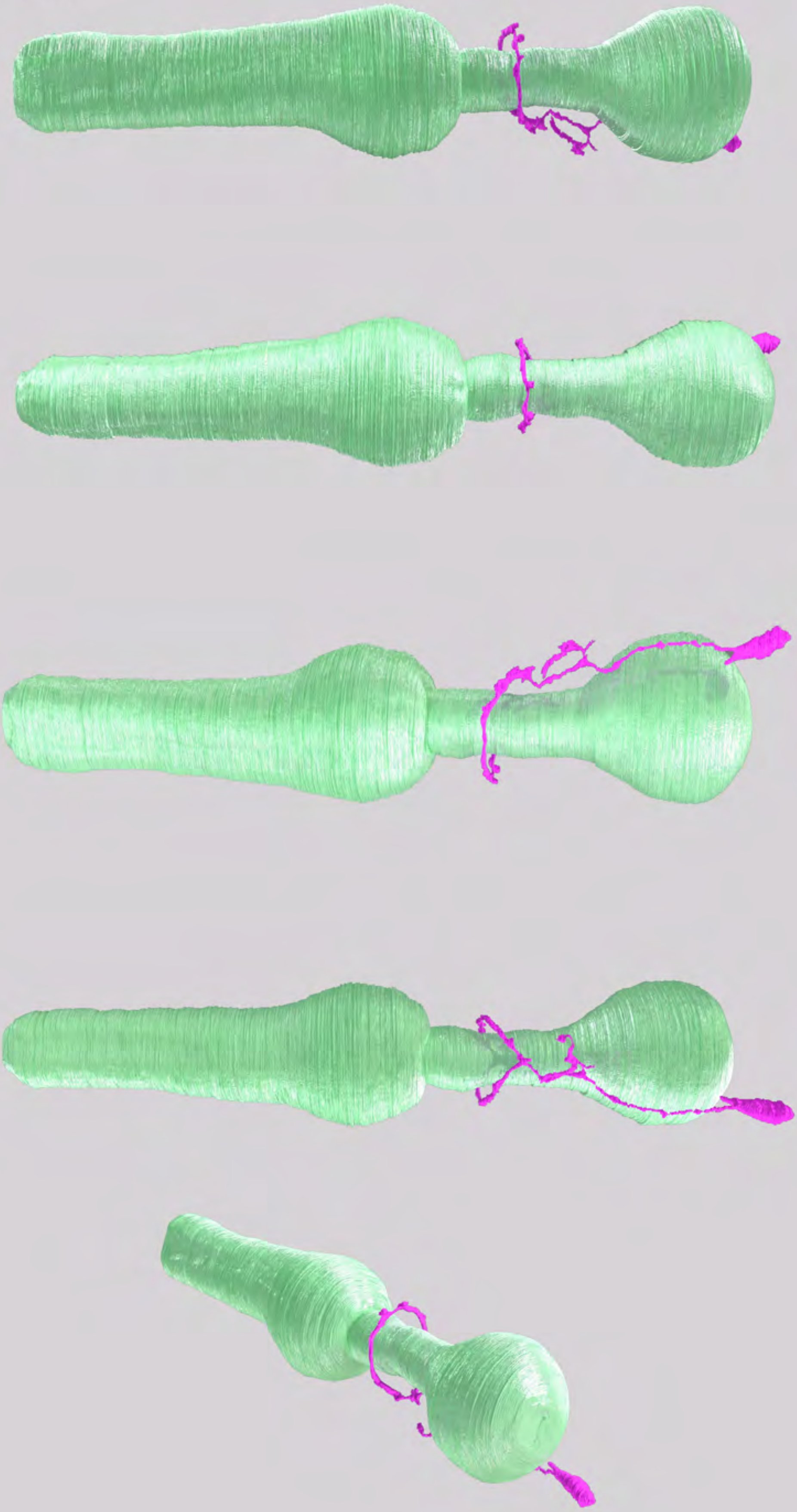

RIVL, RIVR

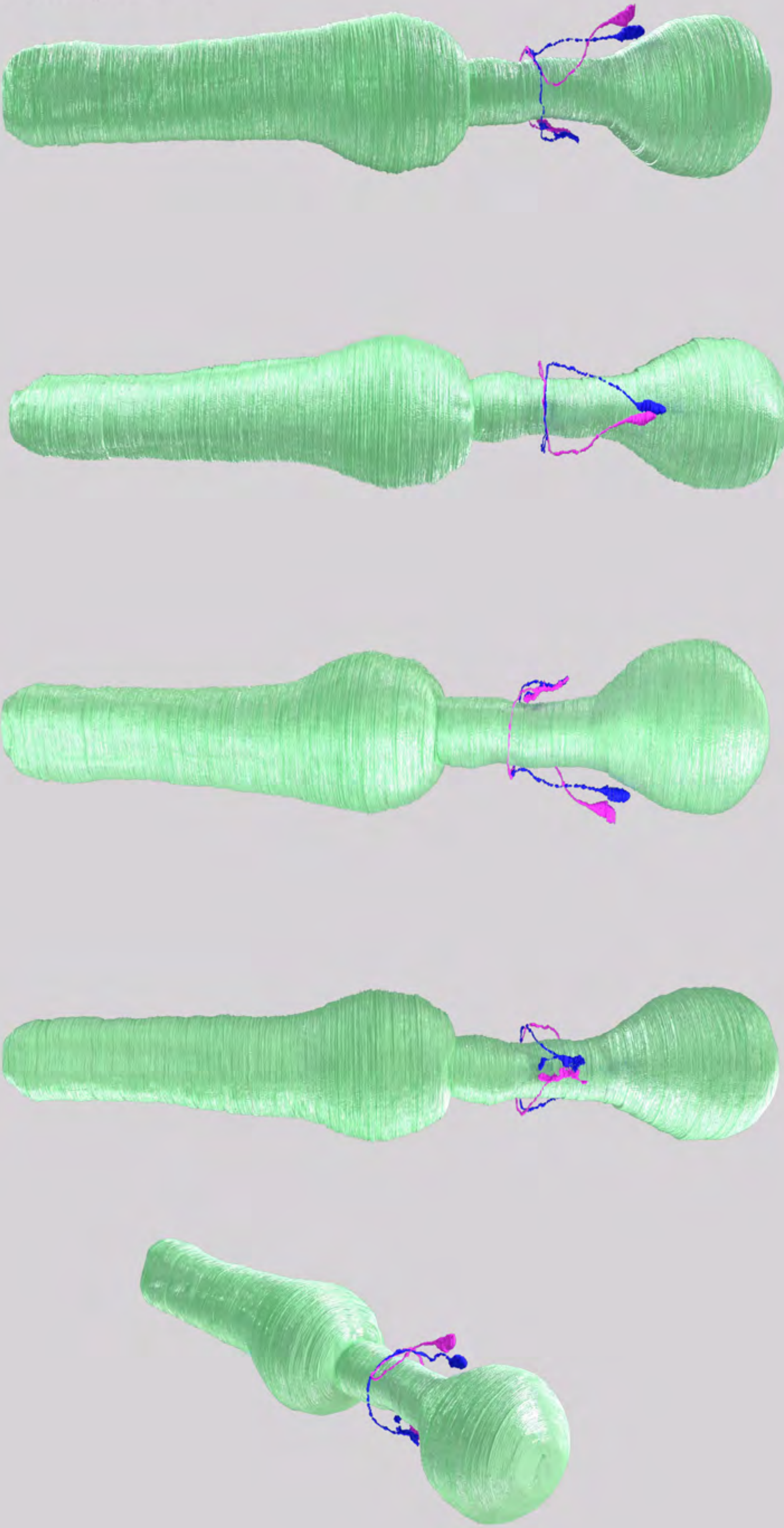

RMDDL, RMDDR

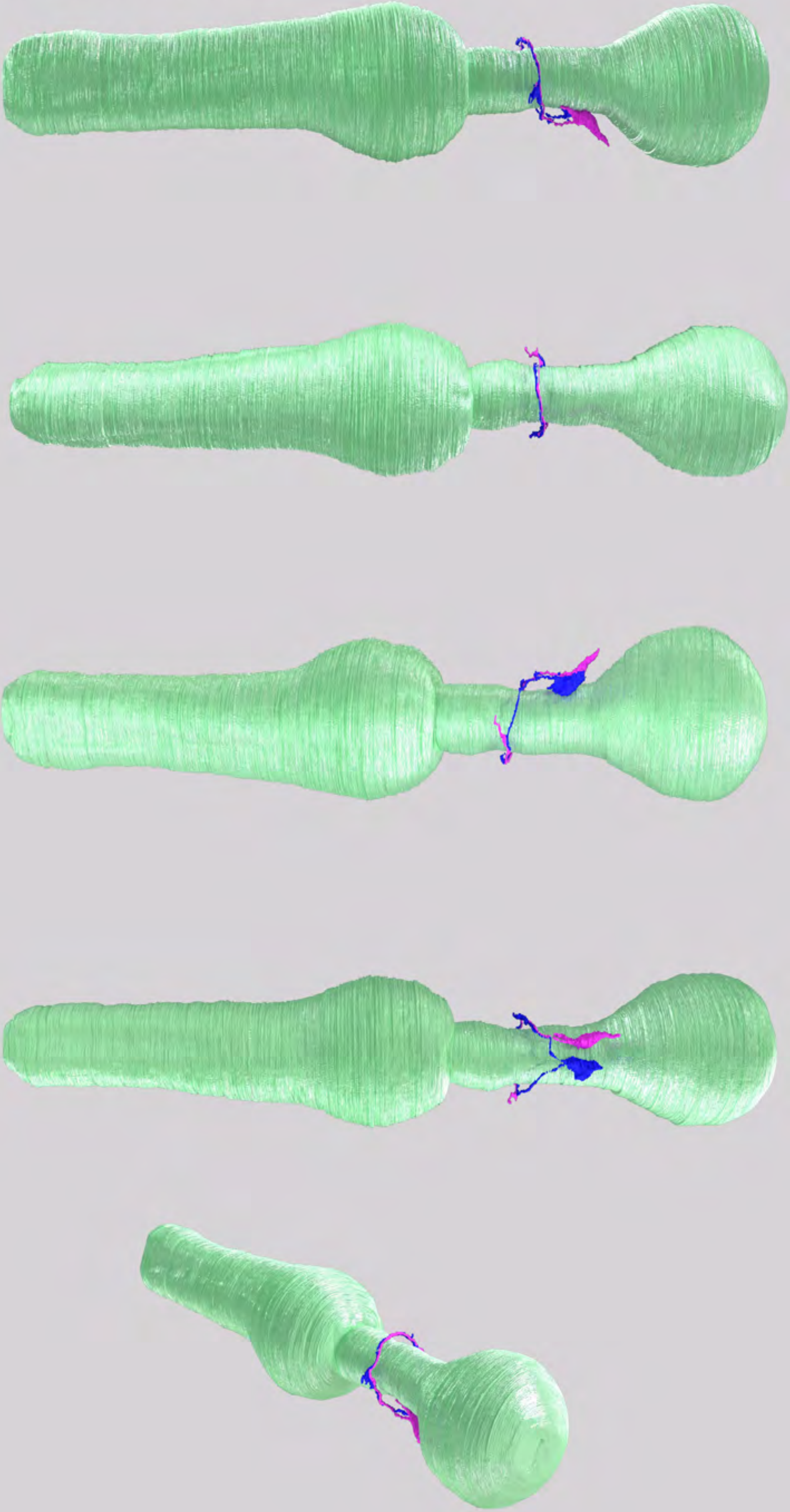

RMDL, RMDR

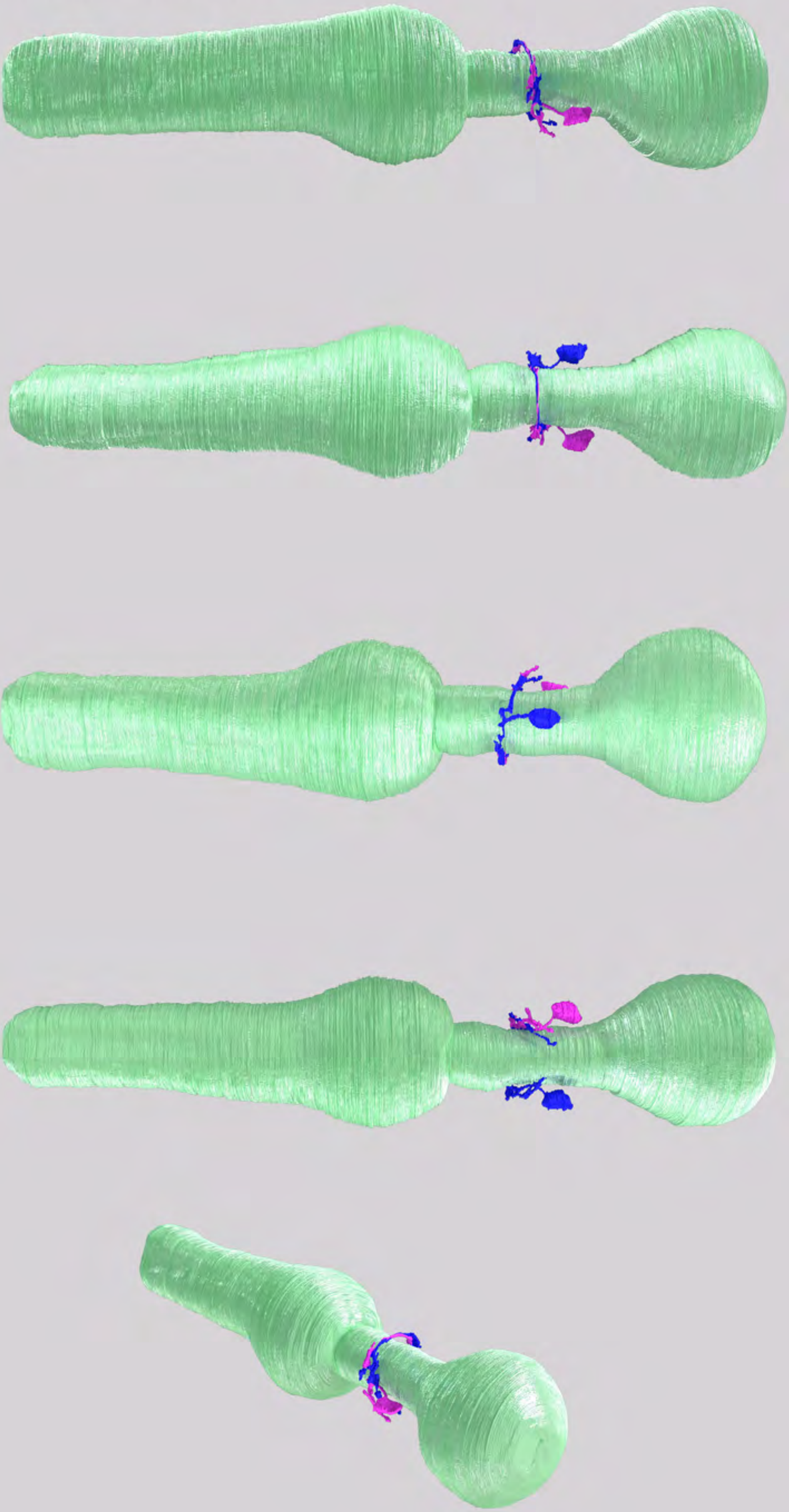

RMDVL, RMDVR

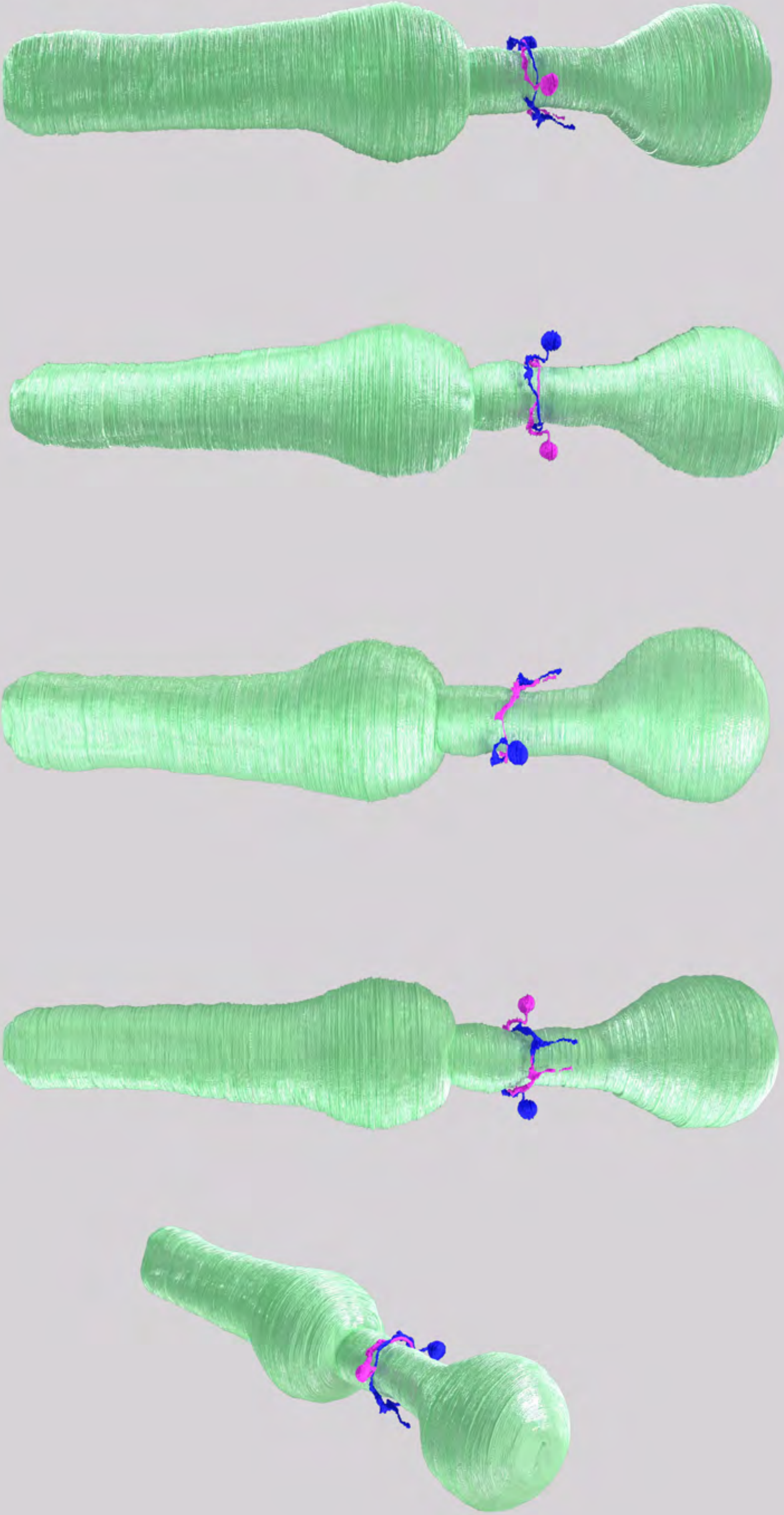

RMED, RMEV

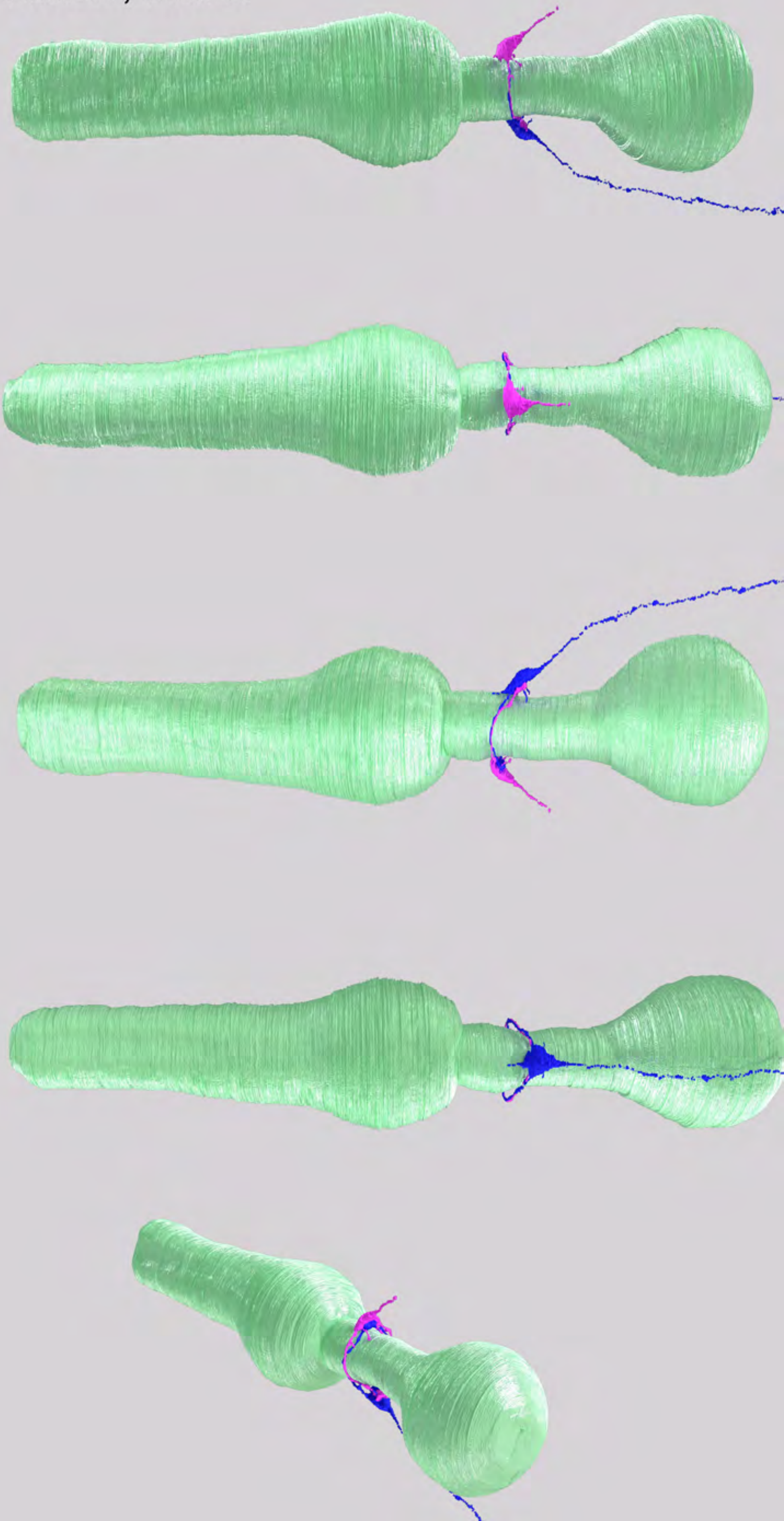

RMEL, RMER

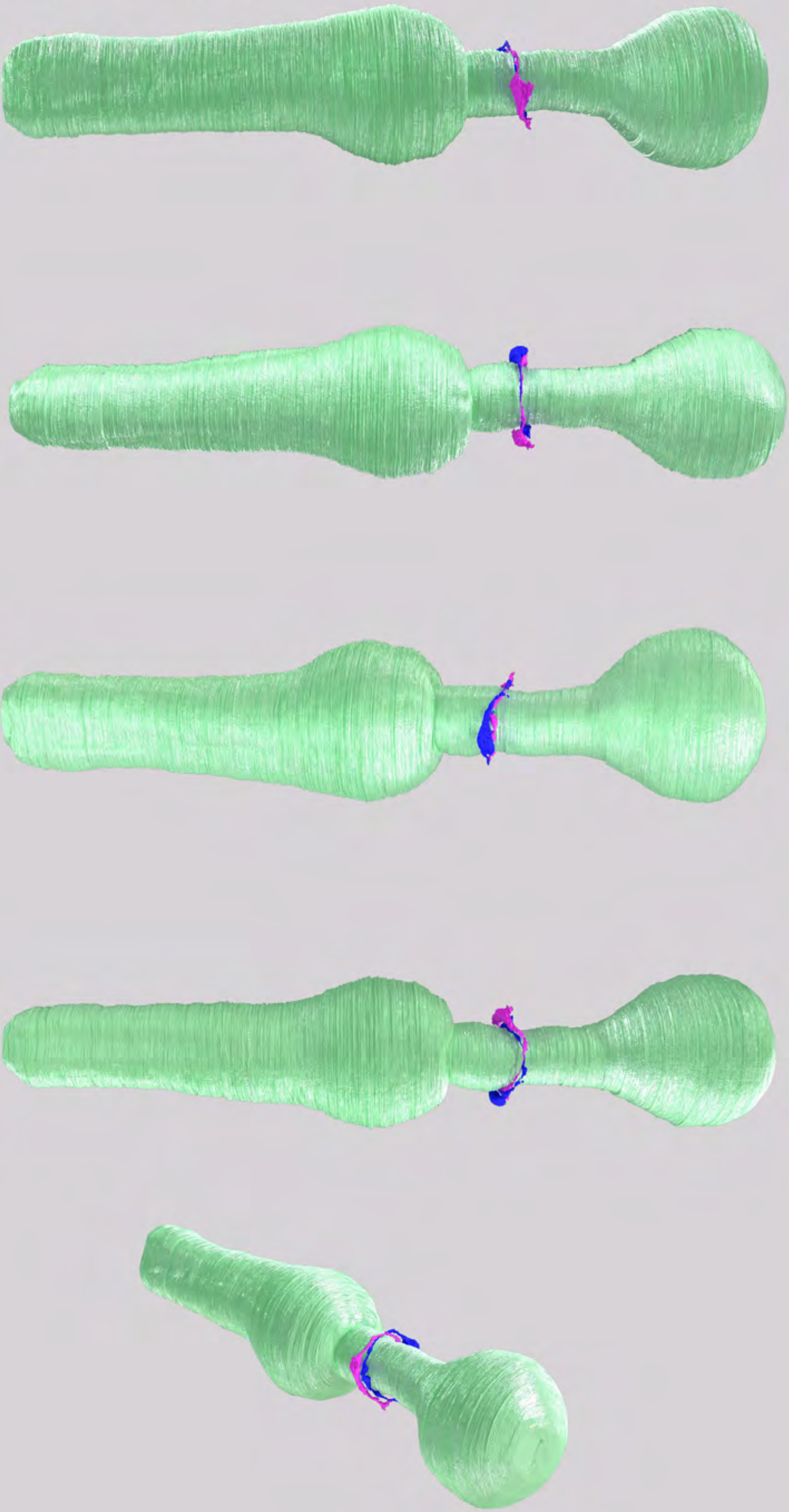

RMFL, RMFR

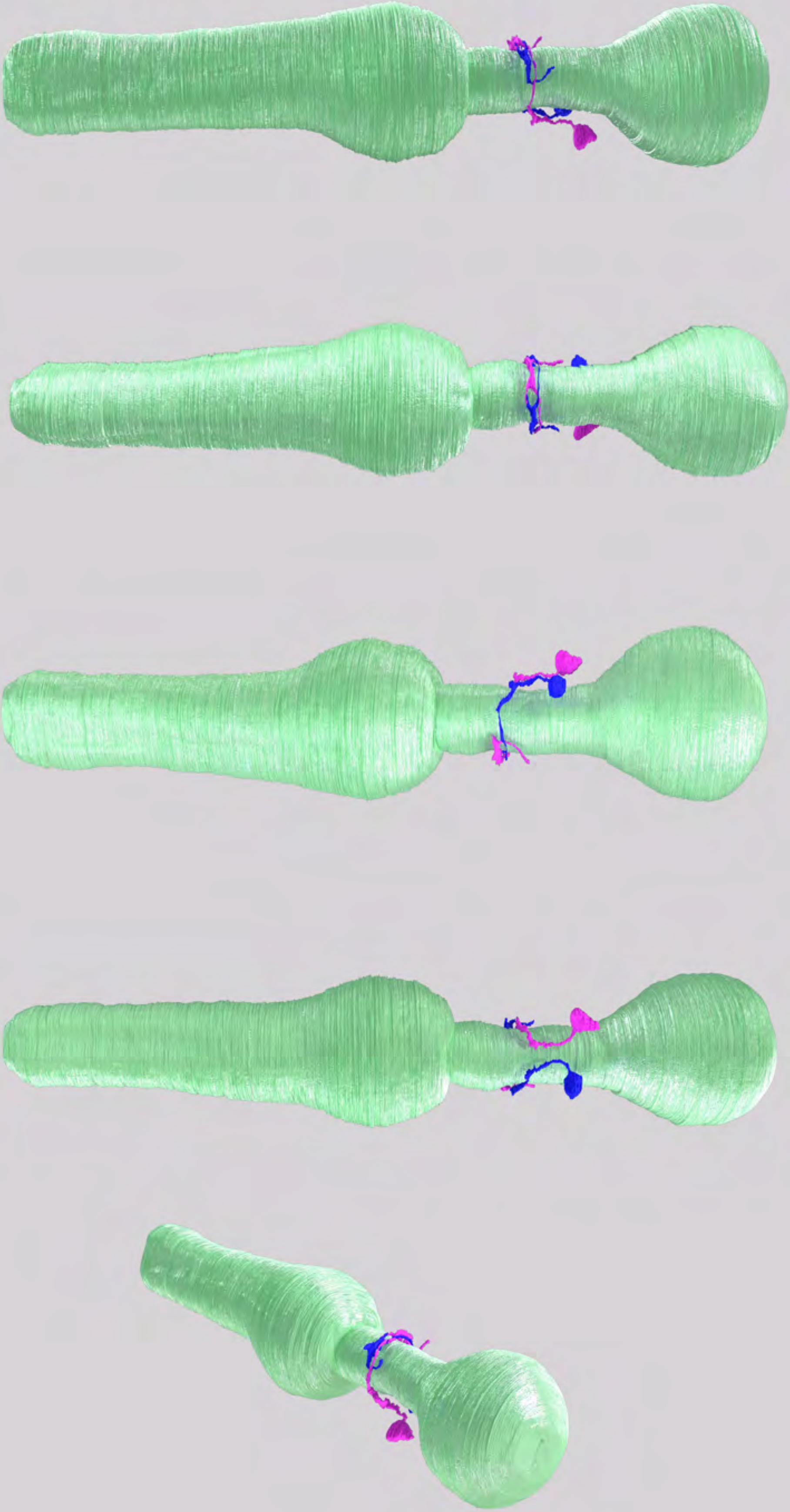

RMGL, RMGR

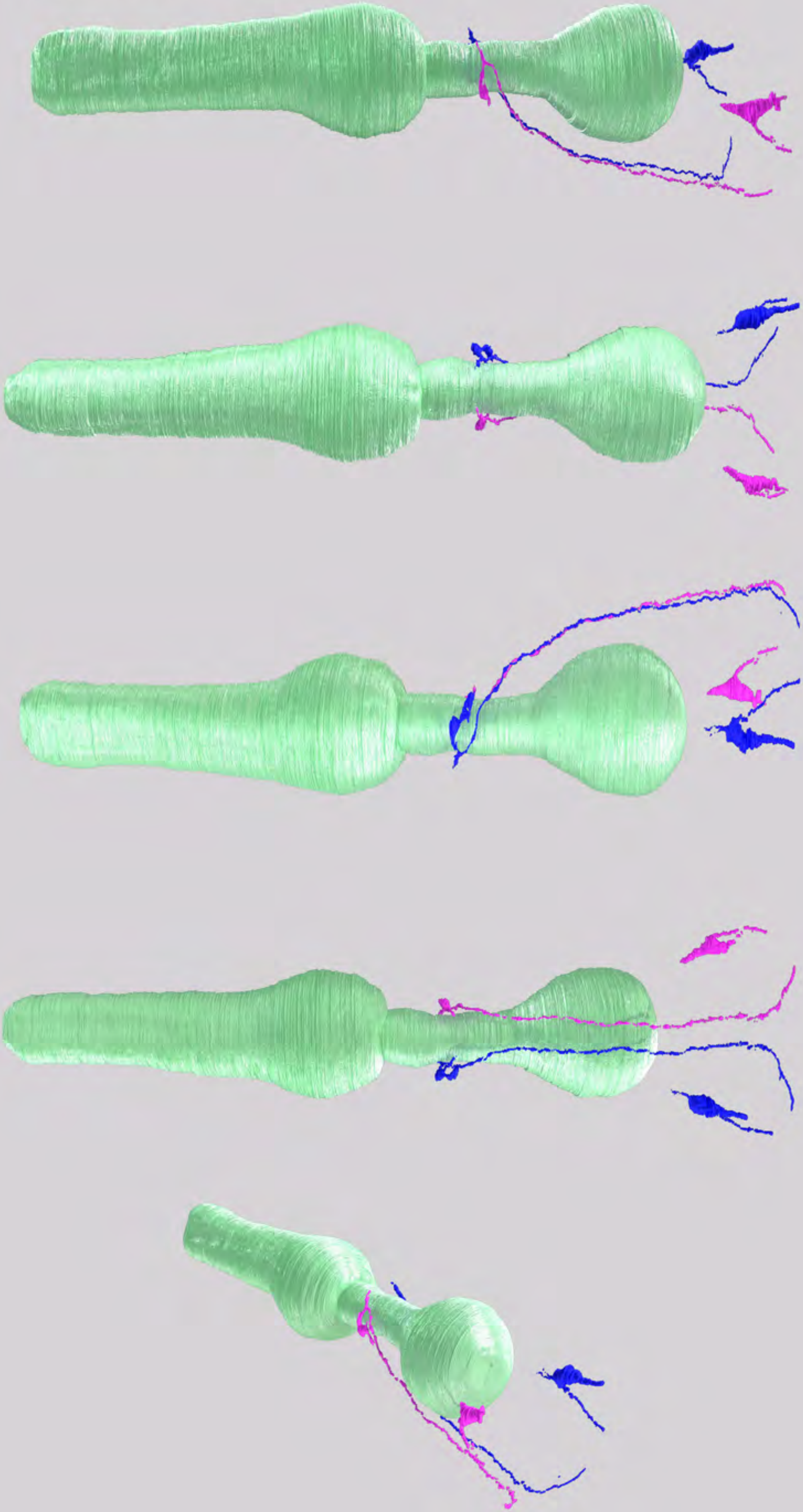

RMHL, RMHR

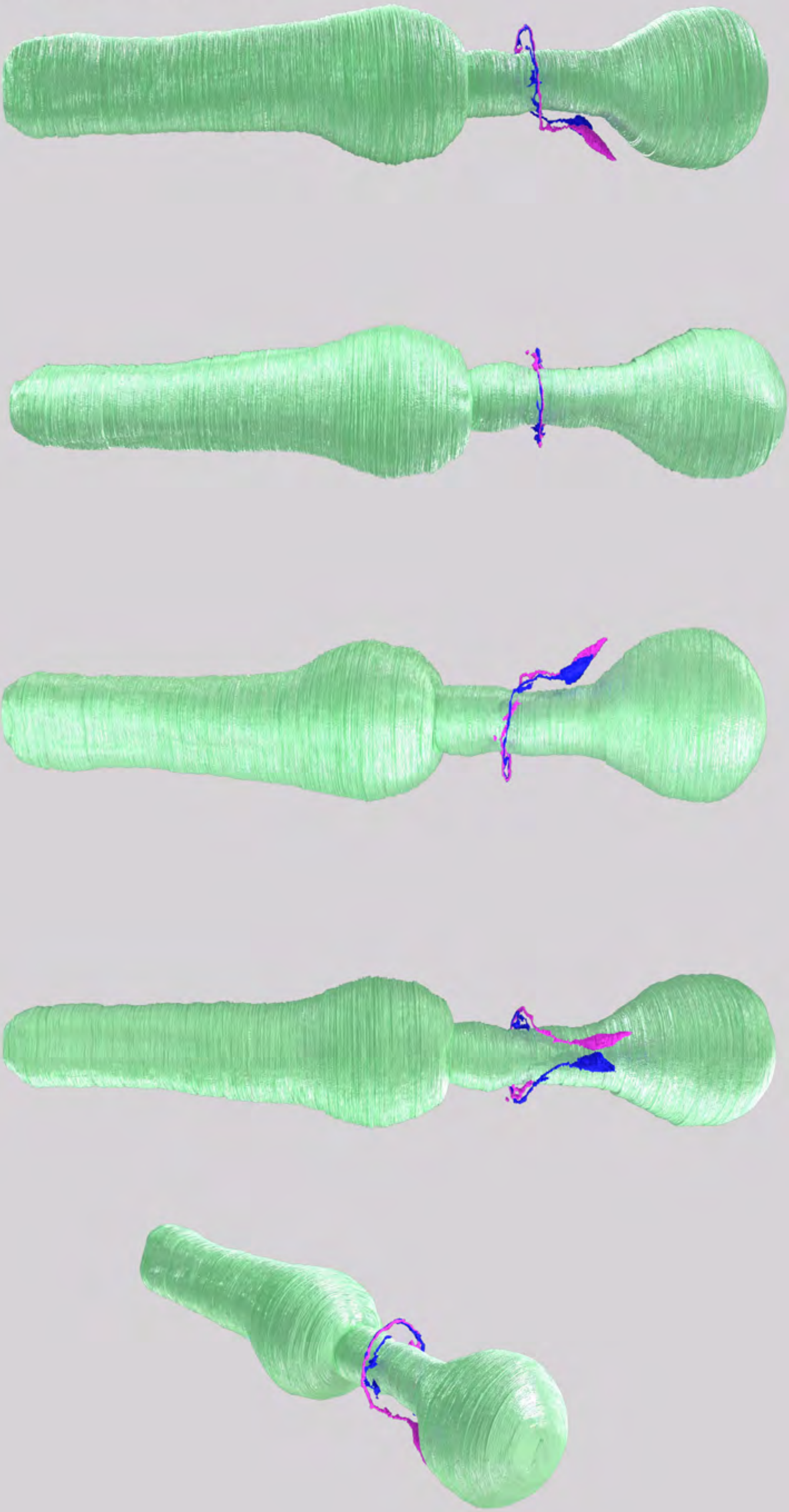

SAADL, SAADR

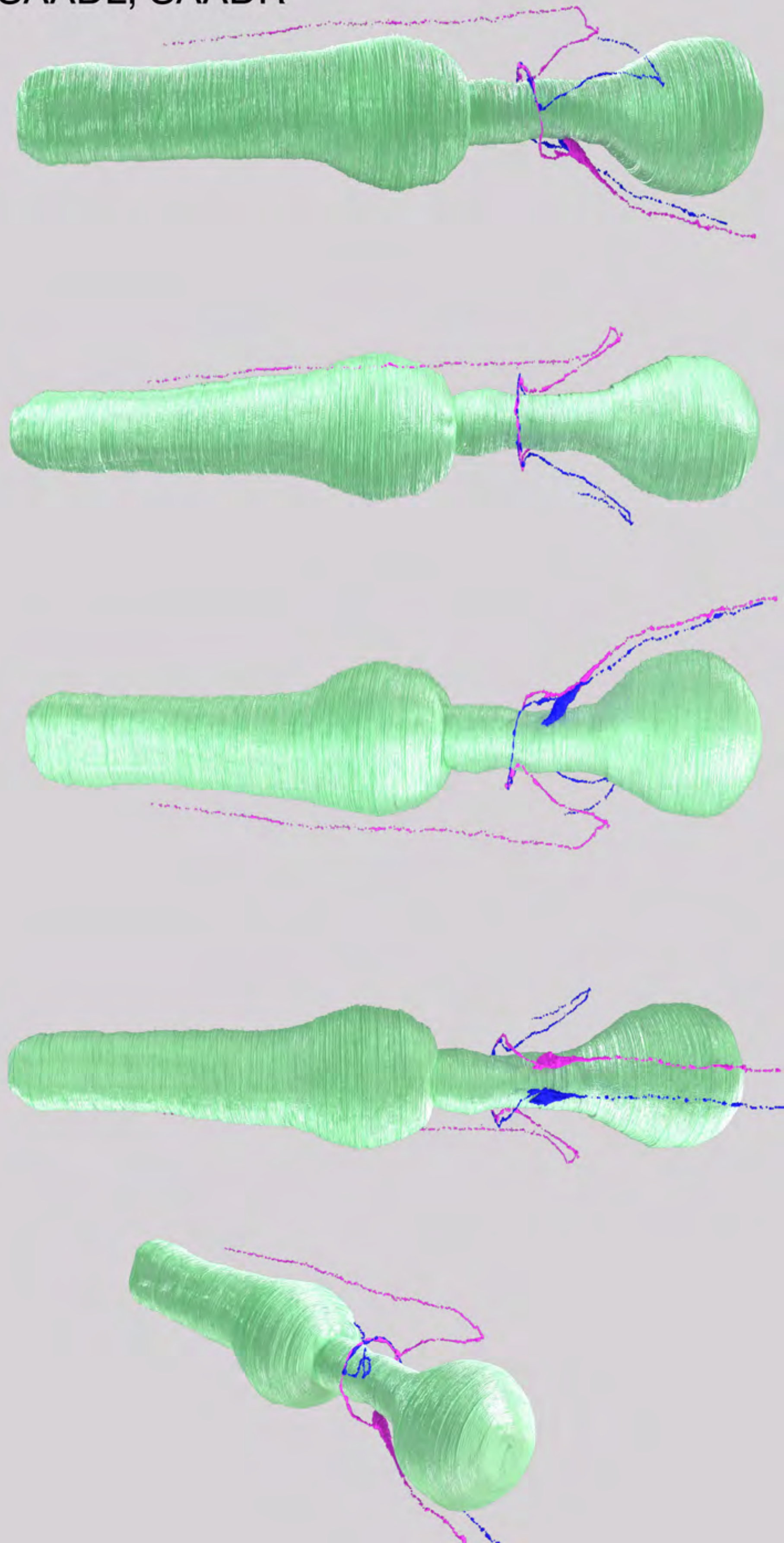

SAAVL, SAAVR

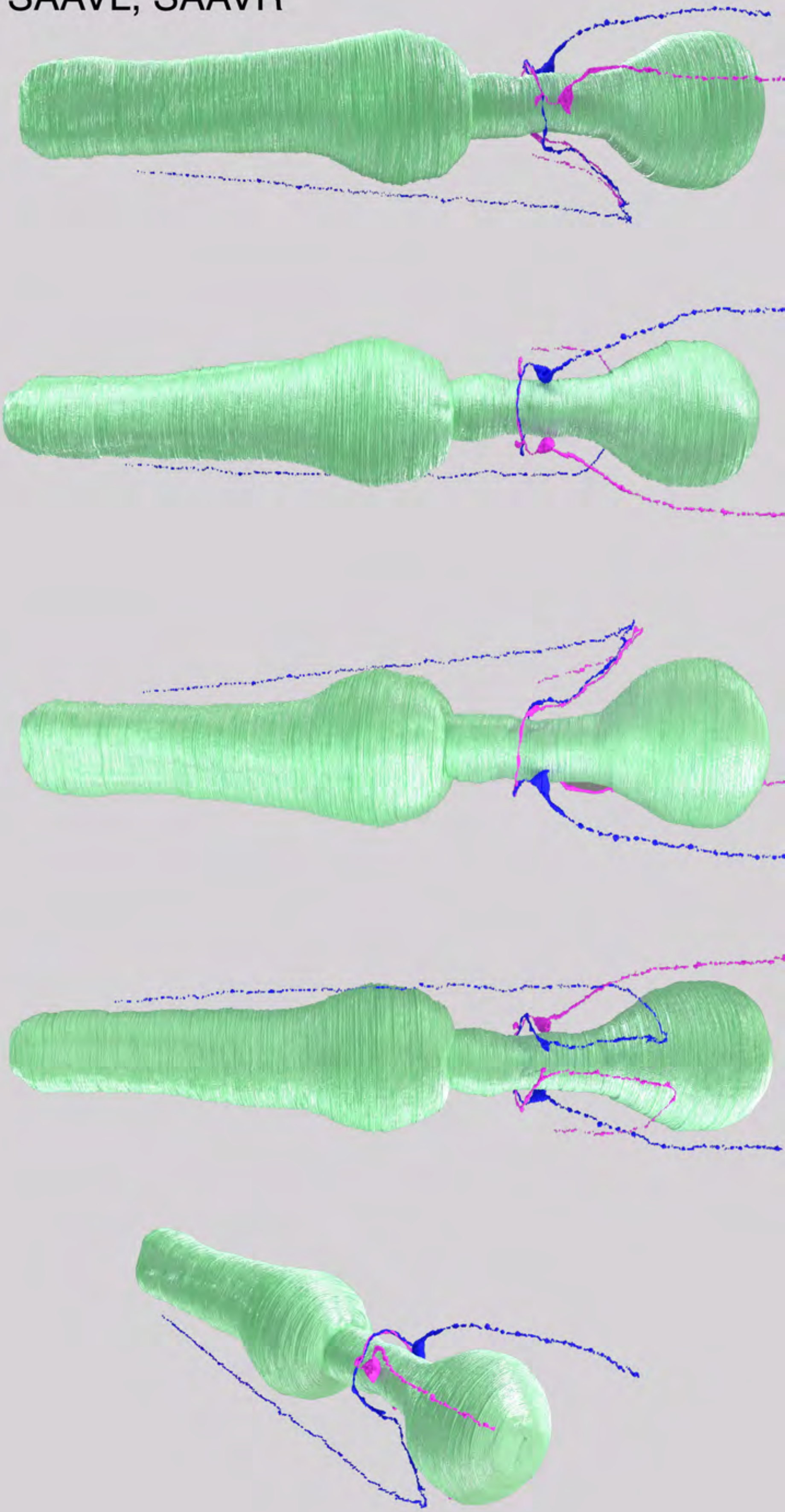

SABD

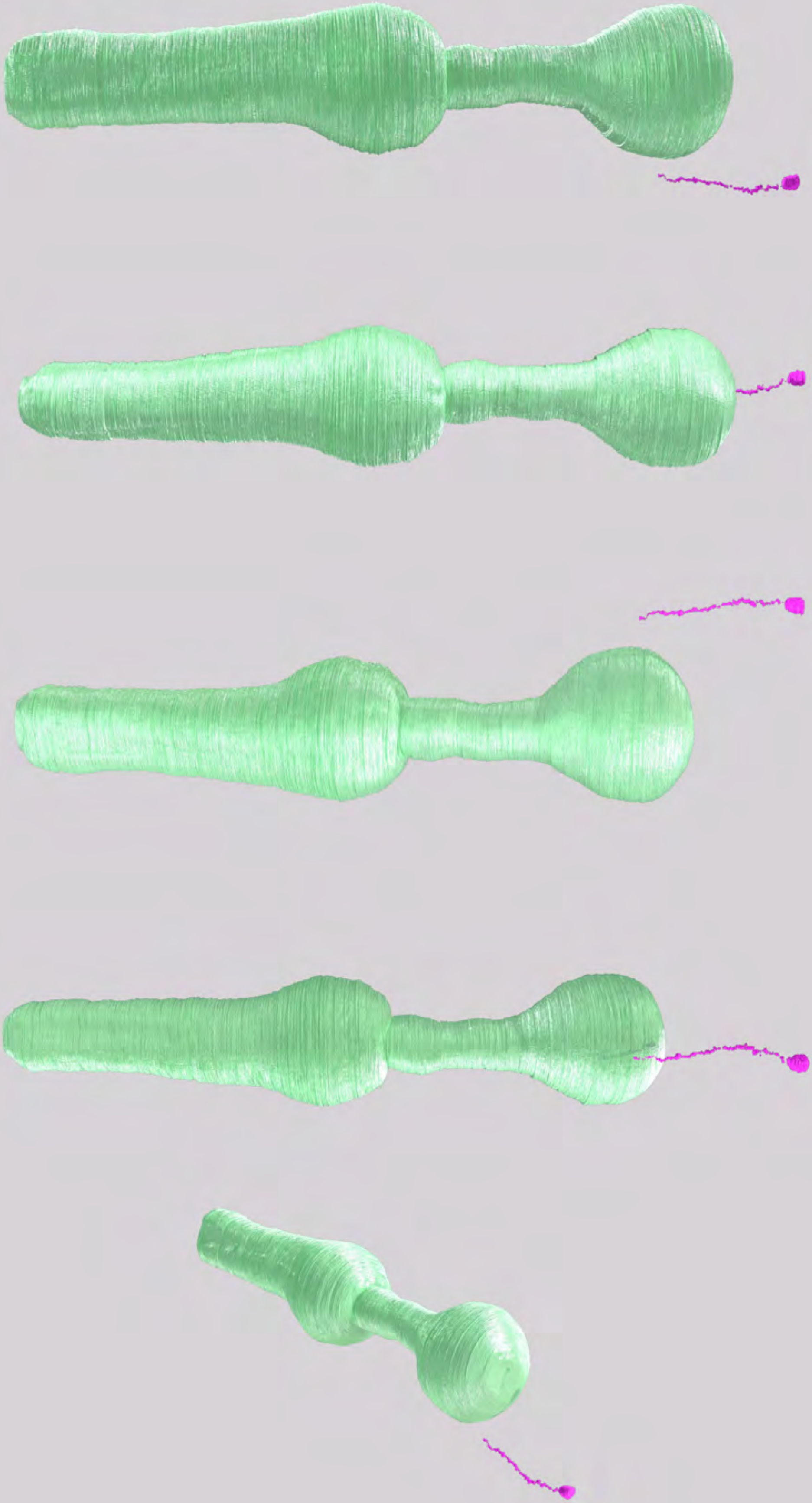

SABVL, SABVR

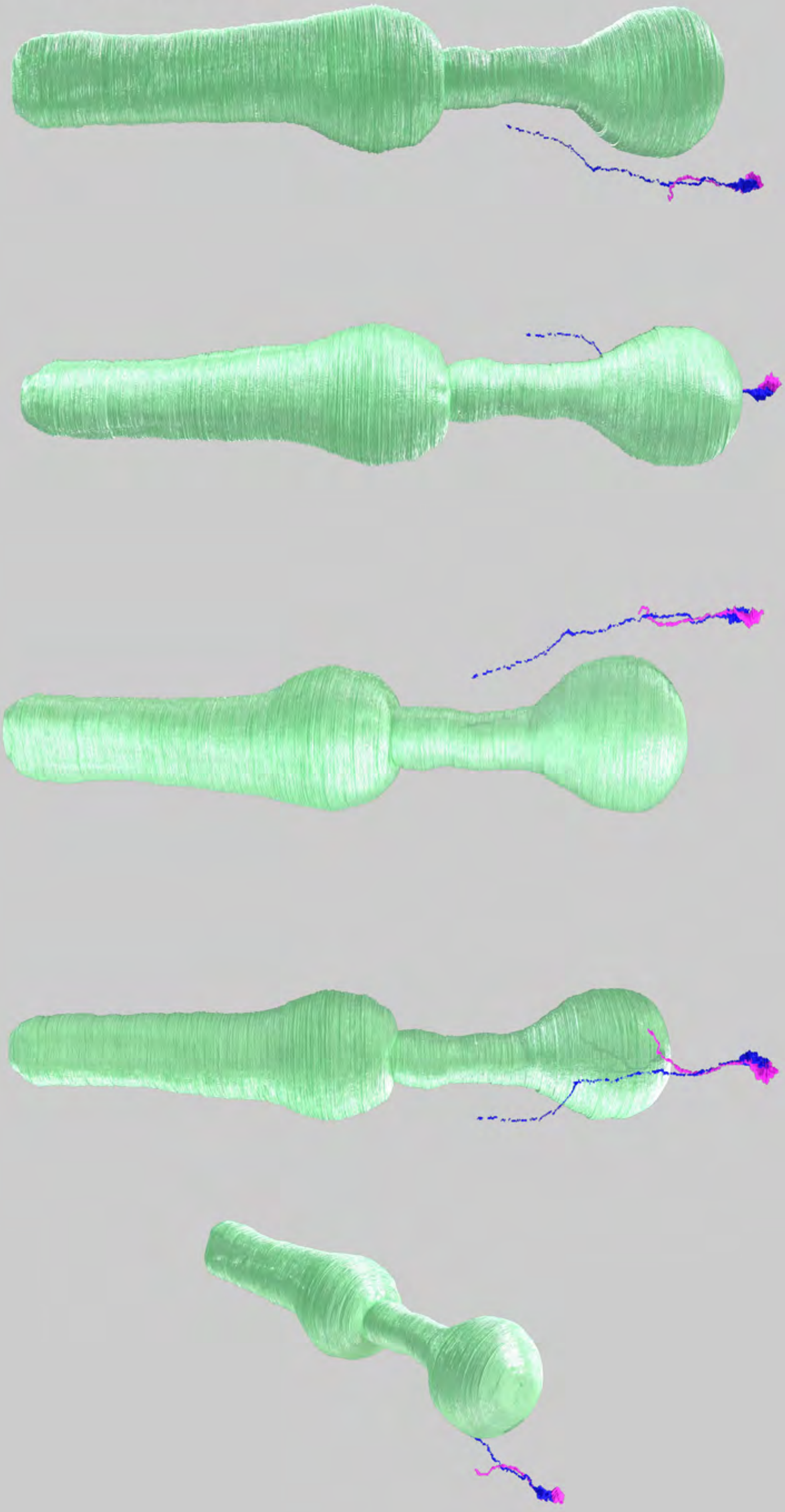

SDQL, SDQR

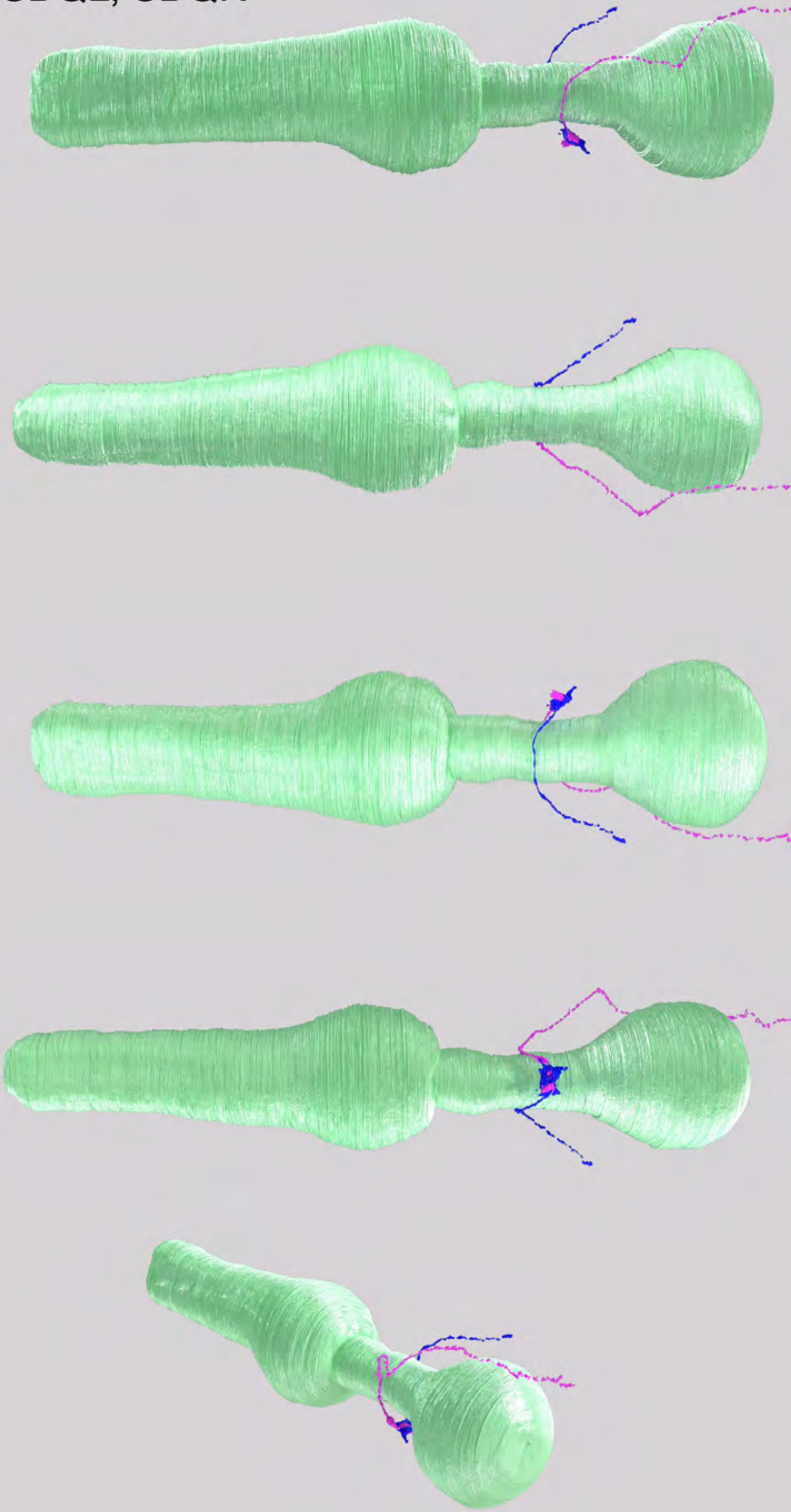

SIADL, SIADR

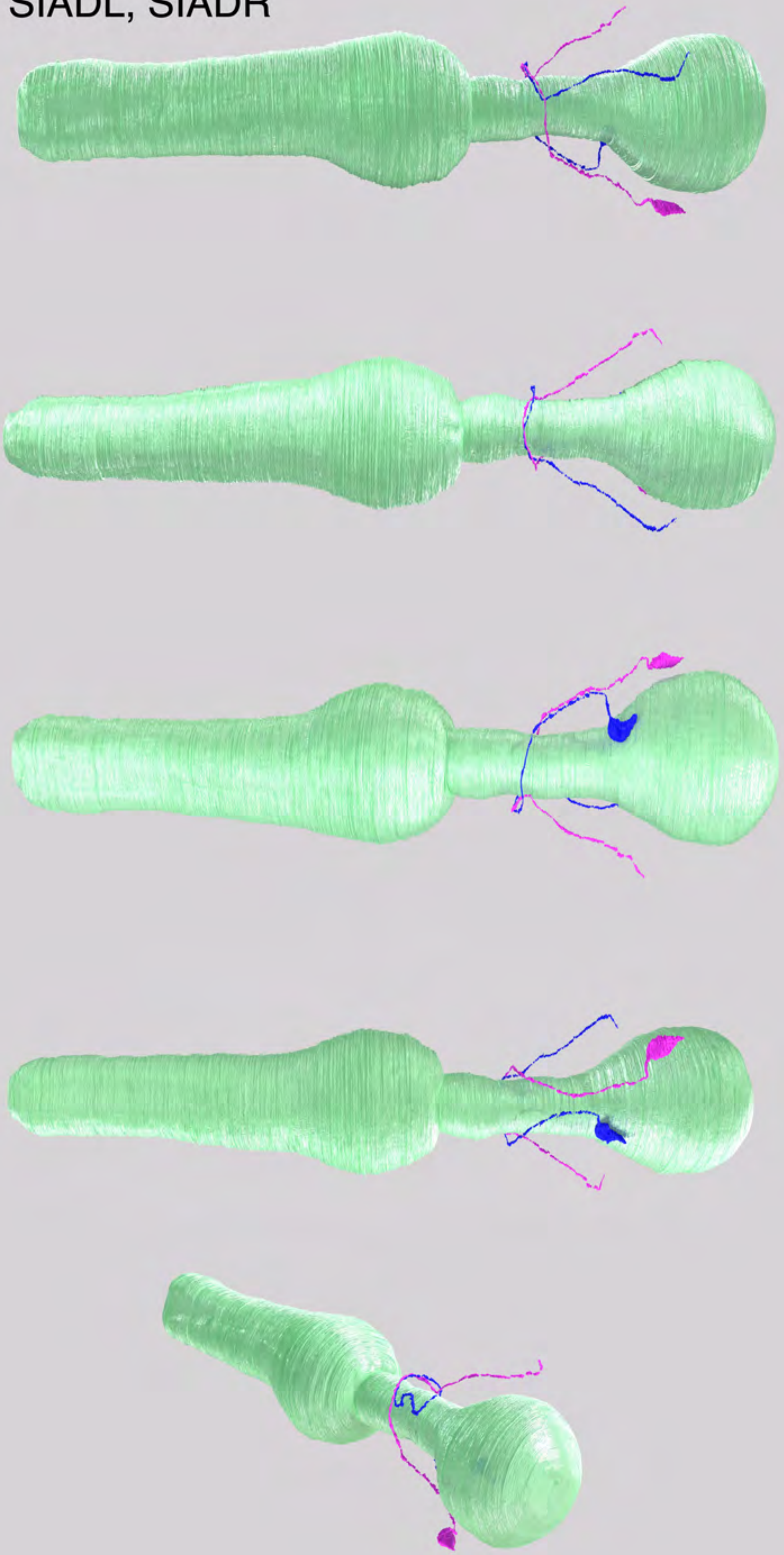

SIAVL, SIAVR

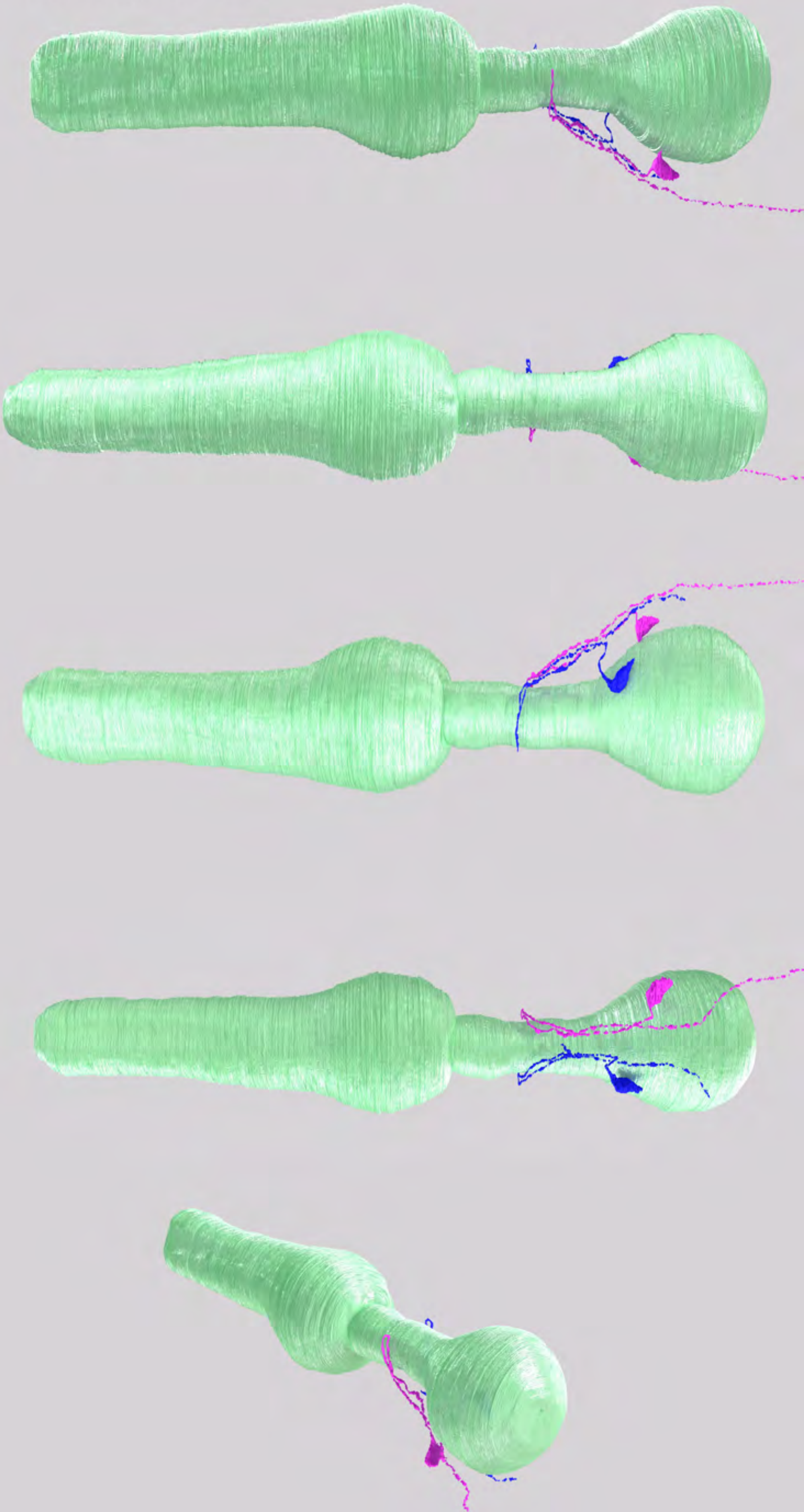

SIBDL, SIBDR

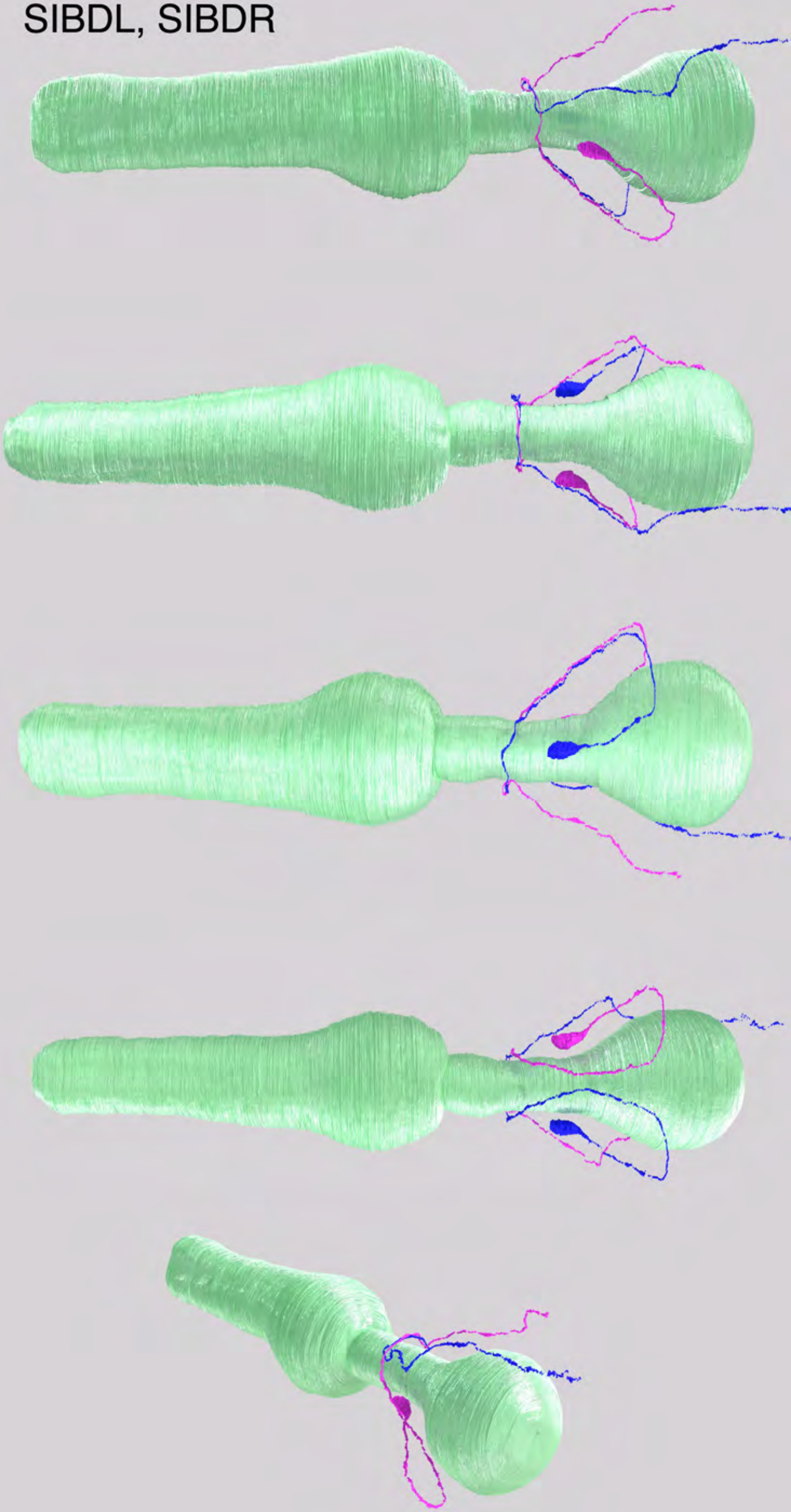

SIBVL, SIBVR

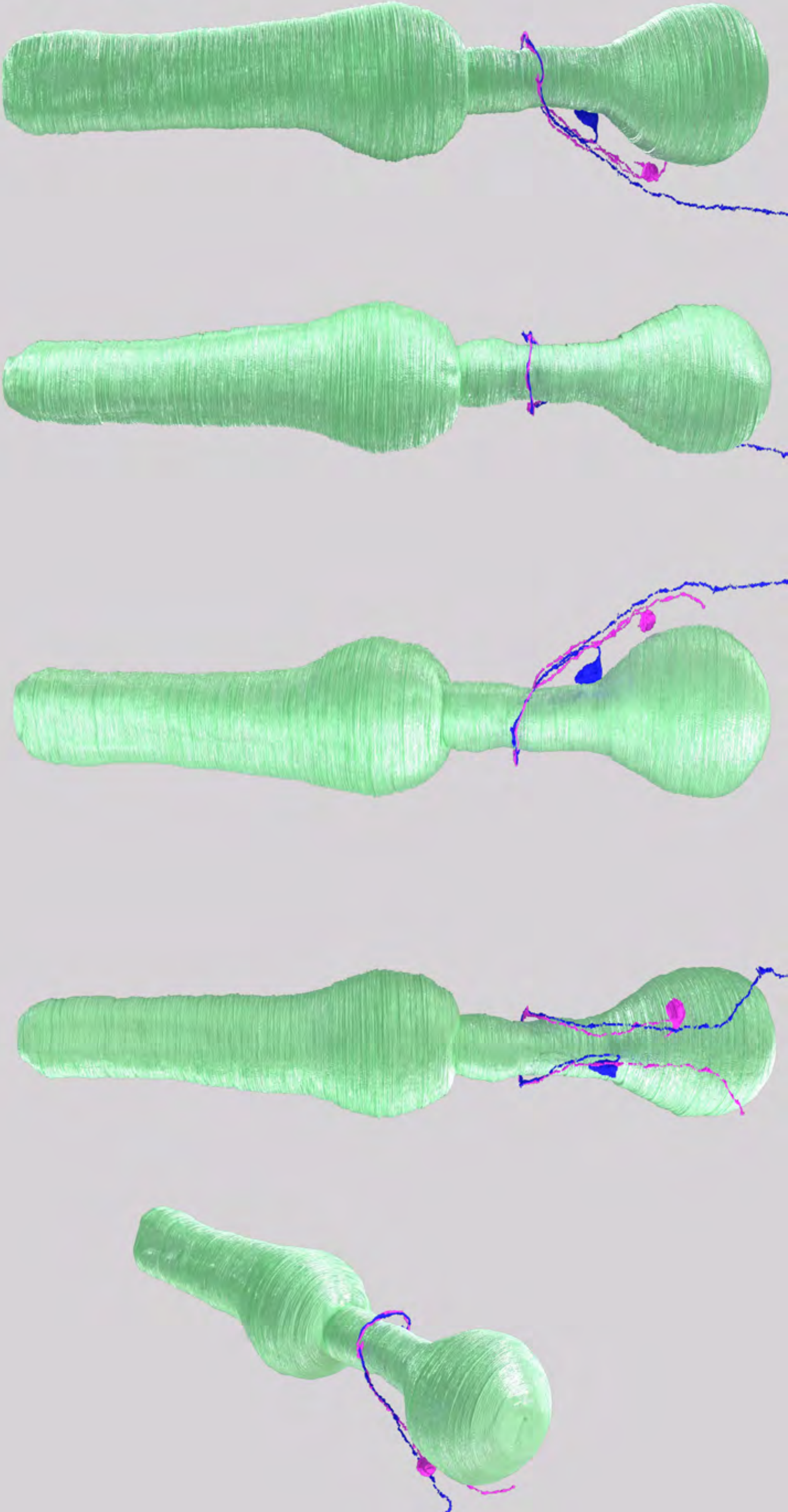

SMBDL, SMBDR

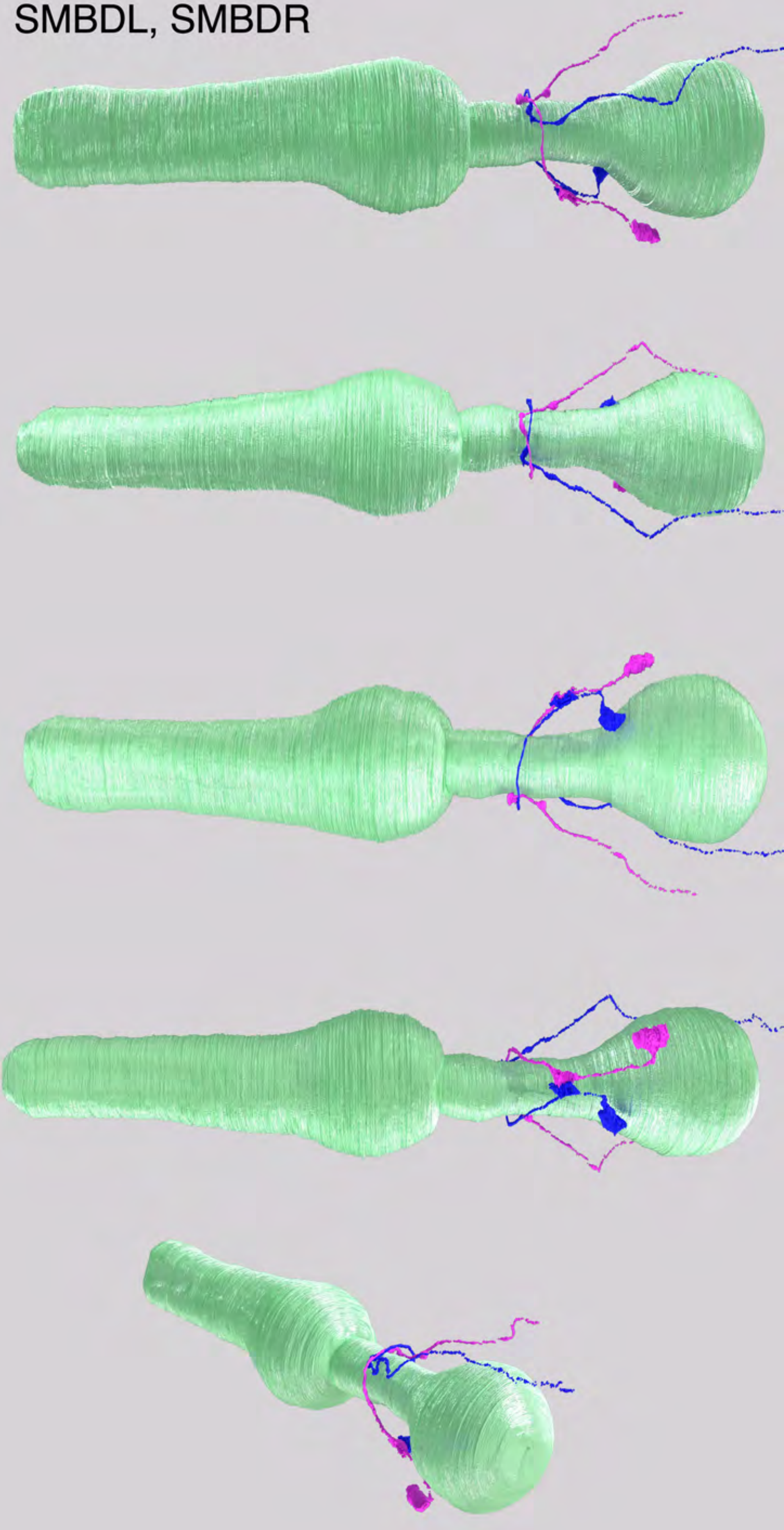

SMBVL, SMBVR

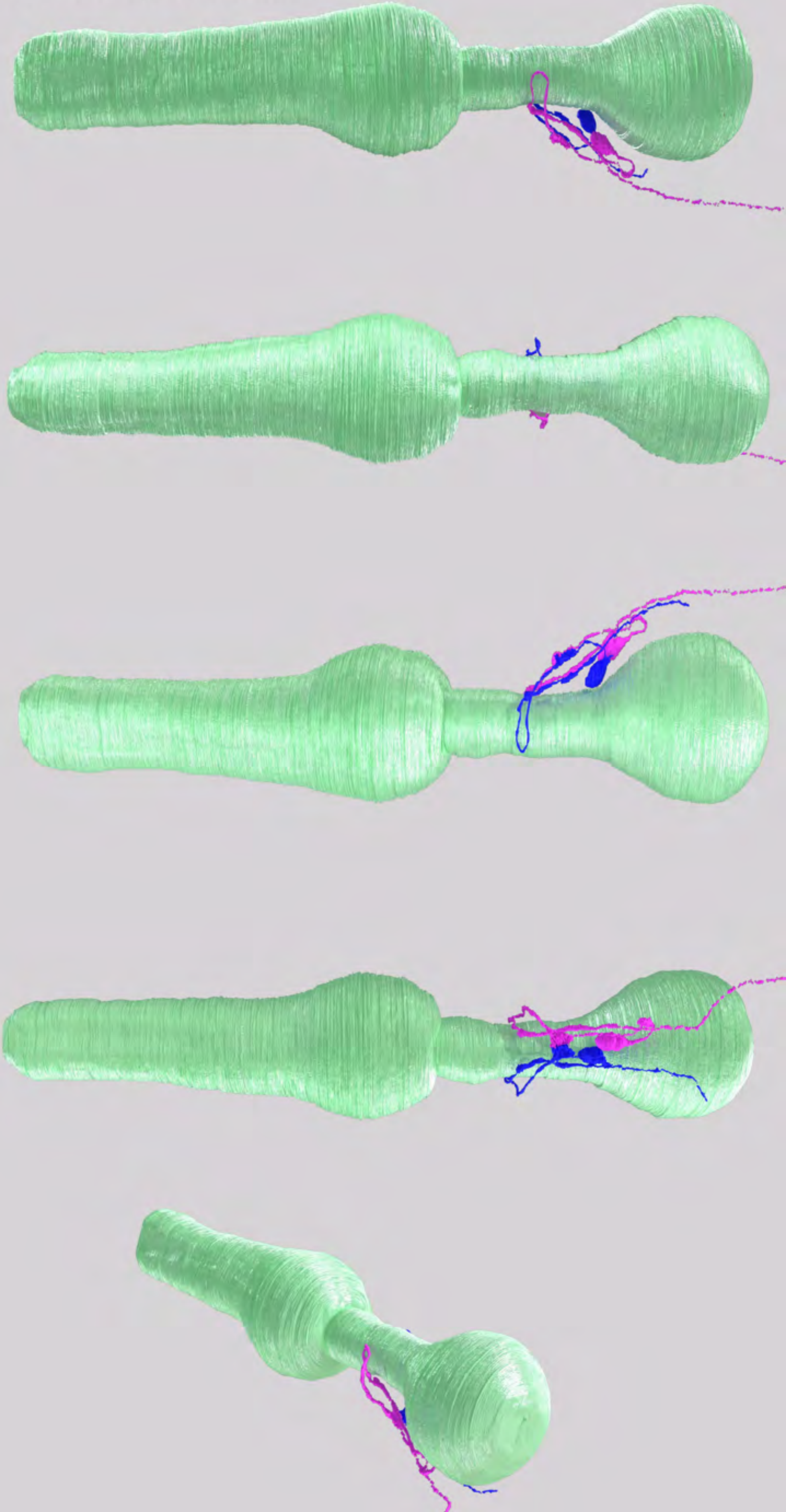

SMDDL, SMDDR

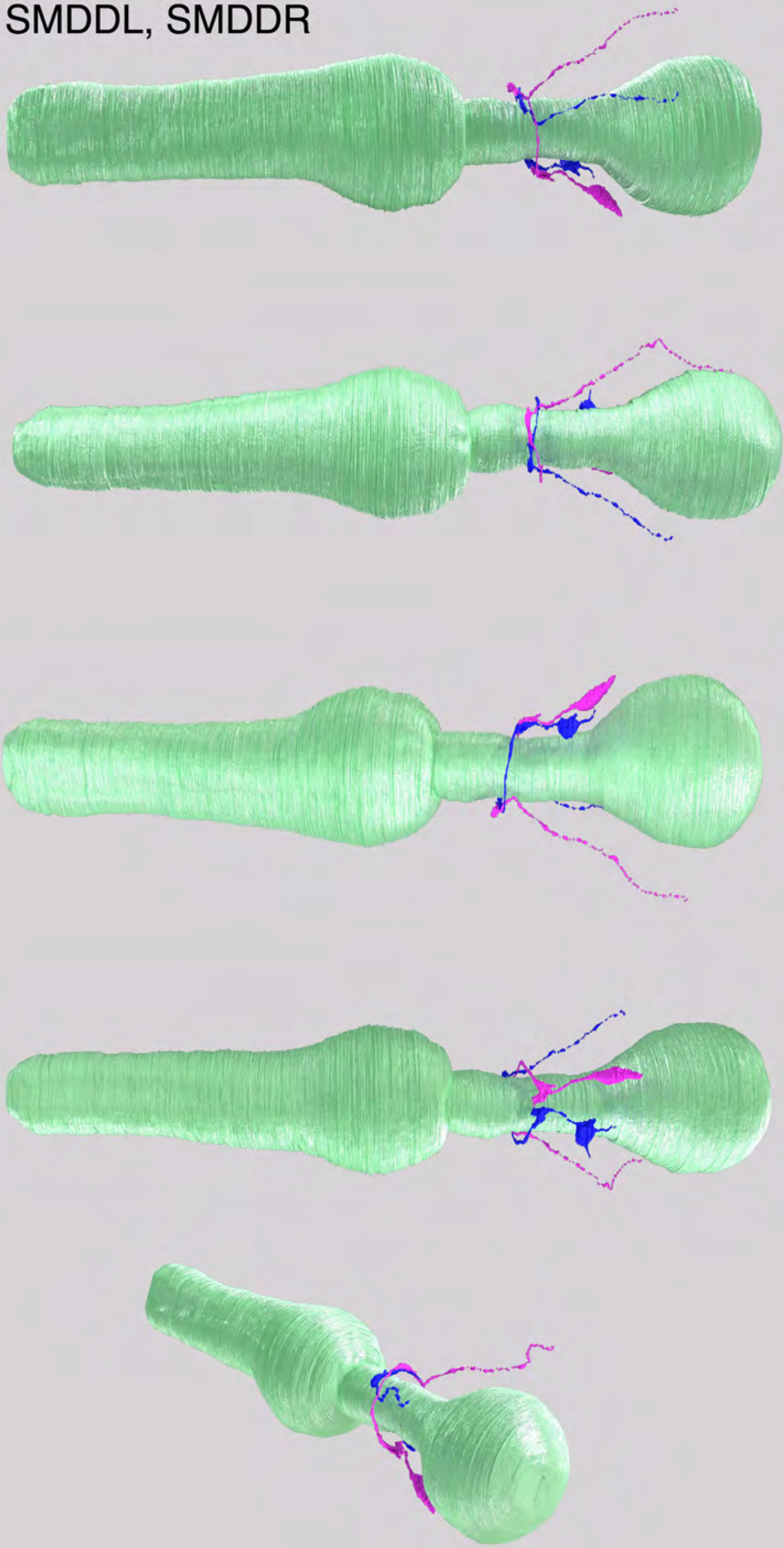

SMDVL, SMDVR

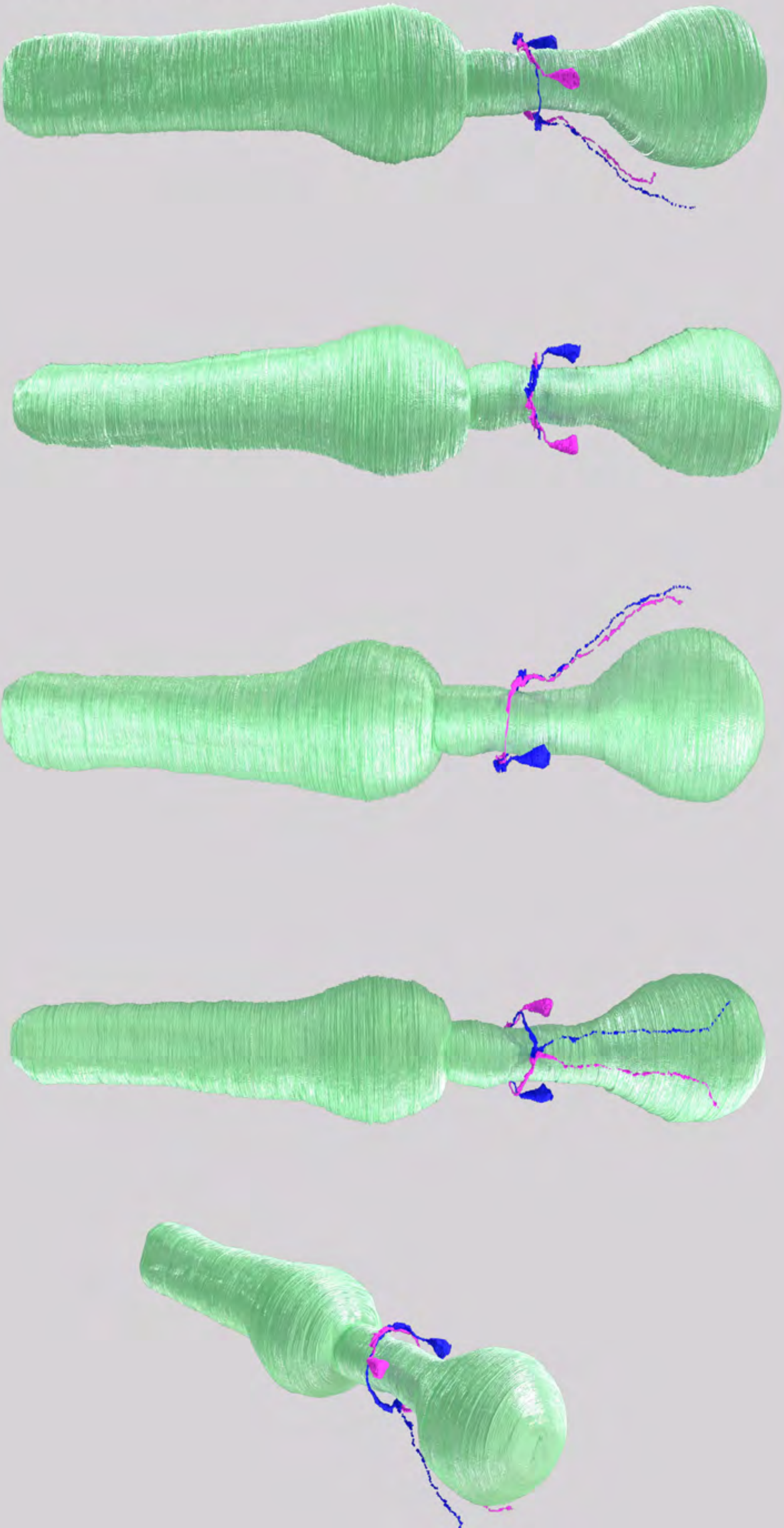

URADL, URADR

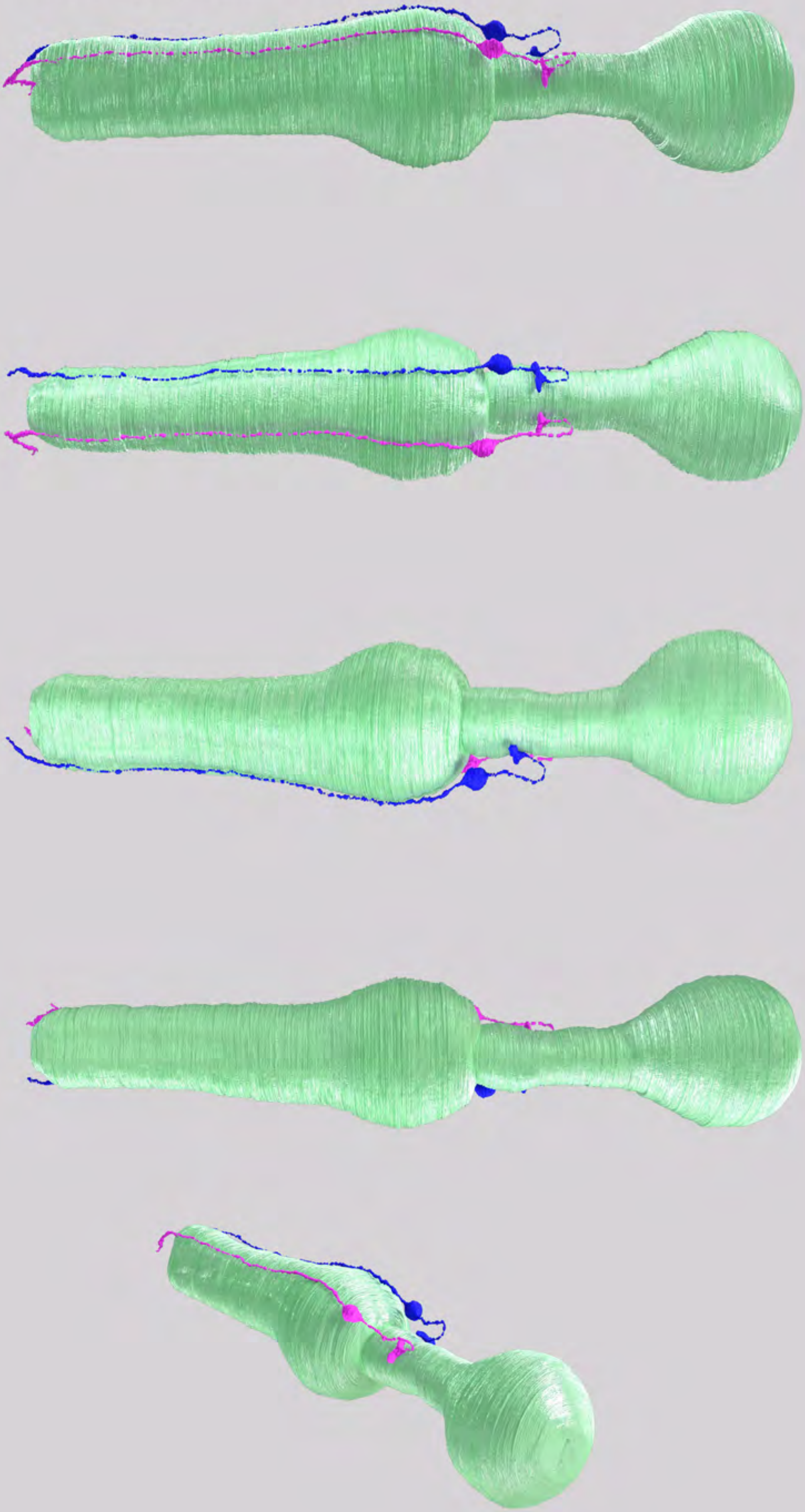

URAVL, URAVR

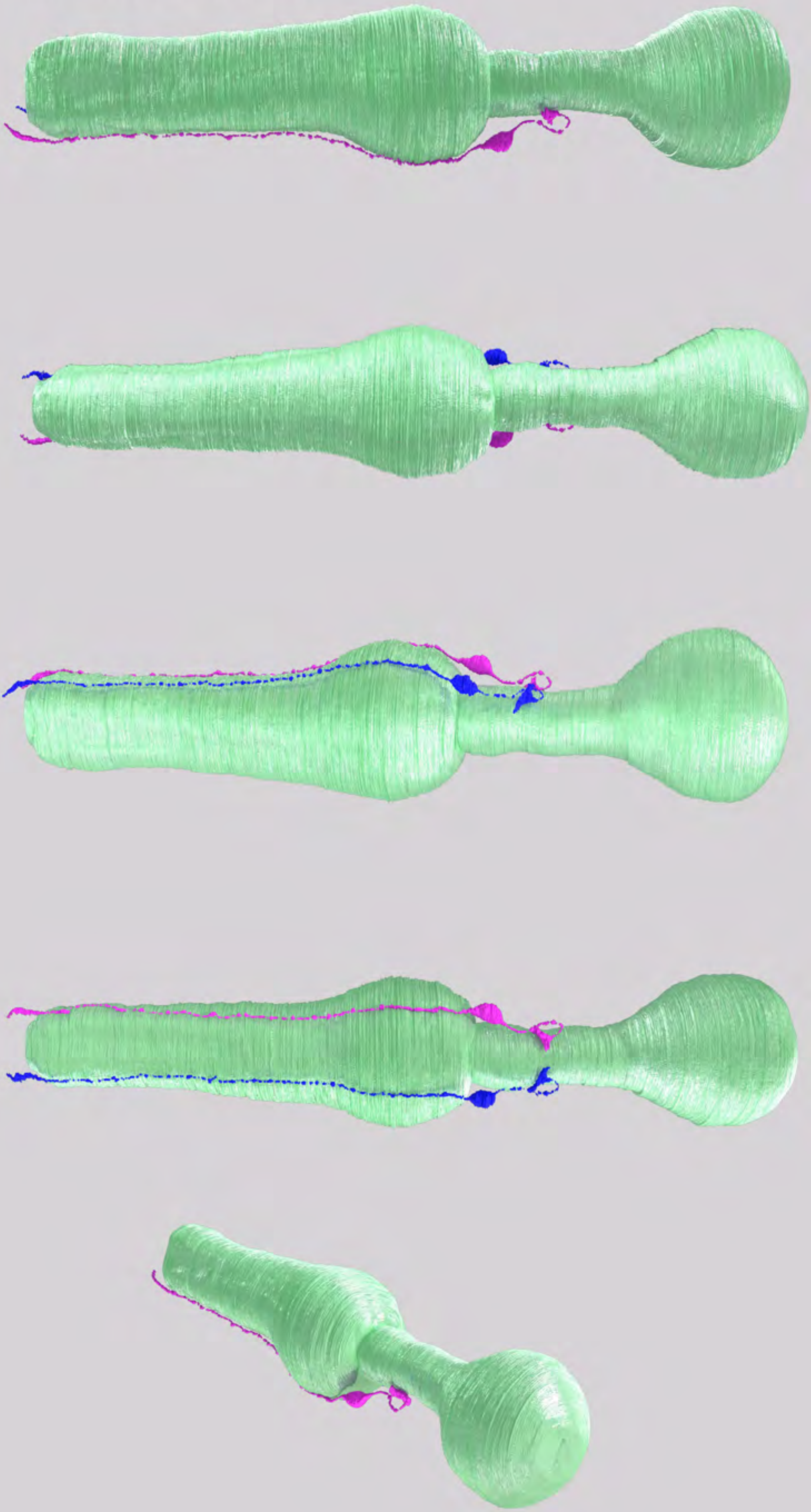

URBL, URBR

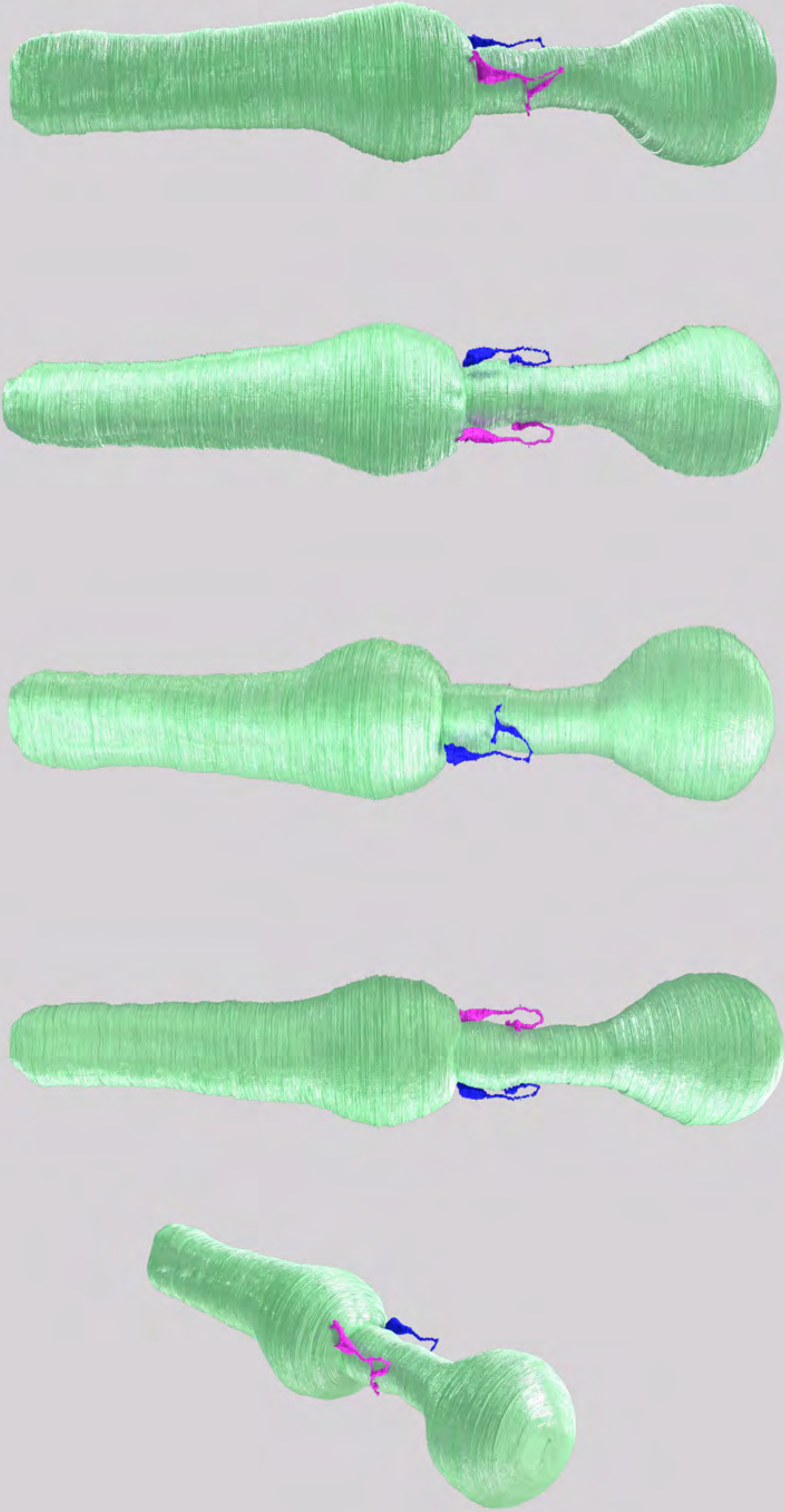

URXL, URXR

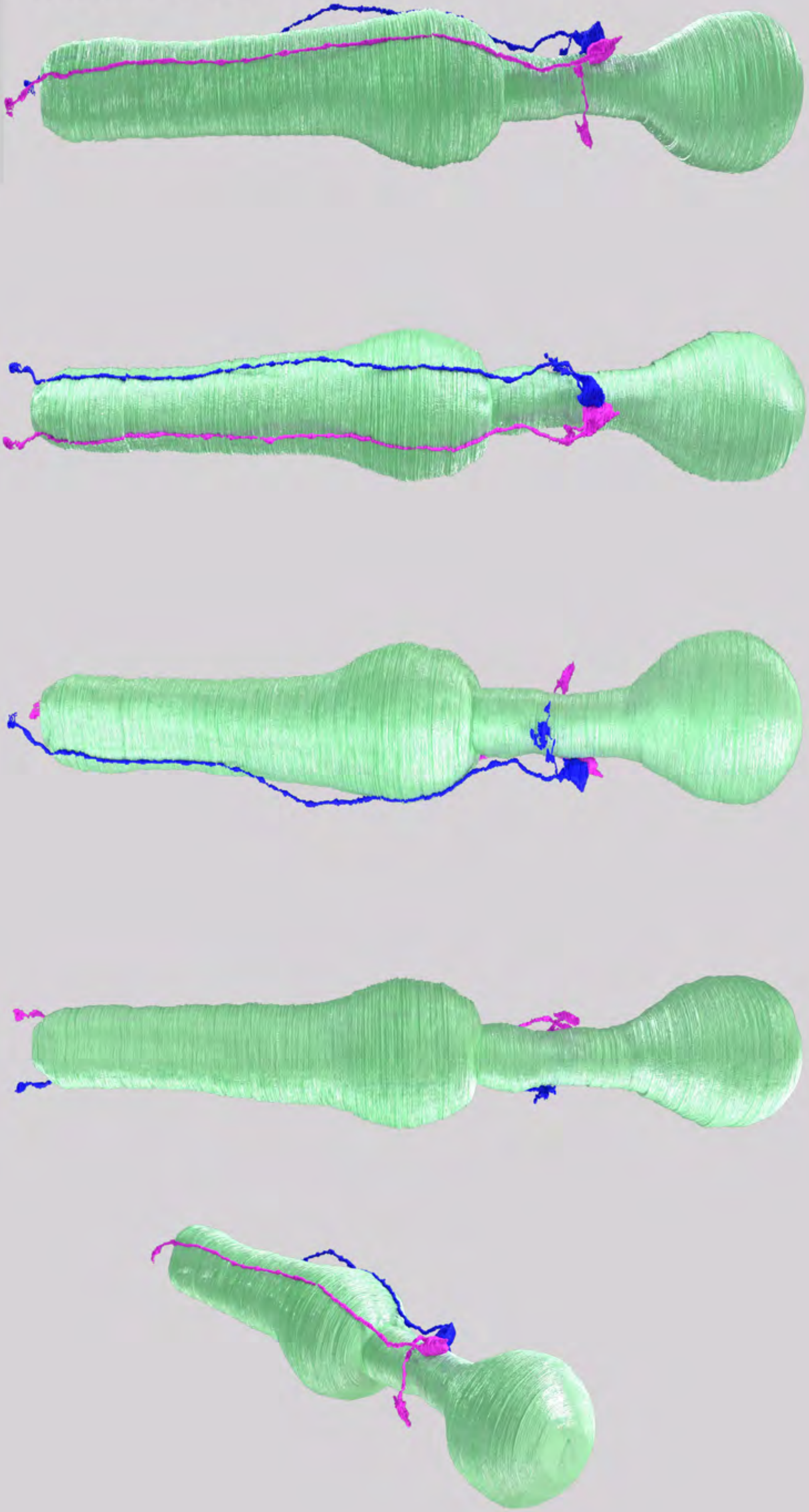

URYDL, URYDR

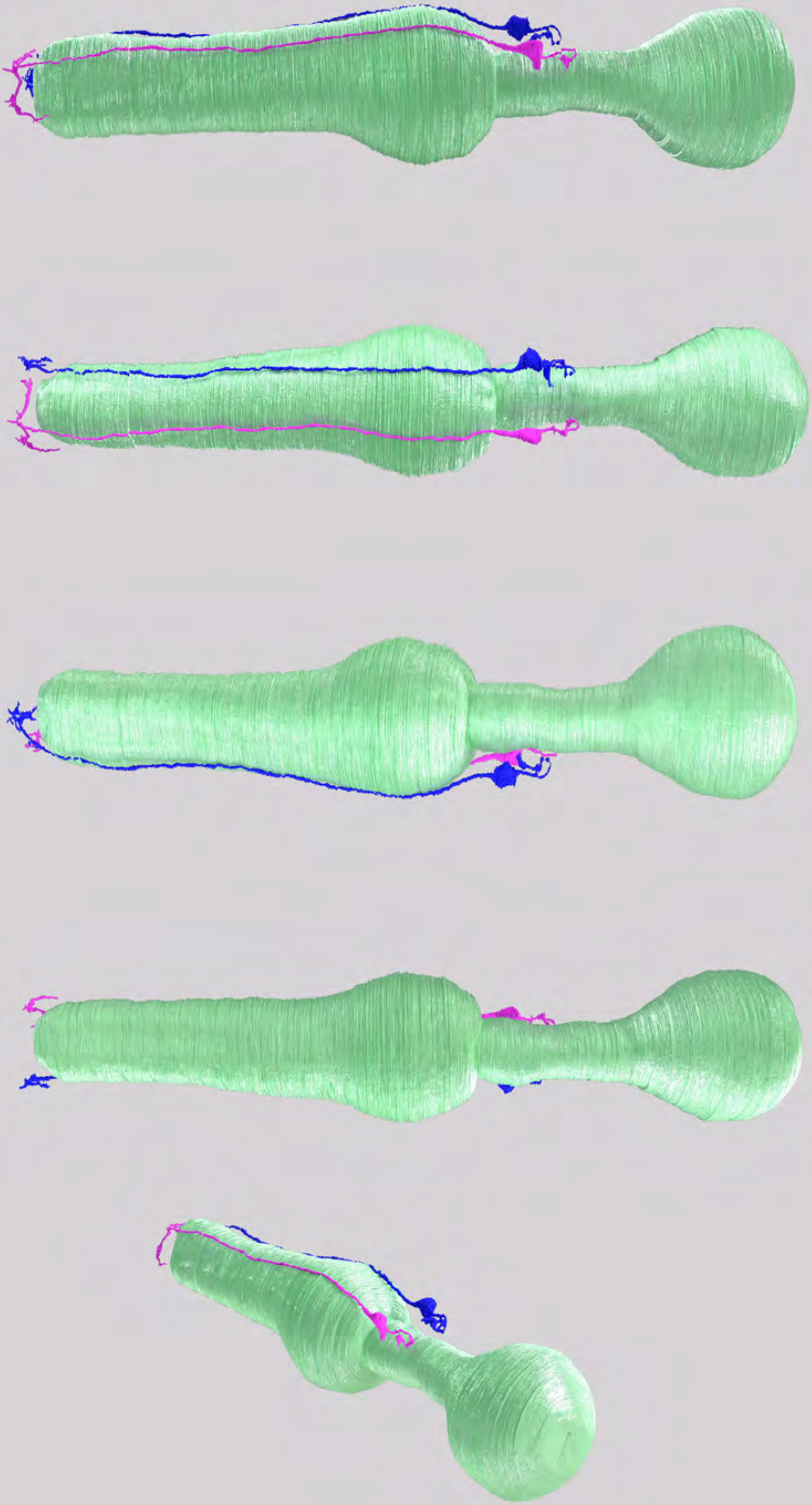

URYVL, URYVR

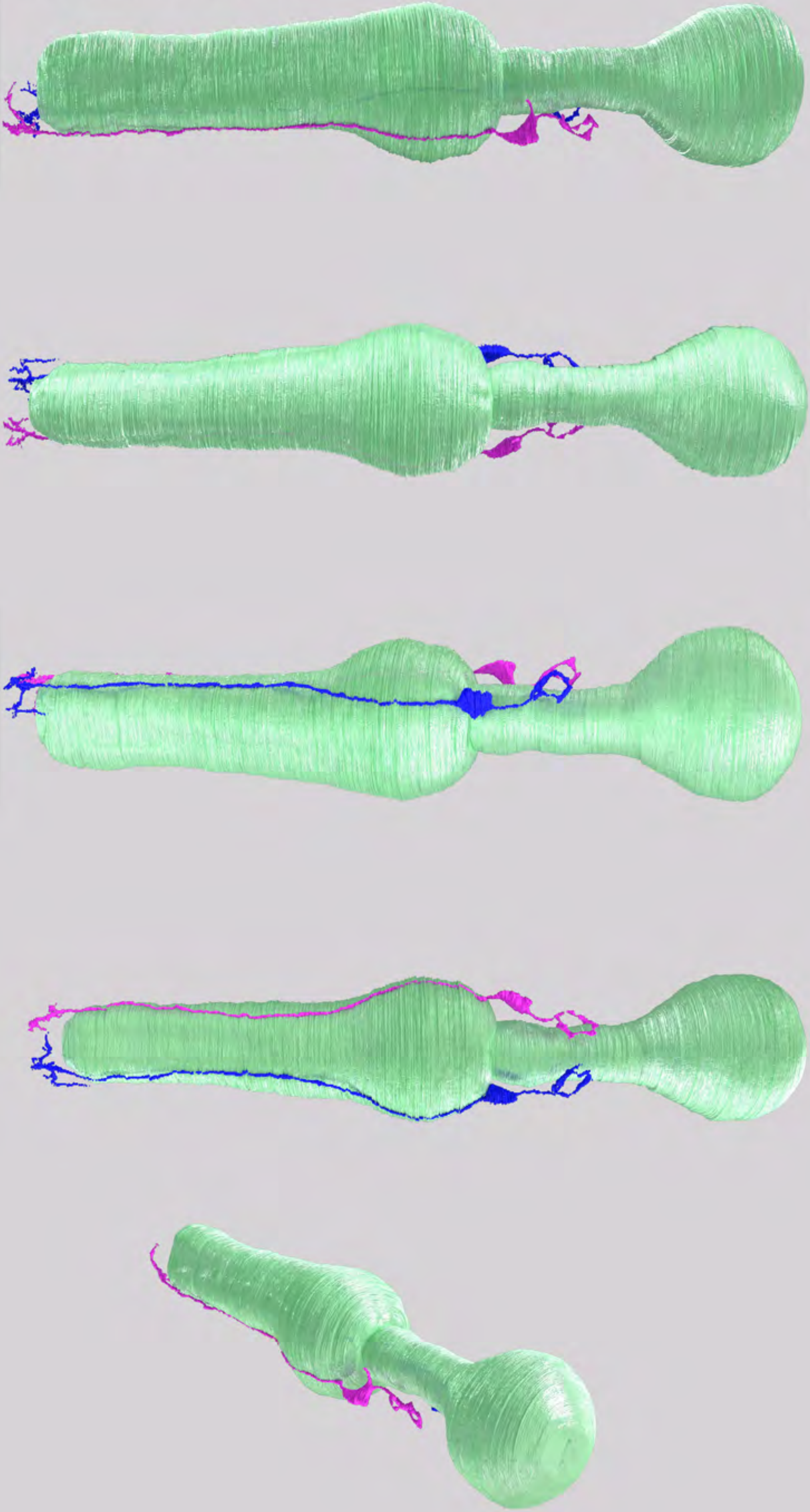

VB1

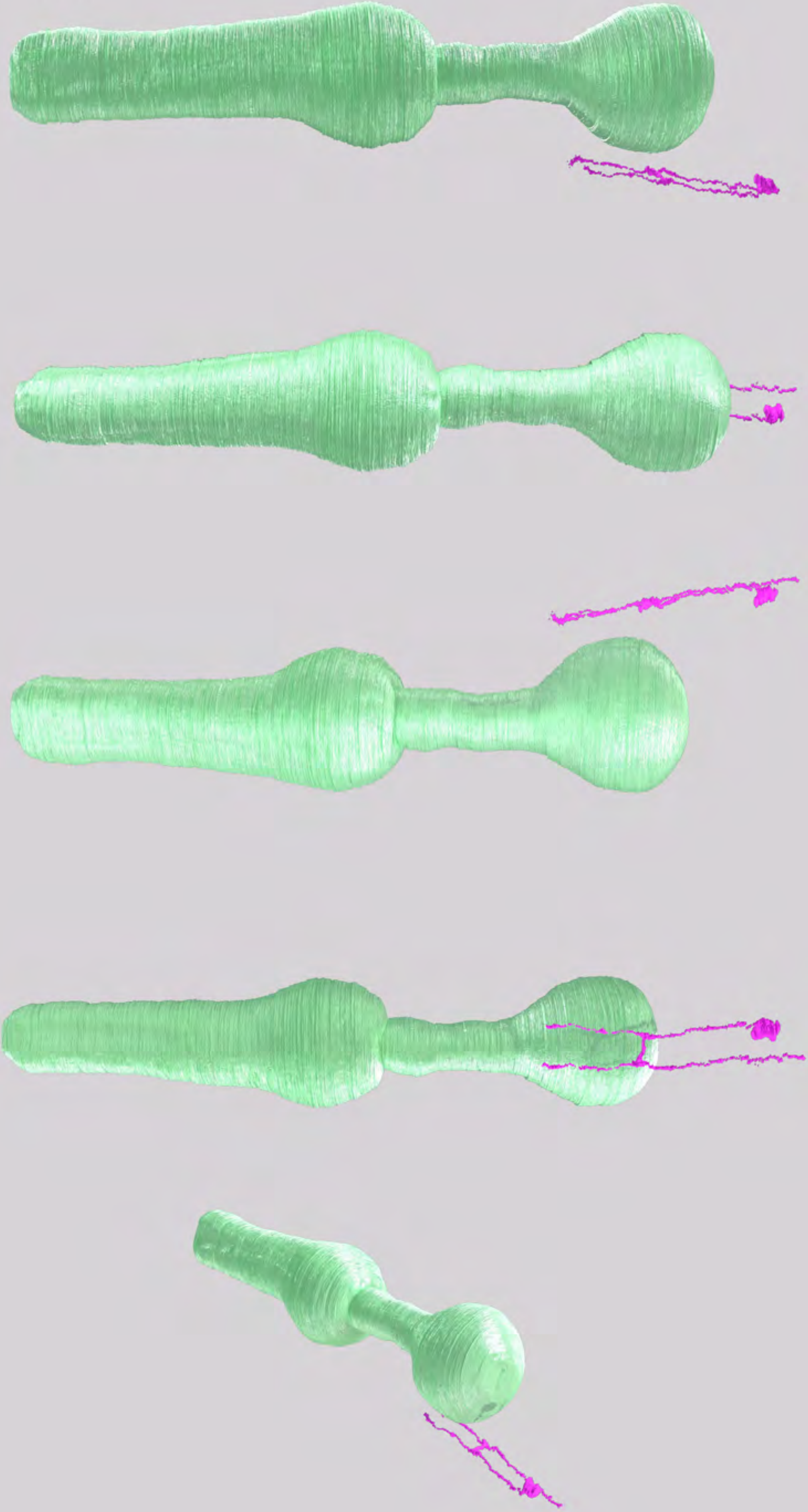

VB2

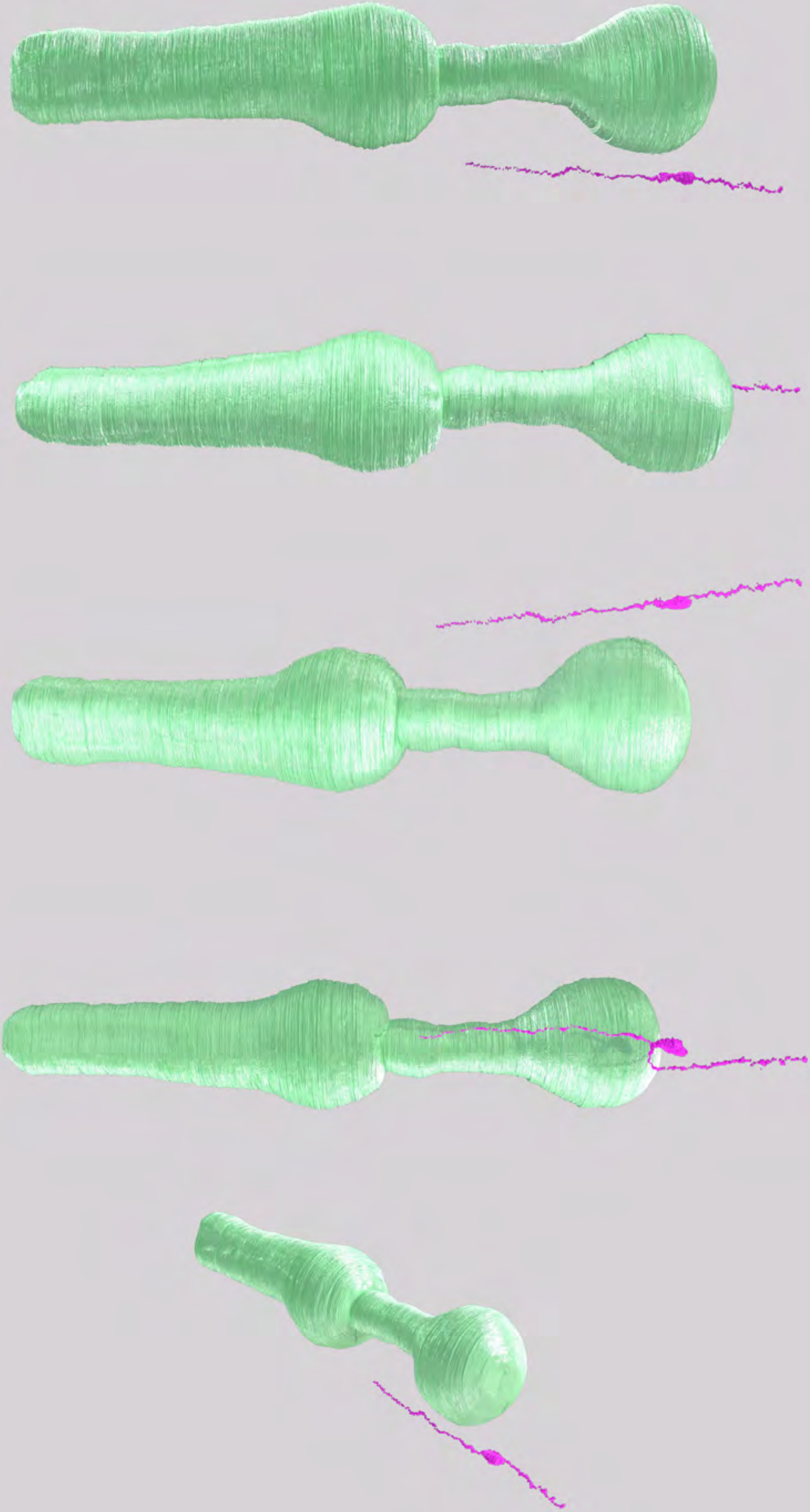

I1L, I1R

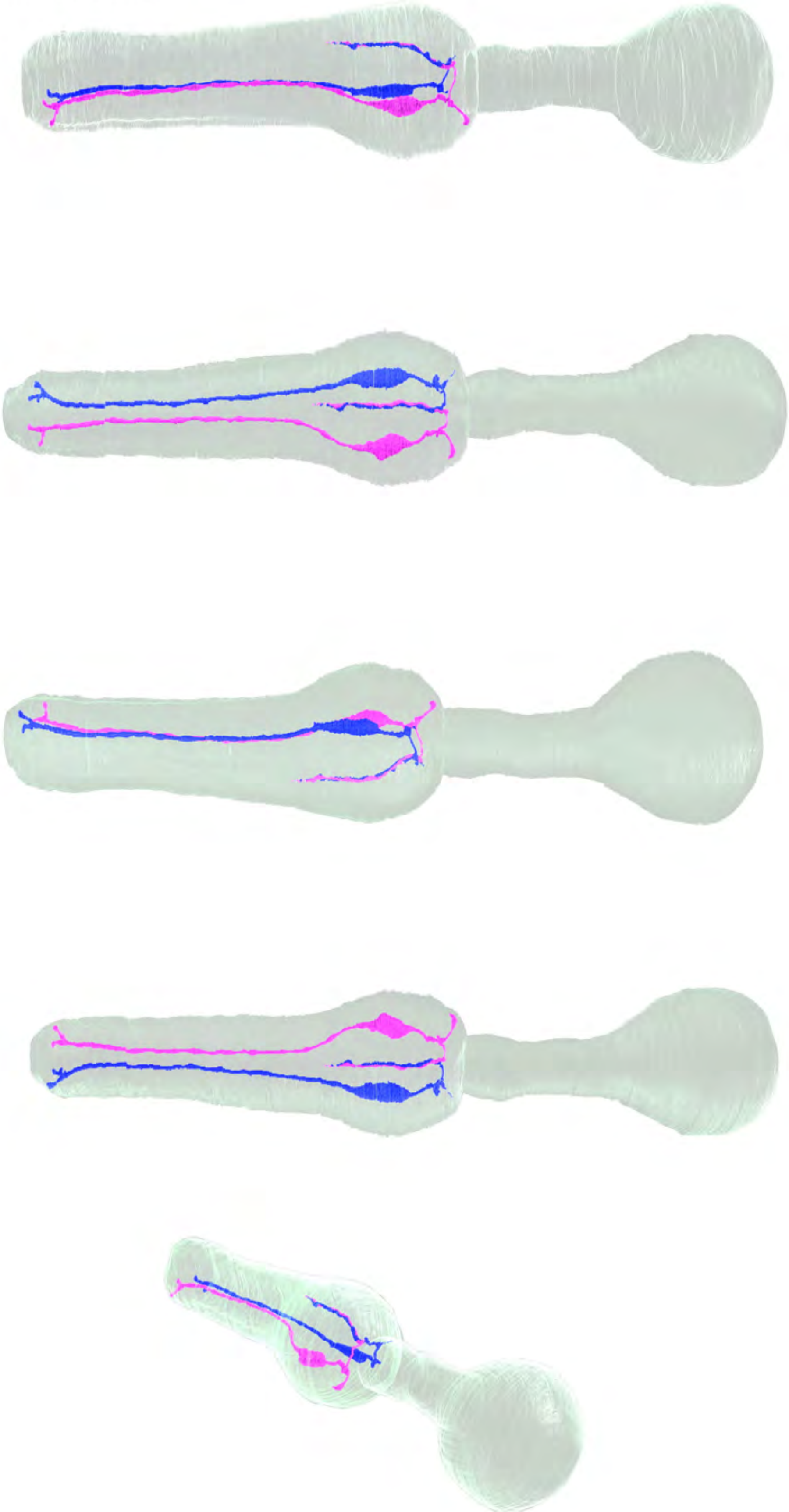

I2L, I2R

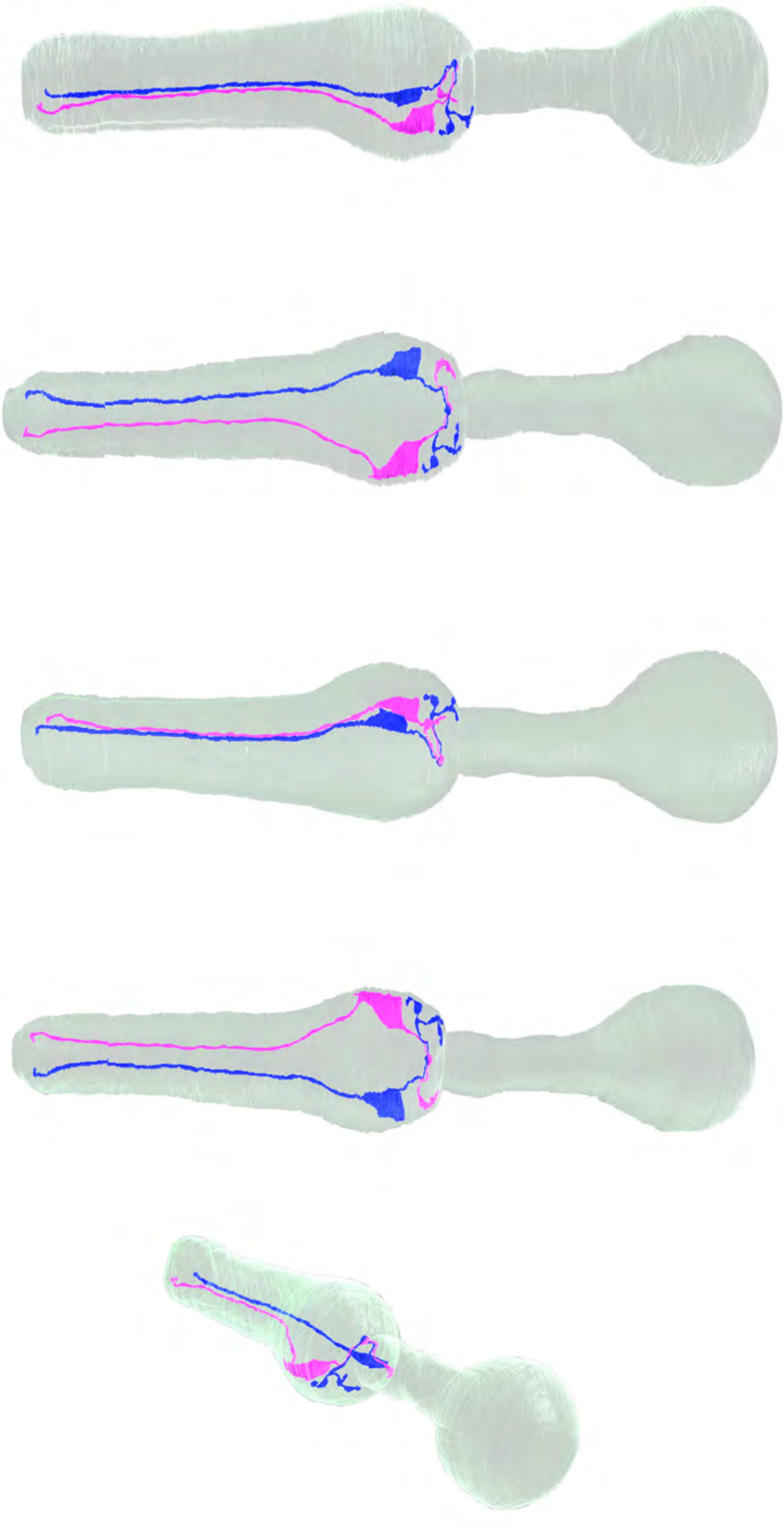

I3

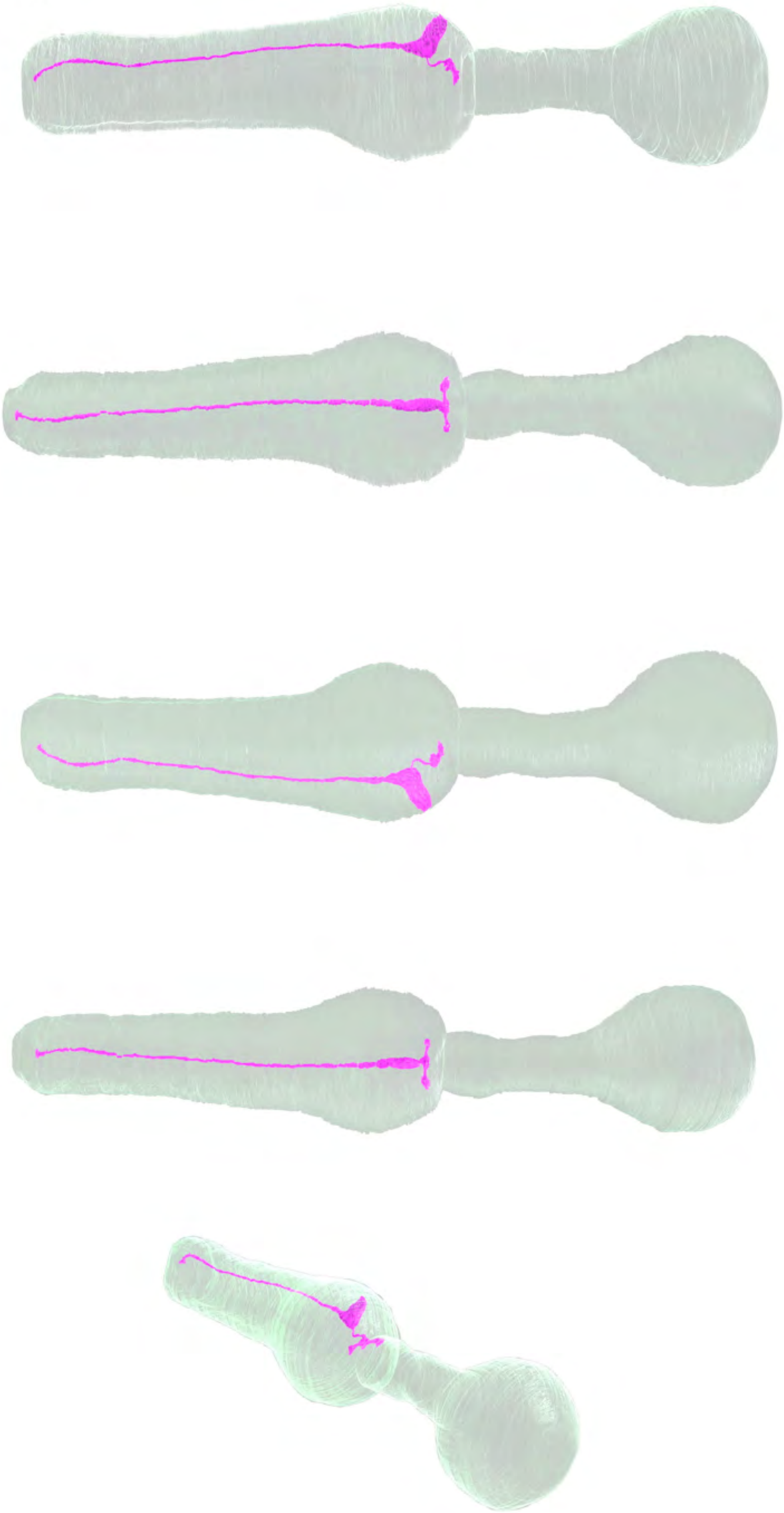

I4

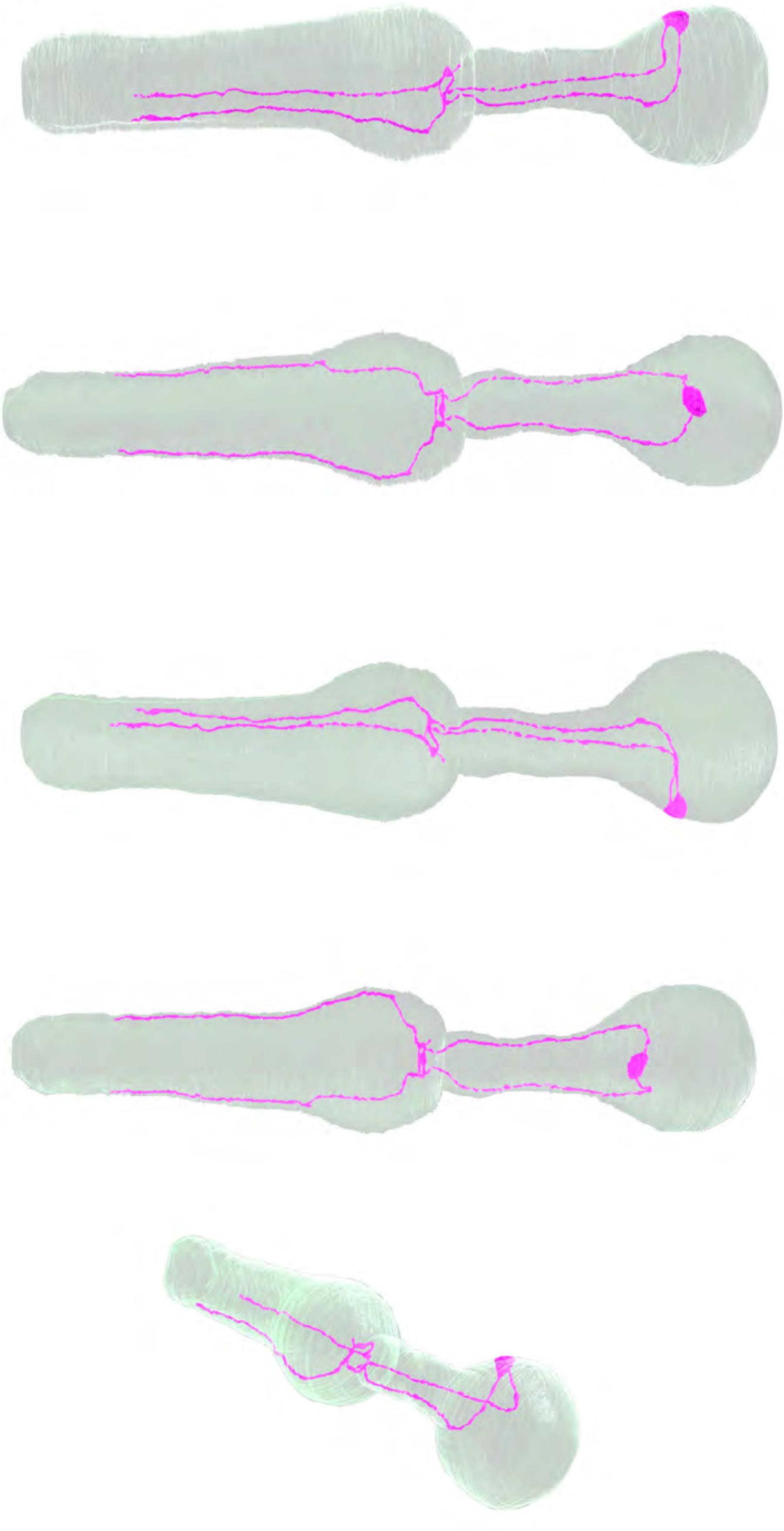

I5

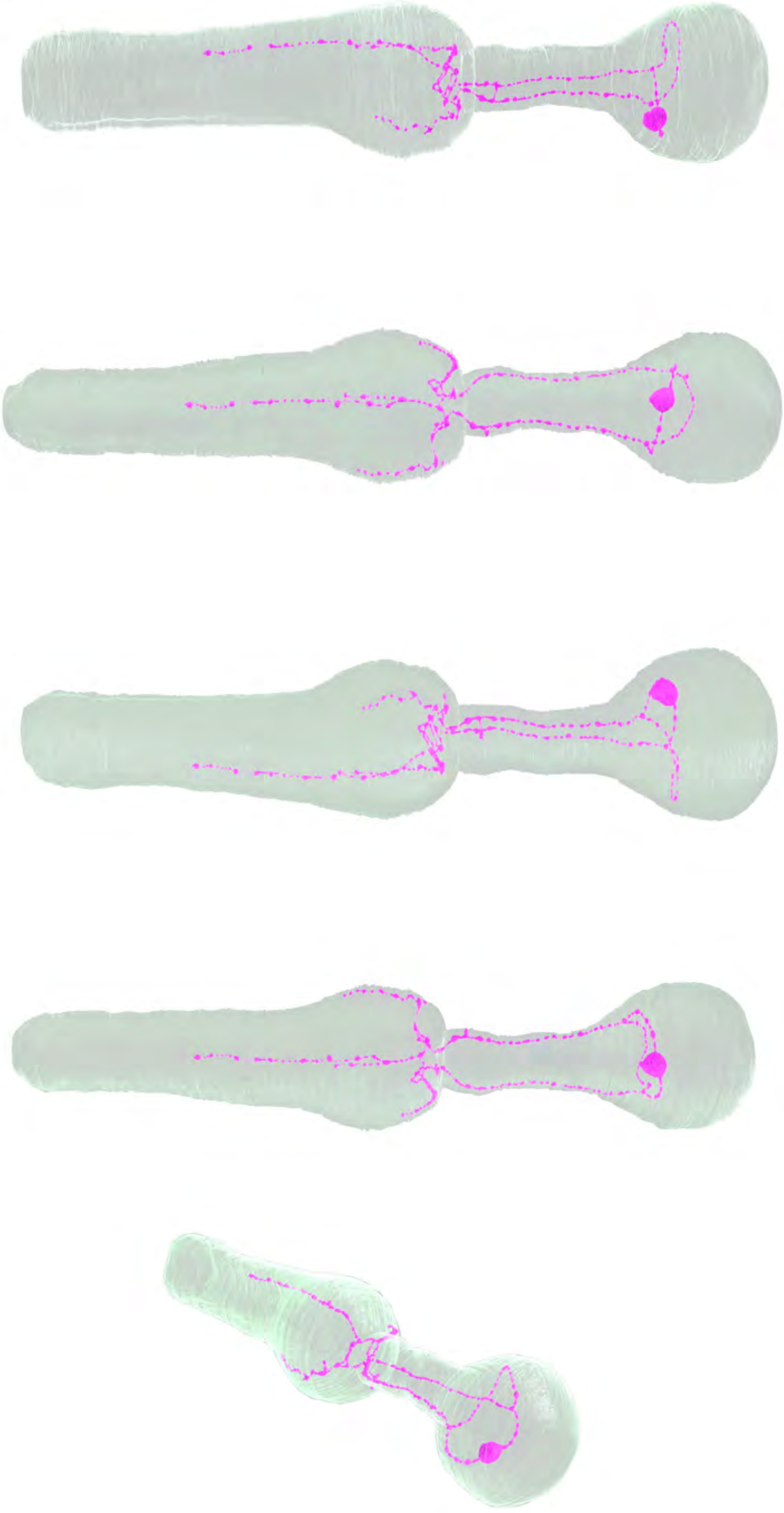

I6

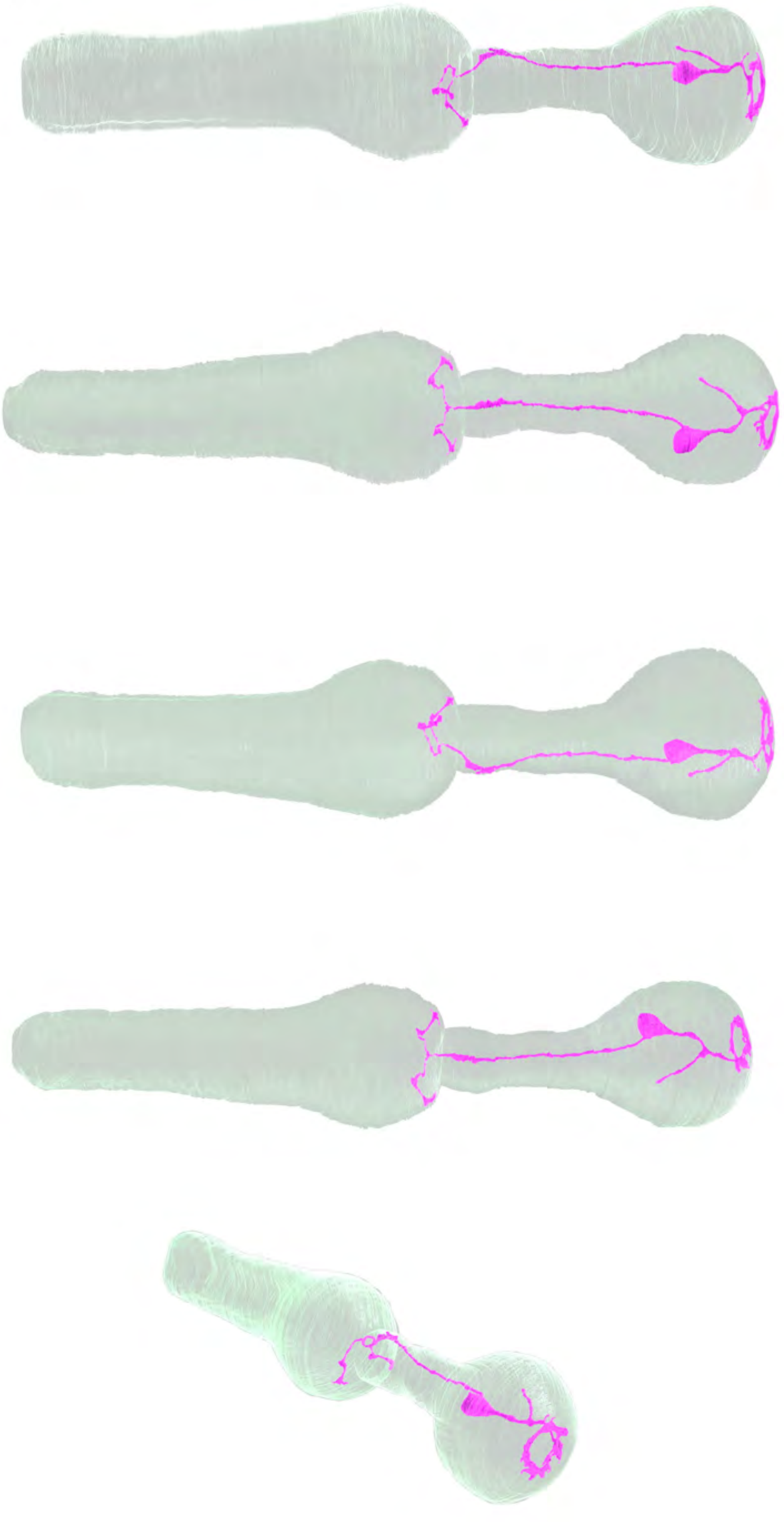

M1

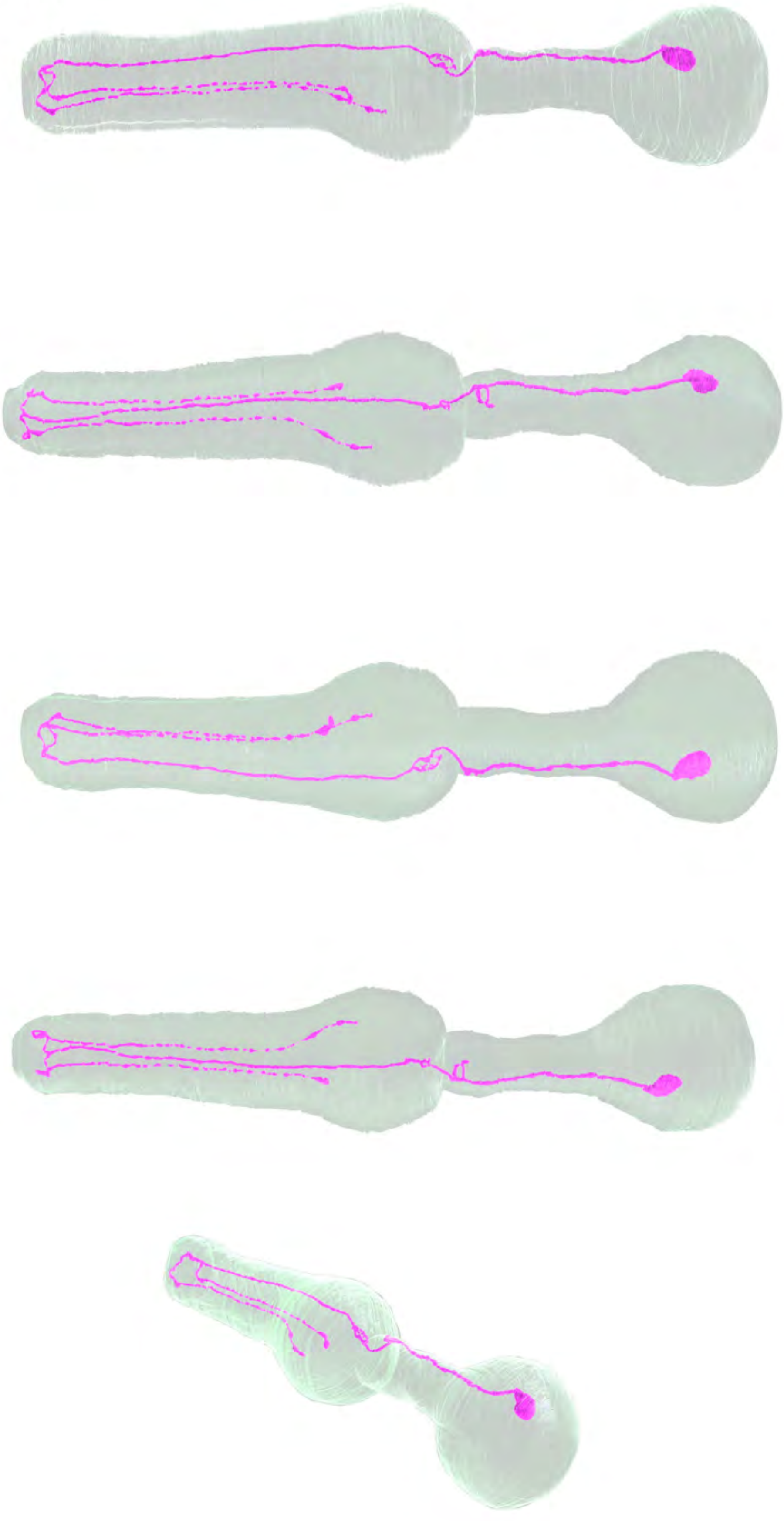

M2L, M2R

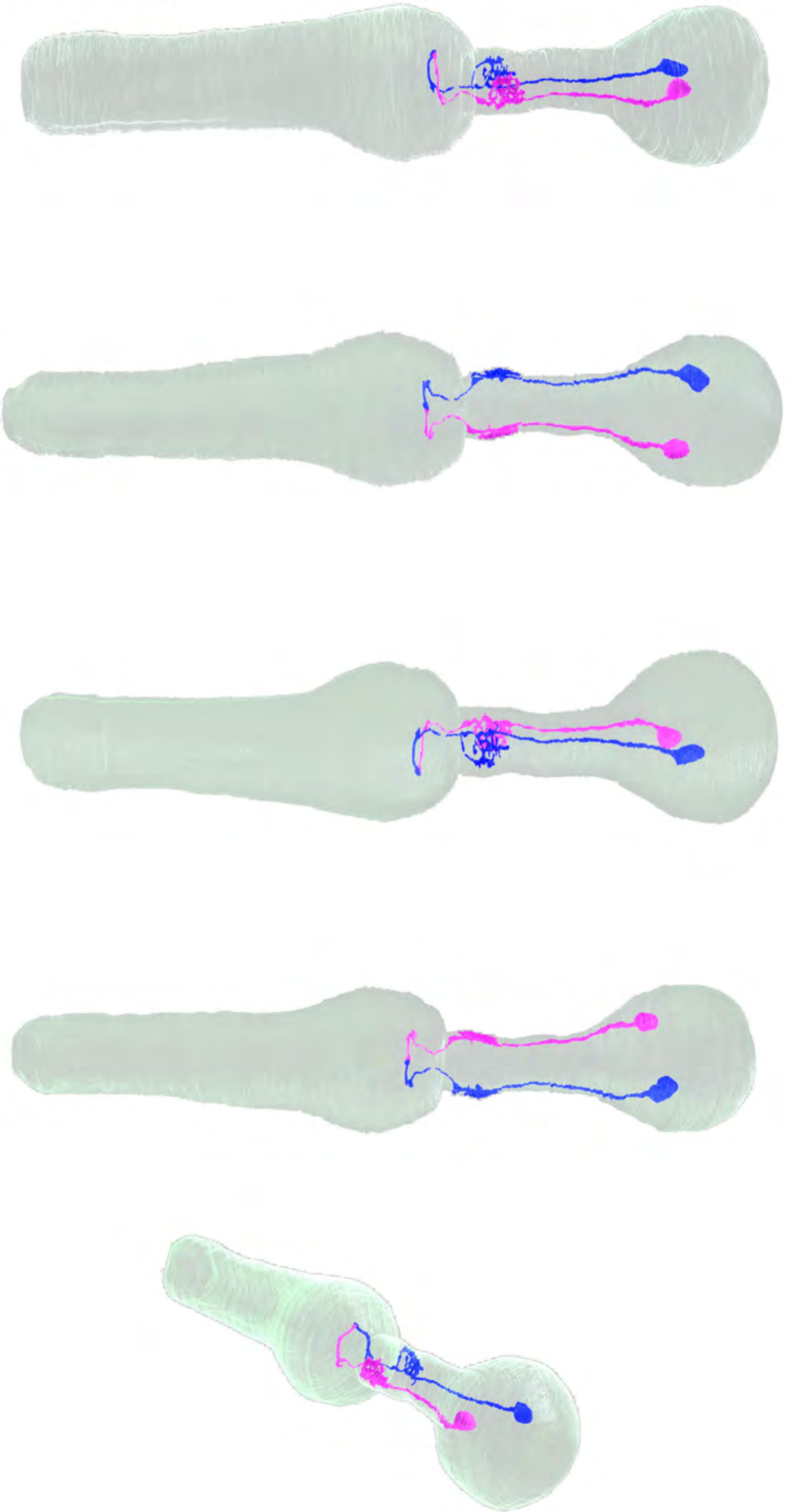

M3L, M3R

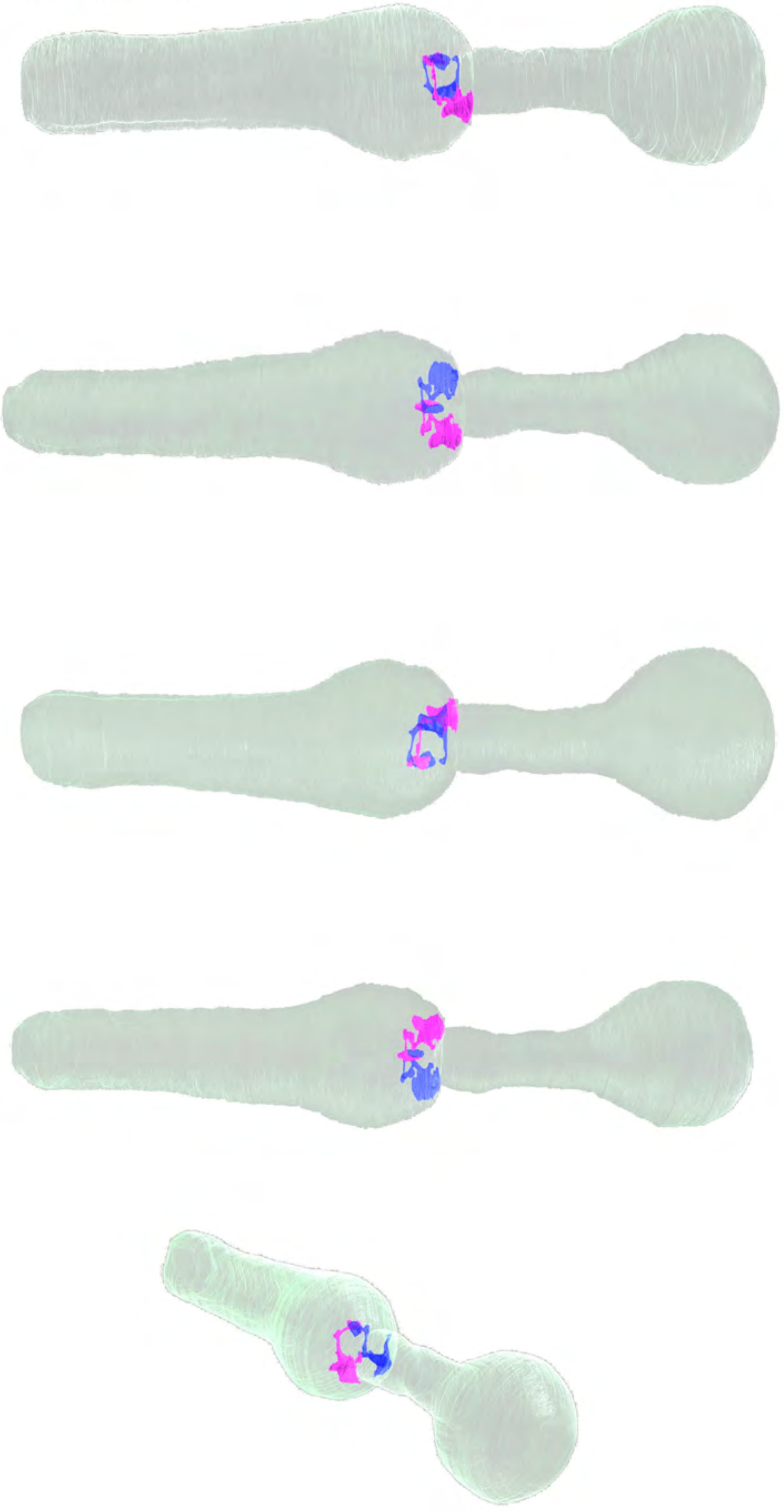

M4

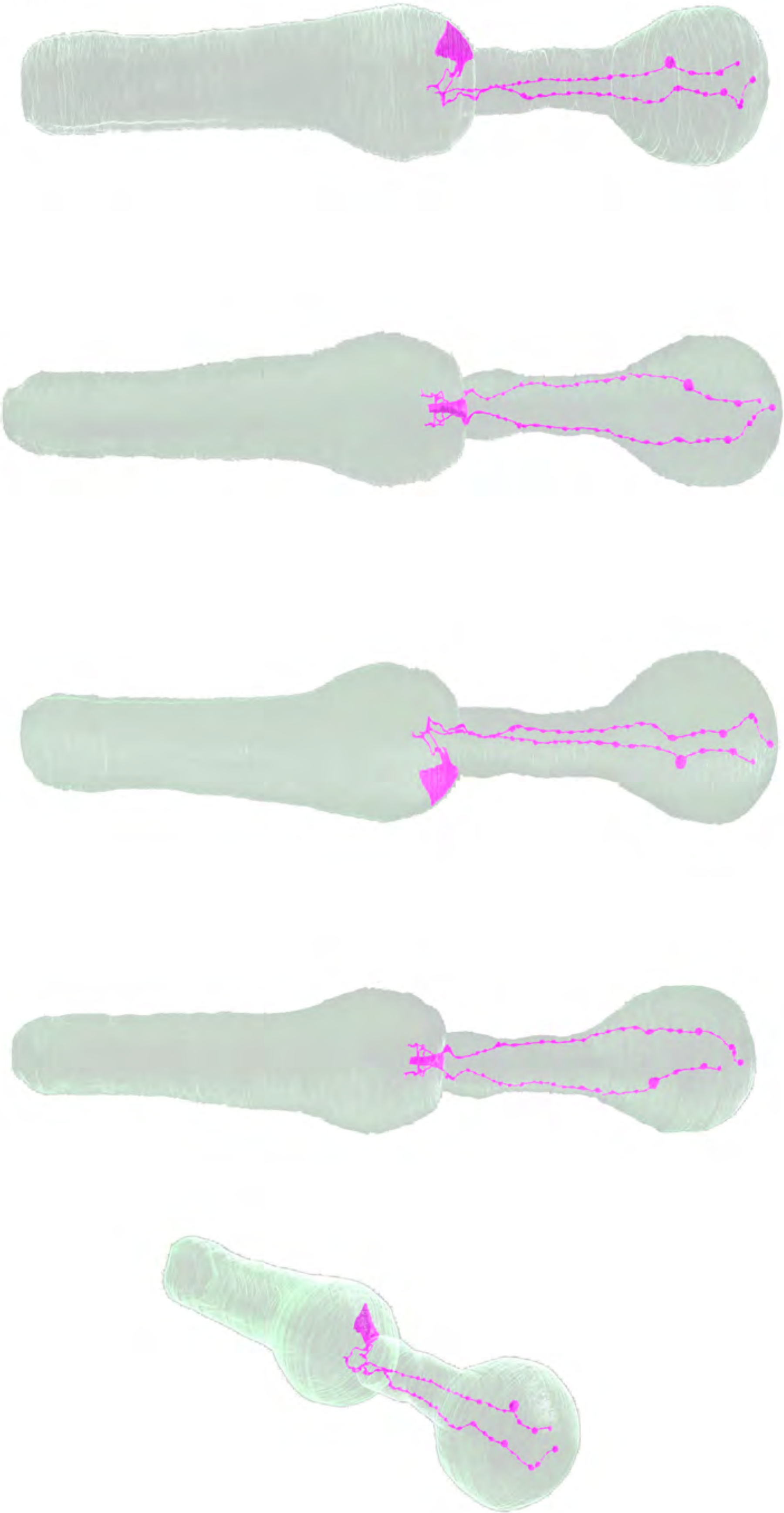

M5

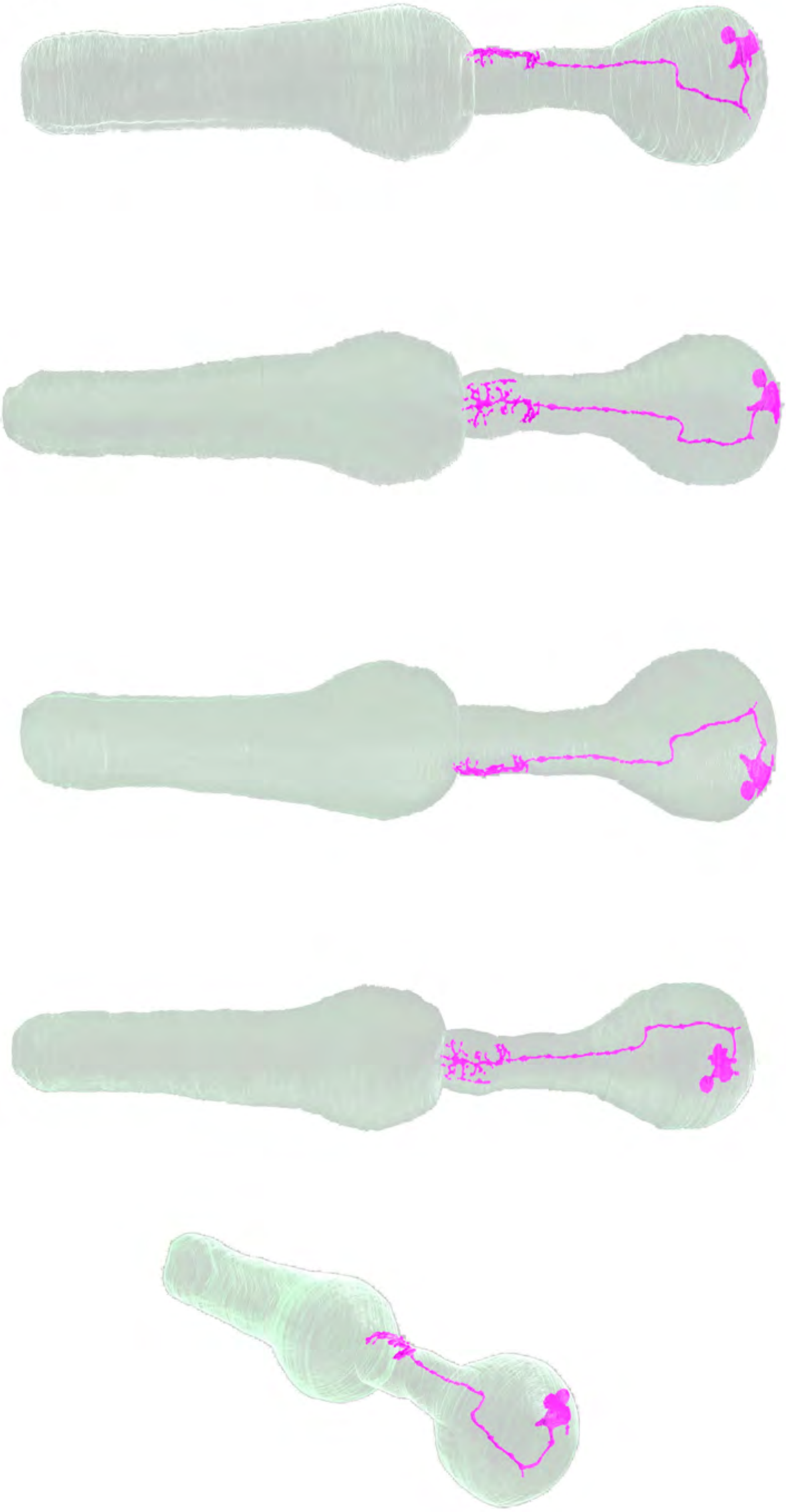

MCL, MCR

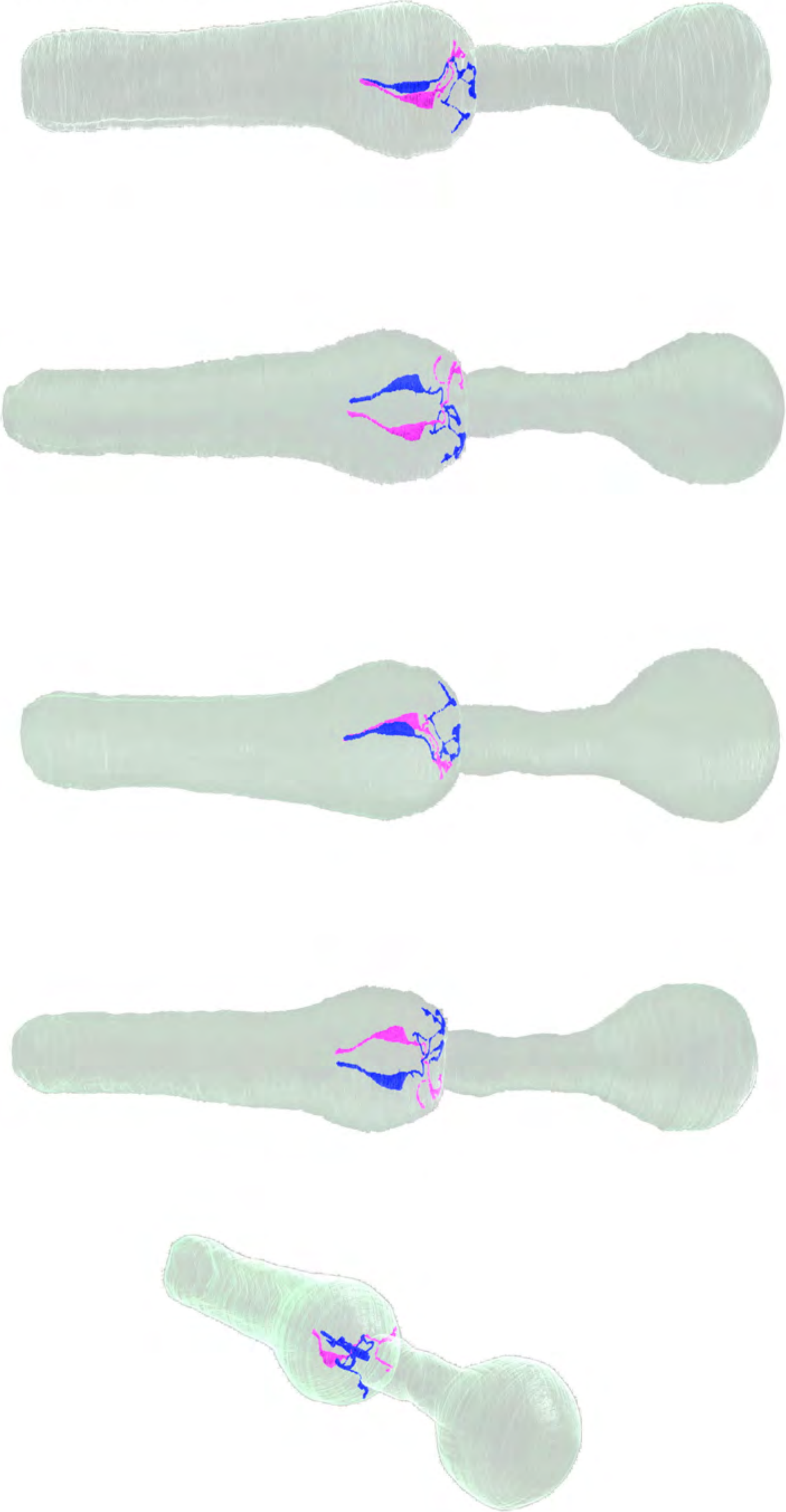

MI

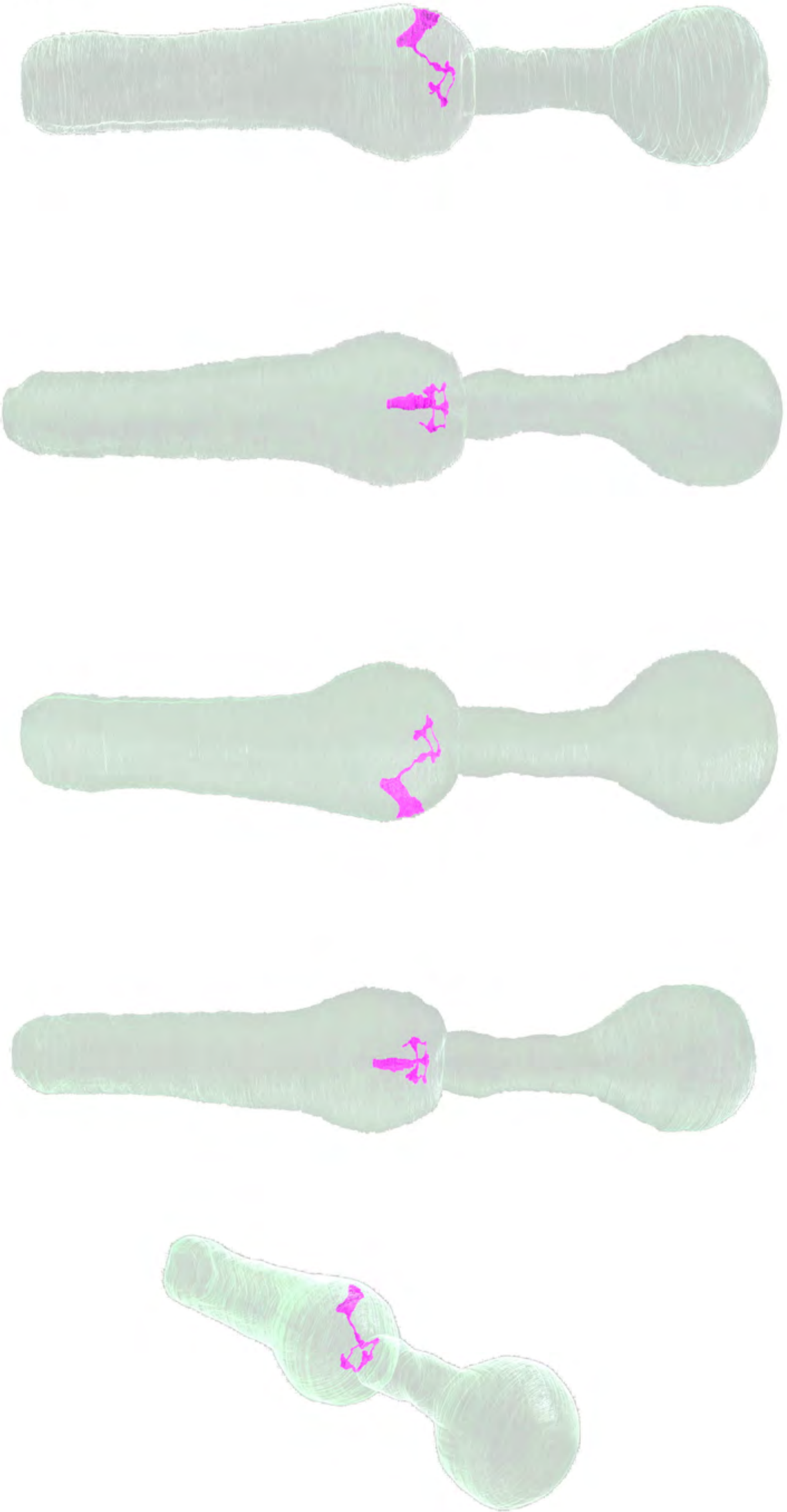

NSML, NSMR

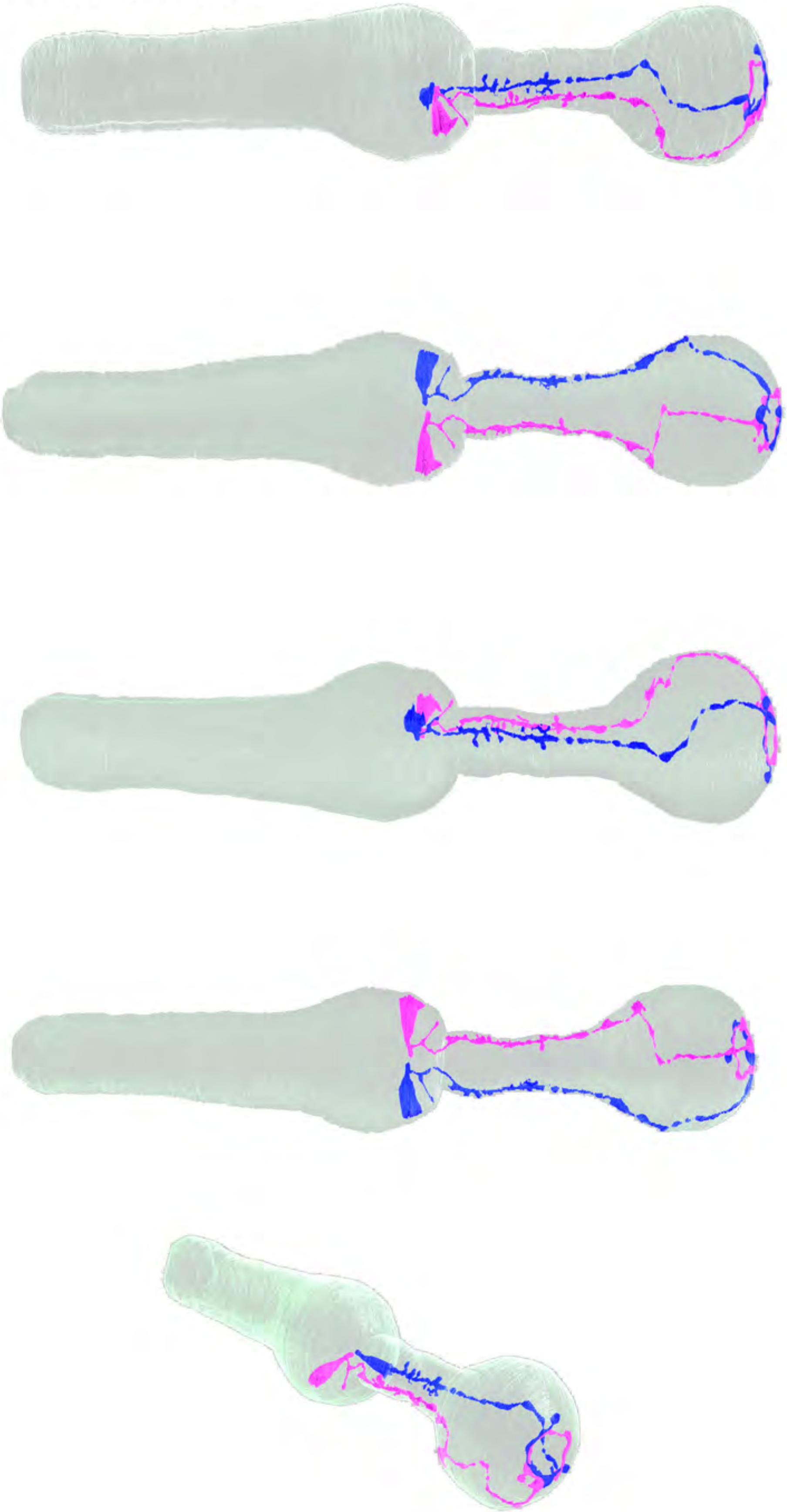

Supplement: Data S5 [file NIHMS2101571-supplement-Data_S5.pdf]
