# Supplementary figures and images for: Comparative connectomics of two distantly related nematode species reveals patterns of nervous system evolution
Source: Science. Author manuscript; Available in PMC 2025 Aug 7. (PMC12330220; doi:10.1126/science.adx2143)

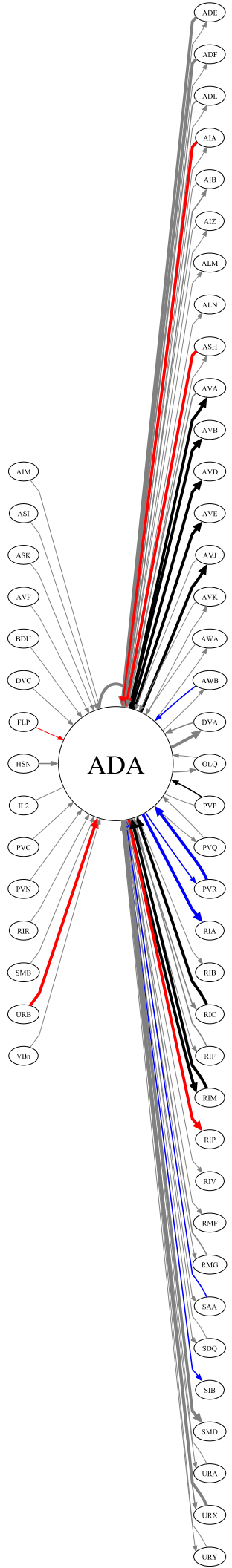

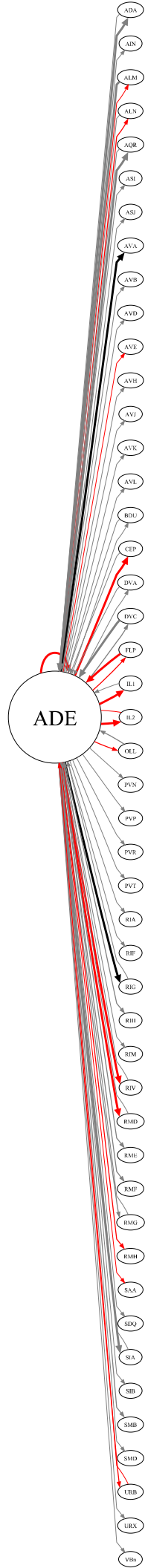

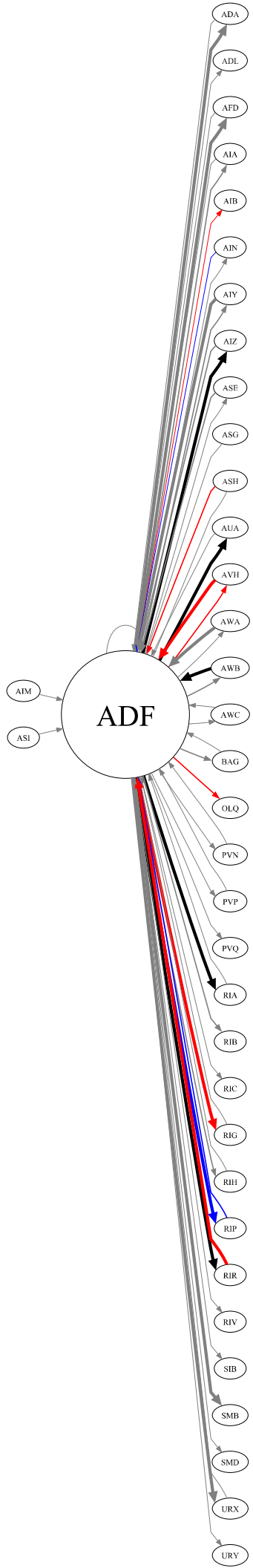

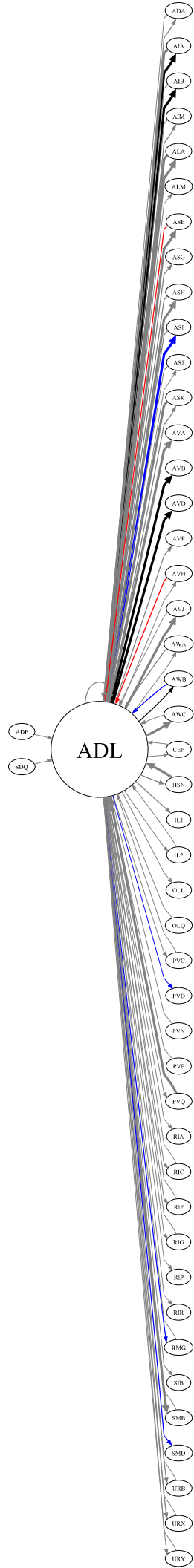

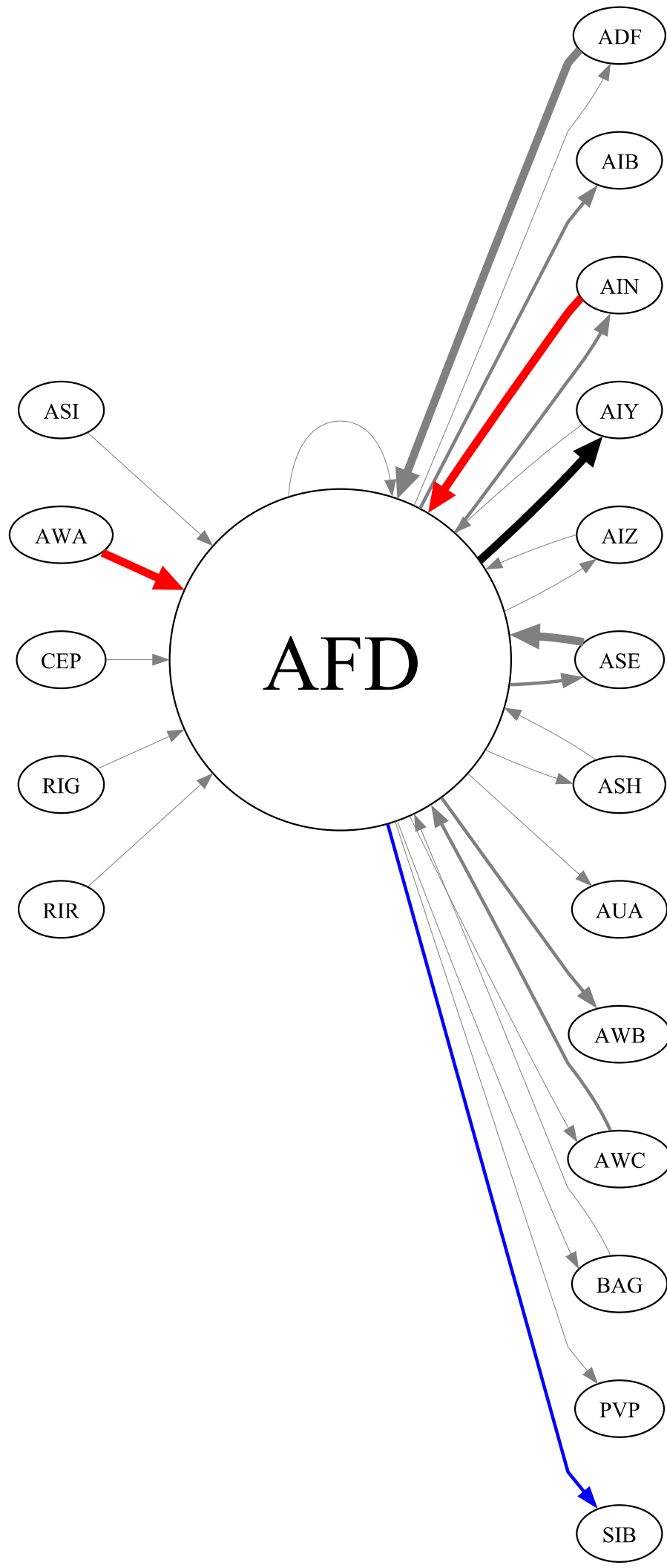

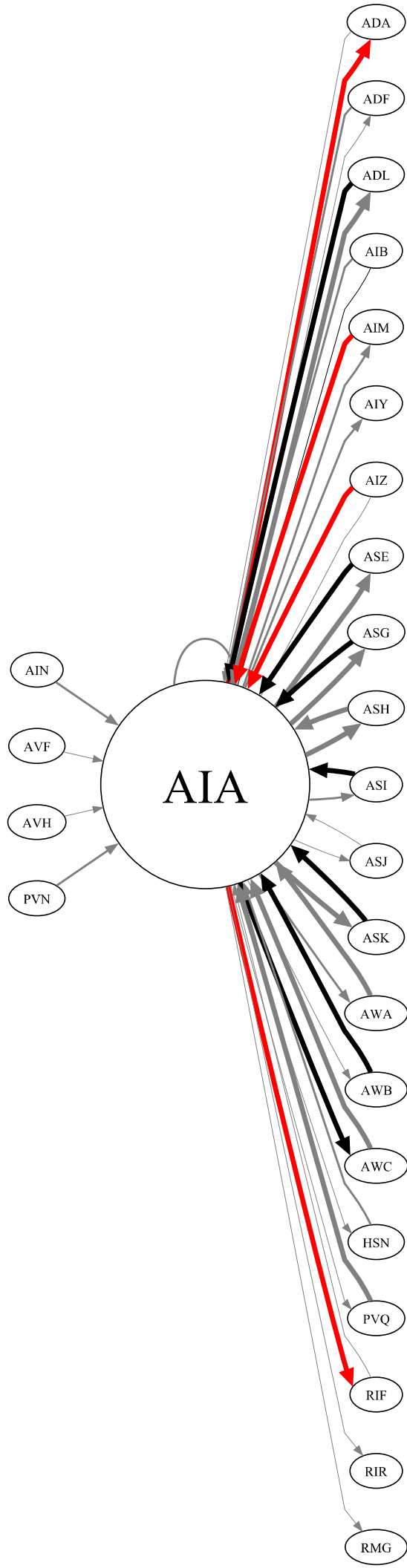

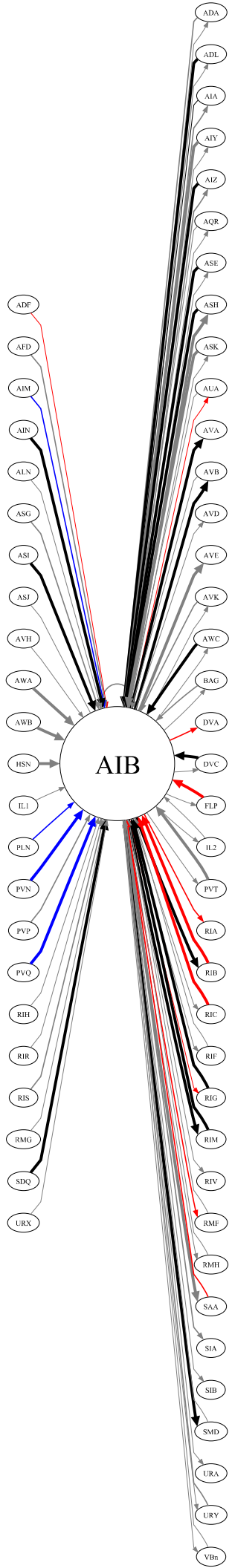

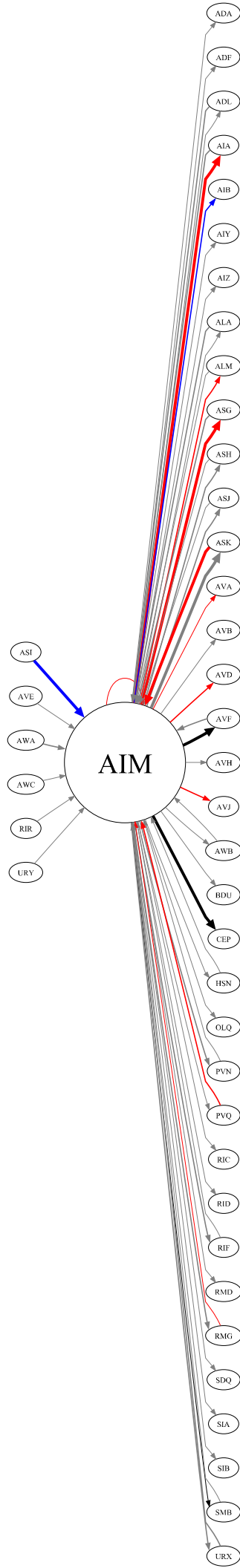

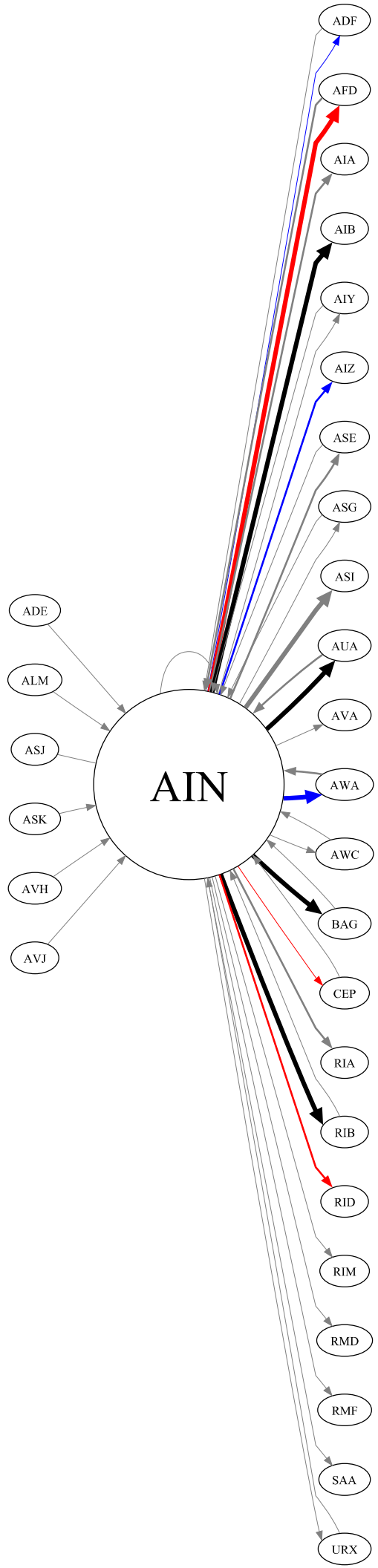

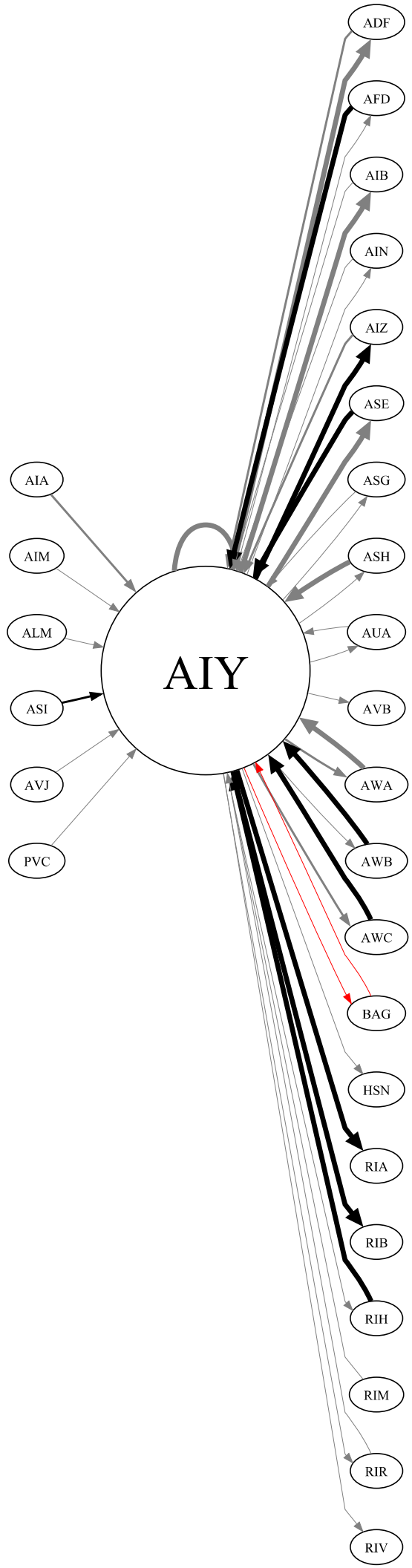

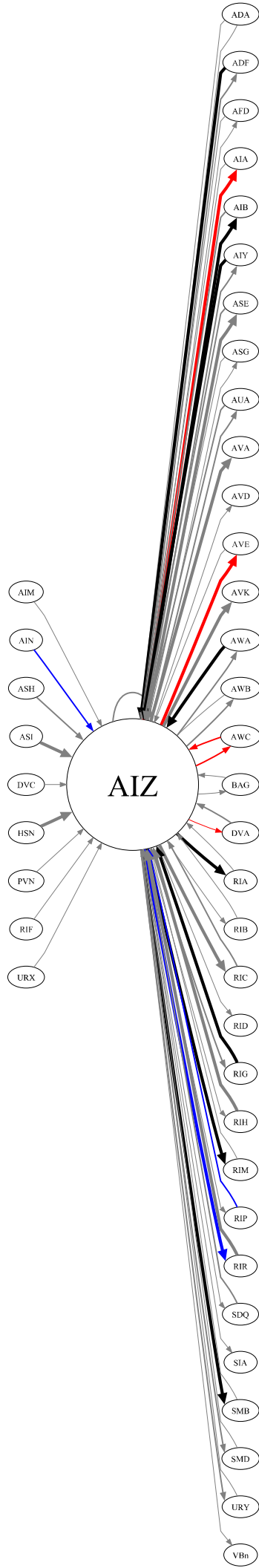

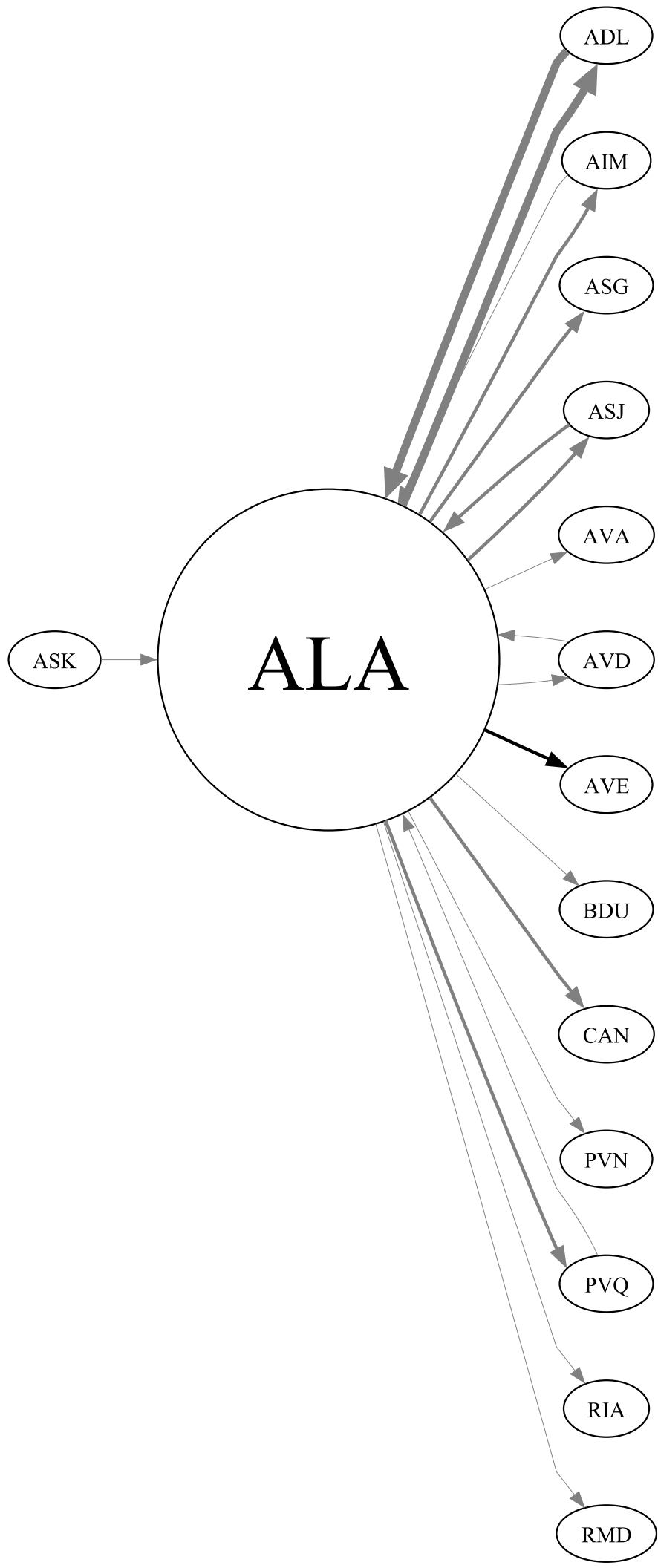

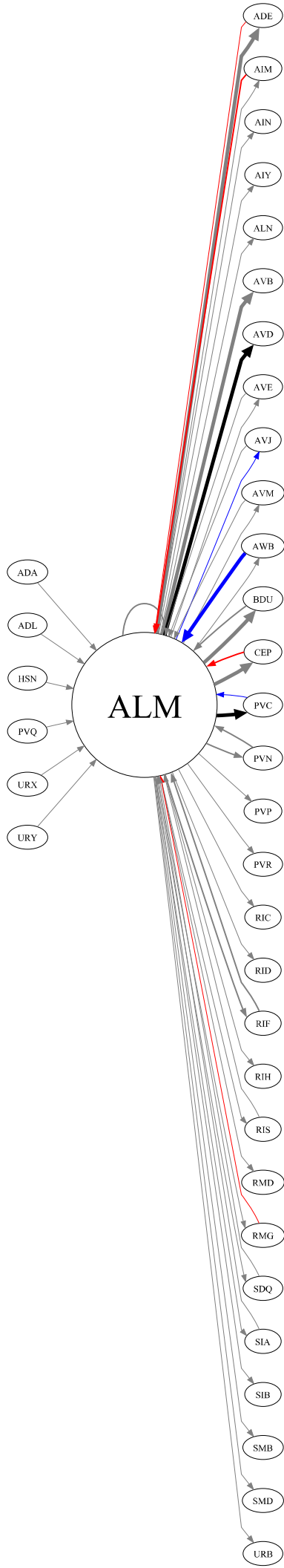

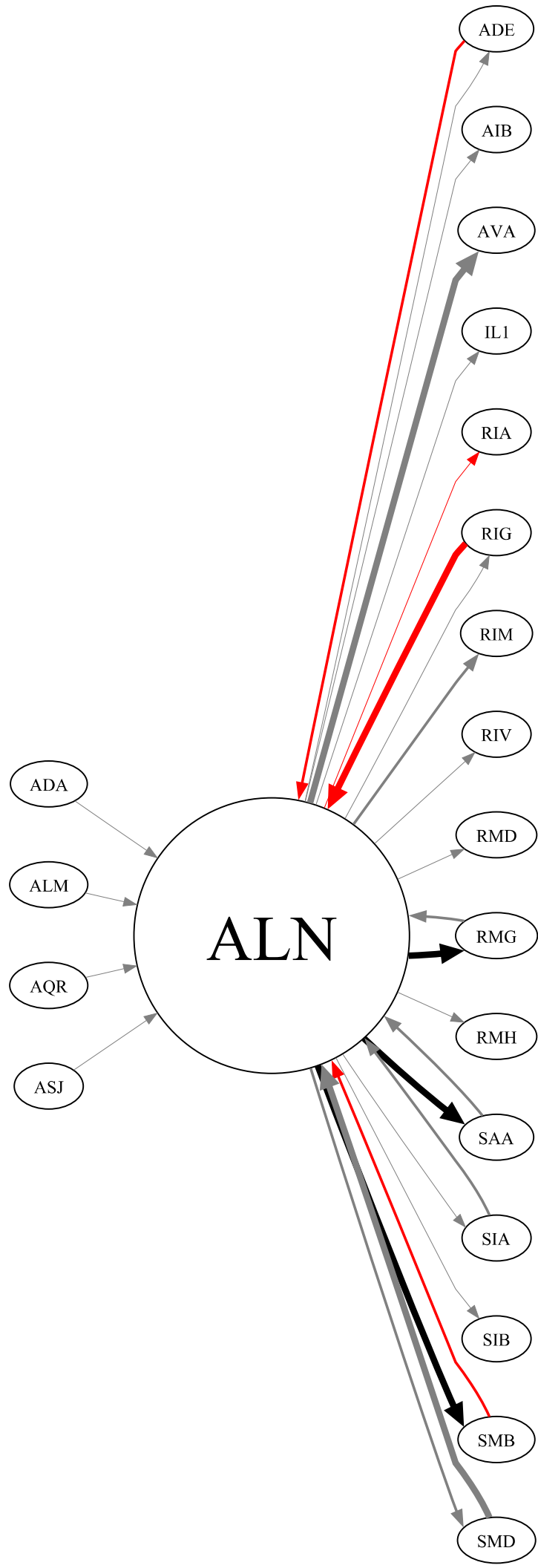

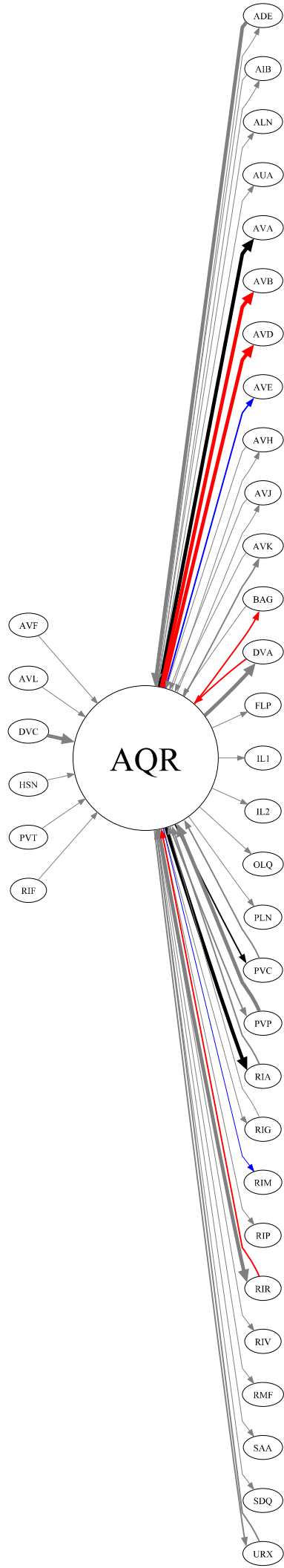

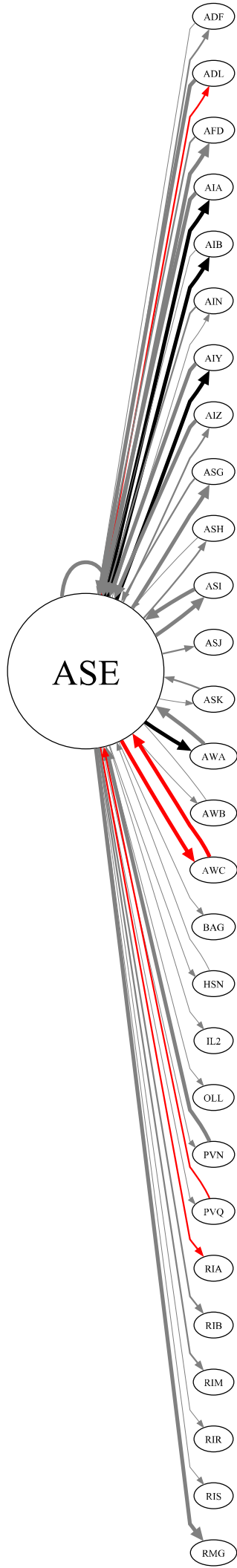

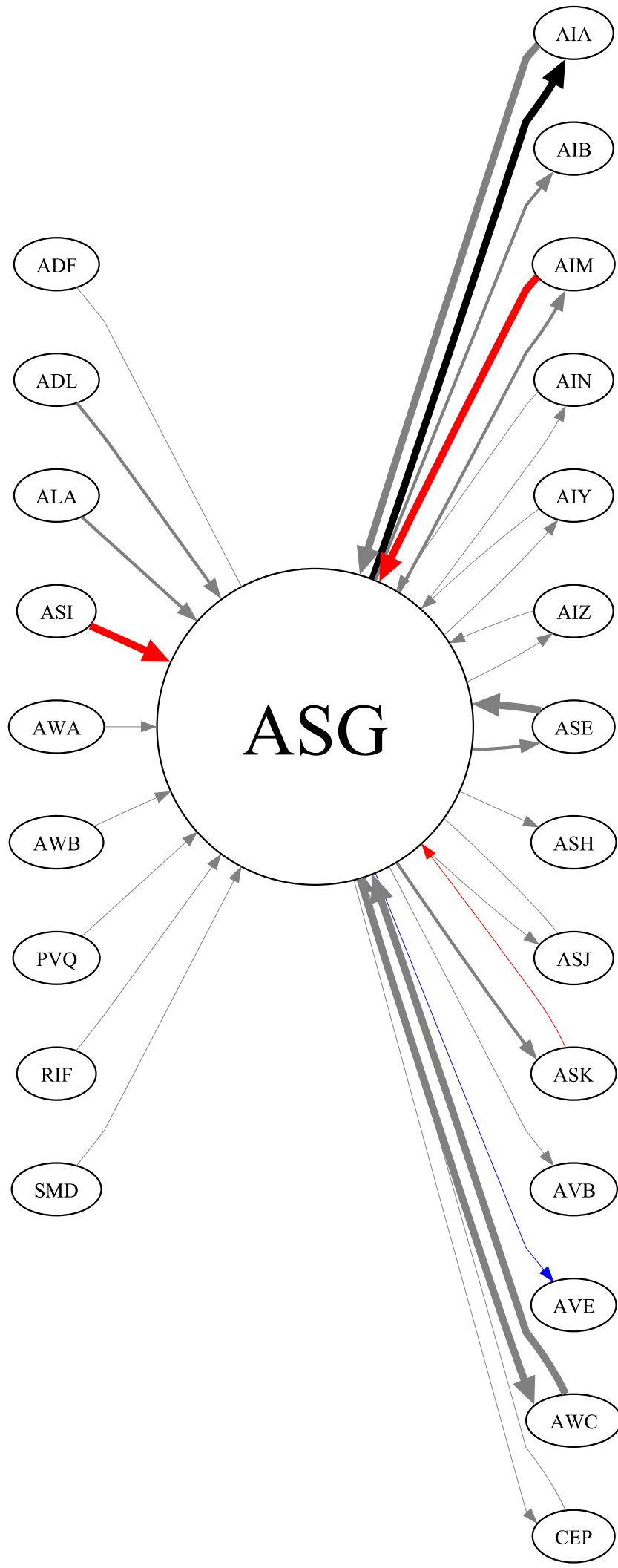

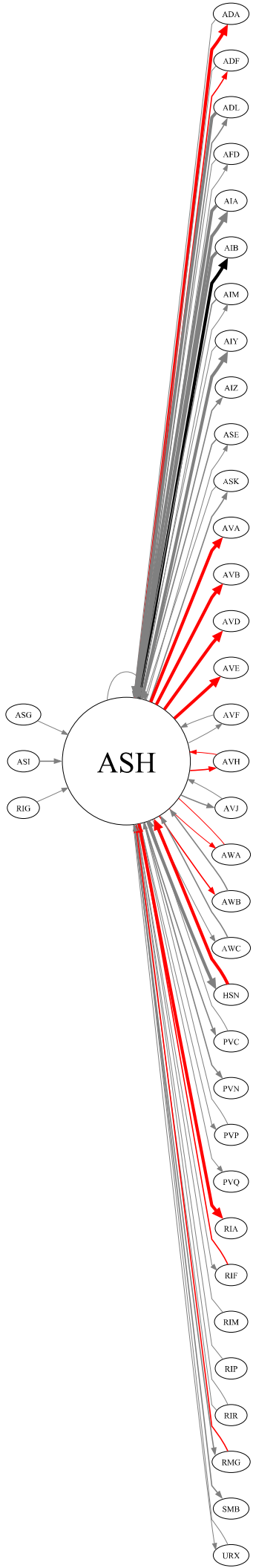

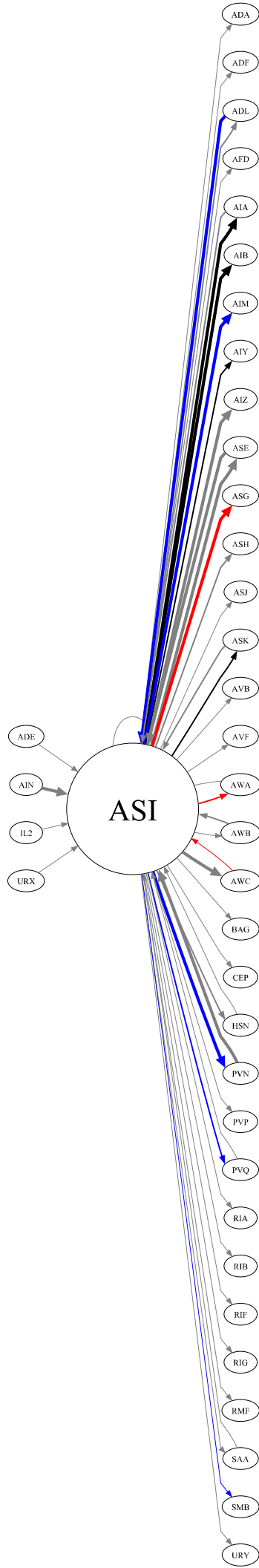

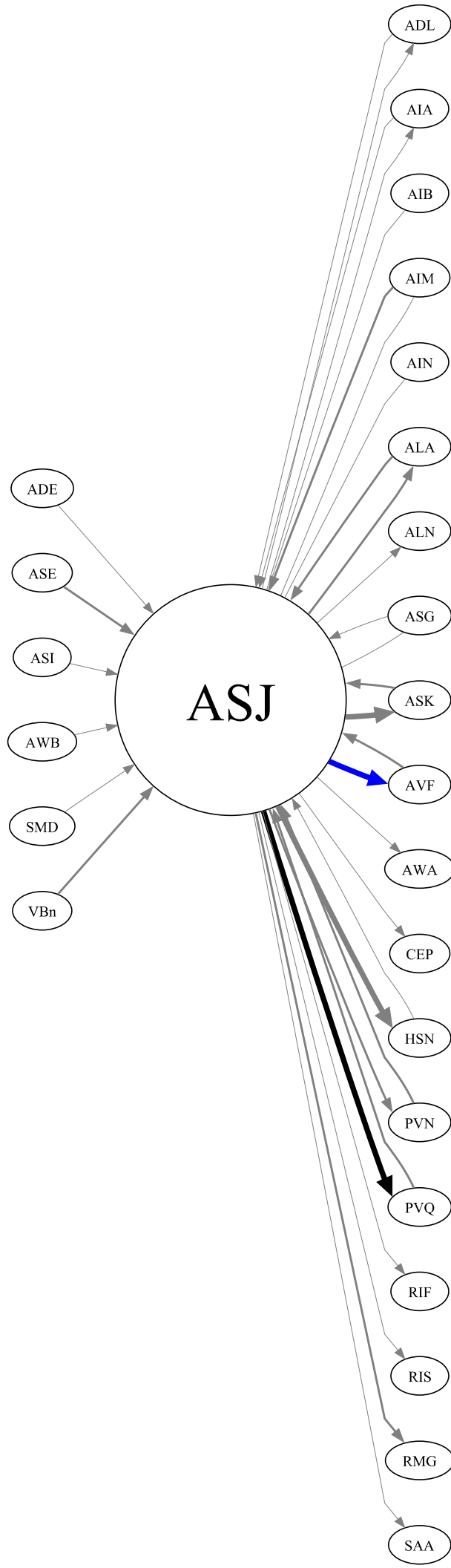

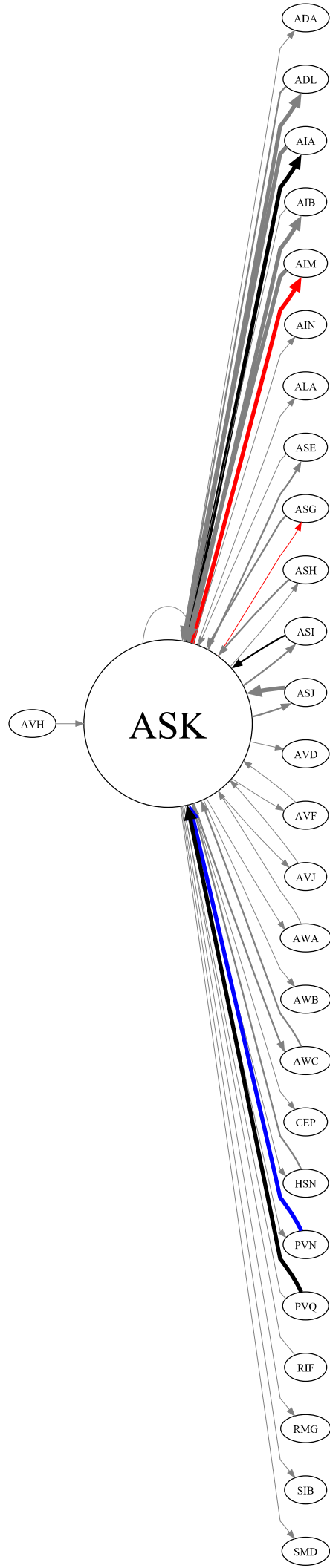

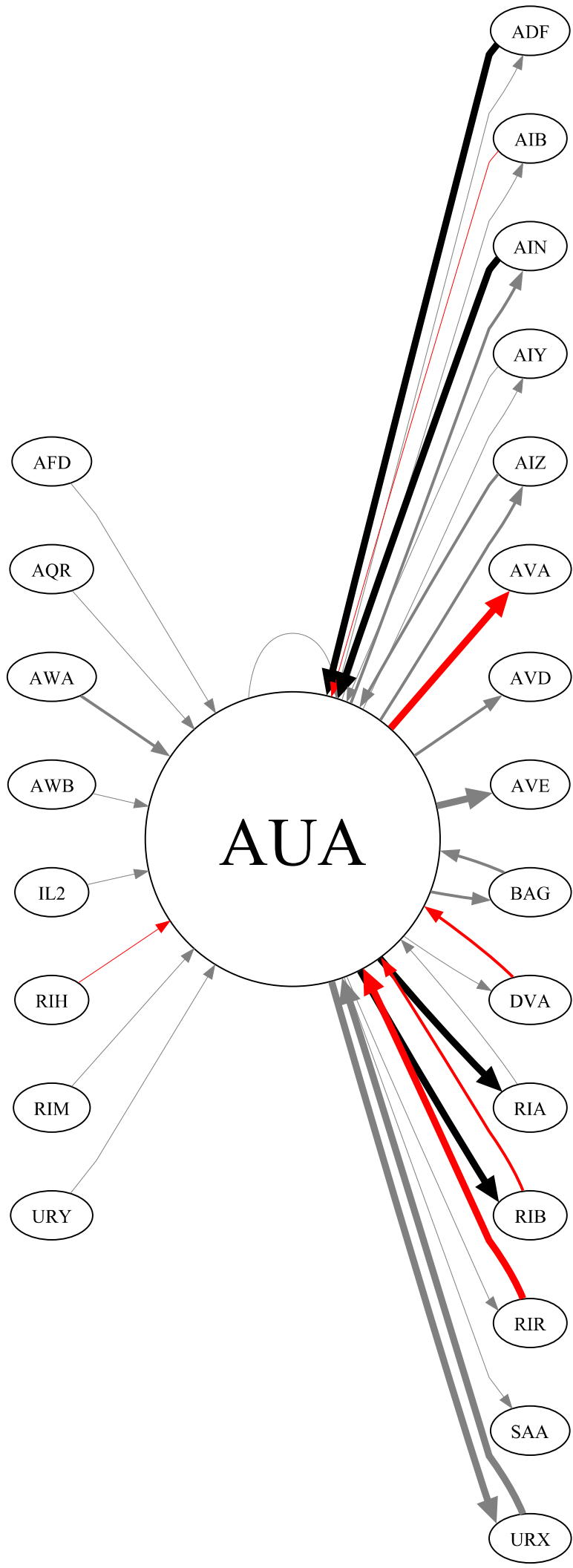

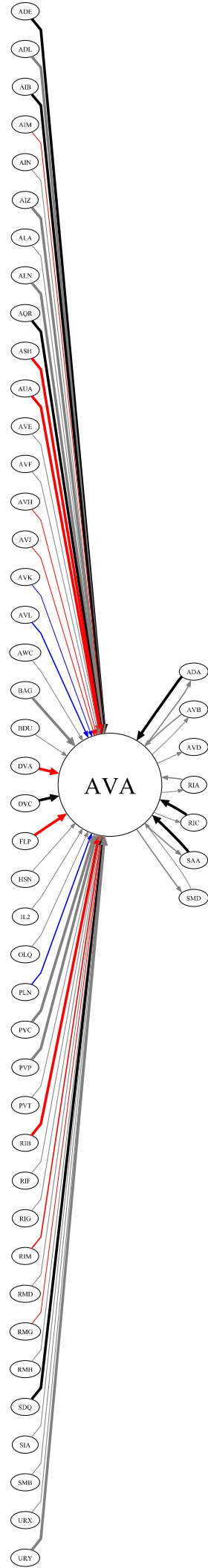

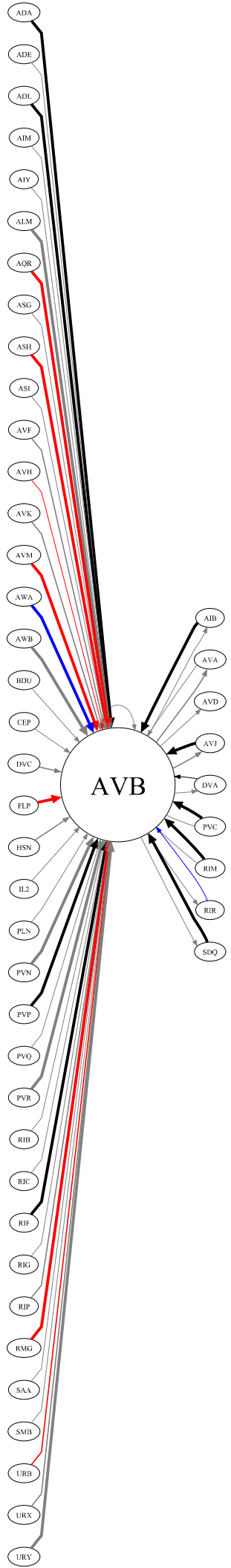

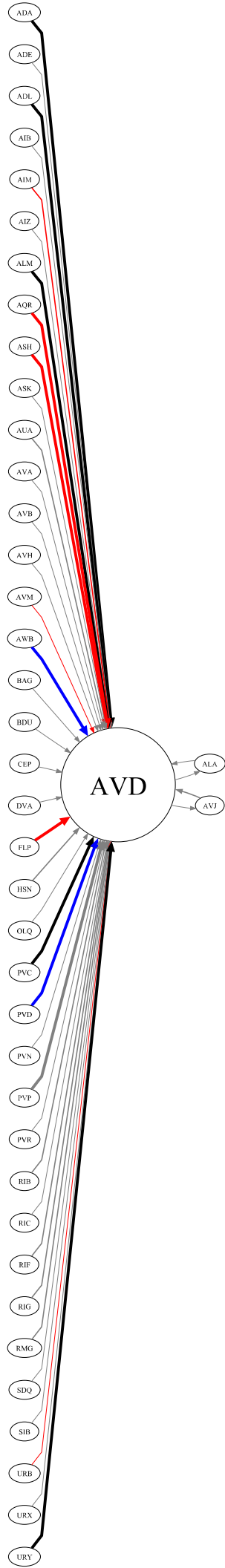

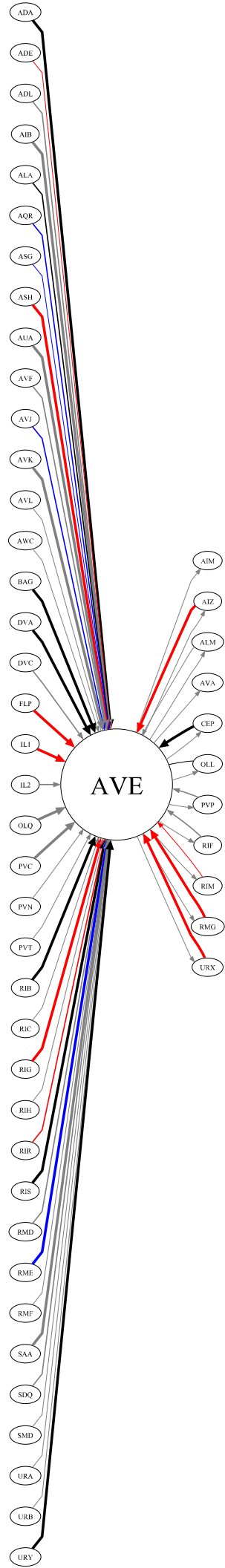

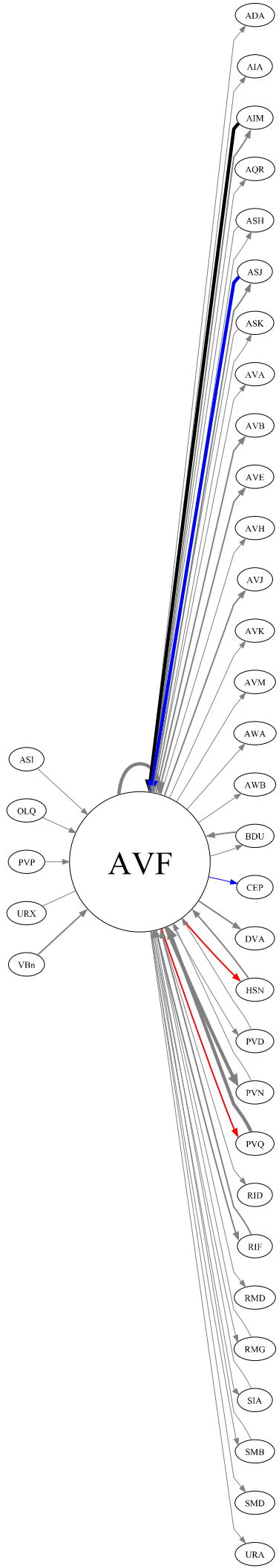

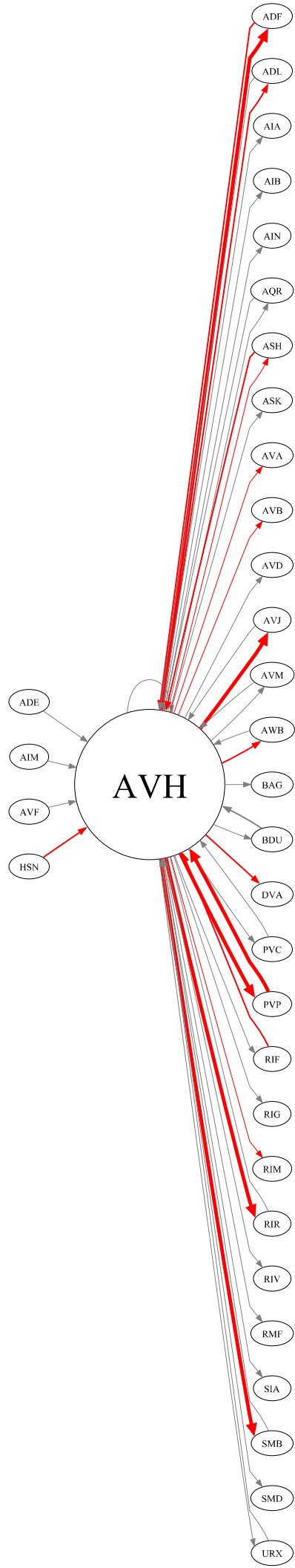

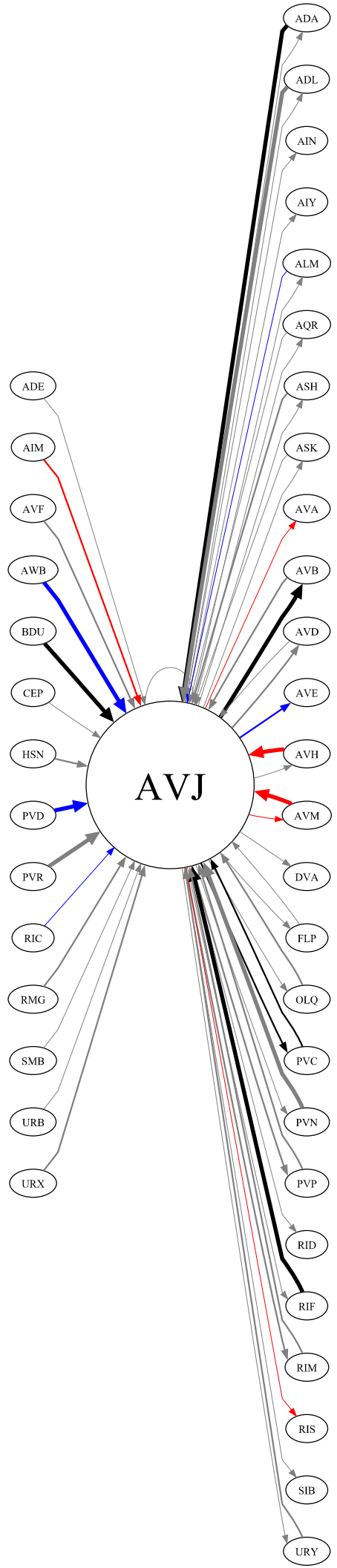

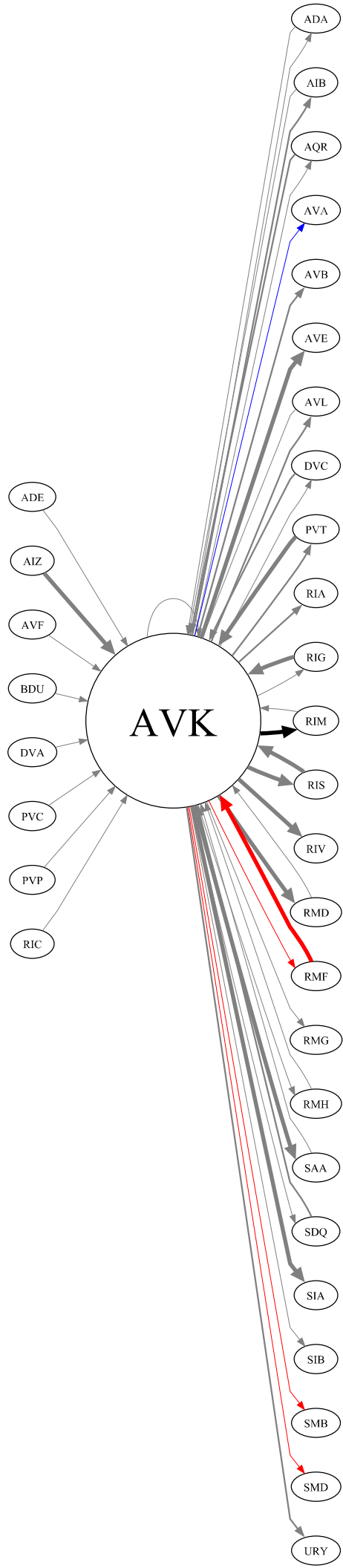

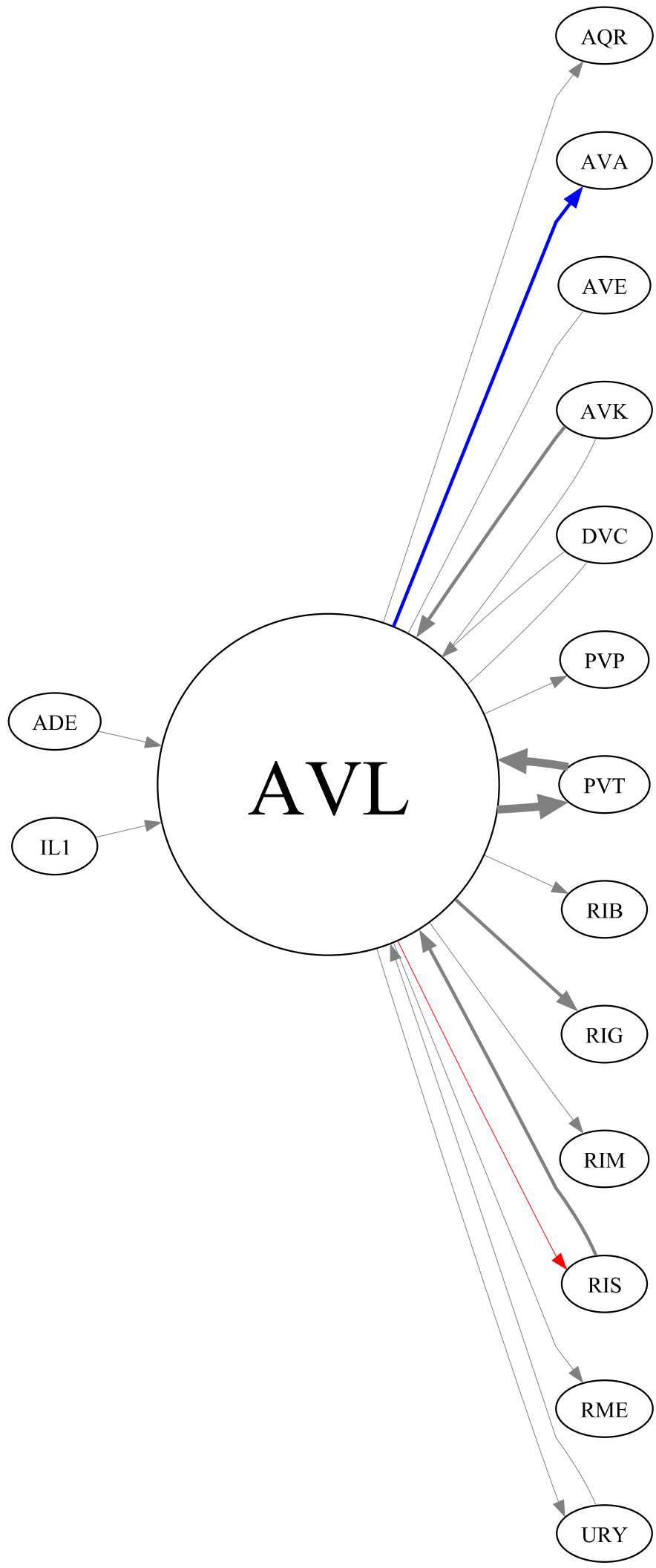

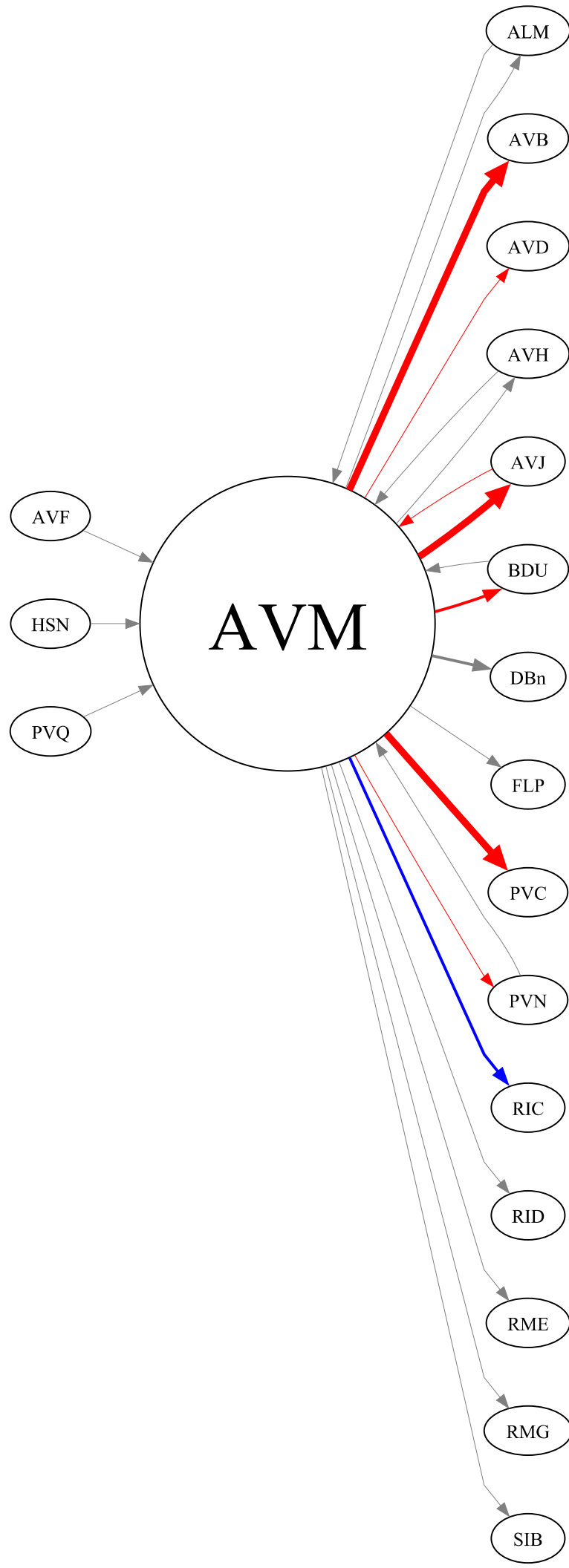

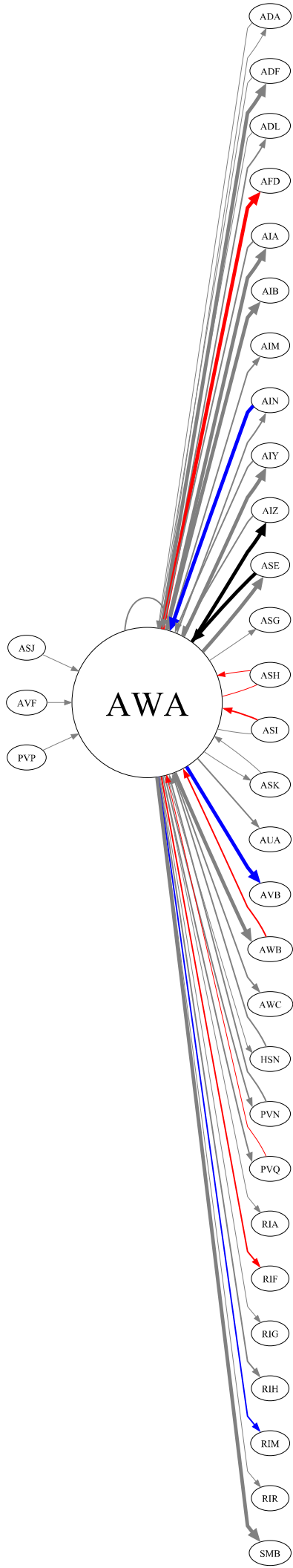

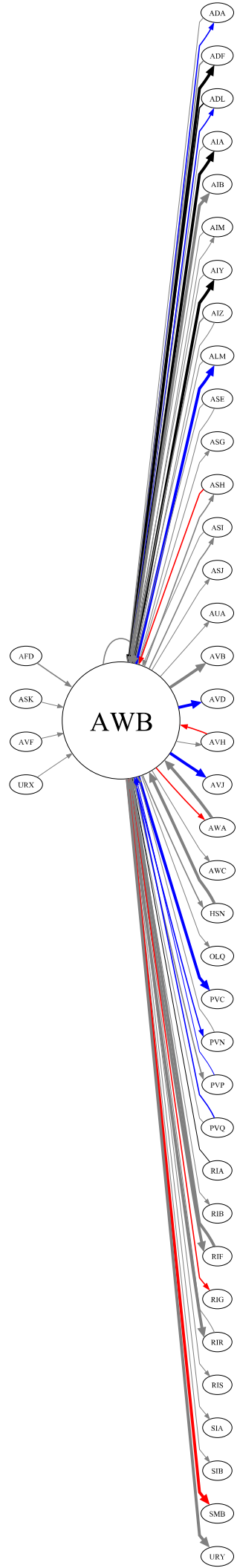

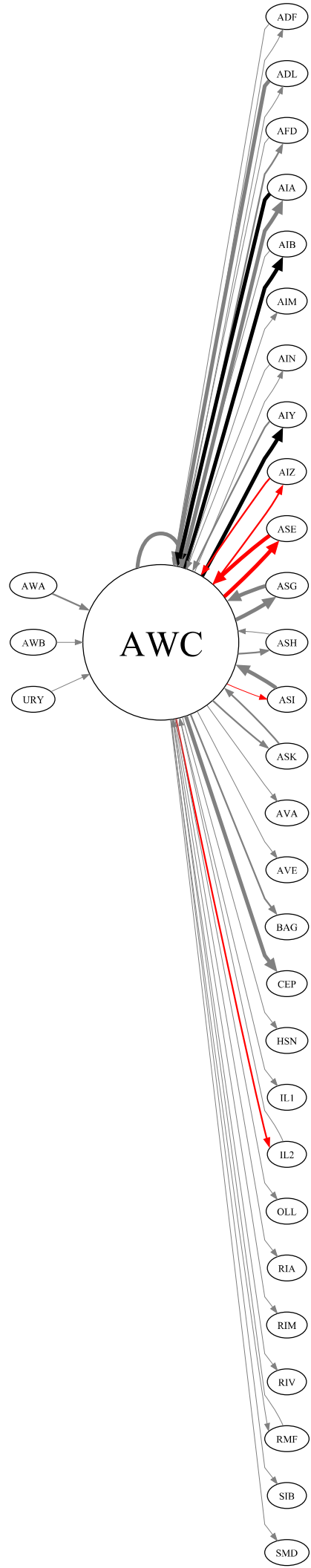

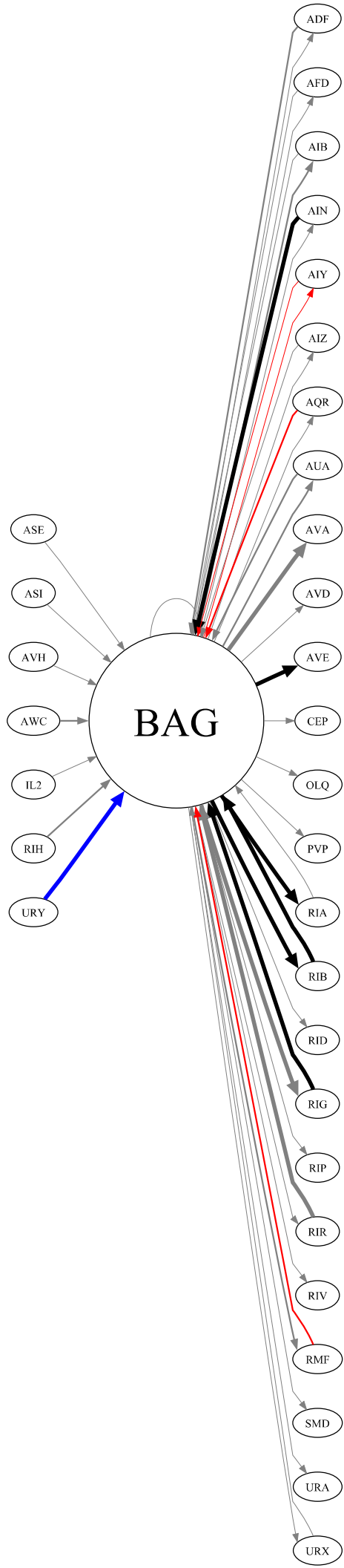

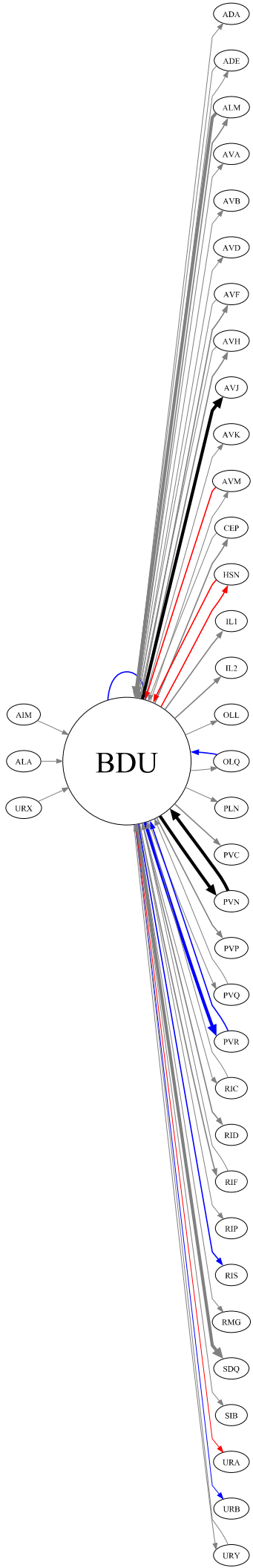

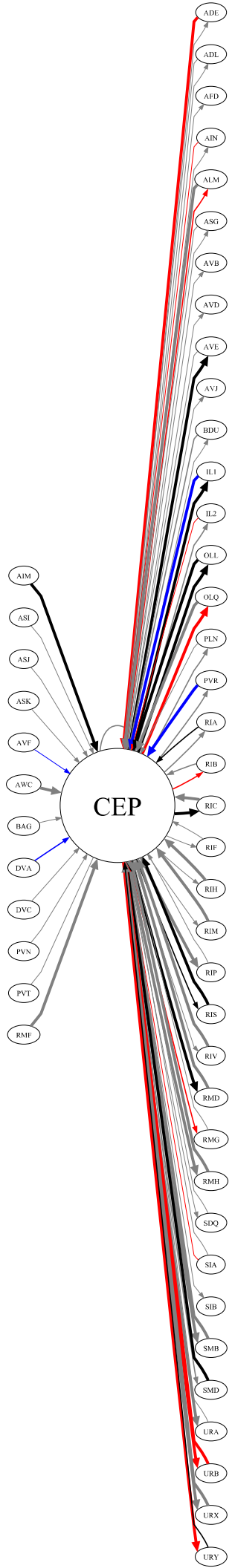

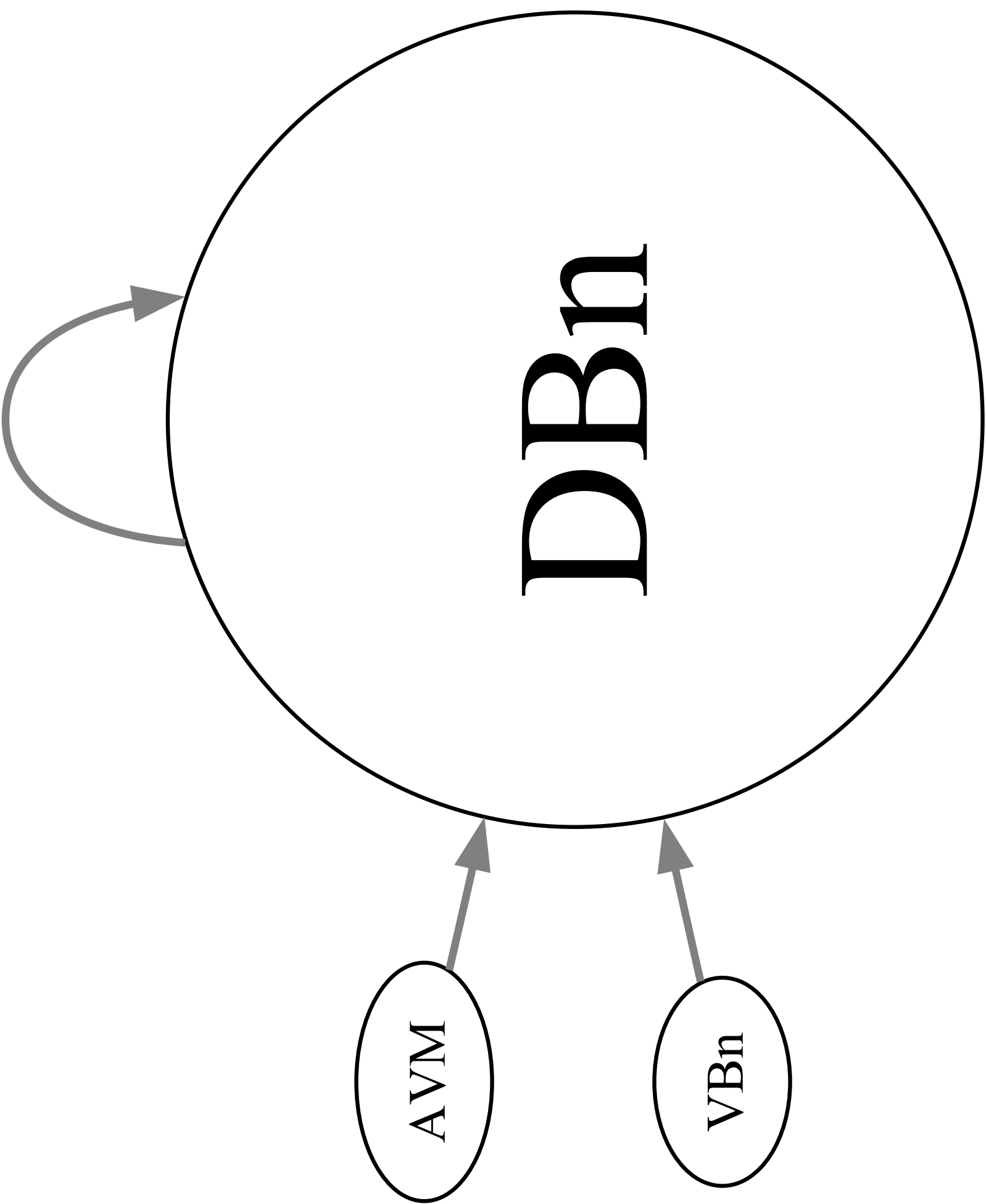

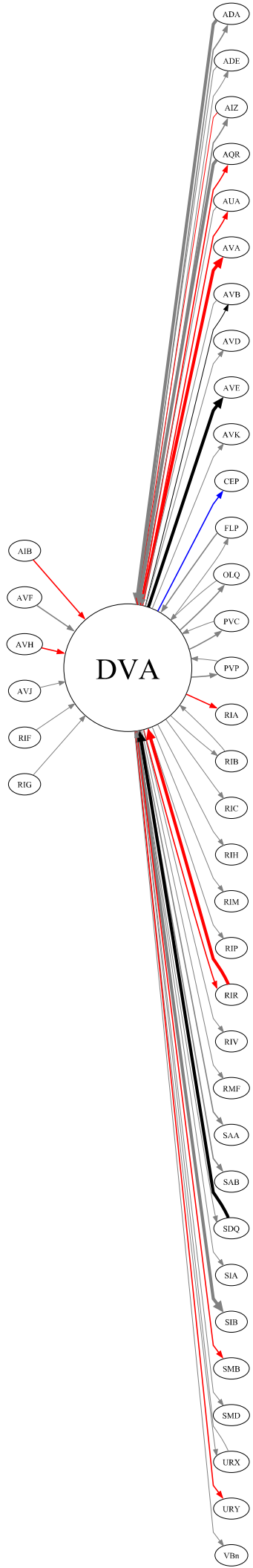

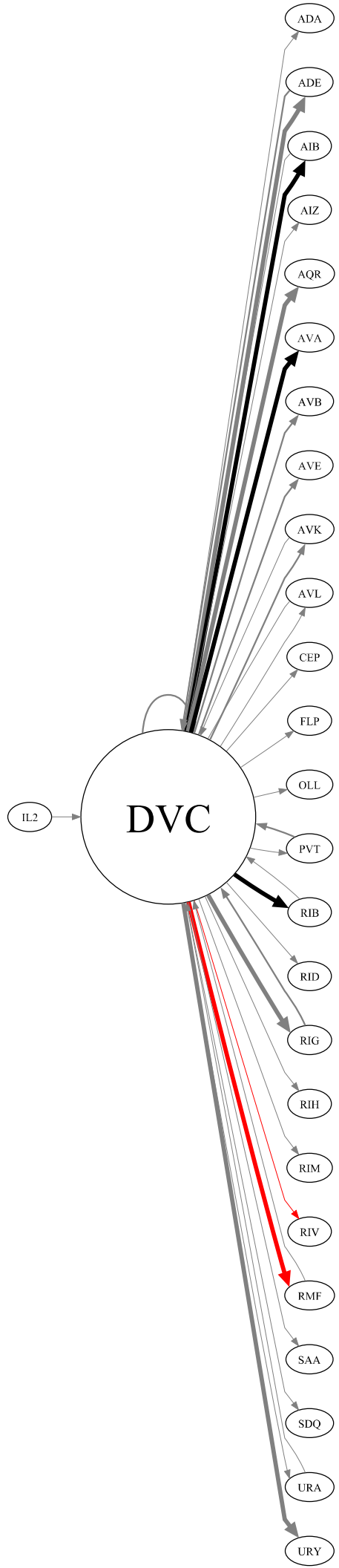

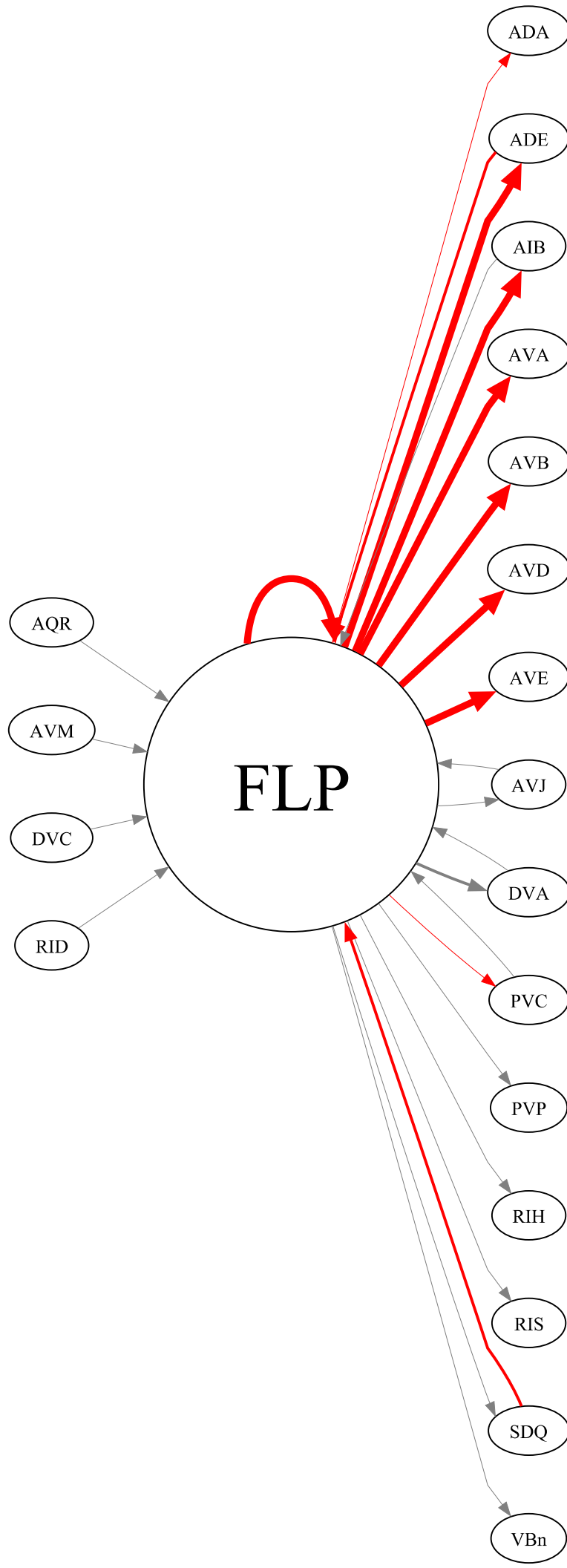

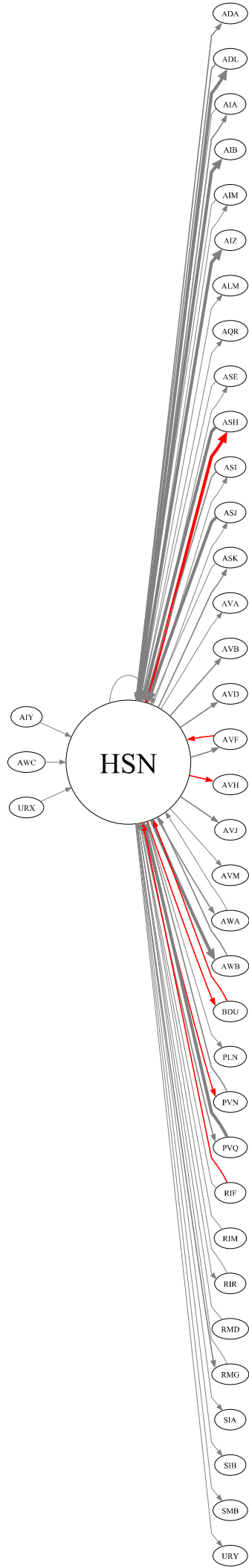

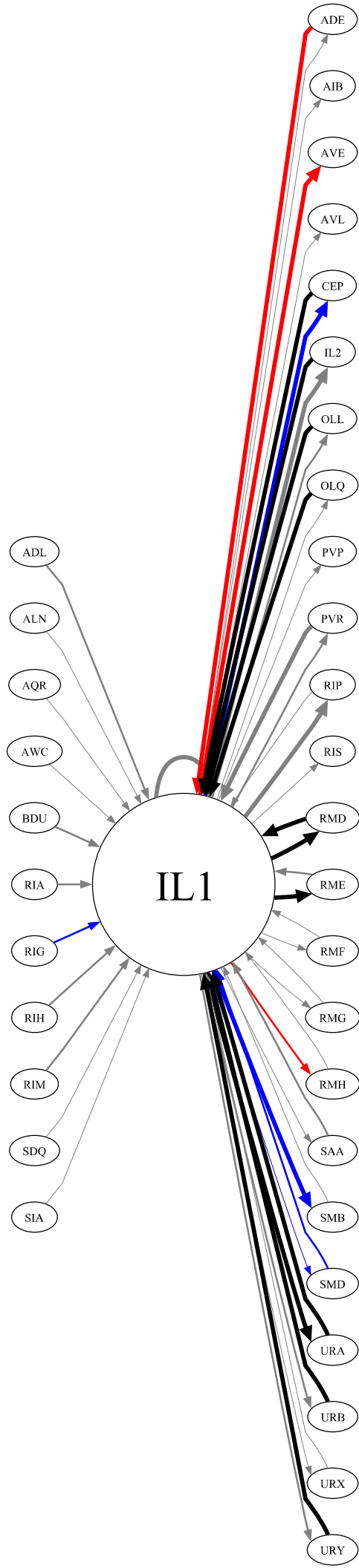

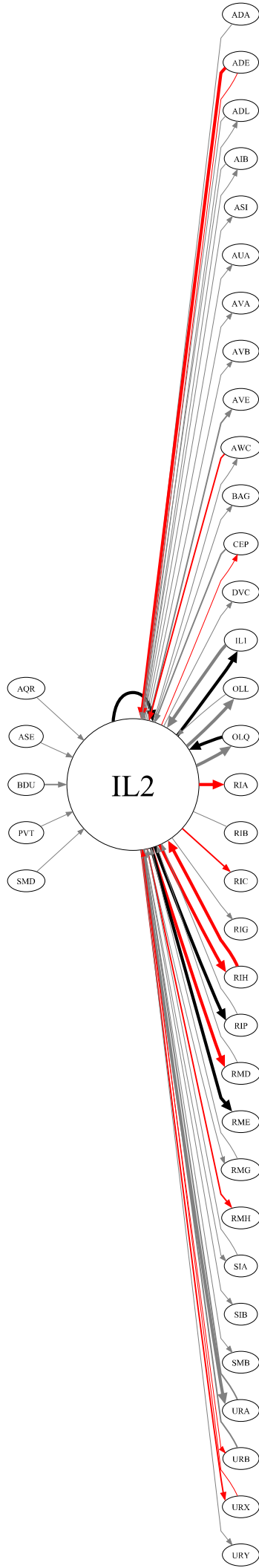

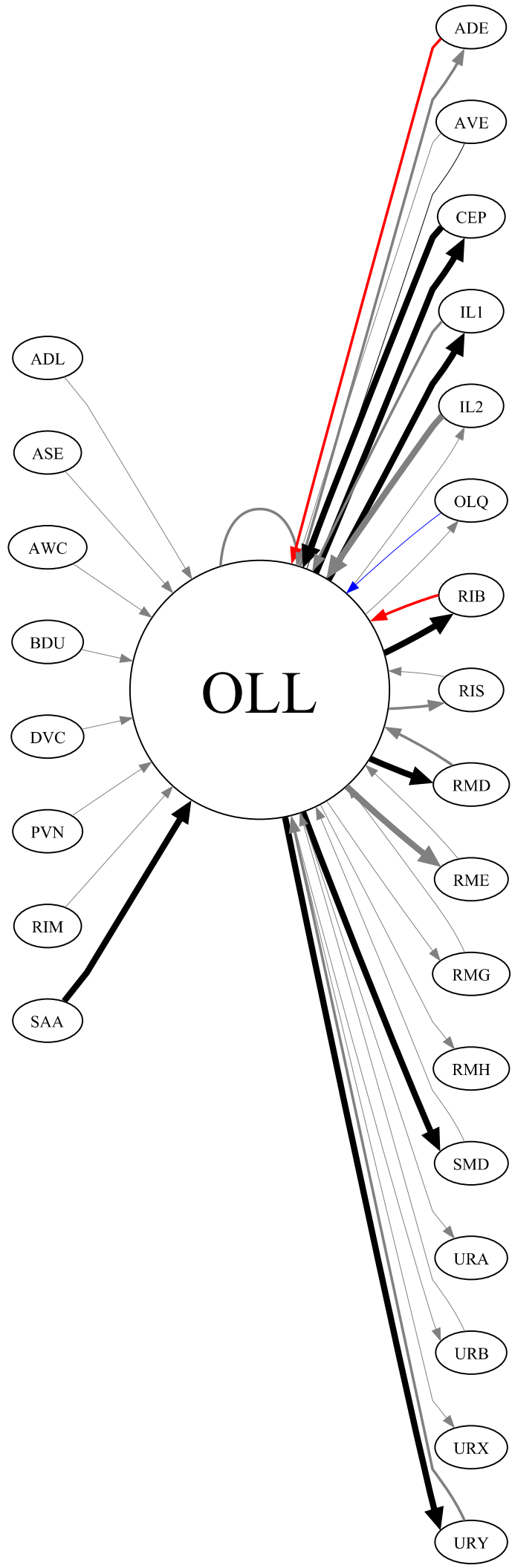

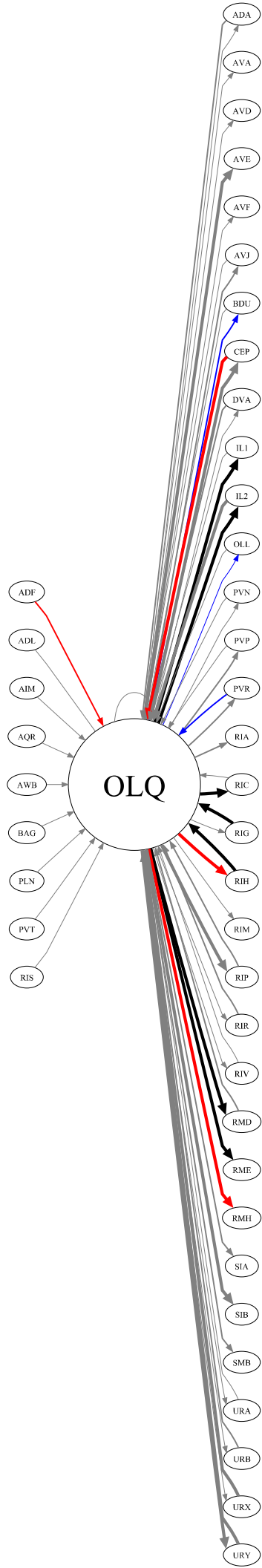

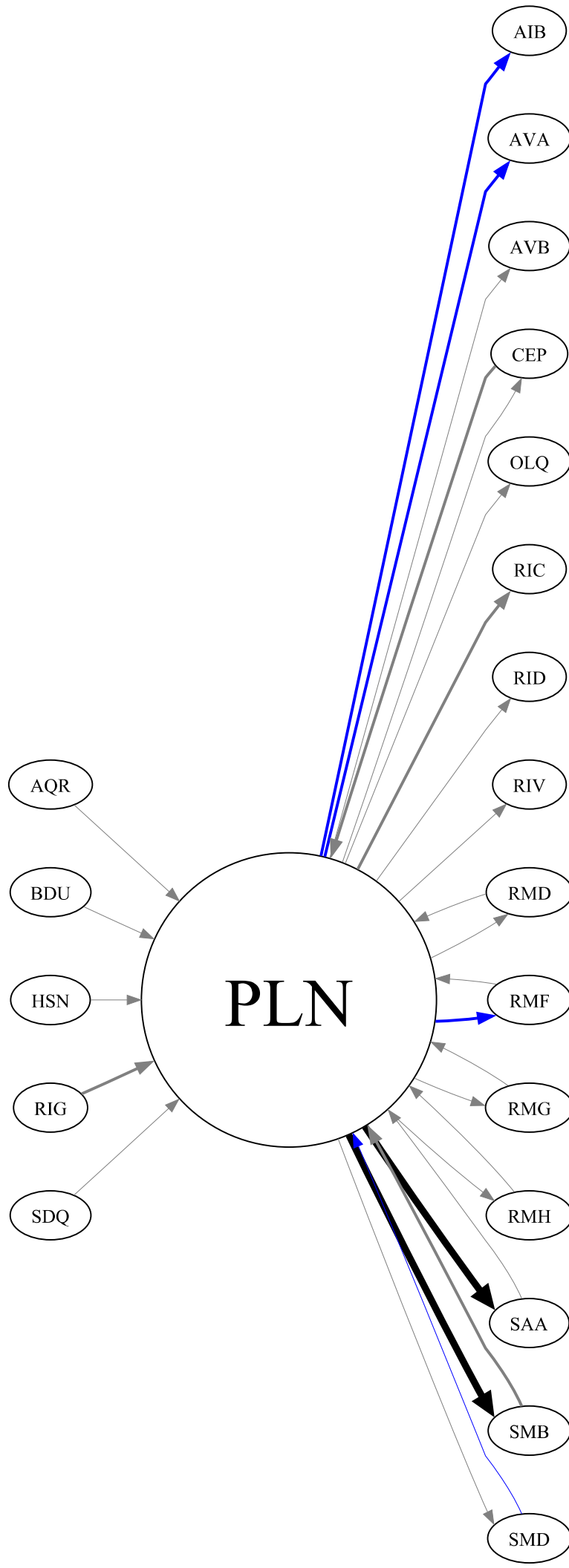

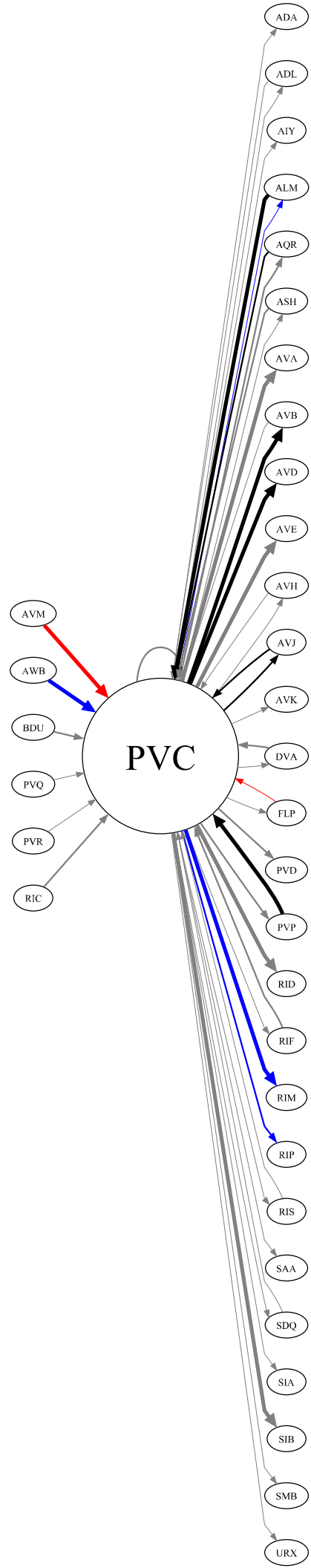

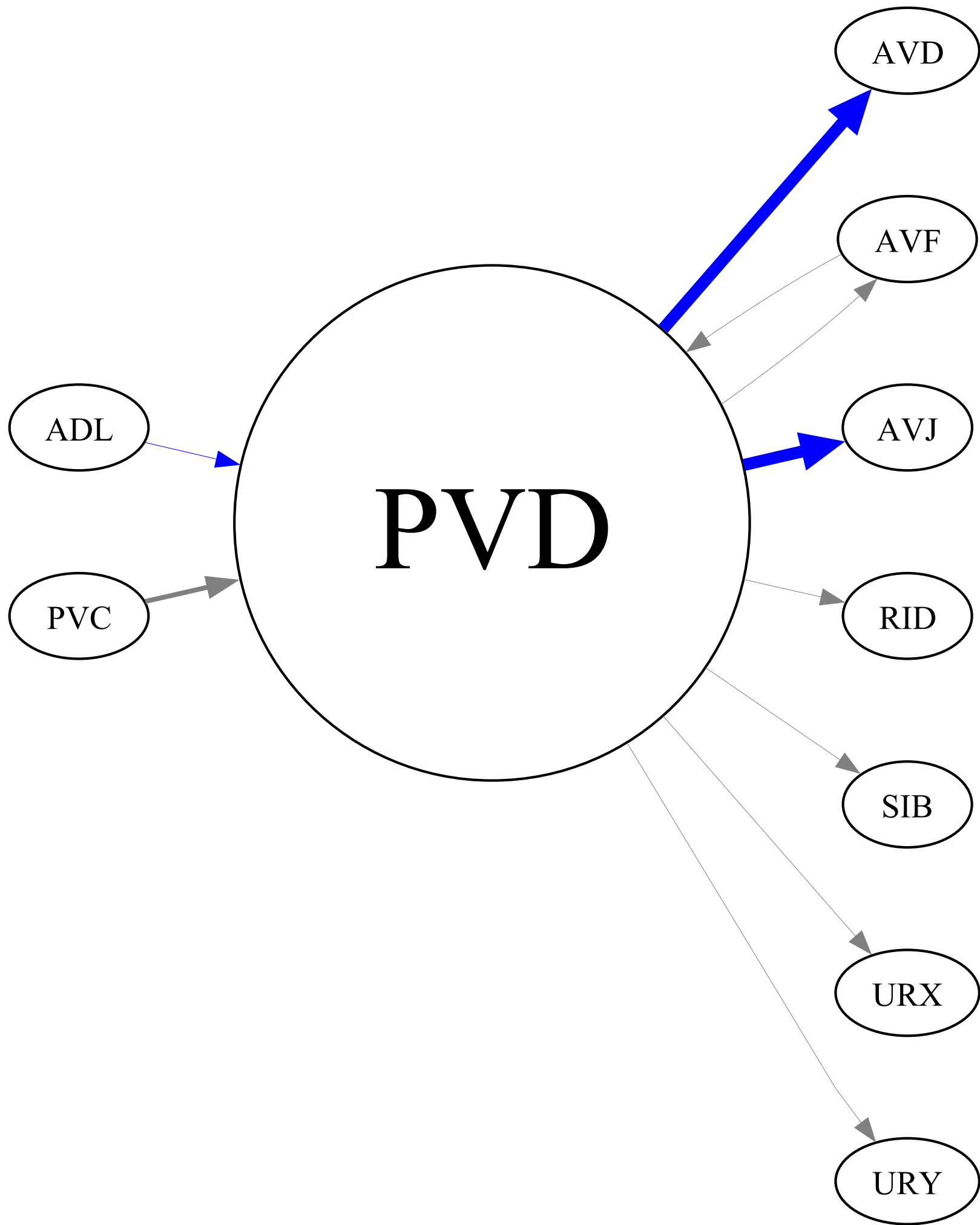

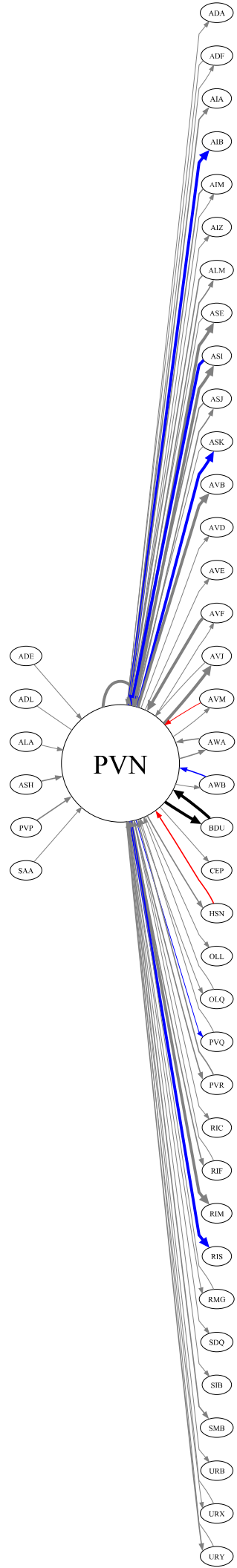

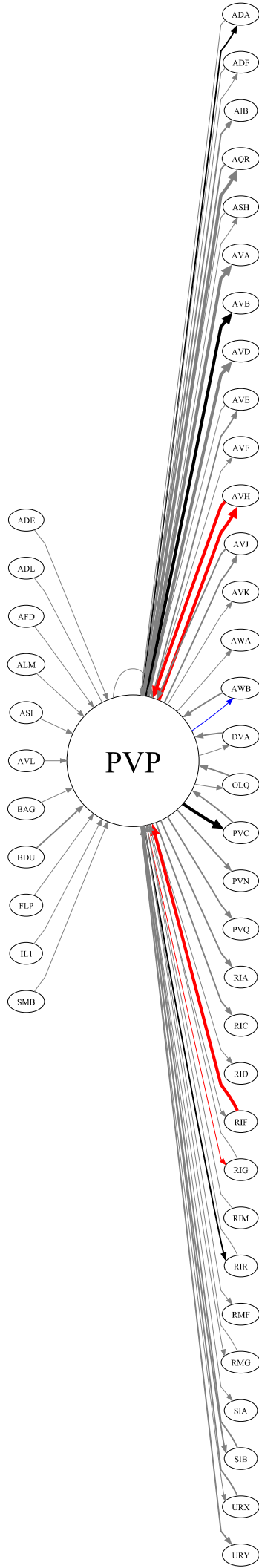

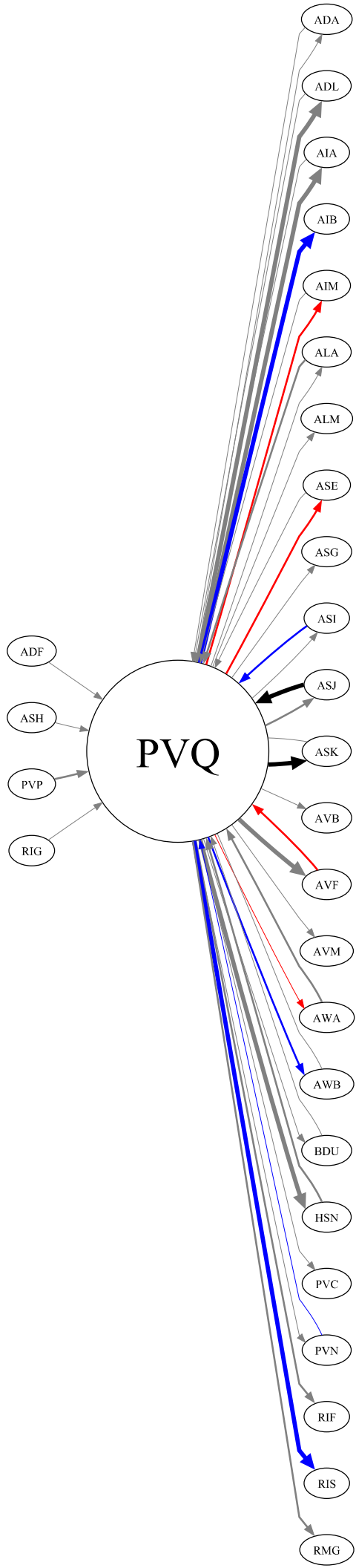

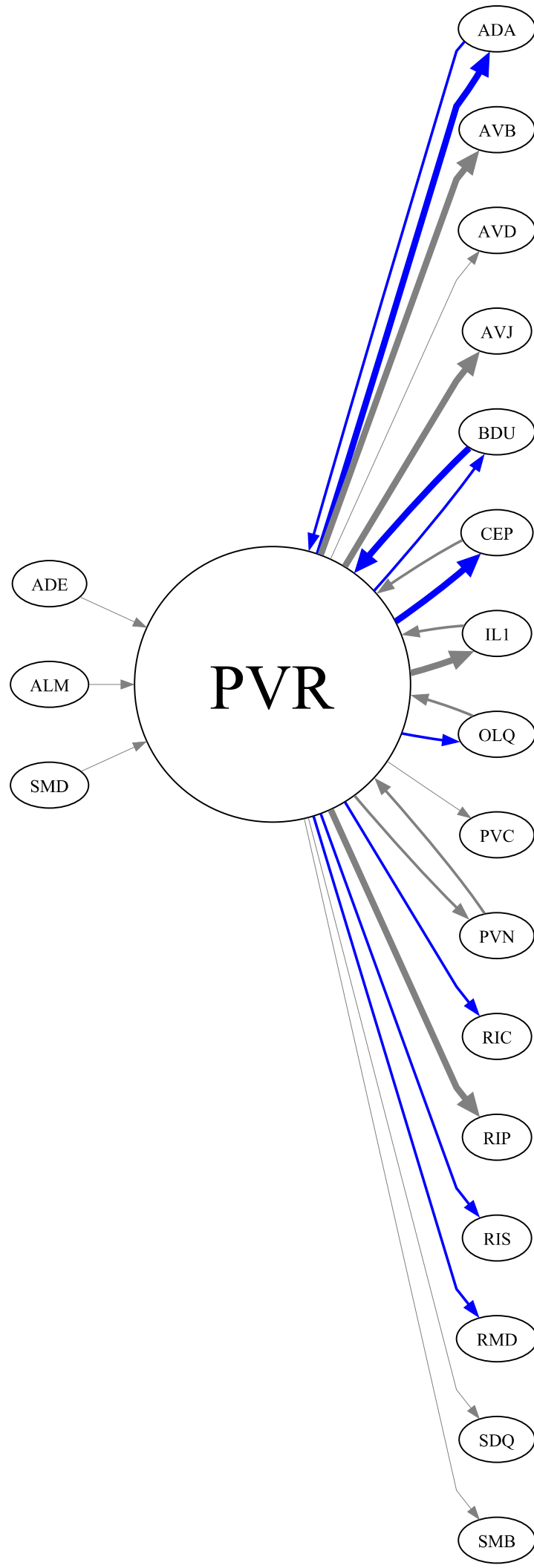

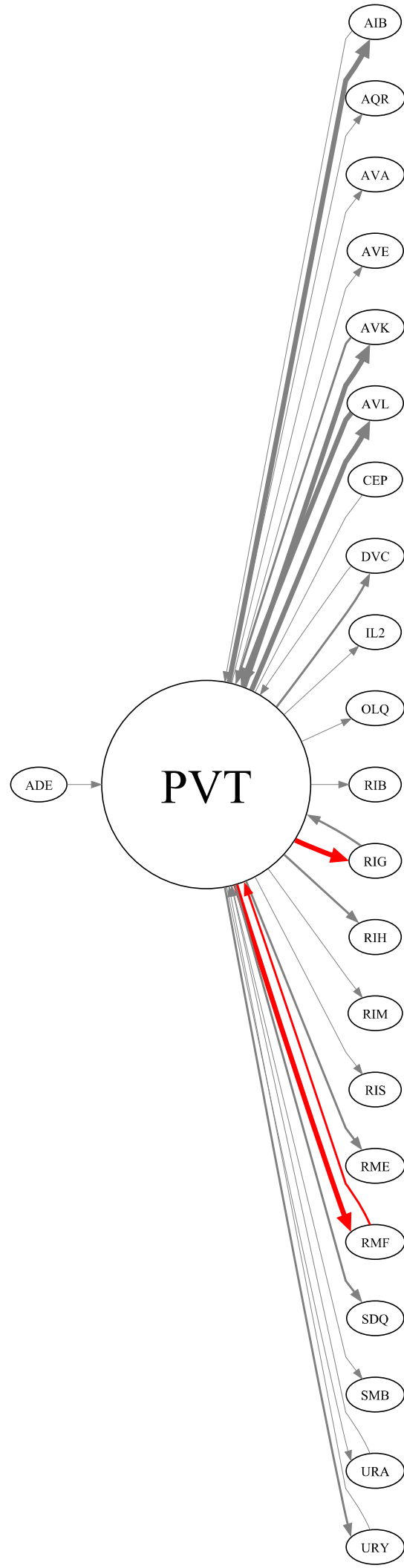

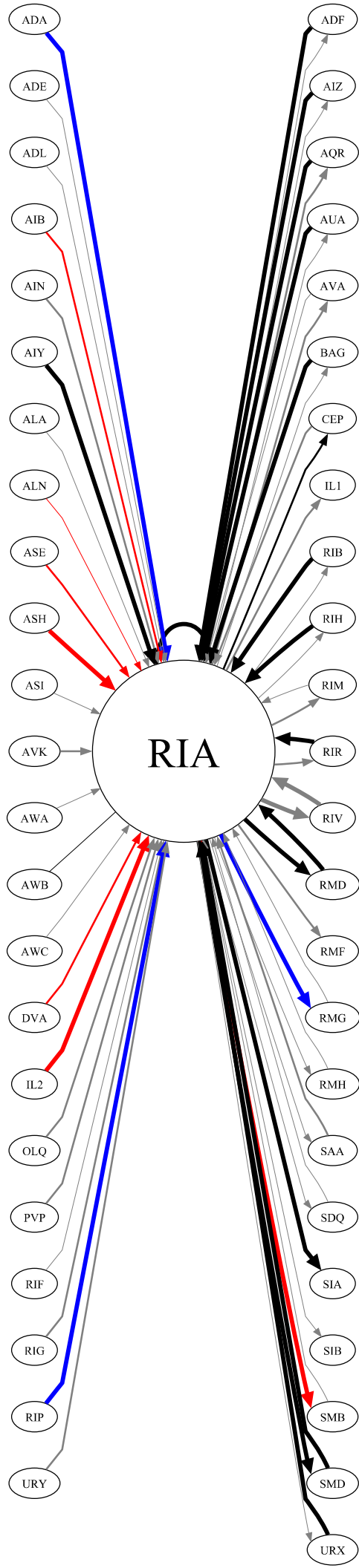

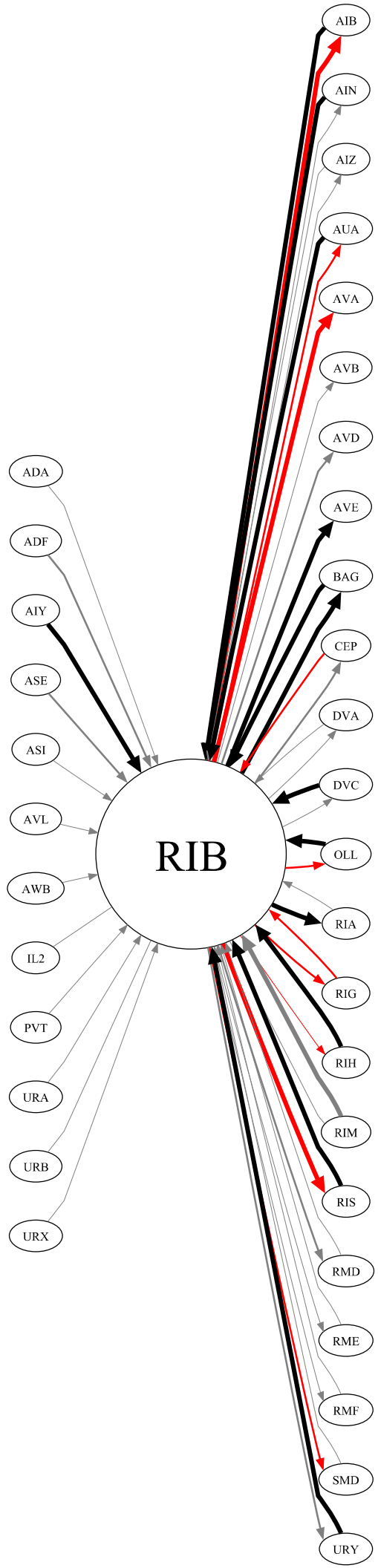

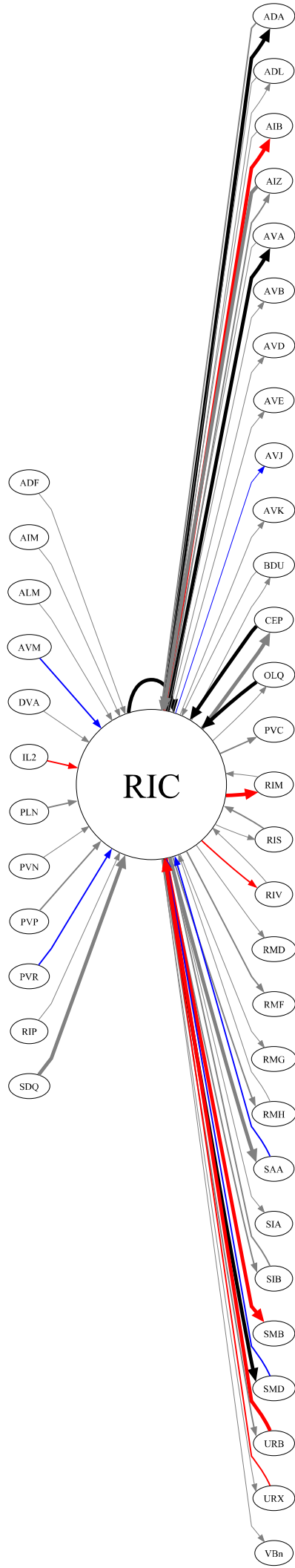

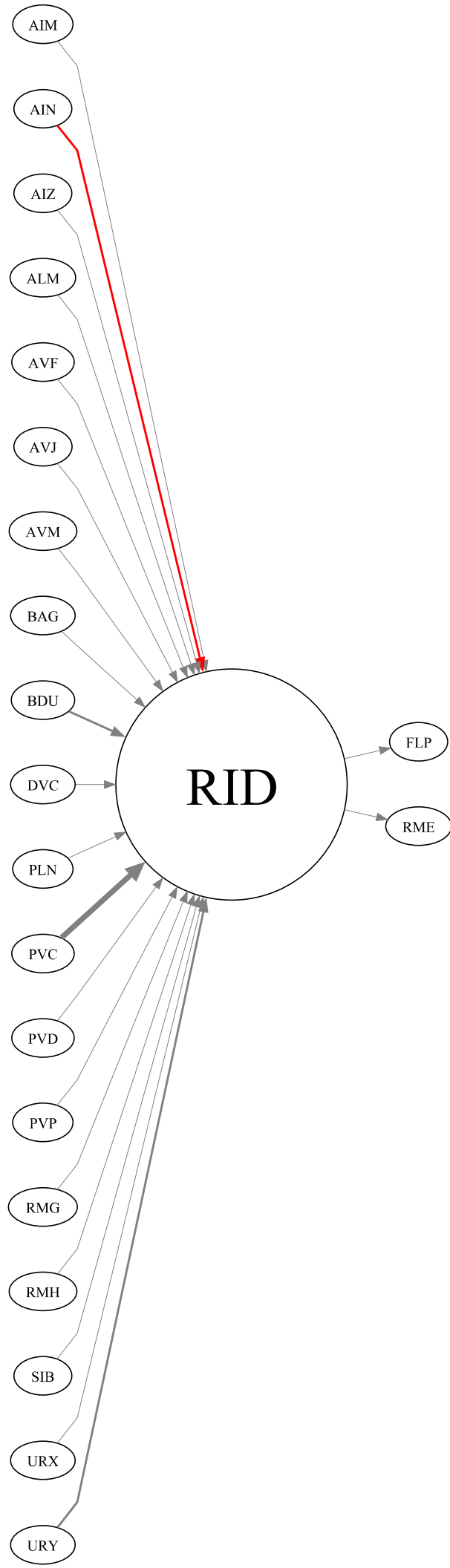

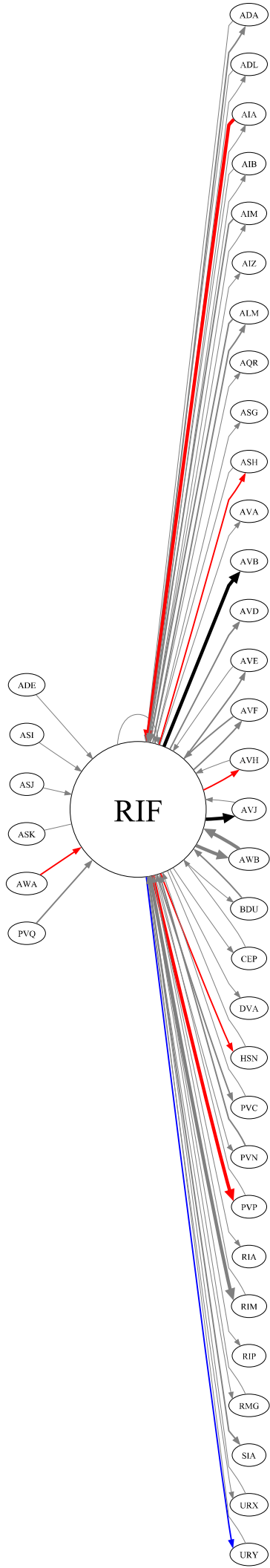

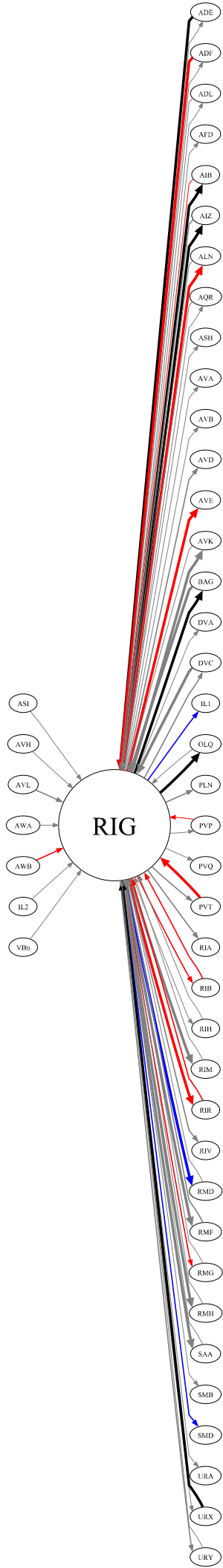

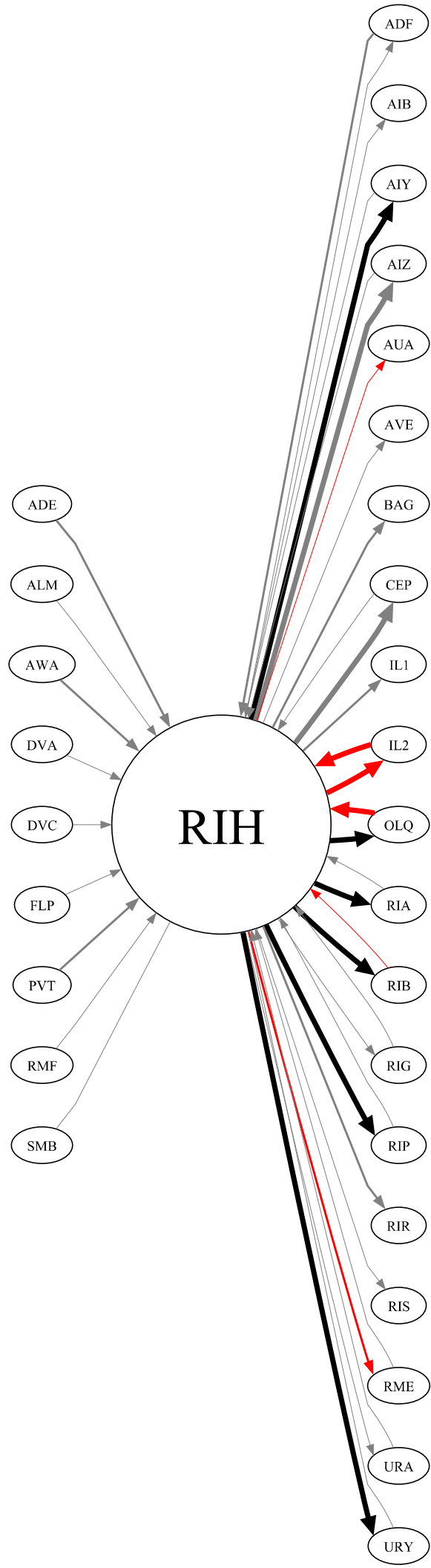

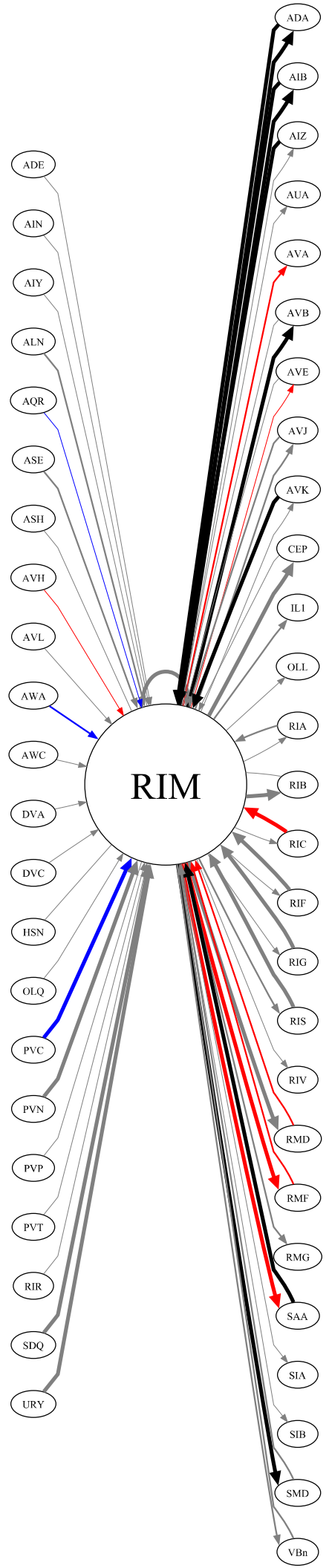

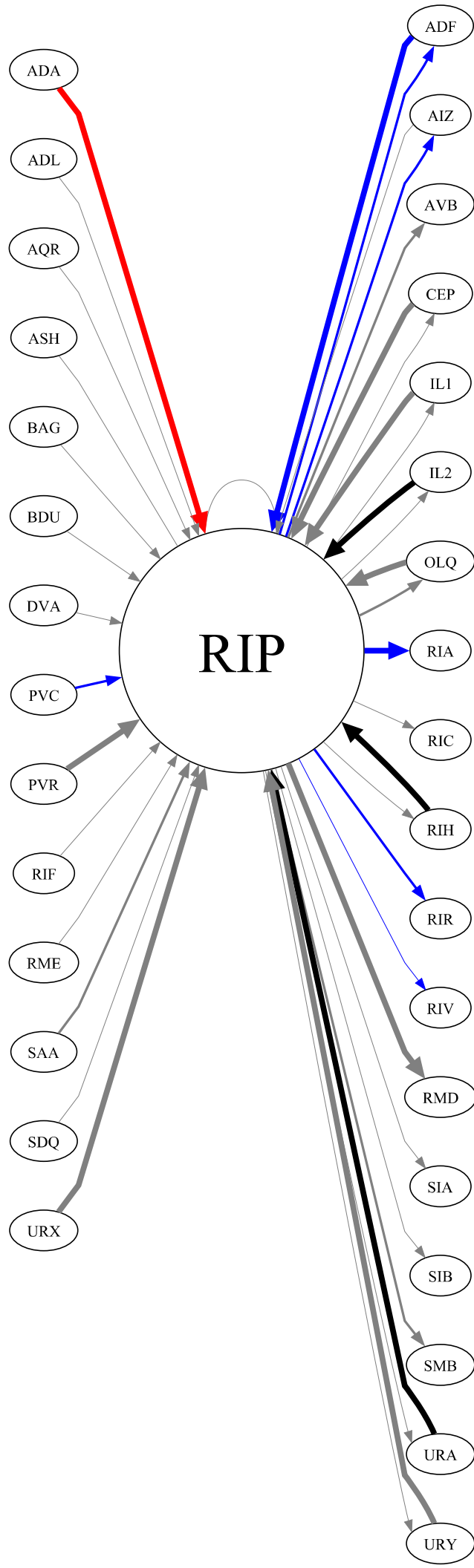

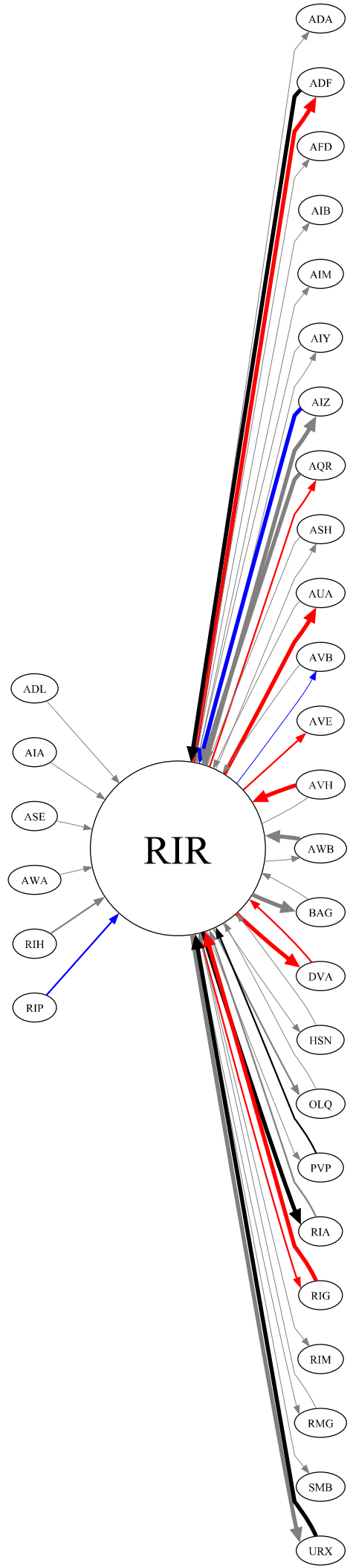

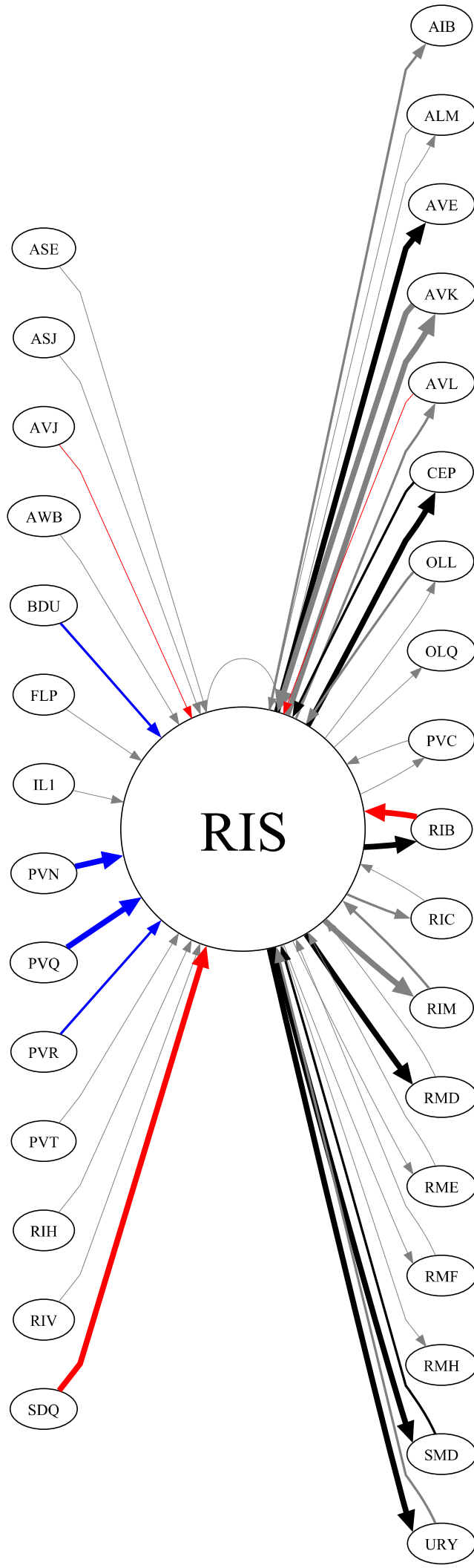

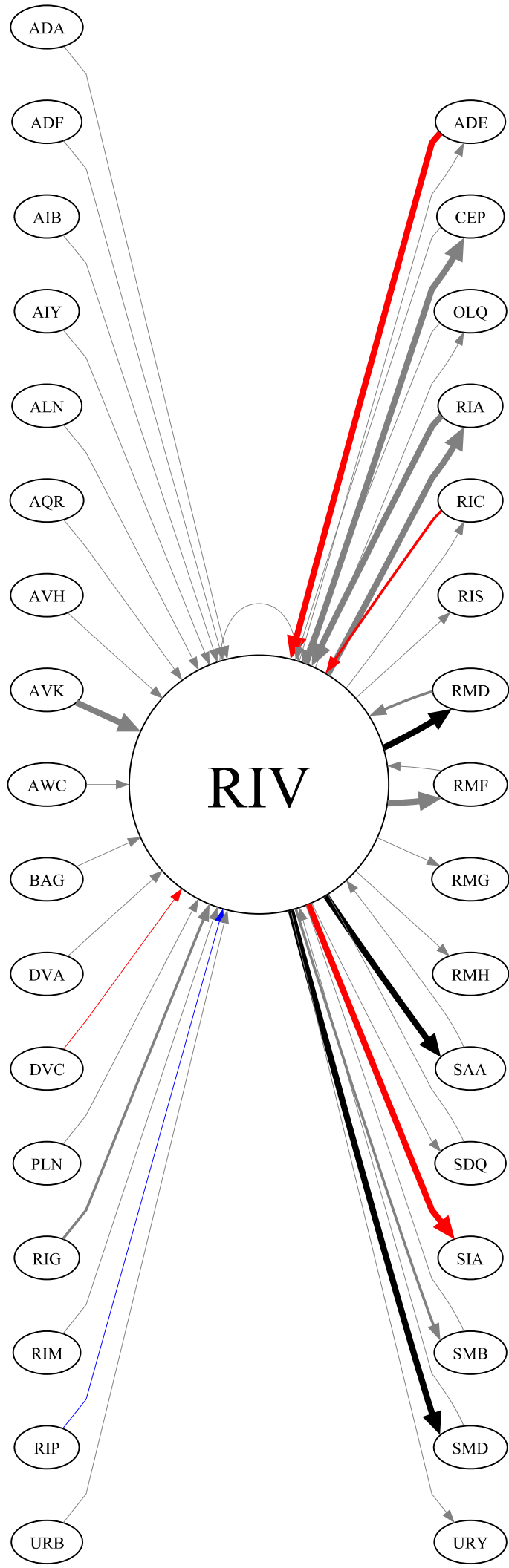

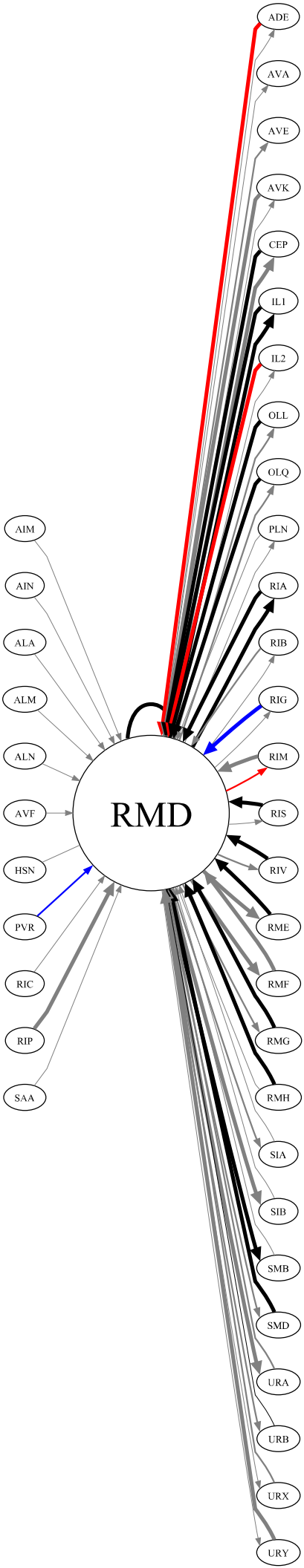

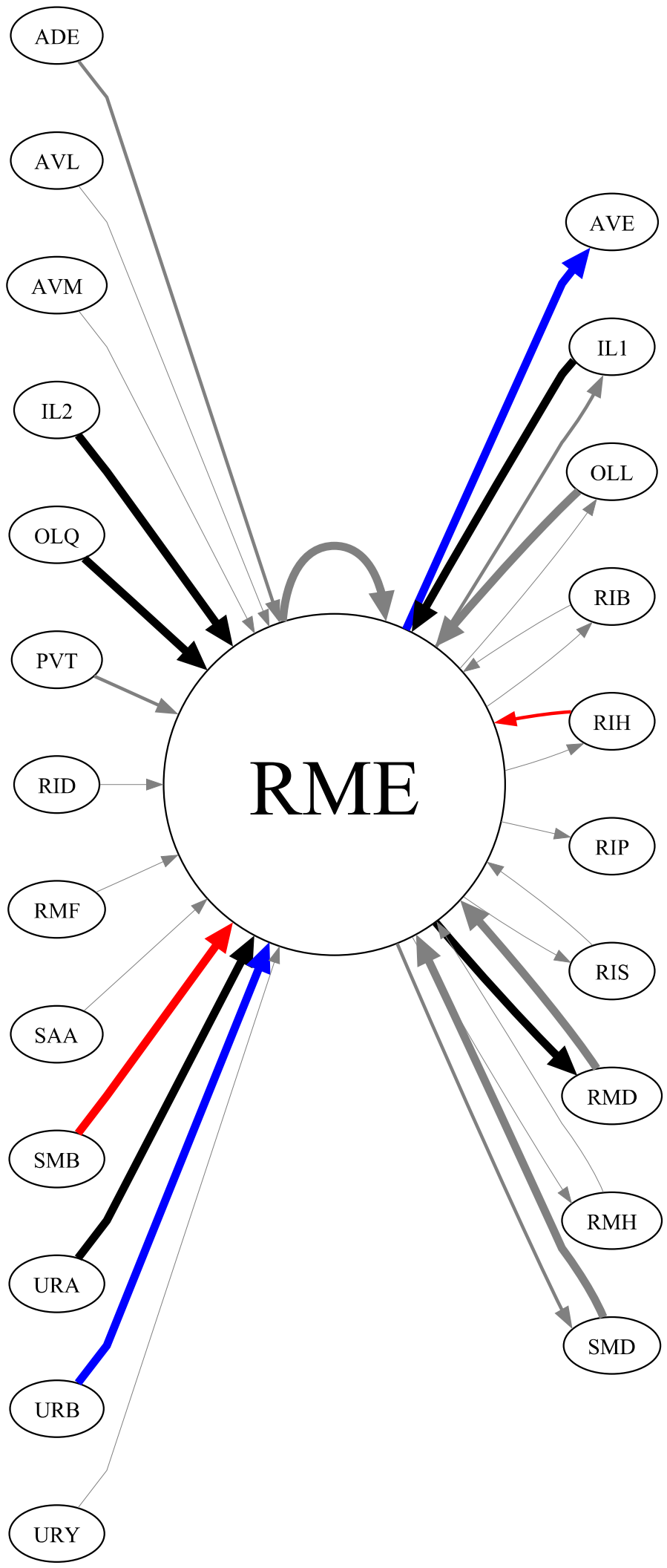

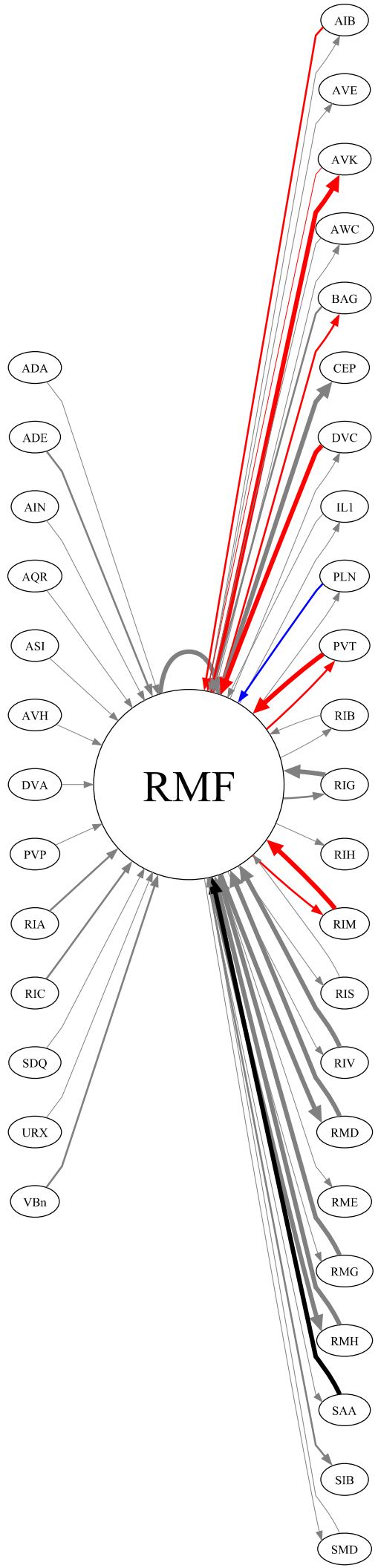

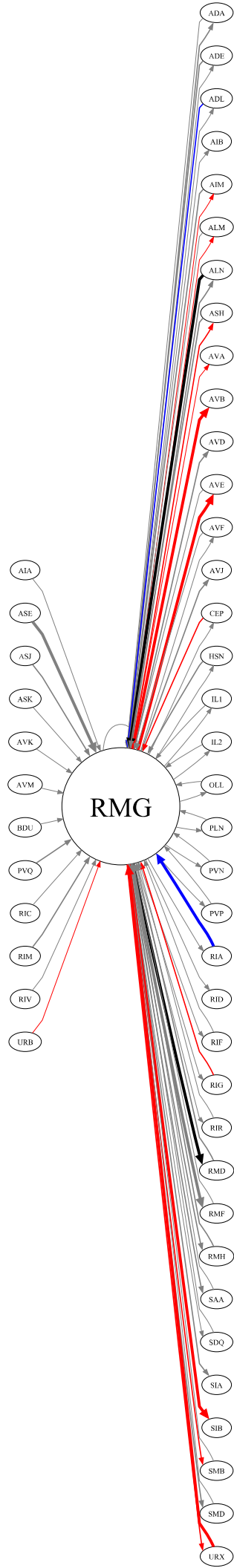

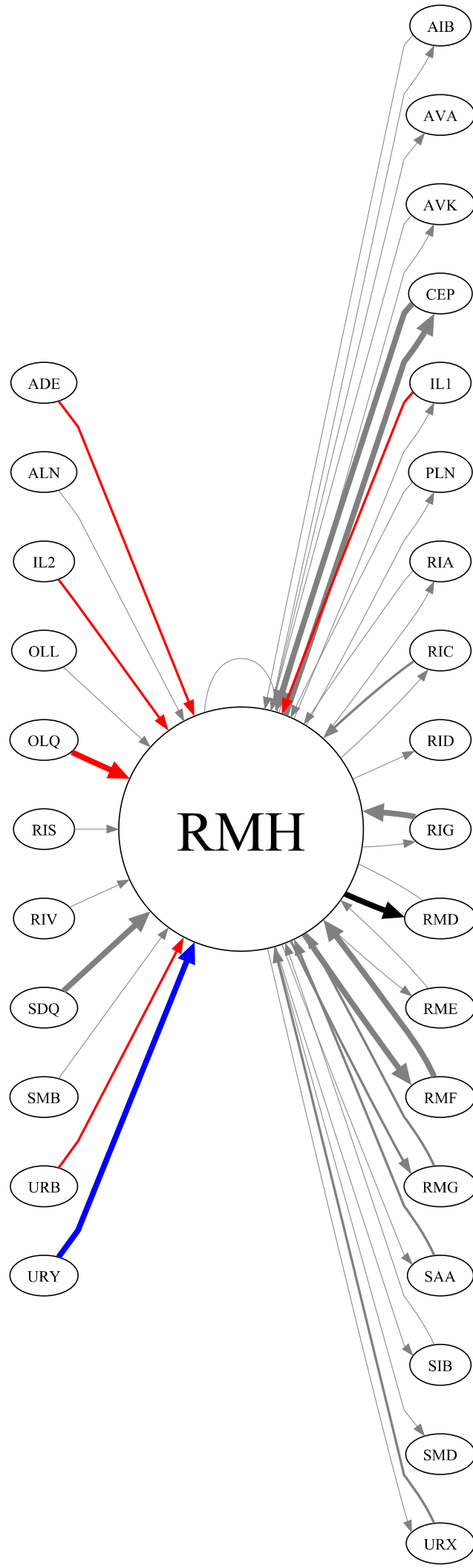

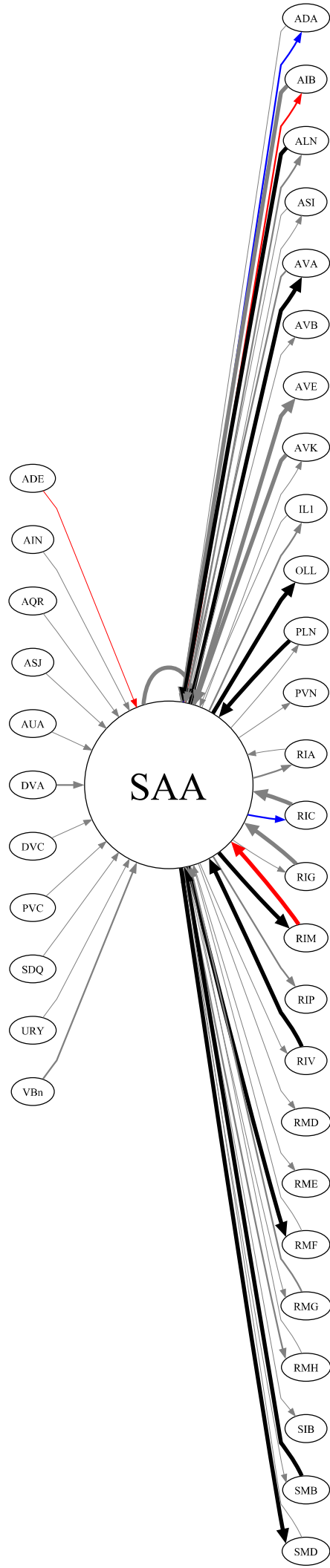

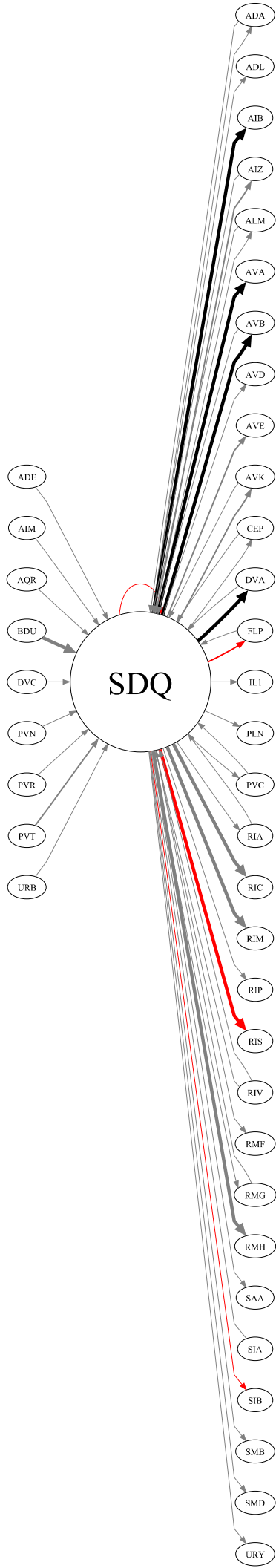

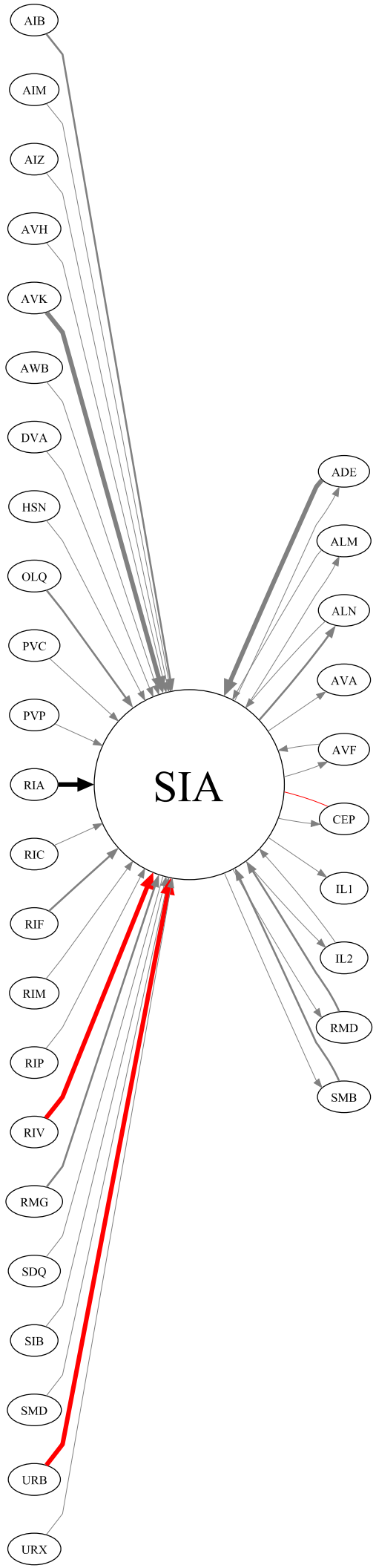

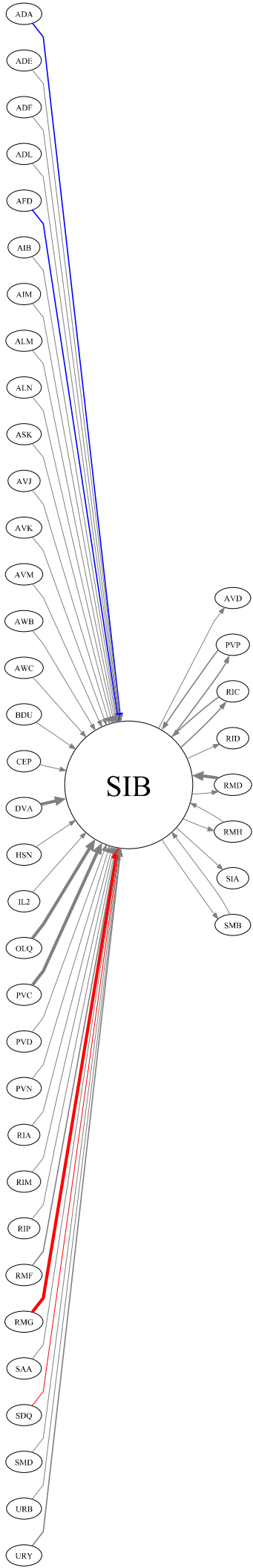

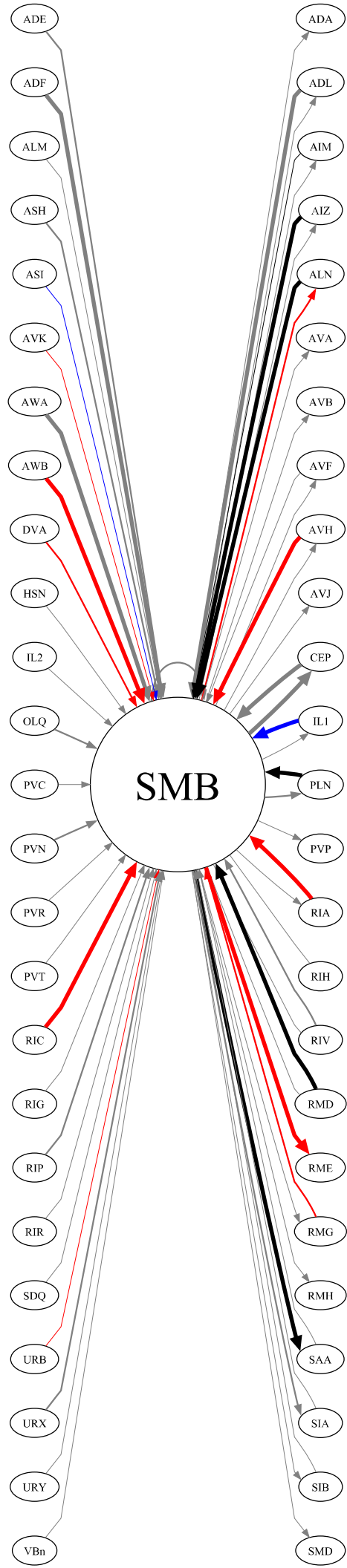

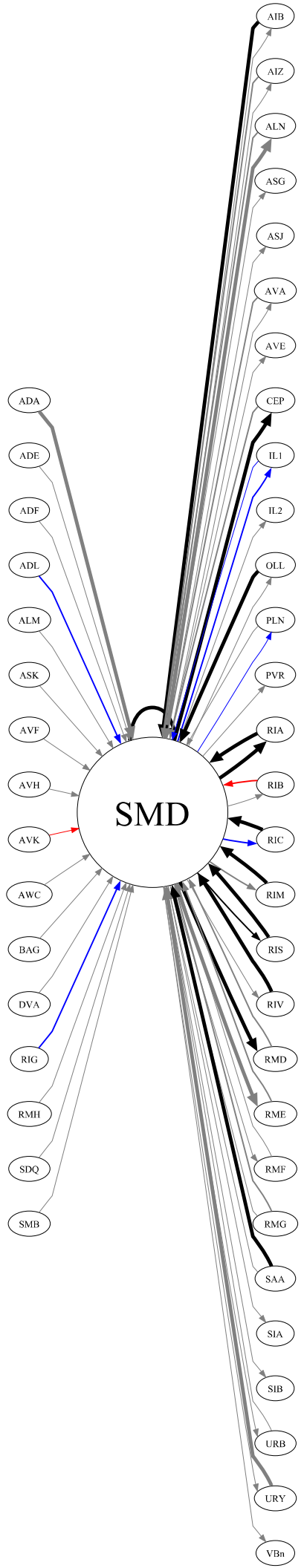

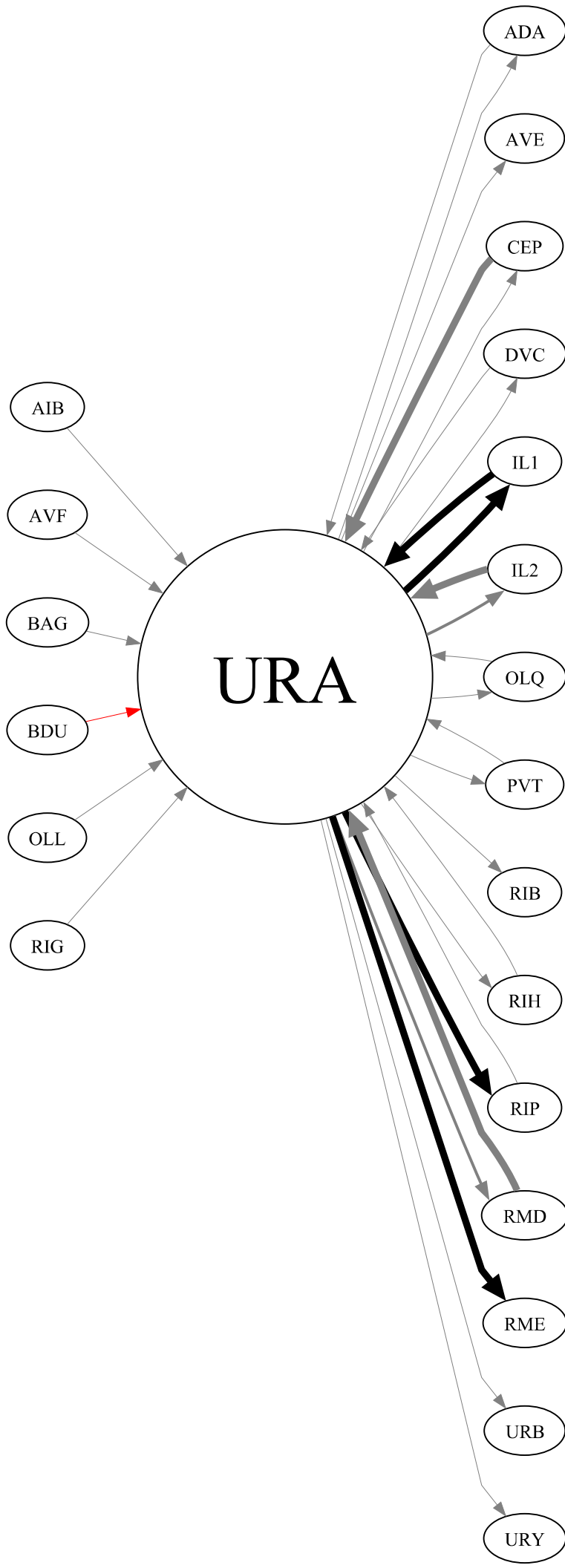

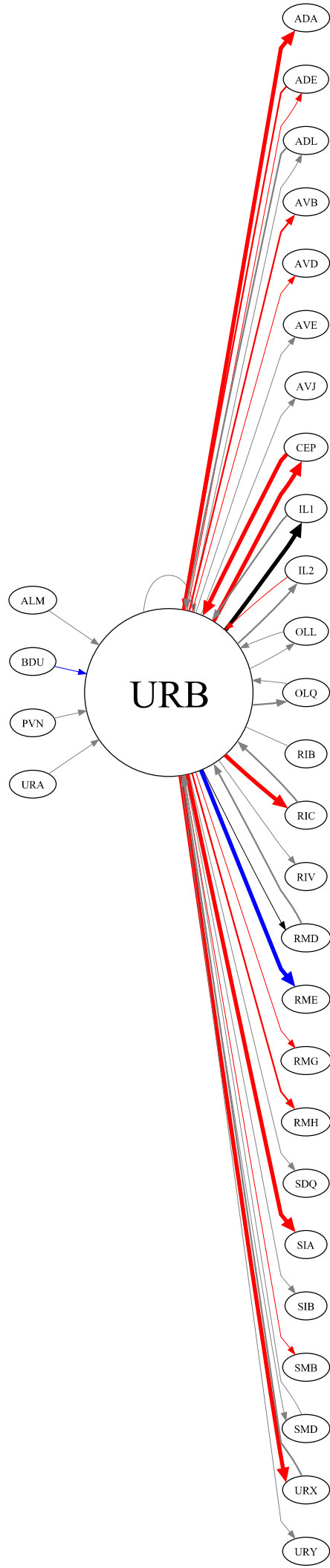

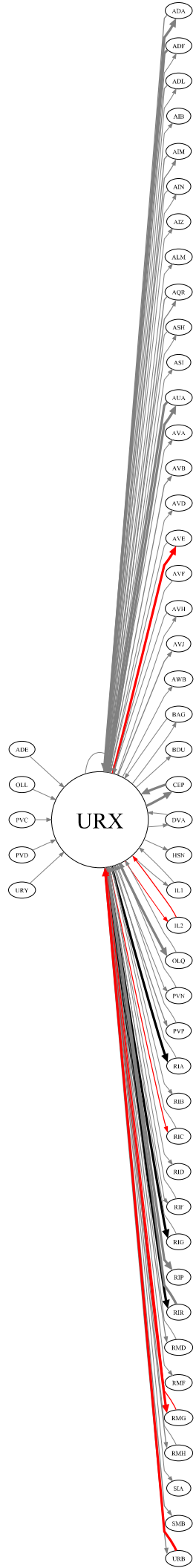

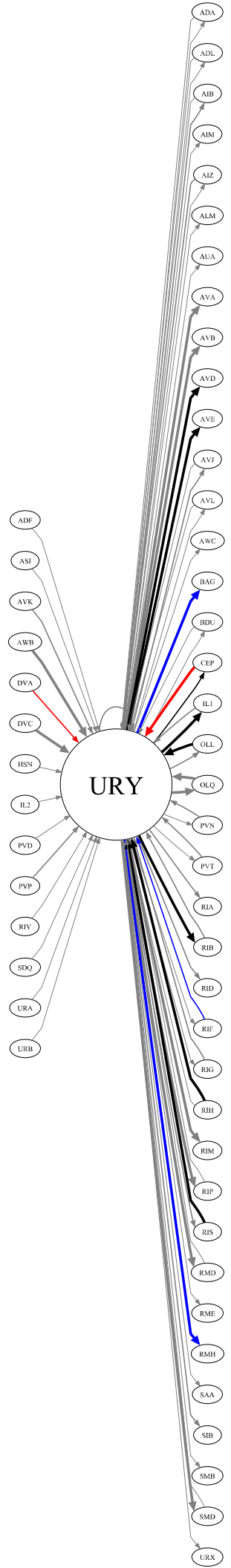

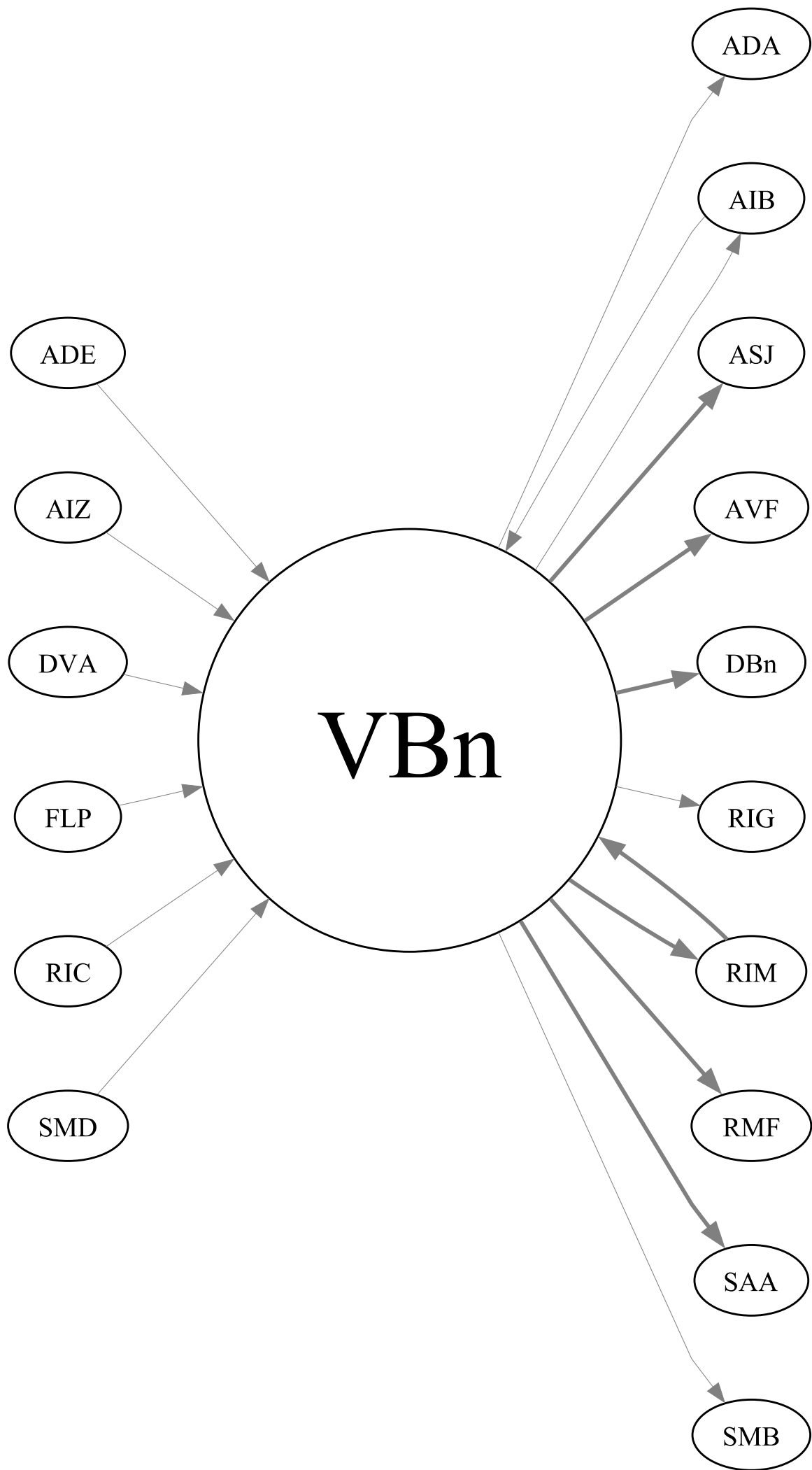

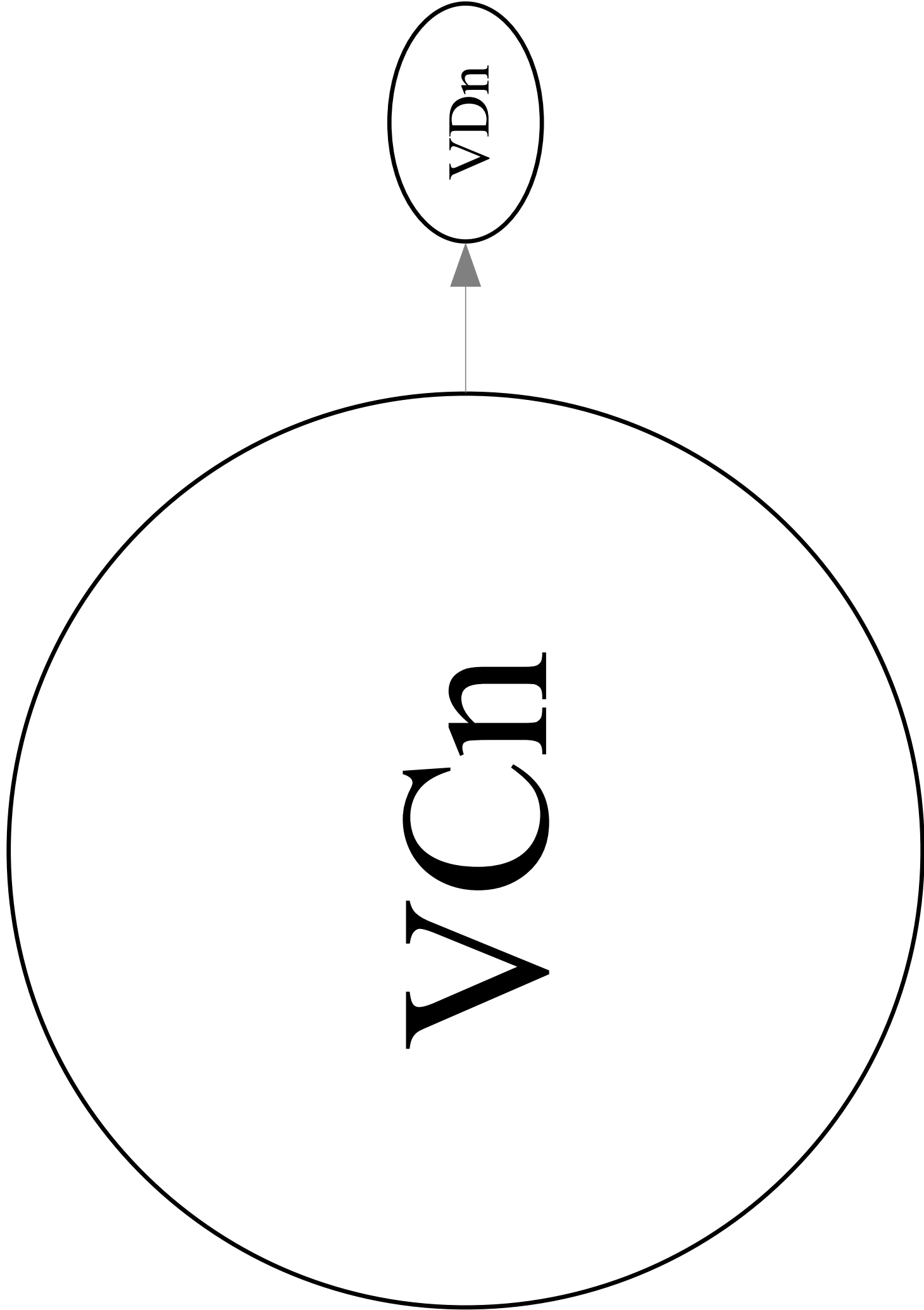

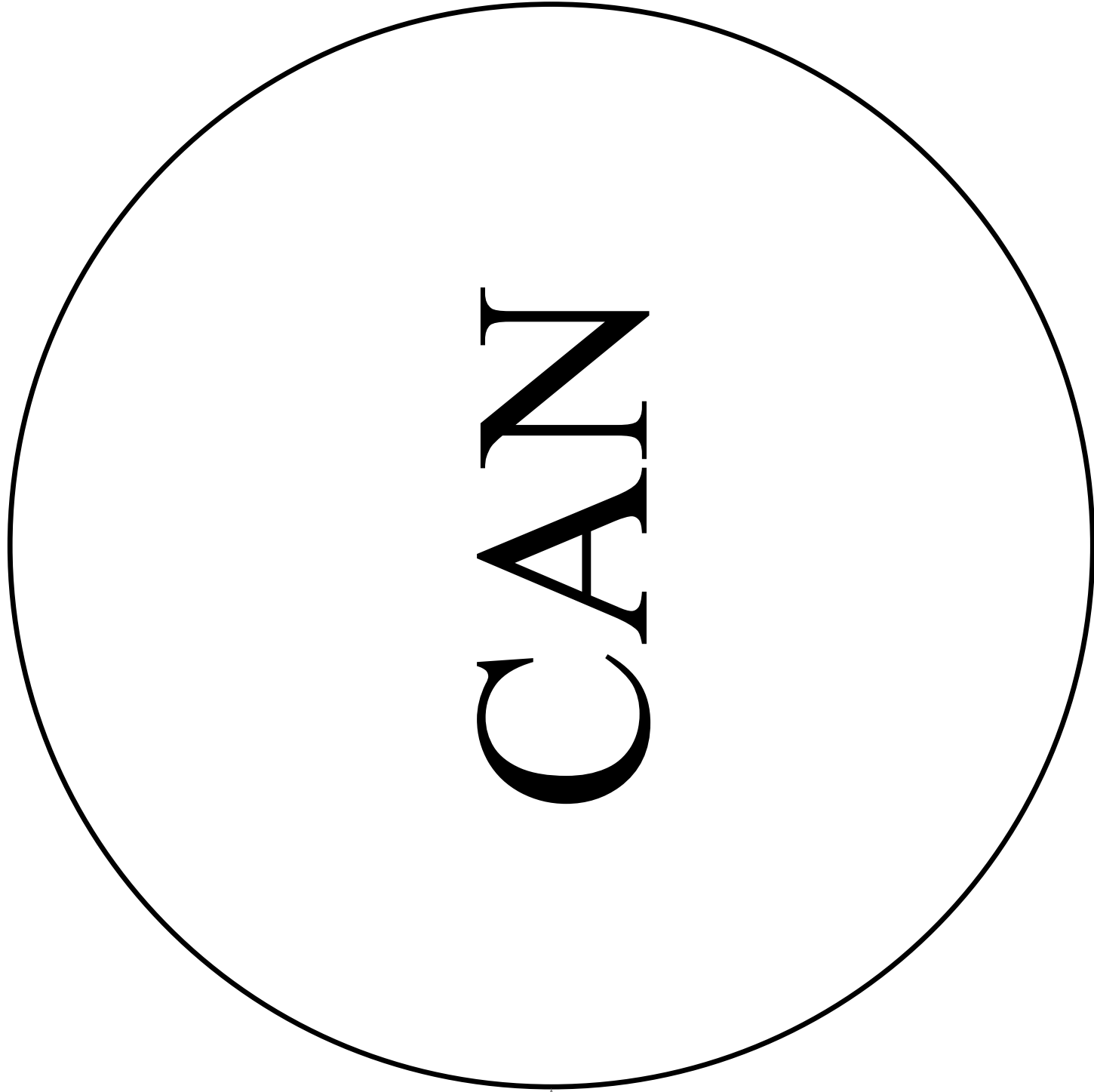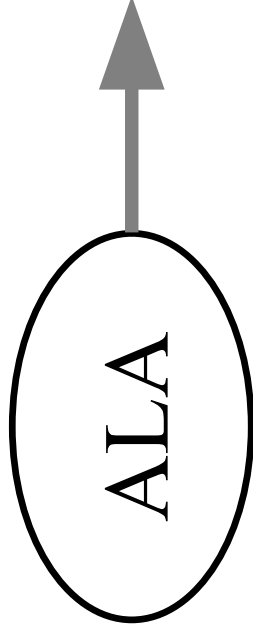

SAB

DVA

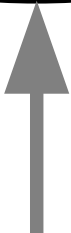

**VDn**

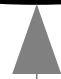

**VCn**

Supplement: Data S6 [file NIHMS2101571-supplement-Data_S6.pdf]
